# Enantio‐ and Diastereoselective Suzuki–Miyaura Coupling with Racemic Bicycles

**DOI:** 10.1002/anie.201906478

**Published:** 2019-07-25

**Authors:** F. Wieland Goetzke, Mike Mortimore, Stephen P. Fletcher

**Affiliations:** ^1^ Department of Chemistry Chemistry Research Laboratory, University of Oxford 12 Mansfield Road Oxford OX1 3TA UK; ^2^ Vertex Pharmaceuticals (Europe) Ltd 86–88 Jubilee Avenue, Milton Park Abingdon Oxfordshire OX14 4RW UK

**Keywords:** asymmetric catalysis, bicycles, boronic acids, rhodium, Suzuki–Miyaura coupling

## Abstract

Herein, we describe a rhodium‐catalyzed enantio‐ and diastereoselective Suzuki–Miyaura cross‐coupling between racemic fused bicyclic allylic chlorides and boronic acids. The highly stereoselective transformation allows for the coupling of aryl, heteroaryl, and alkenyl boronic acids and gives access to functionalized bicyclic cyclopentenes, which can be converted into other five‐membered‐ring scaffolds with up to five contiguous stereocenters. Preliminary mechanistic studies suggest that these reactions occur with overall retention of the relative stereochemistry and are enantioconvergent for pseudo‐symmetric allylic chloride starting materials. In addition, a bicyclic allylic chloride starting material without pseudo‐symmetry undergoes a highly enantioselective regiodivergent reaction.

Conventional cross‐coupling procedures are powerful tools for the construction of C(sp^2^)−C(sp^2^) bonds.^1^ Asymmetric transition‐metal‐catalyzed cross‐coupling reactions that form C(sp^3^)‐hybridized stereocenters are significantly less developed,^2^ despite being highly desired for drug development.^3^ In this context, complex bicyclic and polycyclic structures with multiple C(sp^3^) chiral centers are attractive molecular targets. Baran and co‐workers have recently reported on a modular cycloaddition/desymmetrization/cross‐coupling approach towards bridged bicyclic structures (Figure 1),^4^ and Lautens and co‐workers have developed a rhodium‐catalyzed hydroarylation of strained bicyclic alkenes (Figure 1).^5^ Both methods desymmetrize prochiral *meso* starting materials and produce multiple stereogenic centers in a single reaction step.

**Figure 1:**
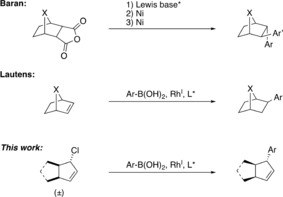
Selected examples of the enantioselective arylation of bicyclic structures. The approaches of Baran and Lautens rely on asymmetric additions to prochiral bicycles. This work reports an asymmetric addition to racemic bicycles containing multiple stereogenic centers.

Asymmetric methods that generate highly enantioenriched chiral molecules from racemic substrates are inherently attractive as there are theoretically more chiral than prochiral molecules.^6^, ^7^ While kinetic resolutions (KRs) are well explored, these procedures suffer from the intrinsic drawback of yields lower than 50 %.^7^ Dynamic asymmetric processes that convert racemic mixtures of starting materials into highly enantioenriched products—theoretically quantitatively—are significantly less developed.^8^ Several powerful enantioconvergent asymmetric allylic substitution reactions that use stabilized nucleophiles (p*K*_a_<25) have been developed with different transition metals.^9^ Using non‐stabilized nucleophiles (p*K*_a_>25) in enantioconvergent asymmetric allylic alkylation was found to be much more challenging and has only recently been reported.^10^, ^11^, ^12^ Building on this work, we reported highly enantioselective rhodium‐catalyzed Suzuki–Miyaura‐type couplings between racemic cyclic allylic halides with sp^2^‐hybridized boronic acid derivatives.^13^ These procedures allow the coupling of functionalized and heterocyclic coupling partners, a powerful strategy for drug development,^14^ and are practical as they share many of the same features that make standard Suzuki–Miyaura reactions popular; they use experimentally convenient and often commercially available boronic acid derivatives.^15^

In order to help address the longstanding need for cross‐coupling products with increased three‐dimensional shape in the pharmaceutical industry,^14^ we wanted to examine bicyclic allylic halides as substrates for rhodium‐catalyzed asymmetric arylation. The development of such a procedure would allow a modular cross‐coupling between two components and absolute control over multiple stereogenic centers in a single reaction step. Challenges in developing the method include how to achieve high enantio‐ and diastereoselectivity from addition to two different chiral species, and the inherent steric congestion of 1,2,3‐trisubstitued cyclopentenes, which makes this transformation challenging.^16^ Comparable transformations of bicyclic starting materials have only been reported using heteroatom nucleophiles in combination with palladium catalysis.^17^

Herein, we present an enantio‐ and diastereoselective rhodium‐catalyzed Suzuki–Miyaura‐type coupling with racemic bicyclic allylic chlorides and discuss mechanistic aspects of this reaction.

Using bicyclic allylic chloride (±)‐**1** as a model compound, we examined conditions similar to those reported for the coupling of 1‐chlorocyclohex‐2‐ene and aryl boronic acids.^13^ (*S*)‐**L1** and (*S*)‐**L2** in combination with [Rh(cod)OH]_2_ and Cs_2_CO_3_ in THF at 65 °C afforded the product **3 aa** with moderate enantioselectivity and incomplete conversion (see Table 1, entries 1 and 2). The products were obtained with relative *trans* stereochemistry. Changing the base to aqueous CsOH (50 wt %) improved the conversion significantly (entries 2 and 3). Different bidentate phosphine ligands were tested (entries 3–11), and excellent enantioselectivities and yields were only obtained with SEGPHOS‐type ligands. (*S*)‐**L8** and (*S*)‐**L9** gave comparable results whereas more sterically demanding (*S*)‐**L10** gave low conversion and lower enantioselectivity. Ligand (*S*)‐**L8** was chosen for further experiments. The reaction is highly diastereoselective, and we were only able to observe trace amounts of the minor diastereomer (dr>20:1) in crude reaction mixtures.

**Table 1:** Selected optimization experiments. 
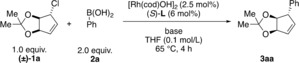

Entry	Ligand	Base	Yield [%]^[a]^	ee [%]^[c]^
1	**L1**	Cs_2_CO_3_	62	78
2	**L2**	Cs_2_CO_3_	65	76
3	**L2**	CsOH (aq. 50 wt %)	74	76
4	**L3**	CsOH (aq. 50 wt %)	76	82
5	**L4**	CsOH (aq. 50 wt %)	92	84
6	**L5**	CsOH (aq. 50 wt %)	95	84
7	**L6**	CsOH (aq. 50 wt %)	75	80
8	**L7**	CsOH (aq. 50 wt %)	62^[b]^	86
9	**L8**	CsOH (aq. 50 wt %)	91	95
10	**L9**	CsOH (aq. 50 wt %)	88	96
11	**L10**	CsOH (aq. 50 wt %)	<20	68

[a] Isolated yields of single diastereomers (dr>20:1). [b] Isolated with 12:1 dr. [c] The ee values were determined by SFC analysis on a chiral non‐racemic stationary phase. 
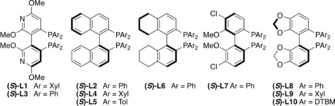

With optimized conditions in hand, we investigated the boronic acid scope (Scheme 1). A broad range of substituted aryl boronic acids gave good to excellent yields and excellent enantioselectivities. Both electron‐rich and ‐poor boronic acids as well as halogenated aryl boronic acids gave good to excellent results (Scheme 1 a, products **3aa**–**3aj**) but the present conditions are not suitable for boronic acids bearing *ortho* substituents. More challenging boronic acids bearing functional groups such as acetyl, olefin, and cyano groups as well as difluoroarenes were compatible with our reaction conditions (**3ak**–**3an**). In many cases, we observed protodeborylation^18^ and homocoupling of the boronic acid, and unreacted allylic halide was recovered. Extended reaction times generally do not improve the yields of the coupling products.

**Scheme 1:**
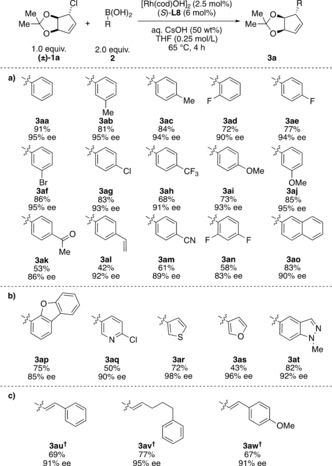
Rhodium‐catalyzed asymmetric cross‐coupling of (±)‐**1 a** with a) aryl boronic acids, b) heteroaryl boronic acids, and c) alkenyl boronic acids. All experiments were performed on 0.4 mmol scale. All compounds were isolated as single diastereomers (dr>20:1). Enantiomeric ratios were determined by SFC analysis on a chiral non‐racemic stationary phase. † Ligand (*S*)‐**L2** used instead.

Heteroaryl and alkenyl boronic acids are known to be challenging coupling partners in Suzuki–Miyaura couplings because of rapid protodeborylation.^18^ Under our conditions, heteroaryl boronic acids, including 2‐chloropyridine, 3‐thiophene, and an azaindole, were well tolerated, with good yields and excellent enantioselectivities (Scheme 1 b). For alkenyl boronic acids including (*E*)‐styrene and (*E*)‐alkenyl boronic acids ligand **L2** was used in order to achieve higher levels of stereoselectivity (Scheme 1 c).^19^

We subsequently prepared additional coupling partners that are pseudo‐symmetric about the allyl chloride unit featuring a fused bicyclo[2.2.1]heptane ((±)‐**1 b**), a sulfamide ((±)‐**1 c**), and a pyrrolidine ((±)‐**1 d**; Scheme 2, for the synthesis of the starting materials see the Supporting Information). These substrates required only minimal alterations from the above reaction conditions to achieve excellent results. For each substrate (Scheme 2), we examined several coupling partners and found that these allowed for absolute control over multiple stereogenic centers in a single reaction step. Allylic chloride (±)‐**1 b** showed competing hydrolysis under the reaction conditions but still allowed for highly stereoselective coupling reactions in moderate to good yields.^20^ The nitrogen‐containing allylic chlorides (±)‐**1 c** and (±)‐**1 d** required slightly increased temperature (heating to 80 °C in a sealed flask) to achieve full conversion with phenylboronic acid and consistently gave excellent enantioselectivities.^21^

**Scheme 2:**
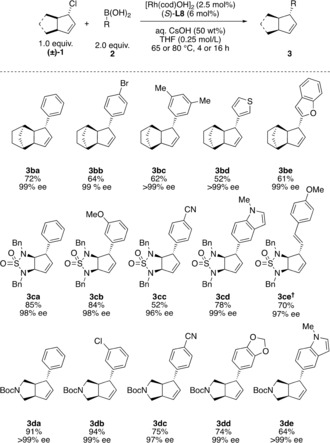
Rhodium‐catalyzed asymmetric cross‐coupling of different allylic chlorides. All experiments were performed on 0.4 mmol scale. All compounds were isolated as single diastereomers (dr>20:1). Enantiomeric ratios were determined by SFC analysis on a chiral non‐racemic stationary phase. † Ligand (*S*)‐**L2** used instead.

Our protocol is very robust and is easily scalable with almost identical results obtained on a 6.0 mmol scale (Scheme 3 a) to give 1.2 g of coupled product. The downstream reactivity of the obtained products was also investigated (Scheme 3 b). The cyclic acetal group of **3 aa** and the sulfamide of **3 ca** can be cleaved to give cyclopentenes bearing vicinal diols (**4**) and amines (**5**) with three defined stereogenic centers in high yields. This illustrates the utility of a fused bicyclic protecting group in a synthetic sequence. Furthermore, **3 aa** was dihydroxylated to give cyclopentene **6** with five contiguous stereocenters.

**Scheme 3:**
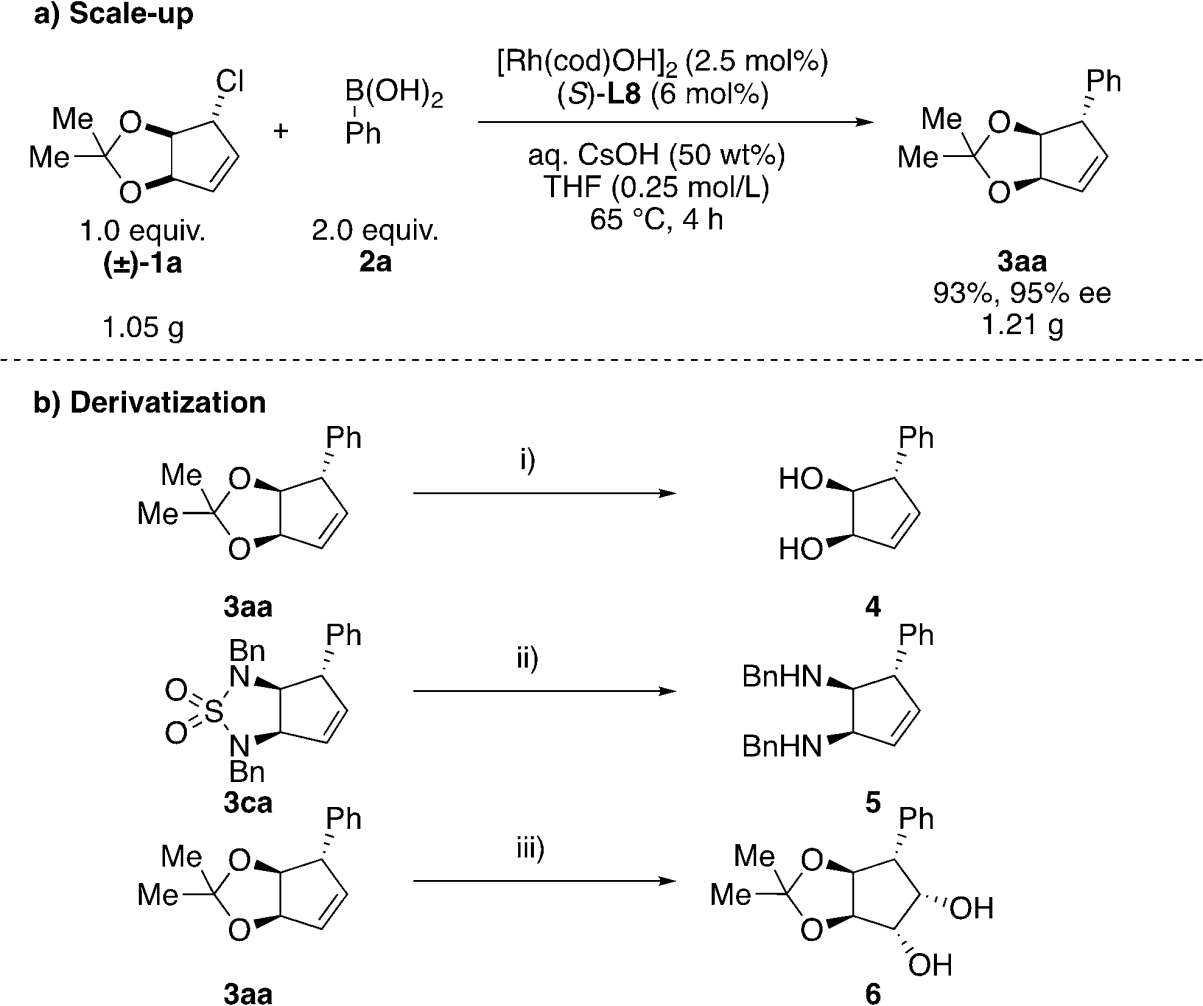
a) Scale‐up experiment and b) derivatization of the obtained products. Conditions: i) AcOH/H_2_O (7:3), 40 °C, 24 h, 98 %; ii) LiAlH_4_, Et_2_O, 16 h, reflux, 93 %; iii) K_2_OsO_4_⋅H_2_O (4 mol %), NMO, acetone/H_2_O (7:3), room temperature, 18 h, 91 % (dr 4:1).

In order to gain a better understanding of the observed process of enantioconvergence, we monitored the enantiomeric excess of allylic chloride **1 a** and product **3 aa** over time at 40 °C (Scheme 4 a).^22^ While the ee of **3 aa** was constant, a kinetic resolution of the racemic allylic chloride occurred, with enantiomerically pure starting material being observed at about 75 % conversion. However, there is no apparent change in the rate of product formation with the observed resolution at about 50 % conversion, and so the step that resolves the allyl chloride is unlikely to be the rate‐determining step.

**Scheme 4:**
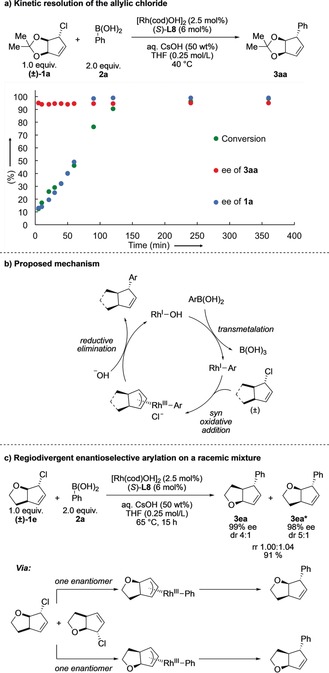
a) Kinetic resolution of **1 a**. b) Proposed mechanism. c) Regioselective catalytic enantioselective arylation of a racemic mixture (for experimental details see the Supporting Information).

We propose the following mechanism in order to explain the enantioconvergence, resolution of starting material, and retention of the relative stereochemistry in the reaction (Scheme 4 b). Following transmetalation of the aryl boronic acid to a Rh^I^–hydroxide complex,^23^ oxidative addition of the allylic chloride occurs via insertion into the C−Cl bond or a *syn* S_N_2′‐type mechanism. As reductive elimination also occurs *syn*, an overall retentive process is observed, maintaining the relative *trans* stereochemistry between the fused ring and the chloride in the starting material and the arene substituent in the product. This mechanism is different to those proposed in previous studies on Rh‐catalyzed arylations of cyclic allylic halides where *anti* oxidative addition was believed to occur.^13^ We propose that both enantiomers of the allylic chloride give the same pseudo‐*meso* Rh–π‐allyl complex.

Based on this mechanistic proposal, we expected that only a pseudo‐symmetric bicyclic allylic halide (structures with a corresponding *meso* carbocation) could converge to a single isomer of desired product. To test this hypothesis, we subjected (±)‐**1 e** as a single regioisomer to our standard reaction conditions (Scheme 4 c). Two regioisomers were formed in a 1:1 ratio with excellent enantiocontrol (98 and 99 % ee) but with decreased diastereoselectivity (*trans* isomers favored over *cis* by a 4:1 and 5:1 ratio). This process is an enantioselective regiodivergent reaction of a racemic mixture where a single catalyst is used to convert each enantiomer of the starting material into a different product with high ee.^24^

Regiodivergent reactions of racemic mixtures differ from parallel kinetic resolutions (PKRs), where two different chiral agents or catalysts react with the two different enantiomers. Here, the complete absence of any matched/mismatched effects, which would lower the ee of one of the formed regioisomers, is remarkable.^25^ Such an asymmetric regiodivergent reaction could potentially be a useful strategy in the synthesis of compound libraries and gives easy access to enantiopure compounds. In addition, this experiment suggests that sterically demanding groups about the allylic chloride are required to achieve the excellent levels of *trans/cis* diastereoselectivity observed with substrates **1 a**–**1 d**.

In summary, we have developed a highly enantio‐ and diastereoselective Suzuki–Miyaura cross‐coupling reaction between bicyclic allylic chlorides and boronic acids. The method gives access to a broad range of valuable, highly functionalized, and novel aryl‐, heteroaryl‐, and alkenyl‐substituted fused‐bicyclic cyclopentenes. Preliminary mechanistic experiments suggest that *syn*‐selective oxidative addition to form a pseudo‐*meso* endocyclic allylic rhodium species occurs, followed by rate‐ and enantiodetermining reductive elimination, to give a single enantiomer of product from racemic starting materials with overall retention of the relative configuration. This approach provides access to complex chiral structures in a modular way, and we envision its ready application in target synthesis and drug development.

## Conflict of interest

Oxford University Innovation has filed a patent application (PCT/GB2016/051612) with S.P.F. named as an inventor. F.W.G. and M.M. declare no competing financial interests.

## Supporting information

As a service to our authors and readers, this journal provides supporting information supplied by the authors. Such materials are peer reviewed and may be re‐organized for online delivery, but are not copy‐edited or typeset. Technical support issues arising from supporting information (other than missing files) should be addressed to the authors.

Supplementary
