# Supplementary material for: Enantio‐ and Diastereoselective Suzuki–Miyaura Coupling with Racemic Bicycles
Source: Angew Chem Int Ed Engl. 2019 Jul 25;58(35):12128–32. doi: 10.1002/anie.201906478 (PMC6771587; doi:10.1002/anie.201906478)
Supplement: Supplementary file 1 — Supplementary [file ANIE-58-12128-s001.pdf]

Angewandte Chemie
Eine Zeitschrift der Gesellschaft Deutscher Chemiker

Supporting Information

Enantio- and Diastereoselective Suzuki–Miyaura Coupling with Racemic Bicycles

*F. Wieland Goetzke, Mike Mortimore, and Stephen P. Fletcher**

anie_201906478_sm_miscellaneous_information.pdf

Table of Contents

1. Experimental Procedures (p. 3-33)
1.1. General Methods (p. 3)
1.2. Procedures for the Synthesis of the Starting Materials (p. 4-11)
1.3. Procedures for the Asymmetric Synthesis (p. 11-32)
1.4. Derivatization Experiments (p. 33-34)
1.5. Mechanistic Studies (p. 34)
2. References (p. 35)
3. NMR spectra (p. 36-98)
4. SFC traces (p. 99-138)

Experimental Procedures

1.1 General Methods

All reactions were carried out in flame-dried glassware, in anhydrous solvents with continuous magnetic stirring under an inert argon atmosphere. Heating was performed using DrySyn heating blocks.

Nuclear magnetic resonance (NMR) spectroscopy measurements were carried out at room temperature. ^{1}H NMR, ^{13}C NMR, ^{19}F NMR, COSY, HSQC, HMBC and NOESY experiments were carried out using Bruker AVG-400 (400/100 MHz), AVH-400 (400/100 MHz), AVX-500 (500/125 MHz) or AVC-500 (500/125 MHz) spectrometers. Chemical shifts (δ) are reported in ppm relative to the residual solvent peak with corresponding coupling constants (*J*) in Hertz (Hz) and multiplicities (s: singlet, d: doublet, t: triplet, q: quartet, m: multiplet and combinations of these and app.: apparent multiplicities). Assignment follows HSQC, COSY, HMBC or/and NOESY spectra, chemical shift and coupling constant analysis.

Optical rotations ($[\alpha]_{20}^{D}$) were recorded using a Perkin Elmer-241 Polarimeter. Concentrations (*c*) are reported in g/100 mL.

Infrared (IR, neat or thin film) spectroscopy was carried out on a Bruker Tensor 27 FT–IR spectrometer with in internal calibration range of 4000 – 600 cm^{-1}. The samples are reported as absorption maxima in cm^{-1} with corresponding relative intensities described as br (broad), s (strong), m (medium), and w (weak).

Chiral SFC (supercritical fluid chromatography) separations were conducted on a Waters Acquity UPC2 system using Waters Empower software. Chiralpak® columns (150×3 mm, particle size 3 μm) were used as specified in the text. Solvents used were of HPLC grade (Fisher Scientific, Sigma Aldrich or Rathburn).

Chiral GC measurements were conducted on an Agilent 7820A GC (He as a vector gas) with the stated column in the characterization. Temperature programs are described as follows: initial temperature (°C) – initial time (min) – temperature gradient (°C/min) – final temperature (°C) – holding time (min). Flow rate is given in mL/min and retention times (R_T) are given in min.

High Resolution Mass spectra were carried out by internal service at the University of Oxford. (1) Electron spray ionisation (ESI^+) were recorded on a Fisons Platform II. (2) Electron ionisation (EI)/Chemical ionisation (CI): Analyses were performed on an Agilent 7200 quadrupole time of flight (Q-ToF) instrument equipped with a direct insertion probe supplied by Scientific instrument Manufacturer (SIM) GmbH. (3) Atmospheric pressure chemical ionisation ($APCI^+$): Analyses were performed using a Thermo Exactive mass spectrometer equipped with Waters Acquity liquid chromatography system.

Commercially available reagents and ligands were purchased from Sigma Aldrich, Alfa Aesar, Acros Organics, Fluorochem and Strem Chemicals and unless otherwise stated were used without further purification. $[Rh(cod)OH]_2$ was bought from Sigma Aldrich and Strem Chemicals or synthesized according to a literature procedure[1]. All aryl and heteroarylboronic acids were used without additional purification. Vinylboronic acids were prepared according to a literature procedure[2]. Dry solvents were collected fresh from an mBraun SPS–800 solvent purification system after having passed through anhydrous alumina columns. Deuterated solvents were purchased from Sigma Aldrich.

1.2. Procedures for the Synthesis of the Starting Materials

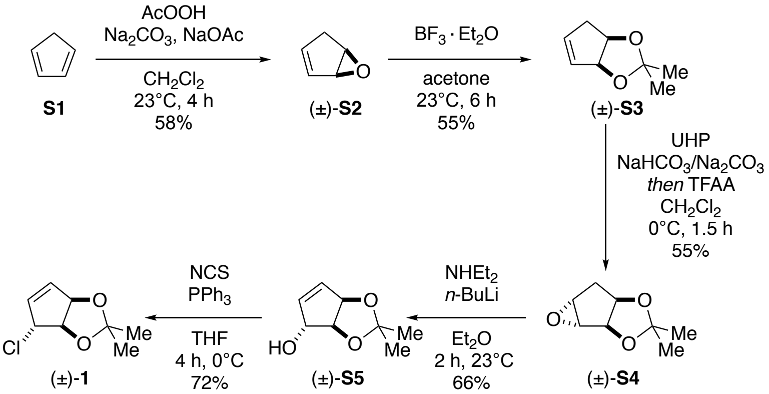

Scheme S1: Overview for the synthesis of (±)-**1a**.

(±)-S2

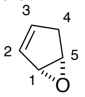

Similar to a procedure by Jung *et. al.*[3], Na_2CO_3 (99.4 g, 0.94 mol) and NaOAc (1.90 g, 23.2 mmol) were added to a solution of freshly cracked cyclopentadiene (**S1**) (30 mL, 424 mmol) in CH_2Cl_2 (470 mL). Peracetic acid (40 wt% in Acetic acid, 64 mL, 380 mmol) was added dropwise over 1 h at 0 °C under an open atmosphere. The reaction mixture was then allowed to reach 23 °C and stirring was continued for additional 3 h. The mixture was then filtered under reduced pressure, the residue was rinsed with CH_2Cl_2 and the filtrate was carefully (the product is volitile) concentrated under reduced pressure at 15 °C. Vacuum distillation (25 mbar, up to 80 °C) afforded the product (±)-**S2** as a colourless oil containing CH_2Cl_2 (estimated yield: 16.9 g, 58%). The spectroscopic data is in agreement with the literature.[3]

^{1}H NMR ($CDCl_3$, 400 MHz): δ (ppm) 6.14 (dtd, *J* = 5.6, 2.2, 1.1 Hz, 1H, C(2)–H), 5.98 (dp, *J* = 6.3, 2.1 Hz, 1H, C(3)–H), 3.91 (ddd, *J* = 3.5, 2.9, 2.1 Hz, 1H, C(5)–H), 3.81 (dtd, *J* = 3.0, 1.9, 1.1 Hz, 1H, C(1)–H), 2.63 (dq, *J* = 19.1, 2.2 Hz, 1H, C(4)–H_2), 2.39 (ddt, *J* = 19.1, 3.5, 2.1 Hz, 1H, C(4)–H_2).

^{13}C NMR ($CDCl_3$, 100 MHz) δ (ppm) 138.0 (C(3)), 131.4 (C(2)), 59.3 (C(1)), 57.0 (C(5)), 35.7 (C(4)).

(±)-S3

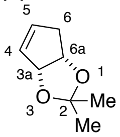

According to procedure by *Trost et. al.*,[4] $BF_3 \cdot Et_2O$ (1.2 mL, 9.7 mmol) was added dropwise to a solution of 6-oxabicyclo[3.1.0]hex-2-ene ((±)-**S2**) (8.00 g, 97.4 mmol) in dry acetone (60 mL) at 0 °C under an argon atmosphere. The reaction mixture was allowed to reach 23 °C and stirred for 6 h. The reaction mixture was concentrated under reduced pressure, dissolved in Et_2O (100 mL), washed with an aq. sat. solution of $NaHCO_3$ (2x 30 mL) and with an aq. sat. solution of NH_4Cl (2x 30 mL), and dried over Na_2SO_4. Purification by flash chromatography (pentane/Et_2O = 100/0 to 90/10) afforded the product (±)-**S3** as a colorless volatile oil (7.55 g, 55% yield). The spectroscopic data is in agreement with the literature.[4]

^{1}H NMR ($CDCl_3$, 400 MHz): δ (ppm) 5.85 – 5.81 (m, 1H, C(5)–H), 5.78 (dq, *J* = 5.9, 1.9 Hz, 1H, C(4)–H), 5.11 (d, *J* = 5.9 Hz, 1H, C(3a)–H), 4.76 (t, *J* = 5.7 Hz, 1H, C(6a)–H), 2.57 – 2.53 (m, 2H, C(6)–H_2), 1.41 (s, 3H, CH_3), 1.35 (s, 3H, CH_3).

^{13}C NMR ($CDCl_3$, 100 MHz) δ (ppm) 132.5 (C(5)), 130.9 (C(4)), 109.9 (C(2)), 85.6 (C(3a)), 77.9 (C(6a)), 39.0 (C(6)), 27.6 (CH_3), 25.8 (CH_3).

(±)-S4

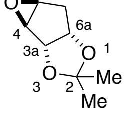

According to procedure by *Trost et. al.*,[4] $NaHCO_3$ (1.89 g, 22.5 mmol), Na_2CO_3 (7.16 g, 67.5 mmol) and urea hyrdogen peroxide (UHP) (10.6 g, 113 mmol) were added to a solution of (±)-**S3** (3.15 g, 22.5 mmol) in CH_2Cl_2 (225 mL) under an argon atmosphere. A solution of trifluoroacetic anhydride (7.8 mL, 56.3 mmol) in CH_2Cl_2 (105 mL) was added dropwise at 0 °C. The mixture was stirred for 3 h, washed with an aq. sat. solution of $NaHCO_3$ (3x 50 mL), and dried over Na_2SO_4. Purification by flash chromatography

(pentane/Et_2O = 90/10 to 70/30) afforded the product (±)-**S4** as a colorless volatile oil (1.94 g, 55% yield) as single diastreomer (dr >20:1). The spectroscopic data is in agreement with the literature.[4]

^{1}H NMR ($CDCl_3$, 400 MHz): δ (ppm) 4.57 (d, *J* = 5.5 Hz, 1H, C(3a)–H), 4.53 (ddt, *J* = 6.4, 4.6, 1.0 Hz, 1H, C(6a)–H), 3.68 – 3.47 (m, 2H, C(4)–H and C(5)–H), 2.28 (dd, *J* = 15.4, 6.0 Hz, 1H, C(6)–H_2), 1.95 (dt, *J* = 15.3, 2.0 Hz, 1H, C(6)–H_2), 1.46 (s, 3H, CH_3), 1.31 (s, 3H, CH_3).

^{13}C NMR ($CDCl_3$, 100 MHz) δ (ppm) 111.95 (C(2)), 81.5 (C(6a)), 80.1 (C(3a)), 59.12 (C(4) or C(5)), 58.90, 35.7 (C(6)), 27.3 (CH_3), 24.8 (CH_3).

(±)-**S5**

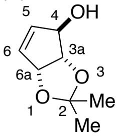

According to procedure by *Trost et. al.*,[4] *n*–butyl lithium (2.5 M in hexane, 9.8 mL, 25 mmol was added slowly to a solution of diethylamine (2.6 mL, 26 mmol) in Et_2O (14 mL) at 0 °C. After 30 min, a solution of **(±)-S4** (1.90 g, 12.4 mmol) in Et_2O (14 mL) was added and the mixture was slowly allowed to reach 23 °C. After 2 h, additional Et_2O (30 mL) was added. The organic layer was washed with an aq. sat. solution of NH_4Cl (3x 15 mL) and dried over $MgSO_4$. Purification by flash chromatography (hexane/EtOAc = 70/30 to 50/50) afforded the product **(±)-S5** as a colorless oil (1.26 g, 66 % yield) as single diastereomer (dr >20:1). The spectroscopic data is in agreement with the literature.[4]

^{1}H NMR ($CDCl_3$, 400 MHz): δ (ppm) 6.03 (dt, *J* = 5.8, 1.4 Hz, 1H, C(6)–H), 5.90 (ddd, *J* = 5.8, 2.2, 1.1 Hz, 1H, C(5)–H), 5.28 (dd, *J* = 5.6, 1.0 Hz, 1H, C(6a)–H), 4.79 (d, *J* = 5.1 Hz, 1H, C(4)–H), 4.52 (dd, *J* = 5.6, 0.8 Hz, 1H, C(3a)–H), 1.91 (d, *J* = 6.0 Hz, 1H, OH), 1.40 (s, 3H, CH_3), 1.35 (s, 3H, CH_3).

^{13}C NMR ($CDCl_3$, 100 MHz) δ (ppm) 135.8 (C(6)), 134.8 (C(5)), 111.9 (C(2)), 86.1 (C(3a)), 84.4 (C(6a)), 81.2 (C(4)), 27.5 (CH_3), 25.9 (CH_3).

(±)-**1**

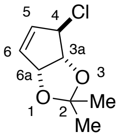

Triphenylphosphane (5.77 g, 22.1 mmol) followed by *N*-chlorosuccinimide (NCS) (3.18 g, 23.6 mmol) was added to a solution of (±)-**S5** (2.30 g, 14.7 mmol) in THF (23 mL) at 0 °C under an argon atmosphere. After 1 h, the mixture was allowed to reach 23 °C and was stirred for additional 4 h. Et_2O (50 mL) was added and the mixture was filtered over Celite. Purification by flash chromatography (hexane/CH_2Cl_2 = 90/10 to 0/100) afforded the product **(±)-1** as a colorless volatile oil (1.85 g, 72% yield) as single diastreomer (dr >20:1).

Determination of the relative stereochemistry:

- Strong NOE of C(2)–CH_3 with C(4)–H (weaker NOE of C(2)–CH_3 with C(3a)–H and C(6a)–H) and NOE of C(2)–CH_3' with C(3a)–H and C(6a)–H indicate *trans* stereochemistry between C(3a)–H and C(4)–H.
- The dihedral angle between C(3a)–H and C(4)–H is close to 90°, therefore a small *J*-coupling is expected. The dihedral angle between C(3a)–H and C(6a)–H is close to zero, therefore a larger *J*-coupling is expected. The observed *J*-coupling between C(3a)–H and C(6a)–H is 5.5 Hz. C(4)–H (app. t, *J* = 1.4 Hz) has a significantly smaller *J*-coupling to C(3a)–H, therefore also supporting the *trans* stereochemistry between C(3a)–H and C(4)–H.

^{1}H NMR ($CDCl_3$, 400 MHz): δ (ppm) 6.05 – 5.99 (m, 1H, C(6)–H), 5.92 (m, 1H, C(5)–H), 5.34 (dd, *J* = 5.5, 1.3 Hz, 1H, C(6a)–H), 4.82 (app. t, *J* = 1.4 Hz, 1H, C(4)–H), 4.80 (d, *J* = 5.5 Hz, 1H, C(3a)–H), 1.40 (s, 3H, CH_3), 1.36 (s, 3H, CH_3).

^{13}C NMR ($CDCl_3$, 100 MHz) δ (ppm) 135.7 (C(6)), 133.6 (C(5)), 112.3 (C(2)), 86.1 (C(3a)), 84.4 (C(6a)), 65.1 (C(4)), 27.6 (CH_3), 26.3 (CH_3).

IR (neat) 2983 (m), 2939 (w), 1374 (w), 1257 (m), 1207 (s), 1158 (m), 1079 (s), 1052 (s), 1004 (w), 869 (s), 771 (s), 718 (w) cm^{-1}.

HRMS (GC-MS EI): m/z calcd for $C_5H_6{}^{37}ClO^+$ $[M - (CH_3)_2CO + H]^+$ 117.0102 found 117.0098 and $C_7H_8{}^{37}ClO^+$ $[M - CH_3]^+$ 159.0207 found 159.0211.

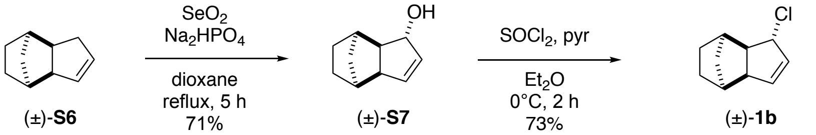

Scheme S2: Overview for the synthesis of (±)-**1b**.

(±)-**S7**

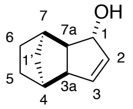

A flask equipped with a reflux condenser was charged with *endo*-5,6-dihydrodicyclopentadiene ((±)-**S6**) (6.00 g, 44.8 mmol), selenium dioxide (9.90 g, 89.6 mmol), Na_2HPO_4 (6.44 g, 44.8 mmol), quartz sand (1.6 g) and dry dioxane (80 mL). The mixture was heated to reflux for 5 h under an argon atmosphere and then filtered over silica and rinsed with EtOAc. The filtrate was concentrated under reduced pressure. Purification by flash chromatography (hexane/EtOAc = 90:10 to 70:30) afforded the product (±)-**S7** as a yellow oil (4.76 g, 71% yield) as single diastreomer (dr >20:1). The spectroscopic data is in agreement with the literature.[5]

^{1}H NMR ($CDCl_3$, 400 MHz): δ (ppm) 5.91 (ddt, *J* = 5.6, 2.0, 0.9 Hz, 1H, C(3)–H), 5.80 (dt, *J* = 5.6, 2.1 Hz, 1H, C(2)–H), 4.67 (s, 1H, C(1)–H), 3.13 (ddp, *J* = 9.2, 4.2, 2.1 Hz, 1H, C(3a)–H), 2.52 – 2.35 (m, 1H, C(7)–H), 2.35 – 2.29 (m, 1H, C(4)–H), 2.25 (ddt, *J* = 8.6, 4.7, 1.7 Hz, 1H, C(7a)–H), 1.52 (dp, *J* = 9.4, 1.9 Hz, 1H, 1x C(1')–H_2), 1.46 (dt, *J* = 9.4, 1.7 Hz, 1H, 1x C(1')–H_2), 1.37 (s, 1H, OH), 1.33 – 1.03 (m, 4H, C(5)–H_2 and C(6)–H_2).

^{13}C NMR ($CDCl_3$, 100 MHz): δ (ppm) 139.4 (C(3)), 133.2 (C(2)), 77.8 (C(1)), 54.5 (C(7a)), 52.1 (C(3a)), 41.6 (C(1')), 39.8 (C(7)), 39.2 (C(4)), 24.9, 23.4 (C(5) and C(6)).

(±)-1b

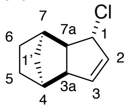

Thionyl chloride (2.0 mL, 28 mmol) was added to solution of (±)-**S7** (2.23 g, 15.0 mmol) in Et_2O (30 mL) 0 °C under an argon atmosphere. After stirring the mixture for 1 h, pyridine (4.1 mL) was added and stirring was continued for 1 h. Then Et_2O (30 mL) was added, the organic layer was washed with H_2O (2x 20 mL) and brine (20 mL), and dried over $MgSO_4$. The mixture was concentrated under reduced pressure at 40 °C. Vacuum distillation (approx. 1 mbar, up to 80 °C) afforded the product **(±)-4** as a colourless oil (1.84 g, 73% yield) with a dr 20:1. The spectroscopic data is in agreement with the literature.[6]

Determination of the relative stereochemistry:

- Strong NOE of C(1)–H with C(5)–H_2/C(6)–H_2.

^{1}H NMR ($CDCl_3$, 400 MHz): δ (ppm) 5.93 (ddt, *J* = 5.6, 2.0, 0.9 Hz, 1H, C(3)–H), 5.80 (dt, *J* = 5.6, 2.1 Hz, 1H, C(2)–H), 4.93 (dq, *J* = 2.7, 1.4 Hz, 1H, C(1)–H), 3.23 (dddt, *J* = 6.8, 5.2, 3.8, 1.9 Hz, 1H, C(3a)–H), 2.74 (ddt, *J* = 9.1, 4.8, 1.7 Hz, 1H, C(7a)–H), 2.42 (t, *J* = 4.6 Hz, 1H, C(7)–H), 2.36 – 2.35 (m, 1H, C(4)–H), 1.53 (dt, *J* = 9.6, 1.9 Hz, 1H, 1x C(1')–H_2), 1.47 (dt, *J* = 9.6, 1.7 Hz, 1H, 1x C(1')–H_2), 1.37 – 1.02 (m, 4H, C(5)–H_2 and C(6)–H_2).

^{13}C NMR ($CDCl_3$, 100 MHz) δ (ppm) 139.7 (C(3)), 132.1 (C(2)), 66.2 (C(1)), 55.2 (C(7a)), 52.2 (C(3a)), 41.4 (C(1')), 40.0 (C(7)), 39.4 (C(4)), 25.1, 22.8 (C(5) and C(6)).

IR (neat) 2952 (s), 2874 (m), 1472 (w), 1453 (w), 1386 (w), 1355 (w), 1126 (w), 1036 (w), 952 (w), 839 (m), 812 (m), 792 (m), 766 (s), 748 (m), 684 (w), 658 (w) cm^{-1}.

HRMS (GC-MS EI): m/z calcd for $C_{10}H_{13}{}^{37}Cl^+$ 168.0706 [M]$^+$ found 168.0698.

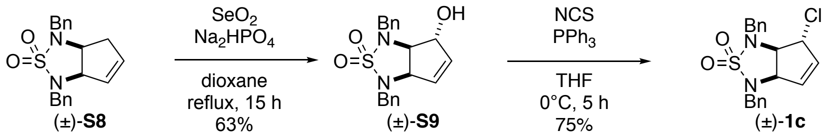

Scheme S3: Overview for the synthesis of (±)-**1c**.

(±)-S9

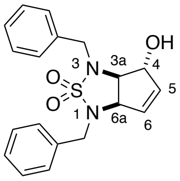

(±)-**S8** was prepared according to a literature procedure by *McDonald et al.*.[7] A flask equipped with a reflux condenser was charged with selenium dioxide (4.62 g, 41.7 mmol), Na_2HPO_4 (3.00 g, 20.9 mmol), quartz sand (700 mg), dry dioxane (40 mL) and (±)-**S8** (7.10 g, 20.9 mmol). The stirred mixture was heated to reflux for 15 h under an argon atmosphere, before being allowed to cool to room temperature and then filtered over Celite. The filtrate was concentrated under reduced pressure and then diluted with EtOAc (100 mL). The organic layer was washed with an aq. sat. solution of NH_4Cl (3x 30 mL), an aq. sat. solution of $NaHCO_3$ (3x 30 mL), and dried over $MgSO_4$. Purification by flash chromatography (hexane/EtOAc = 80/20 to 50/50) afforded the product (±)-**S9** as a highly viscous orange oil that solidified upon standing (4.66 g, 63% yield) as single diastereomer (dr >20:1).

Determination of the relative stereochemistry:

- The dihedral angle between C(3a)–H and C(4)–H is close to 90°, therefore a small *J*-coupling is expected. The dihedral angle between C(3a)–H and C(6a)–H is close to zero, therefore a larger *J*-coupling is expected. The observed *J*-coupling between C(3a)–H and C(6a)–H is 7.9 Hz. C(3a)–H has a significantly smaller *J*-coupling to C(4)–H, therefore also supporting the *trans* stereochemistry between C(3a)–H and C(4)–H.

^{1}H NMR ($CDCl_3$, 400 MHz): δ (ppm) 7.52 – 7.30 (m, 10H, 10x Ar–H), 5.75 (dt, *J* = 5.9, 1.6 Hz, 1H, C(5)–H), 5.32 (dt, *J* = 5.9, 1.7 Hz, 1H, C(6)–H), 4.61 (d, *J* = 13.8 Hz, 1H, 1x PhC*H*$_2$), 4.58 (dd, *J* = 5.0, 3.1 Hz, 1H, C(4)–H), 4.46 (d, *J* = 14.0 Hz, 1H, 1x PhC*H*$_2$), 4.34 (dq, *J* = 7.8, 1.8 Hz, 1H, C(6a)–H), 4.15 (d, *J* = 13.8 Hz, 1H, 1x PhC*H*$_2$), 4.10 (d, *J* = 14.0 Hz, 1H, 1x PhC*H*$_2$), 3.57 (dd, *J* = 7.9, 2.2 Hz, 1H, C(3a)–H), 1.17 (d, *J* = 5.7 Hz, 1H, OH).

^{13}C NMR ($CDCl_3$, 100 MHz) δ (ppm) 136.7 (C(5)), 135.3 , 135.1 (2x C_{quart}), 131.5 (C(6)), 129.6, 129.10, 129.09, 128.9, 128.8, 128.4 (10x C(Ar)–H), 81.1 (C(4)), 67.0 (C(3a)), 65.2 (C(6a)), 50.6, 50.5 (2x $PhCH_2$).

IR ($CHCl_3$ film) 3486 (br. w), 3031 (w), 2917 (s), 1495 (w), 1453 (w), 1296 (m), 1209 (w), 1152 (s), 1095 (m), 1032 (m), 965 (w), 873 (w), 811 (w), 747 (s), 698 (s) cm^{-1}.

HRMS (ESI): m/z calcd for $C_{19}H_{20}O_3N_2NaS^+$ $[M + Na]^+$ 379.10868 found 379.10846.

m.p. 70 – 72 °C.

(±)-1c

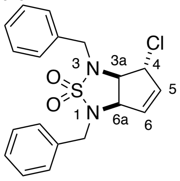

Triphenylphosphane (3.96 g, 15.1 mmol) and then *N*-chlorosuccinimide (2.02 g, 15.1 mmol) were added to solution of (±)-**S9** (4.50 g, 12.6 mmol) in THF (20 mL) at 0 °C under an argon atmosphere. The mixture was stirred for 5 h. Et_2O (50 mL) was added and the mixture was filtered over Celite. Purification by flash chromatography (hexane/CH_2Cl_2 = 50/50 to 30/70) followed by a second purification by flash chromatography (hexane/EtOAc = 90/10 to 80/20) afforded the product (±)-**1c** as a colorless solid (3.55 g, 75% yield) as single diastreomer (dr >20:1).

Determination of the relative stereochemistry:

- Strong NOE of C(4)–H with PhC*H*$_2$ and C(5)–H_2 and a weaker NOE of C(4)–H with C(3a)–H do not allow for an unambiguous assignment, however are in agreement with *trans* stereochemistry between C(3a)–H and C(4)–H.
- The dihedral angle between C(3a)–H and C(4)–H is close to 90°, therefore a small *J*-coupling is expected. The dihedral angle between C(3a)–H and C(6a)–H is close to zero, therefore a larger *J*-coupling is expected. The observed *J*-coupling between C(3a)–H and C(6a)–H is 7.4 Hz. C(4)–H (dt, *J* = 3.1, 1.6 Hz) has a significantly smaller *J*-coupling to C(3a)–H, therefore indicating the *trans* stereochemistry between C(3a)–H and C(4)–H.

^{1}H NMR ($CDCl_3$, 400 MHz): δ (ppm) 7.51 – 7.44 (m, 2H, 2x Ar–H), 7.43 – 7.32 (m, 8H, 8x Ar–H), 6.02 – 5.76 (m, 1H, C(5)–H), 5.53 (ddd, *J* = 5.8, 1.9, 1.2 Hz, 1H, C(6)–H), 4.68 (dt, *J* = 3.1, 1.6 Hz, 1H, C(4)–H), 4.53 (d, *J* = 14.4 Hz, 1H, 1x PhC*H*$_2$), 4.42 (d, *J* = 13.9 Hz, 1H, 1x PhC*H*$_2$), 4.41 (dq, *J* = 7.1, 1.8 Hz, 1H, C(6a)–H) 4.29 (d, *J* = 14.4 Hz, 1H, 1x PhC*H*$_2$), 4.18 (d, *J* = 13.9 Hz, 1H, 1x PhC*H*$_2$), 4.01 (dd, *J* = 7.7, 1.8 Hz, 1H, C(3a)–H).

^{13}C NMR ($CDCl_3$, 100 MHz) δ (ppm) 135.0 (C(5)), 134.7, 134.6 (2x C_{quart}), 131.9 (C(6)), 129.4, 129.1, 129.03, 128.98, 128.63, 128.58 (10x C(Ar)–H), 67.1 (C(3a)), 64.7 (C(6a)), 64.3 (C(1)), 50.8 , 50.5 (2x $PhCH_2$).

IR ($CHCl_3$ film) 3032 (w), 1496 (w), 1454 (w), 1310 (m), 1208 (w), 1160 (s), 1093 (m), 1066 (m), 1029 (w), 964 (w), 814 (m), 745 (s), 699 (s) cm^{-1}.

HRMS (ESI): m/z calcd for $C_{19}H_{19}O_2N_2{}^{37}ClNaS^+$ [M + Na]$^+$ 397.07480 found 397.07472.

m.p. 106 – 107 °C.

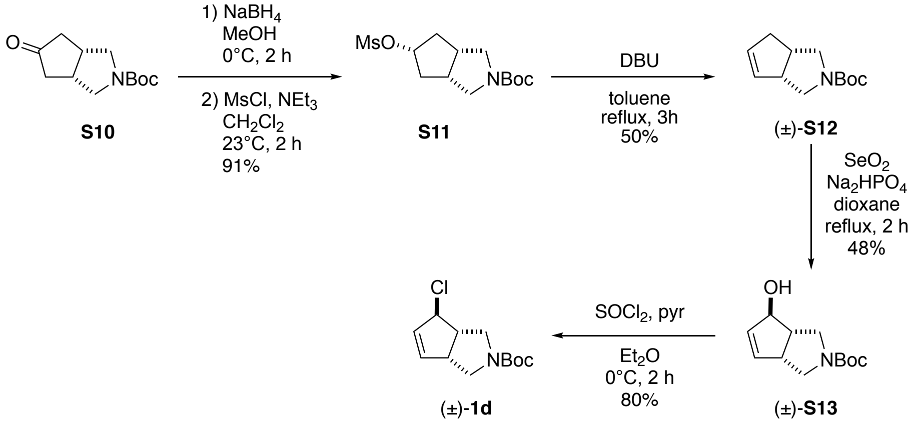

Scheme S4: Overview for the synthesis of (±)-**1d**.

S11

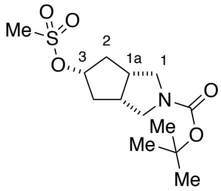

Sodium borohydride (832 mg, 22.0 mmol) was added into a stirred and cooled (0 °C, ice bath) solution of *cis-tert*-butyl 5-oxohexahydrocyclopenta[c]pyrrole-2(1H)-carboxylate (**S10**) (4.50 g, 20.0 mmol) in MeOH (135 mL) under an open atmosphere. After stirring the reaction mixture for 2 h, a sat. aq. solution of NH_4Cl (1 mL) was added dropwise, and the mixture was concentrated under reduced pressure. H_2O (50 mL) was added and the mixture was extracted with EtOAc (3x 100 mL). The combined organic layers were washed with brine (50 mL), dried over $MgSO_4$, and directly used in the next step without any further purification. The crude product was dissolved in CH_2Cl_2 (50 mL) under an argon atmosphere and triethylamine (5.5 mL, 40 mmol) was added. Mesityl chloride (2.3 mL, 30 mmol) was added dropwise at 0 °C. After stirring the reaction mixture for 2 h at 23 °C, H_2O (50 mL) and EtOAc (200 mL) were added. The organic layer was separated and the aq. layer was extracted with EtOAc (2x 100 mL). The combined organic layers were washed with brine (50 mL) and dried over $MgSO_4$. Purification by flash chromatography (hexane/EtOAc = 70/30 to 30/70) afforded the product **S11** as a pale yellow solid (5.54 g, 91% yield) with dr of 14:1.

^{1}H NMR ($CDCl_3$, 400 MHz; major diastereomer): δ (ppm) 5.08 (tt, *J* = 6.8, 5.6 Hz, 1H, C(3)–H), 3.55 (br. s, 2H, 2x C(1)–H_2), 3.36 (s, 2H, 2x C(1)–H_2), 2.98 (s, 3H, SCH_3), 2.68 (br s, 2H), 2.30 (ddd, *J* = 14.7, 8.3, 6.7 Hz 2H, 2x C(2)–H_2), 1.83 (dt, *J* = 14.4, 5.5 Hz, 2H, 2x C(2)–H_2), 1.43 (s, 9H, $C(CH_3)_3$)

^{13}C NMR ($CDCl_3$, 100 MHz; major diastereomer) δ (ppm) 154.5 (C=O), 83.0 (C(3)), 79.4 ($C(CH_3)_3$), 51.8, 51.6 (rotameric 2x C(1)), 41.2, 40.3 (rotameric 2x C(1a)), 38.6 (SCH_3), 38.5 (2x C(2)), 28.6 (3x $C(CH_3)_3$).

IR ($CHCl_3$ film) 2974 (w), 1685 (m), 1480 (w), 1402 (m), 1351 (m), 1248 (w), 1168 (s), 1123 (m), 969 (m), 880 (m), 773 (m) cm^{-1}.

HRMS (ESI): m/z calcd for $C_{13}H_{23}O_5NNaS^+$ [M + Na]$^+$ 328.11891 found 328.11885.

m.p. 52 – 53 °C.

(±)-S12

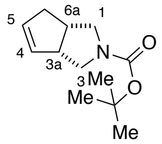

1,8-Diazabicyclo(5.4.0)undec-7-ene (DBU) (8.0 mL, 54 mmol) was added to a stirred solution of **S11 (**5.40 g, 17.7 mmol) in toluene (40 mL) under an argon atmosphere. The mixture was heated to reflux for 3 h, and then cooled and concentrated under reduced pressure. Purification by flash chromatography (hexane/EtOAc = 80/20 to 70/30) afforded the product (±)-**S12** as a colourless oil (1.85 g, 50% yield).

^{1}H NMR ($CDCl_3$, 400 MHz): δ (ppm) 5.68 – 5.64 (m, 1H, C(5)–H), 5.58 (br. s, 1H, C(4)–H), 3.61 (br. s, 1H, 1x C(3)–*H*$_2$), 3.47 (br. s, 1H, 1x C(1)–*H*$_2$), 3.41 – 3.27 (m, 2H, C(3a)–H and 1x C(1)–*H*$_2$), 2.95 (br. s, 1H, 1x C(3)–*H*$_2$), 2.84 (app. t p, *J* = 7.4 Hz, 1H, C(6a)–H), 2.48 (ddq, *J* = 16.6, 7.3, 2.5 Hz, 1H, 1x C(6)–*H*$_2$), 2.13 (br. d, *J* = 16.1 Hz, 1H, 1x C(6)–*H*$_2$), 1.37 (s, 9H, 3x CH_3).

^{13}C NMR ($CDCl_3$, 100 MHz) δ (ppm) 154.5 (C=O), 133.6, 133.2 (rotameric 2x C(4)), 130.5, 130.2 (rotameric 2x C(5)), 79.1 (*C*(CH_3)$_3$), 52.8, 52.5 (rotameric 2x C(3)), 50.6, 50.3 (rotameric 2x C(1)), 50.2, 49.3 (rotameric 2x C(3a)), 40.6, 39.7 (rotameric 2x C(6a)), 38.4 (C(6)), 28.6 (3x CH_3).

IR ($CHCl_3$ film) 2974 (w), 1693 (s), 1478 (w), 1397 (s), 1365 (m), 1338 (w), 1249 (w), 1167 (m), 1109 (m), 881 (w), 772 (w), 684 (w) cm^{-1}.

HRMS (ESI): m/z calcd for $C_{12}H_{19}O_2NNa^+$ [M + Na]$^+$ 232.13080 found 232.13087.

(±)-S13

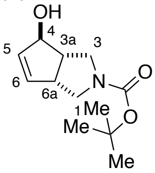

A flask equipped with a reflux condenser was charged with selenium dioxide (1.62 g, 16.2 mmol), Na_2HPO_4 (1.05 g, 8.12 mmol), quartz sand (300 mg), dry dioxane (20 mL) and (±)-**S12** (1.70 g, 8.12 mmol). The mixture was heated to reflux for 2 h under an argon atmosphere, before being allowed to cool to room temperature and then filtered over Celite. The filtrate was concentrated under reduced pressure and then diluted with EtOAc (100 mL). The organic layer was washed with an aq. sat. solution of NH_4Cl (2x 30 mL), an aq. sat. solution of $NaHCO_3$ (2x 30 mL), and dried over $MgSO_4$. Purification by flash chromatography (hexane/EtOAc = 50/50 to 30/70) afforded the product (±)-**S13** as a yellow oil (883 g, 48% yield) as single diastereomer (dr >20:1).

Determination of the relative stereochemistry:

- Strong NOE of C(4)–H with C(3)–H_2 indicates *trans* stereochemistry between C(3a)–H and C(4)–H.

^{1}H NMR ($CDCl_3$, 400 MHz): δ (ppm) 5.89 (br. d, *J* = 4.6 Hz, 1H, C(5)–H), 5.83 (dt, *J* = 5.1, 2.1 Hz, 1H, C(6)–H), 4.59 (s, 1H, C(4)–H), 3.57 (t, *J* = 9.7 Hz, 1H, 1x C(3)–*H*$_2$), 3.52 – 3.47 (br. m, 1H, C(6a)–H), 3.41 (d, *J* = 8.6 Hz, 2H, 2x C(1)–*H*$_2$), 3.18 (br. s, 1H, 1x C(3)–*H*$_2$), 2.72 (dd, *J* = 8.4, 6.5 Hz, 1H, C(3a)–H), 1.87 (br. s, 1H, OH), 1.43 (s, 9H, 3x CH_3).

^{13}C NMR ($CDCl_3$, 100 MHz) δ (ppm) 154.7 (C=O), 138.7 (C(5)), 133.0 (C(6)), 82.6 (C(4)), 79.5 (*C*(CH_3)$_3$), 50.9 (rotameric C(3a)), 49.7 (rotameric C(1) and C(3)), 48.6, 47.8 (rotameric 2x C(6a)), 28.6 (3x CH_3).

IR ($CHCl_3$ film) 3395 (br. s), 2975 (w), 2878 (w), 1670 (s), 1479 (w), 1409 (s), 1365 (m), 1251 (w), 1168 (s), 1117 (m), 1062 (w), 1031 (w), 928 (w), 878 (w), 771 (w) cm^{-1}.

HRMS (ESI): m/z calcd for $C_{12}H_{19}O_3NNa^+$ [M + Na]$^+$ 248.12571 found 248.12576.

(±)-1d

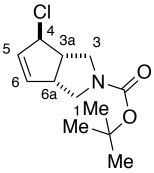

Thionyl chloride (430 µL, 6.1 mmol) was added to solution of (±)-**S13** (855 mg, 3.79 mmol) in Et_2O (7.0 mL) 0 °C under an argon atmosphere. After stirring the mixture for 1 h, pyridine (910 µL) was added and stirring was continued for 1 h. Then Et_2O (50 mL) was added, the organic layer was washed with H_2O (2x 10 mL) and brine (10 mL), and dried over $MgSO_4$. Purification by flash chromatography (hexane/EtOAc = 70/30) afforded the product (±)-**1d** as a colourless solid (743 mg, 80% yield) as single diastereomer (dr >20:1).

Determination of the relative stereochemistry:

- NOE of C(4)–H with C(3a)–H and C(3)–H_2 indicates *trans* stereochemistry between C(3a)–H and C(4)–H.
- *J* coupling analysis was due to rotameric broadening of the peaks difficult.
- High temperature (363 K) NMR in $C_6D_5CD_3$ allowed for *J* coupling analysis. The dihedral angle between C(3a)–H and C(4)–H is close to 90°, therefore a small *J*-coupling is expected. The dihedral angle between C(3a)–H and C(6a)–H is close to zero, therefore a larger *J*-coupling is expected. The observed *J*-coupling between C(3a)–H and C(6a)–H is 6.0 Hz. C(3a)–H has a significantly smaller *J*-coupling to C(4)–H (not resolved), therefore also supporting the *trans* stereochemistry between C(3a)–H and C(4)–H.

^{1}H NMR ($CDCl_3$, 500 MHz): δ (ppm) 5.90 (br. d, *J* = 5.6 Hz, 1H, C(6)–H), 5.86 (dt, *J* = 5.3, 2.3 Hz, 1H, C(5)–H), 4.76 (s, 1H, C(4)–H), 3.64 – 3.56 (m, 2H, 1x C(3)–*H*$_2$ and C(6a)–H), 3.51 – 3.40 (m, 2H, 2x C–*H*$_2$), 3.15 – 3.07 (m, 2H, 1x C(3)–*H*$_2$ and (3a)–H), 1.43 (s, 9H, 3x CH_3).

^{1}H NMR ($C_6D_5CD_3$, 500 MHz; 363 K): 5.46 (dt, *J* = 4.8, 2.2 Hz, 1H, C(5)–H), 5.36 (dd, *J* = 5.6, 2.0 Hz, 1H, C(6)–H), 4.32 (s, 1H, C(4)–H), 3.29 (dd, *J* = 11.6, 9.4 Hz, 1H, 1x C(3)–H_2), 3.26 – 3.20 (m, 1H, 1x C(1)–H_2), 3.09 (dd, *J* = 10.9, 7.8 Hz, 1H, 1x C(1)–H_2), 3.05 – 3.01 (m, 1H, C(6a)–H), 2.96 (dd, *J* = 11.6, 5.8 Hz, 1H, 1x C(3)–H_2), 2.69 (dt, *J* = 9.4, 6.0 Hz, 1H, C(3a)–H), 1.42 (s, 9H, 3x CH_3).

^{13}C NMR ($CDCl_3$, 125 MHz) δ (ppm) 154.4 (C=O), 139.1, 138.9 (2x rotameric C(6)), 132.1, 131.8 (2x rotameric C(5)), 79.7 (*C*(CH_3)$_3$), 68.3 (C(4)), 51.9, 50.9 (2x rotameric C(3a)), 50.1, 49.9 (2x rotameric CH_2), 49.4, 49.0 (2x rotameric CH_2), 48.9, 48.1 (2x rotameric C(6a)), 28.6 (3x CH_3).

IR ($CHCl_3$ film) 2973 (w), 2987 (w), 1691 (s), 1471 (w), 1397 (s), 1240 (w), 1167 (m), 1115 (m), 876 (w), 767 (m), 693 (w) cm^{-1}.

HRMS (ESI): m/z calcd for $C_{12}H_{19}O_2NCl^+$ [M + H]$^+$ 244.10988 found 244.10981.

m.p. 42 – 43 °C.

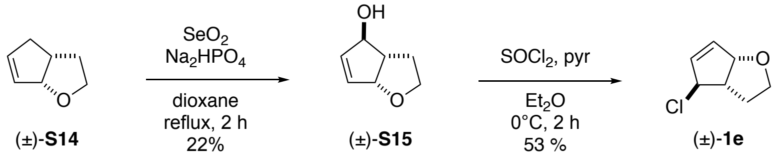

Scheme S5: Overview for the synthesis of (±)-**1e**.

(±)-S15

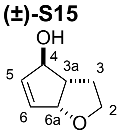

(±)-**S14** was prepared according to a literature procedure by *Rönn et al.*.[8] A flask equipped with a reflux condenser was charged with selenium dioxide (4.33 g, 39.0 mmol), Na_2HPO_4 (2.77 g, 19.5 mmol), quartz sand (1.0 g), dry dioxane (40 mL) and (±)-**S14** (2.15 g, 19.5 mmol). The mixture was stirred for 2 h under reflux under an argon atmosphere and then filtered over Celite. The filtrate was concentrated under reduced pressure and then diluted with EtOAc (100 mL). The organic layer was washed with an aq. sat. solution of NH_4Cl (2x 30 mL), an aq. sat. solution of $NaHCO_3$ (2x 30 mL), and dried over $MgSO_4$. Purification by flash chromatography (hexane/EtOAc = 50/50 to 0/100) afforded the product (±)-**S15** as a yellow oil (540 mg, 22% yield) as single diastereomer (dr >20:1).

Determination of the relative stereochemistry:

- The dihedral angle between C(3a)–H and C(4)–H is close to 90°, therefore a small *J*-coupling is expected. The dihedral angle between C(3a)–H and C(6a)–H is close to zero, therefore a larger *J*-coupling is expected. The observed *J*-coupling between C(3a)–H and C(6a)–H is 6.6 Hz. C(4)–H (s) has a significantly smaller *J*-coupling to C(3a)–H, therefore indicating the *trans* stereochemistry between C(3a)–H and C(4)–H.

^{1}H NMR ($CDCl_3$, 400 MHz): δ (ppm) 5.98 (ddd, *J* = 5.7, 2.3, 0.9 Hz, 1H, C(6)–H), 5.89 (ddd, *J* = 5.6, 2.0, 1.0 Hz, 1H, C(5)–H), 5.27 (dd, *J* = 6.6, 2.1 Hz, 1H, C(6a)–H), 4.61 (s, 1H, C(4)–H), 3.81 (ddd, *J* = 8.7, 7.3, 3.5 Hz, 1H, 1x C(2)–H_2), 3.50 (td, *J* = 9.0, 5.8 Hz, 1H, 1x C(2)–H_2), 2.66 (dddd, *J* = 9.8, 6.5, 3.3, 1.5 Hz, 1H, C(3a)–H), 2.05 (dtd, *J* = 12.5, 9.5, 7.3 Hz, 1H, 1x C(3)–H_2), 1.77 – 1.71 (m, 2H, OH and 1x C(3)–H_2).

^{13}C NMR ($CDCl_3$, 100 MHz) δ (ppm) 136.8 (C(6)), 135.2 (C(5)), 87.6 (C(6a)), 83.5 (C(4)), 66.3 (C(2)), 50.7 (C(3a)), 32.0 (C(3)).

IR (neat) 3362 (br. w), 2944 (w), 2866 (w), 1448 (w), 1357 (m), 1256 (w), 1110 (w), 1057 (s), 1034 (s), 991 (s), 911 (s), 851 (w), 805 (w), 771 (m), 722 (w), 611 (w) cm^{-1}.

HRMS (ESI): m/z calcd for $C_7H_{10}O_2^+$ [M]$^+$ 126.0675 found 126.0671.

(±)-1e

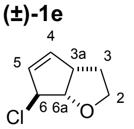

Thionyl chloride (540 µL, 7.4 mmol) was added to solution of (±)-**S15** (500 mg, 3.96 mmol) in Et_2O (8.0 mL) 0 °C under an argon atmosphere. After stirring the mixture for 1 h, pyridine (1.0 mL) was added and stirring was continued for 1 h. Then Et_2O (50 mL) was added, the organic layer was washed with H_2O (10 mL) and brine (10 mL), and dried over $MgSO_4$. Purification by flash chromatography (pentane/Et_2O = 90/10 to 80/20) afforded the product (±)-**1e** as a volatile colourless oil (306 mg, 53% yield) as single diastereomer (dr >20:1) and regioisomer. The minor regioisomer was separated by flash chromatography and obtained as a 4:1 mixture of diastereoisomers (66 mg, 12% yield of mixed diastereoisomers) and not characterized.

Determination of the relative stereochemistry:

- The dihedral angle between C(6)–H and C(6a)–H is close to 90°, therefore a small *J*-coupling is expected. The dihedral angle between C(3a)–H and C(6a)–H is close to zero, therefore a larger *J*-coupling is expected. The observed *J*-coupling between

C(3a)–H and C(6a)–H is 5.5 Hz. C(6)–H (s) has a significantly smaller *J*-coupling to C(6a)–H, therefore indicating the *trans* stereochemistry between C(6a)–H and C(6)–H.

^{1}H NMR ($CDCl_3$, 400 MHz): δ (ppm) 5.88 (dtd, *J* = 4.8, 2.4, 0.8 Hz, 1H, C(5)–H), 5.82 (dd, *J* = 5.6, 2.0 Hz, 1H, C(4)–H), 4.75 (t, *J* = 2.0 Hz, 1H, C(6)–H), 4.67 (d, *J* = 5.5 Hz, 1H, C(6a)–H), 3.86 (ddd, *J* = 8.4, 7.5, 2.4 Hz, 1H, 1x C(2)–H_2), 3.62 – 3.51 (m, 2H, 1x C(2)–H_2 and C(3a)–H), 2.01 (ddt, *J* = 12.4, 10.4, 8.0 Hz, 1H, 1x C(3)–H_2), 1.79 (ddt, *J* = 12.4, 5.4, 2.0 Hz, 1H, 1x C(3)–H_2).
^{13}C NMR ($CDCl_3$, 100 MHz) δ (ppm) 137.6 (C(4)), 132.1 (C(5)), 89.6 (C(3a)), 67.9 (C(6)), 67.5 (C(2)), 49.0 (C(6a)), 30.3 (C(3)).

IR (neat) 2948 (w), 2868 (w), 1610 (w), 1480 (w), 1448 (w), 1362 (w), 1325 (w), 1273 (w), 1201 (w), 1176 (w), 1092 (m), 1070 (s), 1020 (w), 954 (w), 922 (m), 867 (w), 842 (s), 807 (m), 764 (s), 704 (w), 636 (w) cm^{-1}.

HRMS (GC-MS EI) m/z calcd for $C_7H_9{}^{37}ClO^+$ $[M]^+$ 144.0336 found 144.0299.

1.3. Procedures for the Asymmetric Synthesis

General Procedure A
$[Rh(cod)OH]_2$ (4.6 mg, 0.010 mmol, 2.5 mol%) and (*S*)-Segphos (14.6 mg, 0.026 mmol, 6.0 mol%) were added to a flame dried 5 mL round bottom flask, sealed with a rubber septum under an argon atmosphere, dissolved in THF (0.70 mL) and stirred at room temperature. CsOH (50 wt% aq. solution, 70 µL, 0.40 mmol, 1.00 eq) was added and the mixture was heated to 65 °C. After 30 min, a solution (or suspension) of boronic acid (0.80 mmol, 2.0 eq) and allylic chloride (0.40 mmol, 1.0 eq) in THF (0.7 mL) was added via syringe and the flask was rinsed with THF (0.2 mL). The resulting mixture was then stirred at 65 °C or 80 °C for the period of time indicated. The mixture was then cooled to room temperature and diluted with Et_2O (2 mL) or petrolium ether (2 mL) before SiO_2 (50 mg) was added and the solvents were removed in vacuo. Purification by flash chromatography afforded the desired product.

Liquid allylic chlorides were added via syringe (for (±)-**1a** and (±)-**1b:** 0.4 mmol ≙ 62 µL).

Modification to General Procedure A*:
A suspension of the boronic acid was added to the reaction mixture followed by the addition of the neat allyl chloride to the reaction mixture.

General procedure B
The experiment was performed as in general procedure A using (*S*)-BINAP instead of (*S*)-Segphos.

Upscale
Larger-scale experiments were performed in direct analogy to general procedure A.

Racemates
Racemic samples were synthesized with (±)-BINAP instead of (*S*)-Segphos on 0.2 mmol scale.

Comment on relative and absolute stereochemistry.
The absolute stereochemistry of the enantioenriched compounds was assigned by analogy to previous examples of rhodium catalyzed allylic arylation.[9,10] The relative stereochemistry of the allyl chlorides (±)-**1a**, (±)-**1b**, (±)-**1c**, (±)-**1d** and (±)-**1e** and of the coupling products **3aa 3ba**, **3ca**, **3da, 3ea** and **3ea*** was assigned by *J* coupling and 1D NOEs. The absolute and relative stereochemistry of all compounds was assigned by analogy to **3aa**, **3ba**, **3ca**, **3da, 3ea** and **3ea***.

(–)-3aa

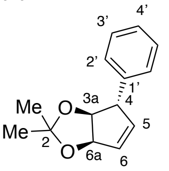

The corresponding compound was prepared following general procedure A using phenylboronic acid. The mixture was stirred at 65 °C for 4 h. Purification by flash chromatography (hexane/Et_2O = 100/0 to 80/20) afforded the product (–)-**3aa** as a colorless oil (78 mg, 91% yield) as a single diastereomer (dr >20:1). SFC analysis showed an enantiomeric excess of 95%.

Determination of the relative stereochemistry:

- The dihedral angle between C(3a)–H and C(4)–H is close to 90°, therefore a small *J*-coupling is expected. The dihedral angle between C(3a)–H and C(6a)–H is close to zero, therefore a larger *J*-coupling is expected. The observed *J*-coupling between C(3a)–H and C(6a)–H is 5.8 Hz. C(4)–H (d, *J* = 2.1 Hz) has a significantly smaller *J*-coupling to C(3a)–H, therefore indicating the *trans* stereochemistry between C(3a)–H and C(4)–H.

^{1}H NMR ($CDCl_3$, 400 MHz): δ (ppm) 7.37 – 7.29 (m, 2H, 2x C(3')–H), 7.26 – 7.21 (m, 1H, C(4')–H), 7.17 – 7.05 (m, 2H, 2x C(2')–H), 6.00 (dt, *J* = 5.7, 1.8 Hz, 1H, C(6)–H), 5.97 – 5.82 (m, 1H, C(5)–H), 5.31 (dq, *J* = 5.9, 1.4 Hz, 1H, C(6a)–H), 4.54 (d, *J* = 5.7 Hz, 1H, C(3a)–H), 4.04 (d, *J* = 2.1 Hz, 1H, C(4)–H), 1.49 (s, 3H, CH_3), 1.34 (s, 3H, CH_3).

^{13}C NMR ($CDCl_3$, 100 MHz) δ (ppm) 141.6 (C(1')), 135.8 (C(5)), 131.9 (C(6)), 128.8 (2x C(3')), 127.7 (2x C(2')), 127.0 (C(4')), 110.5 (C(2)), 86.1 (C(6a)), 85.5 (C(3a)), 58.0 (C(4)), 27.6 (CH_3), 25.8 (CH_3).

IR ($CHCl_3$ film) 3060 (w), 3028 (w), 2985 (m), 2931 (w), 1493 (w), 1454 (w), 1373 (m), 1255 (m), 1212 (s), 1159 (m), 1072 (s), 1049 (s), 885 (m), 755 (m), 701 (m) cm^{-1}.

HRMS (APCI): m/z calcd for $C_{11}H_{11}O^+$ [M – $(CH_3)_2CO$ + H]$^+$ 159.08044 found 159.08041.

SFC Chiralpak® ID; 1500 psi, 30°C; flow: 1.5 mL/min; from 1% to 30% MeOH in 5 min; 97.5:2.5 er (major enantiomer t_R = 1.25 min; minor enantiomer t_R = 1.43 min).

$[\alpha]^{25}_D$ = –244.3 (*c* = 1.0, $CHCl_3$).

(–)-3ab

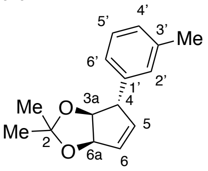

The corresponding compound was prepared following general procedure A using 3-methylphenyl-boronic acid. The mixture was stirred at 65 °C for 4 h. Purification by flash chromatography (petrol ether/Et_2O = 100/0 to 70/30) afforded the product (–)-**3ab** as a pale yellow oil (75 mg, 81% yield) as a single diastereomer (dr >20:1). SFC analysis showed an enantiomeric excess of 95%.

^{1}H NMR ($CDCl_3$, 400 MHz): δ (ppm) 7.20 (t, *J* = 7.5 Hz, 1H, C(5')–H), 7.05 (d, *J* = 7.5 Hz, 1H, C(6')–H), 6.96 – 6.87 (overlapping s and d, 2H, C(2')–H and C(4')–H), 5.99 (dt, *J* = 5.7, 1.8 Hz, 1H, C(6)–H), 5.94 – 5.87 (m, 1H, C(5)–H), 5.47 – 5.17 (m, 1H, C(6a)–H), 4.53 (d, *J* = 5.6 Hz, 1H, C(3a)–H), 4.00 (s, 1H, C(4)–H), 2.33 (s, 3H, C(3')–CH_3), 1.49 (s, 3H, C(CH_3)$_2$), 1.34 (s, 3H, C(CH_3)$_2$).

^{13}C NMR ($CDCl_3$, 100 MHz) δ (ppm) 141.6 (C(1')), 138.5 (C(3')), 135.9 (C(5)), 131.8 (C(6)), 128.7 (C(5')), 128.4 (C(2')), 127.8 (C(4'), 124.7 (C(6')), 110.5 (C(2)), 86.1 (C(6a)), 85.5 (C(3a)), 58.0 (C(4)), 27.6 (C(CH_3)$_2$), 25.8 (C(CH_3)$_2$), 21.5 (C(3')–CH_3).

IR ($CHCl_3$ film) 3055 (w), 2984 (m), 2923 (m), 1373 (m), 1283 (w), 1247 (m), 1212 (m), 1159 (m), 1073 (m), 1049 (s), 867 (m), 781 (m), 704 (m) cm^{-1}.

HRMS (APCI): m/z calcd for $C_{12}H_{13}O^+$ [M – $(CH_3)_2CO$ + H]$^+$ 173.09609 found 173.09610.

SFC Chiralpak® ID; 1500 psi, 30°C; flow: 1.5 mL/min; from 1% to 30% MeOH in 5 min; 97.5:2.5 er (major enantiomer t_R = 1.19 min; minor enantiomer t_R = 1.44 min).

$[\alpha]^{25}_D$ = –229.8 (*c* = 1.0, $CHCl_3$).

(–)-3ac

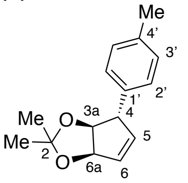

The corresponding compound was prepared following general procedure A using 4-methylphenyl-boronic acid. The mixture was stirred at 65 °C for 4 h. Purification by flash chromatography (petrol ether/Et_2O = 100/0 to 70/30) afforded the product (–)-**3ac** as a colorless oil (77 mg, 84% yield) as a single diastereomer (dr >20:1). SFC analysis showed an enantiomeric excess of 94%.

^{1}H NMR ($CDCl_3$, 400 MHz): δ (ppm) 7.12 (d, *J* = 7.9 Hz, 2H, 2x C(2')–H), 7.02 (d, *J* = 8.0 Hz, 2H, 2x C(3')–H), 5.98 (dt, *J* = 5.7, 1.8 Hz, 1H, C(6)–H), 5.93 – 5.84 (m, 1H, C(5)–H), 5.30 (dq, *J* = 5.7, 1.4 Hz, 1H, C(6a)–H), 4.51 (d, *J* = 5.6 Hz, 1H, C(3a)–H), 4.00 (d, *J* = 2.2 Hz, 1H, C(4)–H), 2.33 (s, 3H, C(4')–CH_3), 1.48 (s, 3H, C(CH_3)$_2$), 1.34 (s, 3H, C(CH_3)$_2$).

^{13}C NMR ($CDCl_3$, 100 MHz) δ (ppm) 138.6 (C(1')), 136.6 (C(5)), 135.9 (C(4')), 129.5 (C(6)), 127.6 (2x C(2')), 114.2 (2x C(3')), 110.4 (C(2)), 86.2 (C(6a)), 85.5 (C(3a)), 57.6 (C(4)), 27.6 (C(CH_3)$_2$), 25.8 (C(CH_3)$_2$), 21.1 (C(4')–CH_3).

IR (neat) 3052 (w), 2985 (m), 2923 (m), 1513 (m), 1456 (w), 1373 (m), 1254 (m), 1212 (m), 1158 (m), 1073 (m), 1049 (s), 887 (m), 866 (m), 776 (w), 731 (w) cm^{-1}.

HRMS (APCI): m/z calcd for $C_{12}H_{13}O^+$ [M – $(CH_3)_2CO$ + H]$^+$ 173.09609 found 173.09603.

SFC Chiralpak® IG; 1500 psi, 30°C; flow: 1.5 mL/min; from 1% to 30% MeOH in 5 min; 97:3 er (minor enantiomer t_R = 1.96 min; major enantiomer t_R = 2.07 min).

$[\alpha]^{25}_D$ = –245.2 (*c* = 1.0, $CHCl_3$).

(–)-3ad

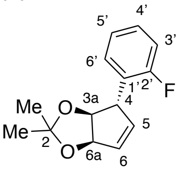

The corresponding compound was prepared following general procedure A using 2-fluorophenyl-boronic acid. The mixture was stirred at 65 °C for 4 h. Purification by flash chromatography (hexane/Et_2O = 100/0 to 80/20) afforded the product (–)-**3ad** as a colourless oil (67 mg, 72% yield) as a single diastereomer (dr >20:1). SFC analysis showed an enantiomeric excess of 90%.

^{1}H NMR ($CDCl_3$, 400 MHz): δ (ppm) 7.22 (dddd, *J* = 8.1, 7.3, 5.2, 1.9 Hz, 1H, C(4')–H), 7.09 – 7.03 (m, 2H, C(3')–H and C(6')–H), 7.00 (app. td, *J* = 7.7, 2.0 Hz, 1H, C(5')–H), 6.01 (dt, *J* = 5.7, 1.9 Hz, 1H, C(6)–H), 5.86 (ddt, *J* = 5.7, 2.5, 0.9 Hz, 1H, C(5)–H), 5.33 – 5.29 (m, 1H, C(6a)–H), 4.59 (dt, *J* = 5.7, 0.7 Hz, 1H, C(3a)–H), 4.27 (dt, *J* = 3.6, 1.9 Hz, 1H, C(4)–H), 1.49 (s, 3H, CH_3), 1.35 (s, 3H, CH_3).

^{13}C NMR ($CDCl_3$, 100 MHz) δ (ppm) 161.2 (d, *J* = 246.5 Hz, C(2')), 134.3 (C(5)), 132.6 (C(6)), 128.9 (d, *J* = 4.4 Hz, C(5')), 128.7 (d, *J* = 8.2 Hz, C(4')) 128.6 (d, *J* = 14.7 Hz, C(3')), 124.3 (d, *J* = 3.6 Hz, C(6')), 115.8 (d, *J* = 21.9 Hz, C(3')), 110.7 (C(2)), 85.6 (C(6a)), 85.1 (C(3a)), 51.5 (d, *J* = 2.1 Hz, C(4)), 27.7 (CH_3), 25.9 (CH_3).

^{19}F NMR ($CDCl_3$, 376 MHz) δ (ppm) -92.21 – -128.33 (m, 1F).

IR ($CHCl_3$ film) 3063 (w), 2987 (w), 2933 (w), 1585 (w), 1490 (m), 1456 (m), 1373 (m), 1212 (s), 1159 (m), 1098 (w), 1073 (m), 1049 (s), 866 (w), 841 (w), 758 (s), 731 (w) cm^{-1}.

HRMS (APCI): m/z calcd for $C_{11}H_{10}OF^+$ 177.07102 [M – $(CH_3)_2CO$ + H]$^+$ found 177.07105.

SFC Chiralpak® IG; 1500 psi, 30°C; flow: 1.5 mL/min; from 1% to 30% MeOH in 5 min; 95:5 er (major enantiomer t_R = 1.46 min; minor enantiomer t_R = 1.76 min).

$[\alpha]^{25}_D$ = –215.5 (*c* = 1.0, $CHCl_3$).

(–)-3ae

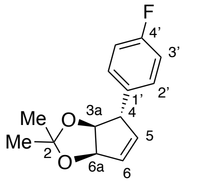

The corresponding compound was prepared following general procedure A using 4-fluorophenyl-boronic acid. The mixture was stirred at 65 °C for 4 h. Purification by flash chromatography (hexane/Et_2O = 100/0 to 70/30) afforded the product (–)-**3ae** as a yellow oil (73 mg, 77% yield) as a single diastereomer (dr >20:1). SFC analysis showed an enantiomeric excess of 94%.

^{1}H NMR ($CDCl_3$, 400 MHz): δ (ppm) 7.16 – 7.05 (m, 2H, 2x C(2')–H), 7.04 – 6.93 (m, 2H, 2x C(3')–H), 6.01 (dt, *J* = 5.6, 1.8 Hz, 1H, C(6)–H), 5.90 (m, 1H, C(5)–H), 5.30 (dq, *J* = 5.7, 1.4 Hz, 1H, C(6a)–H), 4.49 (d, *J* = 5.6 Hz, 1H, C(3a)–H), 4.02 (s, 1H, C(4)–H), 1.49 (s, 3H, CH_3), 1.35 (s, 3H, CH_3).

^{13}C NMR ($CDCl_3$, 100 MHz) δ (ppm) 162.0 (d, *J* = 245.1 Hz, C(4')), 137.4 (d, *J* = 3.4 Hz, 2x C(1')), 135.7 (C(5)), 132.2 (C(6)), 129.2 (d, *J* = 8.1 Hz, 2 xC(2')), 115.7 (d, *J* = 21.4 Hz, 2x (C(3')), 110.7 (C(2)), 86.2 (C(6a)), 85.5 (C(3a)), 57.4 (C(4)), 27.7 (CH_3), 25.9 (CH_3).

^{19}F NMR ($CDCl_3$, 376 MHz) δ (ppm) -116.08 (tt, *J* = 8.6, 5.4 Hz, 1F).

IR ($CHCl_3$ film) 2980 (s), 1604 (w), 1509 (s), 1459 (w), 1375 (m), 1218 (s), 1159 (m), 1074 (m), 1050 (s), 956 (w), 887 (w), 833 (m), 773 (w) cm^{-1}.

HRMS (APCI): m/z calcd for $C_{11}H_{10}OF^+$ [M – $(CH_3)_2CO$ + H]$^+$ 177.07102 found 177.07102.

SFC Chiralpak® ID; 1500 psi, 30°C; flow: 1.5 mL/min; from 1% to 30% MeOH in 5 min; 97:3 er (major enantiomer t_R = 1.09 min; minor enantiomer t_R = 1.24 min).

$[\alpha]^{25}_D$ = –207.7 (*c* = 1.0, $CHCl_3$).

(–)-3af

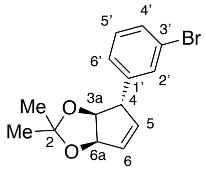

The corresponding compound was prepared following general procedure A using 3-bromophenyl-boronic acid. The mixture was stirred at 65 °C for 4 h. Purification by flash chromatography (hexane/Et_2O = 100/0 to 80/20) afforded the product (–)-**3af** as a colorless oil that solidified upon standing (103 mg, 86% yield) as a single diastereomer (dr >20:1). SFC analysis showed an enantiomeric excess of 95%.

^{1}H NMR ($CDCl_3$, 400 MHz): δ (ppm) 7.37 (ddd, *J* = 7.9, 2.0, 1.1 Hz, 1H, C(4')–H), 7.25 (app. t, *J* = 1.8 Hz, 1H, C(2')–H), 7.18 (app. t, *J* = 7.8 Hz, 1H, C(5')–H), 7.07 (dt, *J*=7.6, 1.4 Hz, 1H, C(6')–H), 6.02 (dt, *J* = 5.7, 1.8 Hz, 1H, C(6)–H), 5.89 (ddt, *J* = 5.7, 2.5, 0.9 Hz, 1H, C(5)–H), 5.29 (ddd, *J* = 5.8, 1.6, 1.0 Hz, 1H, C(6a)–H), 4.50 (d, *J* = 5.7 Hz, 1H, C(3a)–H), 3.99 (d, *J* = 2.2 Hz, 1H, C(4)–H), 1.47 (s, 3H, CH_3), 1.34 (s, 3H, CH_3).

^{13}C NMR ($CDCl_3$, 100 MHz) δ (ppm) 144.1 (C(1')), 135.1 (C(5)), 132.6 (C(6)), 130.7 (C(2'), 130.4 (C(5')), 130.2 (C(4')), 126.5 (C(6')), 123.0 (C(3')), 110.7 (C(2)), 85.8 (C(6a)), 85.3 (C(3a)), 57.7 (C(4)), 27.5 (CH_3), 25.8 (CH_3).

IR ($CHCl_3$ film) 3059 (w), 2986 (w), 2930 (w), 1592 (w), 1567 (w), 1474 (w), 1426 (w), 1373 (m), 1252 (w), 1211 (w), 1158 (m), 1073 (m), 1049 (s), 865 (m), 781 (m), 743 (w), 696 (m) cm^{-1}.

HRMS (APCI): m/z calcd for $C_{11}H_{10}O^{79}Br^+$ [M – $(CH_3)_2CO$ + H]$^+$ 236.99095 found 236.99099 and calcd for $C_{11}H_{10}O^{81}Br^+$ [M – $(CH_3)_2CO$ + H]$^+$ 2368.98891 found 238.98891.

SFC Chiralpak® ID; 1500 psi, 30°C; flow: 1.5 mL/min; from 1% to 30% MeOH in 5 min; 97.5:2.5 er (major enantiomer t_R = 1.39 min; minor enantiomer t_R = 1.98 min).

m.p. 57 – 58 °C.

$[\alpha]^{25}_D$ = –210.4 (*c* = 1.0, $CHCl_3$).

(–)-3ag

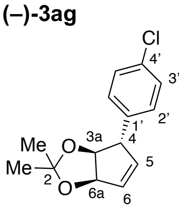

The corresponding compound was prepared following general procedure A using 4-chlorophenyl-boronic acid. The mixture was stirred at 65 °C for 4 h. Purification by flash chromatography (hexane/Et_2O = 100/0 to 70/30) afforded the product (–)-**3ag** as a colorless solid (83 mg, 83% yield) as a single diastereomer (dr >20:1). SFC analysis showed an enantiomeric excess of 93%.

^{1}H NMR ($CDCl_3$, 400 MHz): δ (ppm) 7.38 – 7.19 (m, 2H, 2x C(3')–H), 7.15 – 6.91 (m, 2H, 2x C(2')–H), 6.02 (dt, *J* = 5.7, 1.8 Hz, 1H, C(6)–H), 5.94 – 5.65 (m, 1H, C(5)–H), 5.30 (dq, *J* = 5.6, 1.4 Hz, 1H, C(6a)–H), 4.49 (d, *J* = 5.6 Hz, 1H, C(3a)–H), 4.01 (d, *J* = 2.2 Hz, 1H, C(4)–H), 1.48 (s, 3H, CH_3), 1.34 (s, 3H, CH_3).

^{13}C NMR ($CDCl_3$, 100 MHz) δ (ppm) 140.1 (C(1')), 135.4 (C(5)), 132.8 (C(4')), 132.3 (C(6)), 129.0 (2x C(2')), 128.9 (2x C(3')), 110.7 (C(2)), 86.0 (C(6a)), 85.4 (C(3a)), 57.5 (C(4)), 27.6 (CH_3), 25.8 (CH_3).

IR ($CHCl_3$ film) 2986 (w), 2929 (w), 1492 (m), 1373 (m), 1254 (w), 1212 (m), 1158 (w), 1073 (m), 1050 (s), 1013 (w), 886 (m), 824 (m), 772 (w) cm^{-1}.

HRMS (APCI): m/z calcd for $C_{11}H_{10}O^{37}Cl^+$ [M – $(CH_3)_2CO$ + H]$^+$ 195.03852 found 195.03871.

SFC Chiralpak® IG; 1500 psi, 30°C; flow: 1.5 mL/min; from 1% to 30% MeOH in 5 min; 96.5:3.5 er (minor enantiomer t_R = 2.09 min; major enantiomer t_R = 2.45 min).

m.p. 61 – 62 °C.

$[\alpha]^{25}_D$ = –238.7 (*c* = 1.0, $CHCl_3$).

(–)-3ah

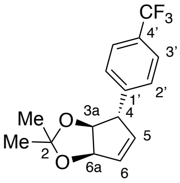

The corresponding compound was prepared following general procedure A using 4-trifluoromethyl-phenylboronic acid. The mixture was stirred at 65 °C for 4 h. Purification by flash chromatography (hexane/Et_2O = 100/0 to 70/30) afforded the product (–)-**3ah** as a colorless oil (78 mg, 68% yield) as a single diastereomer (dr >20:1). SFC analysis showed an enantiomeric excess of 91%.

^{1}H NMR ($CDCl_3$, 400 MHz): δ (ppm) 7.56 (d, *J* = 8.0 Hz, 2H, 2x C(3')–H), 7.24 (d, *J* = 8.3 Hz, 2H, 2x C(2')–H), 6.05 (dt, *J* = 5.7, 1.8 Hz, 1H, C(6)–H), 5.92 – 5.90 (m, 1H, C(5)–H), 5.32 – 5.30 (m, 1H, C(6a)–H), 4.51 (d, *J* = 5.7 Hz, 1H, C(3a)–H), 4.09 (s, 1H, C(4)–H), 1.48 (s, 3H, CH_3), 1.34 (s, 3H, CH_3).

^{13}C NMR ($CDCl_3$, 100 MHz) δ (ppm) 145.8 (C(1')), 135.0 (C(5)), 132.7 (C(6)), 129.39 (q, *J* = 32.4 Hz, C(4')), 128.1 (2x C(2')), 125.8 (weak q, *J* = 3.8 Hz, 2x C(3')), 124.3 (weak q, *J* = 271.8 Hz, just two peaks detected, CF_3), 110.8 (C(2)), 85.8 (C(6a)), 85.4 (C(3a)), 57.9 (C(4)), 27.6 (CH_3), 25.8 (CH_3).

^{19}F NMR ($CDCl_3$, 376 MHz) δ (ppm) -62.46 (br. s, CF_3).

IR ($CHCl_3$ film) 2988 (w), 2934 (w), 1619 (w), 1419 (w), 1375 (w), 1326 (s), 1257 (w), 1213 (m), 1164 (m), 1127 (m), 1068 (m), 1051 (m), 953 (w), 887 (w), 837 (w), 773 (w) cm^{-1}.

HRMS (APCI): m/z calcd for $C_{12}H_{10}OF_3^+$ [M – $(CH_3)_2CO$ + H]$^+$ 227.06783 found 227.06787.

SFC Chiralpak® IG; 1500 psi, 30°C; flow: 1.5 mL/min; from 1% to 30% MeOH in 5 min; 95.5:4.5 er (minor enantiomer t_R = 1.20 min; major enantiomer t_R = 1.38 min).

$[\alpha]^{25}_D$ = –176.4 (*c* = 1.0, $CHCl_3$).

(–)-3ai

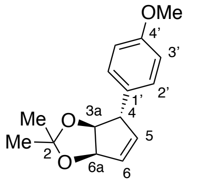

The corresponding compound was prepared following general procedure A using 4-methoxyphenyl-boronic acid. The mixture was stirred at 65 °C for 4 h. Purification by flash chromatography (petrol ether/Et_2O = 100/0 to 60/40) afforded the product (–)-**3ai** as a colorless oil that solidified upon standing (71 mg, 73% yield) as a single diastereomer (dr >20:1). SFC analysis showed an enantiomeric excess of 93%.

^{1}H NMR ($CDCl_3$, 400 MHz): δ (ppm) 7.12 – 6.98 (m, 2H, 2x C(2')–H), 6.88 – 6.82 (m, 2H, 2x C(3')–H), 6.00 – 5.94 (m, 1H, C(6)–H), 5.91 – 5.88 (m, 1H, C(5)–H), 5.35 – 5.17 (m, 1H, C(6a)–H), 4.54 – 4.43 (m, 1H, C(3a)–H), 3.98 (d, *J* = 2.1 Hz, 1H, C(4)–H), 3.79 (s, 3H, OCH_3), 1.48 (s, 3H, C(CH_3)$_2$), 1.33 (s, 3H, C(CH_3)$_2$).

^{13}C NMR ($CDCl_3$, 100 MHz) δ (ppm) 158.7 (C(4')), 136.0 (C(5)), 133.6 (C(1')), 131.6 (C(6)), 128.6 (2x C(2')), 114.2 (2x C(3')), 110.4 (C(2)), 86.2 (C(6a)), 85.4 (C(3a)), 57.1 (C(4)), 55.4 (OCH_3), 27.6 (C(CH_3)$_2$), 25.8 (C(CH_3)$_2$).

IR ($CHCl_3$ film) 3059 (w), 2987 (w), 2932 (w), 2836 (w), 1611 (w), 1512 (s), 1461 (w), 1373 (m), 1251 (s), 1212 (m), 1179 (m), 1073 (m), 1044 (s), 887 (w), 823 (m), 777 (w), 727 (w) cm^{-1}.

HRMS (APCI): m/z calcd for $C_{12}H_{13}O_2^+$ [M – $(CH_3)_2CO$ + H]$^+$ 189.09101 found 189.09092.

SFC Chiralpak® IF; 1500 psi, 30°C; flow: 1.5 mL/min; from 1% to 30% MeOH in 5 min; 96.5:3.5 er (major enantiomer t_R = 2.25 min; minor enantiomer t_R = 2.55 min).

m.p. 44 – 45 °C.

$[\alpha]^{25}_D$ = –228.8 (*c* = 1.0, $CHCl_3$).

(–)-3aj

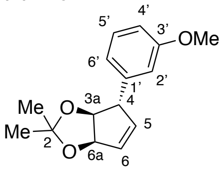

The corresponding compound was prepared following general procedure A using 3-methoxyphenyl-boronic acid. The mixture was stirred at 65 °C for 4 h. Purification by flash chromatography (petrol ether/Et_2O = 90/10 to 70/30) afforded the product (–)-**3aj** as a colorless oil (84 mg, 85% yield) as a single diastereomer (dr >20:1). SFC analysis showed an enantiomeric excess of 95%.

^{1}H NMR ($CDCl_3$, 400 MHz): δ (ppm) 7.23 (app. t, *J* = 7.9 Hz, 1H, C(5')–H), 6.78 (ddd, *J* = 8.3, 2.6, 1.0 Hz, 1H, C(4')–H), 6.72 (dt, *J* = 7.6, 1.4 Hz, 1H, C(6')–H), 6.67 (dd, *J* = 2.6, 1.6 Hz, 1H, C(2')–H), 6.09 – 5.96 (m, 1H, C(6)–H), 5.91 (ddt, *J* = 5.7, 2.4, 0.9 Hz, 1H, C(5)–H), 5.36 – 5.23 (m, 1H, C(5a)–H), 4.53 (d, *J* = 5.7 Hz, 1H, C(3a)–H), 4.00 (d, *J* = 2.1 Hz, 1H, C(4)–H), 3.79 (s, 3H, OCH_3), 1.48 (s, 3H, C(CH_3)$_2$), 1.34 (s, 3H, C(CH_3)$_2$).

^{13}C NMR ($CDCl_3$, 100 MHz) δ (ppm) 160.1 (C(3')), 143.3 (C(1')), 135.6 (C(5)), 132.0 (C(6)), 129.9 (C(5')), 120.0 (C(5')), 113.5 (C(2')), 112.2 (C(4')), 110.5 (C(2)), 86.0 (C(6a)), 85.5 (C(3a)), 58.1 (C(4)), 55.3 (OCH_3), 27.6 (C(CH_3)$_2$), 25.8 (C(CH_3)$_2$).

IR ($CHCl_3$ film) 2987 (w), 2933 (w), 1601 (m), 1489 (m), 1458 (w), 1373 (m), 1317 (m), 1261 (w), 1214 (m), 1157 (m), 1073 (m), 1048 (s), 866 (m), 774 (m), 699 (w) cm^{-1}.

HRMS (APCI): m/z calcd for $C_{12}H_{13}O_2^+$ [M – $(CH_3)_2CO$ + H]$^+$ 189.09101 found 189.09124.

SFC Chiralpak® IG; 1500 psi, 30°C; flow: 1.5 mL/min; from 1% to 30% MeOH in 5 min; 97.5:2.5 er (major enantiomer t_R = 2.19 min; minor enantiomer t_R = 3.67 min).

$[\alpha]^{25}_D$ = –221.9 (*c* = 1.0, $CHCl_3$).

(–)-3ak

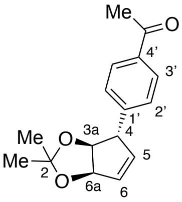

The corresponding compound was prepared following general procedure A using 4-acetylphenyl-boronic acid. The mixture was stirred at 65 °C for 4 h. Purification by flash chromatography (hexane/Et_2O = 90/10 to 40/60) afforded the product (–)-**3ak** as a pale yellow oil (55 mg, 53% yield) as a single diastereomer (dr >20:1). SFC analysis showed an enantiomeric excess of 86%.

^{1}H NMR ($CDCl_3$, 400 MHz): δ (ppm) 7.90 (d, *J* = 8.3 Hz, 2H, 2x C(2')–H), 7.21 (d, *J* = 8.2 Hz, 2H, 2x C(3')–H), 6.04 (ddd, *J* = 5.7, 2.6, 1.1 Hz, 1H, C(6)–H), 6.00 – 5.80 (m, 1H, C(5)–H), 5.31 (ddd, *J* = 5.7, 2.5, 1.5 Hz, 1H, C(6a)–H), 4.52 (dt, *J* = 5.7, 1.1 Hz, 1H, C(3a)–H), 4.09 (d, *J* = 1.8 Hz, 1H, C(4)–H), 2.58 (s, 3H, C(O)CH_3), 1.48 (s, 3H, C(CH_3)$_2$), 1.34 (s, 3H, C(CH_3)$_2$).

^{13}C NMR ($CDCl_3$, 100 MHz) δ (ppm) 197.7 (C=O), 147.2 (C(1')), 136.1 (C(4')), 135.1 (C(5)), 132.6 (C(6)), 129.0 (2x C(2')), 127.9 (2x C(3')), 110.8 (C(2)), 85.8 (C(6a)), 85.4 (C(3a)), 58.1 (C(4)), 27.6 (C(CH_3)$_2$), 26.7 (C(O)CH_3), 25.9 (C(CH_3)$_2$).

IR ($CHCl_3$ film) 2987 (w), 2931 (w), 1684 (s), 1606 (m), 1414 (w), 1363 (m), 1267 (s), 1211 (m), 1159 (w), 1073 (m), 1050 (s), 959 (w), 887 (w), 864 (w), 831 (w) cm^{-1}.

HRMS (APCI): m/z calcd for $C_{13}H_{13}O_2^+$ [M – $(CH_3)_2CO$ + H]$^+$ 201.09101 found 201.09112 and m/z calcd for $C_{16}H_{19}O_3^+$ [M + H]$^+$ 259.13287 found 259.13308.

SFC Chiralpak® ID; 1500 psi, 30°C; flow: 1.5 mL/min; from 1% to 30% MeOH in 5 min; 93:7 er (major enantiomer t_R = 2.85 min; minor enantiomer t_R = 3.35 min).

$[\alpha]^{25}_D$ = –251.1 (*c* = 1.0, $CHCl_3$).

(–)-3al

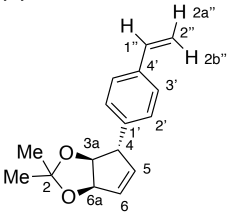

The corresponding compound was prepared following general procedure A using 4-vinylphenyl-boronic acid. The mixture was stirred at 65 °C for 4 h. Purification by flash chromatography (hexane/Et_2O = 100/0 to 80/20) afforded the product (–)-**3al** as a colourless oil that solidified upon standing (41 mg, 42% yield) as a single diastereomer (dr >20:1). SFC analysis showed an enantiomeric excess of 92%.

^{1}H NMR ($CDCl_3$, 400 MHz): δ (ppm) 7.35 (d, *J* = 8.2 Hz, 2H, 2x C(3')–H), 7.08 (d, *J* = 8.2 Hz, 2H, 2x C(2')–H), 6.69 (dd, *J* = 17.6, 10.9 Hz, 1H, C(1'')–H), 6.00 (dt, *J* = 5.7, 1.8 Hz, 1H, C(6)–H), 5.91 (ddd, *J* = 4.8, 2.4, 1.2 Hz, 1H, C(5)–H), 5.72 (dd, *J* = 17.5, 0.9 Hz, 1H, C(2b'')–H), 5.31 (dd, *J* = 5.7, 1.3 Hz, 1H, C(6a)–H), 5.23 (dd, *J* = 10.9, 0.9 Hz, 1H, C(2a'')–H), 4.52 (d, *J* = 5.7 Hz, 1H, C(3a)–H), 4.02 (s, 1H, C(4)–H), 1.48 (s, 3H, CH_3), 1.34 (s, 3H, CH_3).

^{13}C NMR ($CDCl_3$, 100 MHz) δ (ppm) 141.3 (C(1')), 136.5 (C(1''), 136.5 (2x C(4')), 135.7 (C(5)), 132.0 (C(6)), 127.9 (C(2')), 126.7 (C(2')), 113.8 (2x C(2'')), 110.6 (C(2)), 86.1 (C(6a)), 85.5 (C(3a)), 57.8 (C(4)), 27.6 (CH_3), 25.9 (CH_3).

IR ($CHCl_3$ film) 3053 (w), 2986 (w), 2929 (w), 1511 (w), 1373 (m), 1254 (w), 1211 (m), 1159 (w), 1073 (m), 1049 (s), 887 (w), 837 (m), 774 (w) cm^{-1}.

HRMS (APCI): m/z calcd for $C_{13}H_{12}O^+$ 184.0883 [M – $(CH_3)_2CO$ + H]$^+$ found 184.0883.

SFC Chiralpak® IG; 1500 psi, 30°C; flow: 1.5 mL/min; from 1% to 30% MeOH in 5 min; 96:4 er (minor enantiomer t_R = 2.31 min; major enantiomer t_R = 2.64 min).

m.p. 45 – 47 °C.

$[\alpha]^{25}_D$ = –288.3 (*c* = 1.0, $CHCl_3$).

(–)-3am

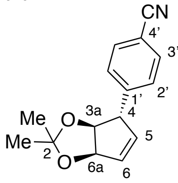

The corresponding compound was prepared following general procedure A using 4-cyanophenyl-boronic acid. The mixture was stirred at 65 °C for 4 h. Purification by flash chromatography (hexane/Et_2O = 90/10 to 60/40) afforded the product (–)-**3am** as a colourless oil (59 mg, 61% yield) as a single diastereomer (d.r. >20:1). SFC analysis showed an enantiomeric excess of 89%.

^{1}H NMR ($CDCl_3$, 400 MHz): δ (ppm) 7.68 – 7.53 (m, 2H, 2x C(3')–H), 7.25 – 7.20 (m, 2H, 2x C(2')–H), 6.06 (dt, *J* = 5.7, 1.8 Hz, 1H, C(6)–H), 5.89 (ddt, *J* = 5.8, 2.5, 0.9 Hz, 1H, C(5)–H), 5.30 (dq, *J* = 5.8, 1.5 Hz, 1H, C(6a)–H), 4.49 (dt, *J* = 5.6, 1.1 Hz, 1H, C(3a)–H), 4.08 (d, *J* = 2.0 Hz, 1H, C(4)–H), 1.48 (s, 3H, CH_3), 1.34 (s, 3H, CH_3).

^{13}C NMR ($CDCl_3$, 100 MHz) δ (ppm) 147.3 (C(4')), 134.6 (C(5)), 133.2 (C(6)), 132.7 (2x C(3')), 128.5 (2x C(2')), 118.9 (CN), 111.7 (C(2)), 111.0 (C(4')), 85.7 (C(6a)), 85.3 (C(3a)), 58.3 (C(4)), 27.6 (CH_3), 25.8 (CH_3).

IR ($CHCl_3$ film) 2984 (m), 2932 (w), 2229 (m), 1608 (w), 1504 (w), 1375 (m), 1256 (m), 1211 (s), 1158 (m), 1074 (s), 1050 (s), 886 (m), 864 (m), 835 (m), 771 (w) cm^{-1}.

HRMS (APCI): m/z calcd for $C_{15}H_{16}O_2N^+$ $[M + H]^+$ 242.11756 found 242.11742 and calcd for $C_{12}H_{10}ON^+$ $[M – (CH_3)_2CO + H]^+$ 184.07569 found 184.07561.

SFC Chiralpak® IC; 1500 psi, 30°C; flow: 1.5 mL/min; from 1% to 30% MeOH in 5 min; 94.5:5.5 er (major enantiomer t_R = 2.25 min; minor enantiomer t_R = 2.33 min).

$[\alpha]^{25}_D$ = –268.9 (*c* = 1.0, $CHCl_3$).

(–)-3an

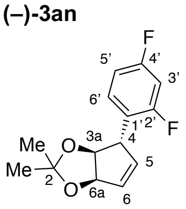

The corresponding compound was prepared following general procedure A using 2,4-difluorophenyl-boronic acid. The mixture was stirred at 65 °C for 4 h. Purification by flash chromatography (hexane/Et_2O = 100/0 to 80/20) afforded the product (–)-**3an** as a colourless oil (59 mg, 58% yield) as a single diastereomer (dr >20:1). SFC analysis showed an enantiomeric excess of 83%.

^{1}H NMR ($CDCl_3$, 400 MHz): δ (ppm) 6.96 (td, *J* = 8.7, 6.3 Hz, 1H, C(6')–H), 6.87 – 6.75 (m, 2H, C(3')–H and C(5')–H), 6.02 (dt, *J* = 5.7, 1.8 Hz, 1H, C(6)–H), 5.84 (ddt, *J* = 5.7, 2.5, 0.9 Hz, 1H, C(5)–H), 5.30 (ddt, *J* = 5.9, 2.2, 1.2 Hz, 1H, C(6a)–H), 4.57 – 4.50 (m, 1H, C(3a)–H), 4.23 (s, 1H, C(4)–H), 1.49 (s, 3H, CH_3), 1.36 (s, 3H, CH_3).

^{13}C NMR ($CDCl_3$, 100 MHz) δ (ppm) 162.8 (dd, *J* = 122.7, 11.9 Hz, C(2')), 160.3 (dd, *J* = 124.2, 11.8 Hz, C(4')), 134.0 (C(5)), 132.9 (C(6)), 129.5 (dd, *J* = 9.6, 6.0 Hz, C(6')), 124.6 (dd, *J* = 14.9, 3.8 Hz, C(1')), 111.3 (dd, *J* = 21.0, 3.8 Hz, C(5')), 110.8 (C(2)), 104.2 (app. t, *J* = 25.6 Hz, C(3')), 85.5 (C(6a)), 85.0 (C(3a)), 51.1 (d, *J* = 1.7 Hz, C(4)), 27.6 (CH_3), 25.9 (CH_3).

^{19}F NMR ($CDCl_3$, 376 MHz) δ (ppm) -111.19 – -112.28 (m, 1F), -112.51 – -114.51 (m, 1F).

IR ($CHCl_3$ film) 2989 (w), 2932 (w), 1530 (s), 1350 (s), 1243 (w), 1211 (m), 1159 (w), 1075 (m), 1050 (m), 868 (w), 739 (m), 689 (w) cm^{-1}.

HRMS (APCI): m/z calcd for $C_{11}H_{19}OF_2^+$ $[M – (CH_3)_2CO + H]^+$ 195.06160 found 195.06192.

SFC Chiralpak® IG; 1500 psi, 30°C; flow: 1.5 mL/min; from 1% to 30% MeOH in 5 min; 91.5:8.5er (major enantiomer t_R = 1.23 min; minor enantiomer t_R = 1.48 min).

$[\alpha]^{25}_D$ = –206.7 (*c* = 1.0, $CHCl_3$).

(–)-3ao

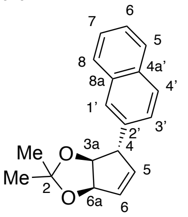

The corresponding compound was prepared following general procedure A using 2-naphtylboronic acid. The mixture was stirred at 65 °C for 4 h. Purification by flash chromatography (hexane/Et_2O = 100/0 to 80/20) afforded the product (–)-**3o** as a colourless solid (89 mg, 83% yield) as a single diastereomer (dr >20:1). SFC analysis showed an enantiomeric excess of 90%.

^{1}H NMR ($CDCl_3$, 400 MHz): δ (ppm) 7.85 – 7.71 (m, 3H, 3x C(Ar)–H), 7.59 – 7.54 (m, 1H, C(Ar)–H), 7.51 – 7.37 (m, 2H, 2x C(Ar)–H), 7.33 – 7.18 (m, 1H, C(Ar)–H), 6.07 (dt, *J* = 5.7, 1.8 Hz, 1H, C(6)–H), 6.03 – 6.00 (m, 1H, C(5)–H), 5.37 (dq, *J* = 5.6, 1.3 Hz, 1H, C(6a)–H), 4.61 (d, *J* = 5.7 Hz, 1H, C(3a)–H), 4.21 (t, *J* = 2.2 Hz, 1H, C(4)–H), 1.52 (s, 3H, CH_3), 1.36 (s, 3H, CH_3)

^{13}C NMR ($CDCl_3$, 100 MHz) δ (ppm) 139.1 ($C(Ar)_{quart}$), 135.7 (C(5)), 133.7 ($C(Ar)_{quart}$), 132.6 ($C(Ar)_{quart}$), 132.2 (C(6)), 128.6 , 127.8, 126.4, 126.2, 125.9, 125.8 (6x (C(Ar)–H), 110.6 (C(2)), 86.0 (C(5a)), 85.6 (C(3a)), 58.2 (C(4), 27.7 (CH_3), 25.9 (CH_3).

IR ($CHCl_3$ film) 2976 (m), 2924 (m), 2867 (w), 1375 (w), 1213 (w), 1053 (s), 1012 (m), 861 (w), 823 (w), 773 (w), 747 (w) cm^{-1}.

HRMS (APCI): m/z calcd for $C_{15}H_{13}O^+$ [M – $(CH_3)_2CO$ + H]$^+$ 209.09609 found 209.09620.

SFC Chiralpak® IC; 1500 psi, 30°C; flow: 1.5 mL/min; from 1% to 30% MeOH in 5 min; 95:5 er (minor enantiomer t_R = 1.95 min; major enantiomer t_R = 2.10 min).

m.p. 123 – 124 °C.

$[\alpha]^{25}_D$ = –294.0 (*c* = 1.0, $CHCl_3$).

(–)-3ap

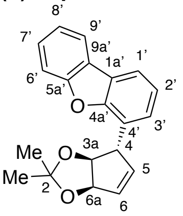

The corresponding compound was prepared following general procedure A using 4-(dibenzofuranyl) boronic acid. The mixture was stirred at 65 °C for 4 h. Purification by flash chromatography (hexane/Et_2O = 95/0 to 70/30) afforded the product (–)-**3ap** as a colourless oil (92 mg, 75% yield) as a single diastereomer (dr >20:1). SFC analysis showed an enantiomeric excess of 85%.

^{1}H NMR ($CDCl_3$, 400 MHz): δ (ppm) 7.95 (ddd, *J* = 7.7, 1.4, 0.7 Hz, 1H, C(9')–H), 7.84 (dd, *J* = 7.7, 1.3 Hz, 1H, C(3')–H), 7.62 (d, *J* = 8.3 Hz, 1H, C(6')–H), 7.47 (ddd, *J* = 8.4, 7.3, 1.4 Hz, 1H, C(7')–H), 7.35 (app. td, *J* = 7.5, 1.0 Hz, 1H, C(8')–H), 7.27 (app. t, *J* = 7.6 Hz, 1H, C(2')–H), 7.10 (dd, *J* = 7.4, 1.2 Hz, 1H, C(1')–H), 6.09 (dt, *J* = 5.8, 1.8 Hz, 1H, C(6)–H), 5.98 (ddt, *J* = 4.8, 2.6, 1.0 Hz, 1H, C(5)–H), 5.57 – 5.36 (m, 1H, C(6a)–H), 4.76 (dd, *J* = 5.6, 0.7 Hz, 1H, C(3a)–H), 4.63 (d, *J* = 2.2 Hz, 1H, C(4)–H), 1.55 (s, 3H, CH_3), 1.38 (s, 3H, CH_3).

^{13}C NMR ($CDCl_3$, 100 MHz) δ (ppm) 156.3 (C(5a')), 154.3 (C(4a')), 134.4 (C(5)), 132.6 (C(6)), 127.3 (C(7')), 125.7 (C(1')), 125.6 (C(4')), 124.5 (C(1a')), 124.5 (C(9a')), 123.0 (C(2')), 122.9 (C(8')), 120.8 (C(9')), 119.4 (C(3')), 112.1(C(6')), 110.7 (C(2)), 85.9 (C(5a)), 85.0 (C(3a)), 52.3 (C(4)), 27.8 (CH_3), 26.1 (CH_3).

IR ($CHCl_3$ film) 3059 (w), 2984 (w), 2932 (w), 1451 (m), 1423 (w), 1374 (w), 1253 (m), 1210 (s), 1187 (w), 1049 (m), 866 (w), 753 (s) cm^{-1}.

HRMS (APCI): m/z calcd for $C_{17}H_{13}O_2^+$ [M – $(CH_3)_2CO$ + H]$^+$ 249.09101 found 249.09062.

SFC Chiralpak® IF; 1500 psi, 30°C; flow: 1.5 mL/min; from 1% to 30% MeOH in 5 min; 92.5:7.5 er (minor enantiomer t_R = 2.85 min; major enantiomer t_R = 3.31 min).

$[\alpha]^{25}_D$ = –120.9 (*c* = 1.0, $CHCl_3$).

(–)-3aq

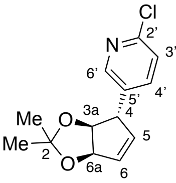

The corresponding compound was prepared following general procedure A using 6-chloro-3-pyridinylboronic acid. The mixture was stirred at 65 °C for 4 h. Purification by flash chromatography (hexane/Et_2O = 90/10 to 50/50 afforded the product (–)-**3aq** as a colorless oil (50 mg, 50 % yield) as a single diastereomer (dr >20:1). SFC analysis showed an enantiomeric excess of 90%.

^{1}H NMR ($CDCl_3$, 400 MHz): δ (ppm) 8.21 (d, *J* = 2.5 Hz, 1H, C(6')–H), 7.38 (dd, *J* = 8.2, 2.5 Hz, 1H, C(4')–H), 7.28 (d, *J* = 8.2 Hz, 1H, C(3')–H), 6.07 (dt, *J* = 5.9, 1.7 Hz, 1H, C(6)–H), 5.89 (ddd, *J* = 5.7, 2.5, 1.0 Hz, 1H, C(5)–H), 5.31 (dq, *J* = 5.7, 1.4 Hz, 1H, C(6a)–H), 4.49 (d, *J* = 5.7 Hz, 1H, C(3a)–H), 4.04 (s, 1H, C(4)–H), 1.48 (s, 3H, CH_3), 1.35 (s, 3H, CH_3).

^{13}C NMR ($CDCl_3$, 100 MHz) δ (ppm) 150.3 (C(2')), 149.1 (C(6')), 137.9 (C(4')), 136.1 (C(4')), 134.3 (C(5)), 133.3 (C(5)), 124.4 (C(3')), 111.1 (C(2), 85.6 (C(3a)), 85.3 (C(3a)), 55.0 (C(4)), 27.6 (CH_3), 25.9 (CH_3).

IR ($CHCl_3$ film) 3054 (w), 2990 (w), 2932 (w), 1584 (w), 1564 (w), 1458 (m), 1376 (m), 1246 (w), 1211 (m), 1158 (w), 1106 (m), 1075 (m), 1050 (s), 886 (m), 864 (w), 834 (w), 773 (w), 743 (w), 708 (w) cm^{-1}.

HRMS (APCI): m/z calcd for $C_{13}H_{15}O_2NCl^+$ $[M + H]^+$ 252.07858 found 252.07822.

SFC Chiralpak® IF; 1500 psi, 30°C; flow: 1.5 mL/min; from 1% to 30% MeOH in 5 min; 95:5 er (minor enantiomer t_R = 3.84 min; major enantiomer t_R = 3.99 min).

$[\alpha]^{25}_D$ = –214.5 (*c* = 1.0, $CHCl_3$).

(–)-3ar

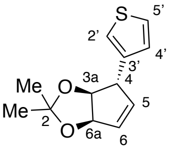

The corresponding compound was prepared following general procedure A using 3-thienylboronic acid. The mixture was stirred at 65 °C for 4 h. Purification by flash chromatography (hexane/Et_2O = 100/0 to 80/20 afforded the product (–)-**3ar** as a colorless oil (64 mg, 72% yield) as a single diastereomer (dr >20:1). SFC analysis showed an enantiomeric excess of 98%.

^{1}H NMR ($CDCl_3$, 400 MHz): δ (ppm) 7.29 (dd, *J* = 5.0, 3.0 Hz, 1H, C(5')–H), 6.95 – 6.93 (m, 1H, C(2')–H), 6.91 (dd, *J* = 5.0, 1.3 Hz, 1H, C(4')–H) 5.96 (app. s, 2H, C(5)–H and C(6)–H), 5.28 (d, *J* = 5.7 Hz, 1H, C(6a)–H), 4.54 (d, *J* = 5.7 Hz, 1H, C(3a)–H), 4.11 (s, 1H, C(4)–H), 1.47 (s, 3H, CH_3), 1.34 (s, 3H, CH_3).

^{13}C NMR ($CDCl_3$, 100 MHz) δ (ppm) 142.4 (C(3')), 135.4 (C(5)), 131.7 (C(6)), 127.3 (C(4')), 126.4 (C(5')), 120.8 (C(2')), 110.6 (C(2)), 85.3 (C(6a)), 85.3 (C(3a)), 52.9 (C(4)), 27.6 (CH_3), 25.9 (CH_3).

IR ($CHCl_3$ film) 3101 (w), 3060 (w), 2985 (w), 2930 (w), 1373 (m), 1246 (w), 1211 (m), 1158 (m), 1073 (m), 1049 (s), 871 (m), 851 (m), 777 (m), 660 (w) cm^{-1}.

HRMS (APCI): m/z calcd for $C_9H_9OS^+$ $[M – (CH_3)_2CO + H]^+$ 165.03686 found 165.03687.

SFC Chiralpak® ID; 1500 psi, 30°C; flow: 1.5 mL/min; from 1% to 30% MeOH in 5 min; 99:1 er (major enantiomer t_R = 1.45 min; minor enantiomer t_R = 1.60 min).

$[\alpha]^{25}_D$ = –230.7 (*c* = 1.0, $CHCl_3$).

(–)-3as

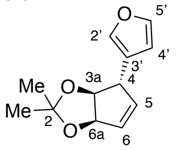

The corresponding compound was prepared following general procedure A using 3-furanylboronic acid. The mixture was stirred at 65 °C for 4 h. Purification by flash chromatography (hexane/Et_2O = 100/0 to 90/10 afforded the product (–)-**3as** as a colorless oil (35 mg, 43% yield) as a single diastereomer (dr >20:1). SFC analysis showed an enantiomeric excess of 96%.

^{1}H NMR ($CDCl_3$, 400 MHz): δ (ppm) 7.38 (app. t, *J* = 1.7 Hz, 1H, C(5')–H), 7.18 (dt, *J* = 1.7, 0.9 Hz, 1H, C(2')–H), 6.24 (dd, *J* = 1.9, 0.9 Hz, 1H, C(4')–H), 6.10 – 5.70 (m, 2H, C(5)–H and C(6)–H), 5.25 (dq, *J* = 5.8, 1.3 Hz, 1H, C(6a)–H), 4.53 (dq, *J* = 5.7, 0.8 Hz, 1H, C(3a)–H), 3.89 (s, 1H, C(4)–H), 1.46 (s, 3H, CH_3), 1.35 (s, 3H, CH_3).

^{13}C NMR ($CDCl_3$, 100 MHz) δ (ppm) 143.6 (C(5')), 139.0 (C(2')), 135.0 (C(5)), 131.7 (C(6)), 125.7 (C(3')), 110.6 (C(2)), 110.1 (C(4')) , 85.3 (C(6a)), 85.1 (C(3a)), 48.3 (C(4)), 27.6 (CH_3), 25.9 (CH_3).

IR ($CHCl_3$ film) 2987 (w), 2931 (w), 1502 (w), 1374 (m), 1247 (w), 1212 (m), 1158 (m), 1072 (s), 1050 (s), 1027 (m), 873 (s), 777 (m), 728 (w), 665 (w) cm^{-1}.

HRMS (APCI): m/z calcd for $C_9H_9O_2^+$ [M – $(CH_3)_2CO$ + H]$^+$ 149.05971 found 149.05995.

SFC Chiralpak® IG; 1500 psi, 30°C; flow: 1.5 mL/min; from 1% to 30% MeOH in 5 min; 98:2 er (major enantiomer t_R = 1.43 min; minor enantiomer t_R = 1.52 min).

$[\alpha]^{25}_D$ = –170.4 (*c* = 1.0, $CHCl_3$).

(–)-3at

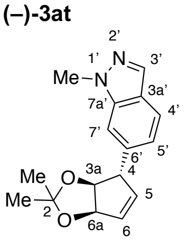

The corresponding compound was prepared following general procedure A using 1-methyl-1*H*-indazole-6-boronic acid. The mixture was stirred at 65 °C for 4 h. Purification by flash chromatography (hexane/Et_2O = 60/40 to 30/70 afforded the product (–)-**3at** as a colorless solid (88 mg, 82% yield) as a single diastereomer (dr >20:1). SFC analysis showed an enantiomeric excess of 92%.

^{1}H NMR ($CDCl_3$, 400 MHz): δ (ppm) 7.93 (d, *J* = 0.9 Hz, 1H, (C(3')–H), 7.66 (dd, *J* = 8.3, 0.8 Hz, 1H, C(4')–H), 7.12 (d, *J* = 0.8 Hz, 1H, C(5')–H), 6.93 (dd, *J* = 8.4, 1.4 Hz, 1H, C(7')–H), 6.07 (dt, *J* = 5.7, 1.8 Hz, 1H, C(6)–H), 5.99 (ddt, *J* = 5.7, 2.5, 0.9 Hz, 1H, C(5)–H), 5.34 (dq, *J* = 5.6, 1.4 Hz, 1H, C(6a)–H), 4.58 (d, *J* = 5.7 Hz, 1H, C(3a)–H), 4.19 (d, *J* = 2.3 Hz, 1H, C(4)–H), 4.04 (s, 3H, NCH_3), 1.51 (s, 3H, C(CH_3)$_2$), 1.35 (s, 3H, C(CH_3)$_2$).

^{13}C NMR ($CDCl_3$, 100 MHz) δ (ppm) 140.4 (C(7a')), 140.3 (C(6')), 135.6 (C(5)), 132.7 (C(3')), 132.4 (C(6)), 123.3 (C(3a)), 121.5 (C(4')), 120.9 (C(7')), 110.7 (C(2)), 107.4 (C(5')), 86.2 (C(6a)), 85.5 (C(3a)), 58.6 (C(4)), 35.6 (NCH_3), 27.6 (C(CH_3)$_2$), 25.8 (C(CH_3)$_2$).

IR ($CHCl_3$ film) 2980 (m), 2934 (m), 1740 (w), 1623 (w), 1475 (w), 1374 (m), 1255 (m), 1213 (m), 1158 (m), 1073 (m), 1048 (s), 962 (m), 882 (m), 840 (m), 772 (w), 629 (w) cm^{-1}.

HRMS (APCI): m/z calcd for $C_{16}H_{18}O_2N_2O^+$ [M + H]$^+$ 271.14410 found 271.14374.

SFC Chiralpak® IG; 1500 psi, 30°C; flow: 1.5 mL/min; from 1% to 30% MeOH in 5 min; 96:4 er (major enantiomer t_R = 3.20 min; minor enantiomer t_R = 3.61 min).

m.p. 100 – 102 °C.

$[\alpha]^{25}_D$ = –263.3 (*c* = 1.0, $CHCl_3$).

(–)-3au

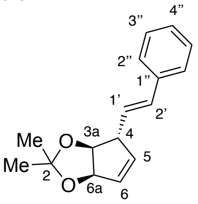

The corresponding compound was prepared following general procedure B using (*E*)-styrylboronic acid. The mixture was stirred at 65 °C for 4 h. Purification by flash chromatography (hexane/Et_2O = 100/0 to 70/30 afforded the product (–)-**3au** as a pale yellow oil (66 mg, 69 % yield; containing minor impurities) as a single diastereomer (dr >20:1, ~33:1). SFC analysis showed an enantiomeric excess of 91%.

^{1}H NMR ($CDCl_3$, 400 MHz): δ (ppm) 7.37 – 7.27 (m, 4H, 2x C(2'')–H and 2x C(3'')–H), 7.25 – 7.19 (m, 1H, C(4'')–H), 6.41 (dd, *J* = 15.9, 1.2 Hz, 1H, C(2')–H), 6.14 (dd, *J* = 15.9, 7.8 Hz, 1H, C(1')–H), 5.92 (dt, *J* = 5.7, 1.7 Hz, 1H, C(6)–H), 5.86 (ddt, *J* = 5.8, 2.4, 0.9 Hz, 1H, C(5)–H), 5.23 (dq, *J* = 6.1, 1.4 Hz, 1H, C(6a)–H), 4.54 (dd, *J* = 5.7, 1.2 Hz, 1H, C(3a)–H), 3.62 (ddt, *J* = 7.8, 2.7, 1.3 Hz, 1H, C(4)–H), 1.46 (s, 3H, CH_3), 1.36 (s, 3H, CH_3).

^{13}C NMR ($CDCl_3$, 100 MHz) δ (ppm) 137.1 (C(1'')), 135.3 (C(5)), 131.9 (C(6)), 131.1 (C(2')), 129.9 (C(1')), 128.7 (C(3'')), 127.6 (C(4'')), 126.3 (C(2'')), 110.4 (C(2)), 85.2 (C(6a)), 84.0 (C(3a)), 54.8 (C(4)), 27.6 (CH_3), 25.9 (CH_3)

IR ($CHCl_3$ film) 3026 (w), 2984 (w), 2931 (w), 1494 (w), 1452 (w), 1373 (m), 1246 (w), 1210 (m), 1158 (m), 1048 (s), 997 (m), 869 (m), 770 (w), 745 (m), 695 (m) cm^{-1}.

HRMS (APCI): m/z calcd for $C_{13}H_{13}O^+$ [M – $(CH_3)_2CO$ + H]$^+$ 185.09609 found 185.09628.

SFC Chiralpak® IB; 1500 psi, 30°C; flow: 1.5 mL/min; from 1% to 30% MeOH in 5 min; 95.5:4.5 er (major enantiomer t_R = 1.58 min; minor enantiomer t_R = 1.76 min).

$[\alpha]^{25}_D$ = –265.3 (*c* = 1.0, $CHCl_3$).

(–)-3av

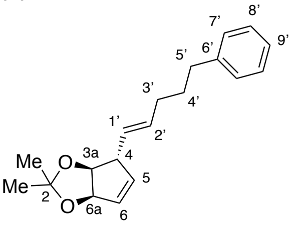

The corresponding compound was prepared following general procedure B using (*E*)-(5-phenylpent-1-en-1-yl)boronic acid. The mixture was stirred at 65 °C for 4 h. Purification by flash chromatography (hexane/Et_2O = 100/0 to 80/20 afforded the product (–)-**3av** as a pale yellow oil (88 mg, 77% yield) as a single diastereomer (dr >20:1; ~25:1). SFC analysis showed an enantiomeric excess of 95%.

^{1}H NMR ($CDCl_3$, 400 MHz): δ (ppm) 7.34 – 7.24 (m, 2H, 2x C(8')–H), 7.18 (m, 3H, 2x C(7')–H and C(9')–H), 5.82 (dt, *J* = 5.7, 1.7 Hz, 1H, C(6)–H), 5.76 (dd, *J* = 5.7, 2.3 Hz, 1H, C(5)–H), 5.49 (dtd, *J* =15.5, 6.6, 1.0 Hz, 1H, C(2')–H), 5.35 (ddt, *J* = 15.4, 7.6, 1.4 Hz, 1H, C(1')–H), 5.16 (dd, *J* = 5.8, 1.1 Hz, 1H, C(6a)–H), 4.42 (d, *J* = 5.7 Hz, 1H, C(3a)–H), 3.40 (d, *J* = 7.7 Hz, 1H, C(4)–H), 2.89 – 2.50 (app. t, *J* = 7.6 Hz, 1H, C(5')–H_2), 2.04 (app. q, *J* = 7.1 Hz, 1H, C(3')–H_2), 1.85 – 1.63 (app. p, *J* = 7.6 Hz, 1H, C(4')–H_2), 1.43 (s, 3H, C(CH_3)$_2$), 1.34 (s, 3H, C(CH_3)$_2$).

^{13}C NMR ($CDCl_3$, 100 MHz) δ (ppm) 142.5 (C(6')), 136.0 (C(5)), 131.7 (C(2')), 131.0 (C(6)), 130.3 (C(1')), 128.6 (C(7')), 128.4 (C(8')), 125.8 (C(9')), 110.2 (C(2)), 85.2 (C(6a)), 84.2 (C(3a)), 54.6 (C(4)), 35.5 (C(5')), 32.1 (C(3')), 31.1 (C(4')), 27.6 (C(CH_3)$_2$), 25.9 (C(CH_3)$_2$).

IR ($CHCl_3$ film) 2987 (w), 2927 (m), 1602 (w), 1495 (w), 1451 (w), 1372 (m), 1212 (m), 1159 (w), 1054 (s), 970 (m), 869 (m), 744 (m), 700 (m) cm^{-1}.

HRMS (APCI): m/z calcd for $C_{16}H_{19}O^+$ [M – $(CH_3)_2CO$ + H]$^+$ 227.14304 found 227.14350.

SFC Chiralpak® ID; 1500 psi, 30°C; flow: 1.5 mL/min; from 1% to 30% MeOH in 5 min; 97.5:2.5 er (major enantiomer t_R = 1.78 min; minor enantiomer t_R = 2.09 min).

$[\alpha]^{25}_D$ = –167.9 (*c* = 1.0, $CHCl_3$).

(–)-3aw

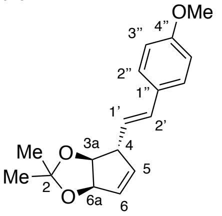

The corresponding compound was prepared following general procedure B using (*E)*-(4-methoxy-styryl)boronic acid. The mixture was stirred at 65 °C for 4 h. Purification by flash chromatography (hexane/Et_2O = 100/0 to 70/30 afforded the product (–)-**3aw** as a colorless solid (73 mg, 67% yield) as a single diastereomer (dr >20:1; ~40:1). SFC analysis showed an enantiomeric excess of 91%.

^{1}H NMR ($CDCl_3$, 400 MHz): δ (ppm) 7.31 – 7.07 (m, 2H, 2x C(2'')–H), 6.91 – 6.73 (m, 2H, 2x C(3'')–H), 6.34 (d, *J* = 15.8 Hz, 1H, C(2')–H), 5.98 (dd, *J* = 15.9, 7.9 Hz, 1H, C(1')–H), 5.89 (dt, *J* = 5.7, 1.7 Hz, 1H, C(6)–H), 5.85 (ddt, *J* = 5.8, 2.4, 0.9 Hz, 1H, C(5)–H), 5.22 (dq, 1H, *J* = 5.7, 1.6 Hz, C(6a)–H), 4.52 (d, *J* = 5.7 Hz, 1H, C(3a)–H), 3.79 (s, 3H, C(4'')OCH_3), 3.59 (ddt, *J* = 7.9, 2.7, 1.3 Hz, 1H, C(4)–H), 1.45 (s, 3H, C(CH_3)$_2$), 1.35 (s, 3H, C(CH_3)$_2$)

^{13}C NMR ($CDCl_3$, 100 MHz) δ (ppm) 159.2 (C(4'')), 135.5 (C(5)), 131.7 (C(6)), 130.4 (C(2')), 129.9 (C(1'')), 127.7 (C(1')), 127.4 (2x C(2'')), 114.1 (2x C(3'')), 110.4 (C(2)), 85.2 (C(6a)), 84.1 (C(3a)), 55.4 (C(4'')O*CH_3*), 54.8 (C(4)), 27.6 (C(*CH_3*)$_2$), 25.9 (C(*CH_3*)$_2$).

IR ($CHCl_3$ film) 2985 (w), 2933 (w), 1607 (m), 1511 (s), 1462 (m), 1373 (m), 1293 (w), 1248 (s), 1209 (m), 1177 (m), 1044 (s), 968 (m), 869 (m), 848 (m), 809 (m), 751 (w) cm^{-1}.

HRMS (APCI): m/z calcd for $C_{14}H_{15}O_2^+$ [M – $(CH_3)_2CO$ + H]$^+$ 215.10658 found 215.10658.

SFC Chiralpak® IA; 1500 psi, 30°C; flow: 1.5 mL/min; from 1% to 30% MeOH in 5 min; 95.5:4.5 er (minor enantiomer t_R = 2.55 min; minor enantiomer t_R = 2.75 min).

m.p. 51 – 52 °C.

[α]$^{25}_D$ = –273.1 (*c* = 1.0, $CHCl_3$).

(–)-3ba

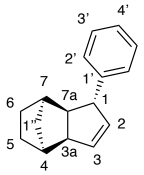

The corresponding compound was prepared following general procedure A using phenylboronic acid. The mixture was stirred at 65 °C for 14 h. Purification by flash chromatography (hexane) afforded the product **(–)-3ba** as a colorless oil (61 mg, 72% yield) as a single diastereomer (dr >20:1). SFC analysis showed an enantiomeric excess of 99%. The spectroscopic data is in agreement with the literature.[11]

Determination of the relative stereochemistry:

- NOE of C(1)–H with overlapping C(1'')–H_2 and C(5)–H_2/C(6)–H_2 is not unambiguous.Weak NOE between C(1)–H and
- C(7a)–H indicates *trans* stereochemistry between C(1)–H and C(7a)–H.

^{1}H NMR ($CDCl_3$, 400 MHz): δ (ppm) 7.31 – 7.23 (m, 2H, 2x C(3')–H), 7.18 – 7.09 (m, 3H, 2x C(2')–H) and C(4')–H), 5.84 – 5.73 (m, 1H, C(3)–H), 5.69 (dt, *J* = 5.6, 2.1 Hz, 1H, C(2)–H), 3.69 (p, *J* = 2.5 Hz, 1H, C(1)–H), 3.17 (dddq, *J* = 10.4, 5.2, 3.5, 1.8 Hz, 1H, C(3a)–H), 2.44 – 2.22 (m, 3H C(4)–H, C(7)–H and C(7a)–H), 1.43 (s, 2H, C(1'')–H_2), 1.41 – 1.19 (m, 4H, C(5)–H_2 and C(6)–H_2).

^{13}C NMR ($CDCl_3$, 100 MHz) δ (ppm) 147.8 (C(1')), 134.3 (C(2)), 134.1 (C(3)), 128.5 (2x C(3')), 127.5 (2x C(2')), 125.9 (C(4')), 54.4 (C(7a)), 52.9 (C(3a)), 51.4 (C(1)), 41.2 (C(7)), 41.1 (C(1'')), 39.3 (C(4)), 25.3, 23.0 (C(5) and C(6)).

IR ($CHCl_3$ film) 2976 (s), 2949 (s), 2873 (m), 1493 (w), 1451 (w), 1385 (w), 1251 (w), 1153, 1074 (w), 955 (w), 763 (m), 745 (m), 698 (m) cm^{-1}.

HRMS (GC-MS EI): m/z calcd for $C_{18}H_{18}^+$ [M]$^+$ 210.1403 found 210.1400.

SFC Chiralpak® IG; 1500 psi, 30°C; flow: 1.5 mL/min; from 1% to 30% MeOH in 5 min; 99.5:0.5 er (major enantiomer t_R = 1.84 min; minor enantiomer t_R = 2.02 min).

[α]$^{25}_D$ = –324.1 (*c* = 1.0, $CHCl_3$).

(–)-3bb

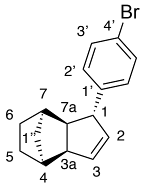

The corresponding compound was prepared following general procedure A using 4-bromophenyl-boronic acid. The mixture was stirred at 65 °C for 14 h. Purification by flash chromatography (hexane) afforded the product (–)-**3bb** as a colorless oil (74 mg, 64% yield) as a single diastereomer (dr >20:1). SFC analysis showed an enantiomeric excess of 99%.

^{1}H NMR ($CDCl_3$, 400 MHz): δ (ppm) 7.43 – 7.33 (m, 2H, 2x C(3')–H), 7.16 – 6.83 (m, 2H, 2x C(2')–H), 5.91 – 5.73 (m, 1H, C(3)–H), 5.66 (dt, *J* = 5.6, 2.2 Hz, 1H, C(2)–H), 3.66 (m, 1H, C(1)–H), 3.19 – 3.13 (m, 1H, C(3a)–H), 2.38 – 2.26 (m, 3H, C(4)–H, C(7)–H and C(7a)–H), 1.45 (s, 2H, C(1'')–H_2), 1.43 – 1.22 (m, 4H, C(5)–H_2 and C(6)–H_2).

^{13}C NMR ($CDCl_3$, 100 MHz) δ (ppm) 146.8 (C(1')), 134.9 (C(3)), 133.5 (C(2)), 131.5 (C(3')), 129.2 (C(2')), 119.6 (C(4')), 54.3 (C(7a)), 52.8 (C(3a)), 50.9 (C(1)), 41.2 (C(7)), 41.1 (C(1'')), 39.2 (C(4)), 25.3, 22.9 (C(5) and C(6)).

IR ($CHCl_3$ film) 3041 (w), 2948 (s), 2872 (m), 1484 (m), 1073 (w), 1010 (w), 863 (w), 845 (w), 822 (w), 800 (w), 758 (w), 654 (w) cm^{-1}.

HRMS (GC-MS EI): m/z calcd for $C_{13}H_{17}{}^{79}Br^+$ $[M]^+$ 288.0508 found 288.0512 and $C_{13}H_{17}{}^{81}Br^+$ $[M]^+$ 290.0493 found 290.0490.

SFC Chiralpak® IG; 1500 psi, 30°C; flow: 1.5 mL/min; from 1% to 30% MeOH in 5 min; 99.5:0.5 er (minor enantiomer t_R = 2.84 min; major enantiomer t_R = 3.64 min).

$[\alpha]^{25}_D$ = –287.0 (*c* = 1.0, $CHCl_3$).

(–)-3bc

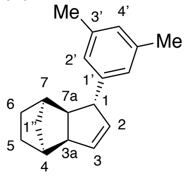

The corresponding compound was prepared following general procedure A* using 3,5-dimethyl-phenylboronic acid. The mixture was stirred at 65 °C for 14 h. Purification by flash chromatography (hexane) afforded the product (–)-**5c** as a colorless oil (59 mg, 62% yield) as a single diastereomer (dr >20:1). SFC analysis showed an enantiomeric excess of >99%.

^{1}H NMR ($CDCl_3$, 400 MHz): δ (ppm) 6.86 (s, 1H, C(4')–H), 6.79 (s, 2H, 2x C(2')–H), 5.79 (dtd, *J* = 5.6, 2.0, 0.8 Hz, 1H, C(3)–H), 5.73 (dt, *J* = 5.6, 2.1 Hz, 1H, C(2)–H), 3.68 (p, *J* = 2.5 Hz, 1H, C(1)–H), 3.22 (ddq, *J* = 10.1, 5.1, 1.7 Hz, 1H, C(3a)–H), 2.44 – 2.34 (m, 3H, C(4)–H, C(7)–H and C(7a)–H), 2.33 (app. d, *J* = 0.7 Hz, 6H, 2x CH_3), 1.48 (s, 2H, C(1'')–H_2), 1.47 – 1.20 (m, 4H, C(5)–H_2 and C(6)–H_2).

^{13}C NMR ($CDCl_3$, 100 MHz) δ (ppm) 147.7 (C(1')), 138.0 (2x C(3')), 134.3 (C(3)), 134.1 (C(2)), 127.6 (C(4')), 125.3 (C(2')), 54.3 (C(7a)), 52.9 (C(3a)), 51.2 (C(1)), 41.2 (C(7)), 41.1 (C(1'')), 39.3 (C(4)), 25. 3, 23.0 (C(5) and C(6)), 21.5 (2x CH_3).

IR ($CHCl_3$ film) 3040 (w), 3014 (w), 2947 (s), 2921 (m), 2871 (m), 1603 (m), 1470 (w), 1451 (w), 851 (w), 764 (m), 700 (w) cm^{-1}.

HRMS (GC-MS EI): m/z calcd for $C_{18}H_{22}^+$ $[M]^+$ 238.1716 found 238.1722.

SFC Chiralpak® IG; 1500 psi, 30°C; flow: 1.5 mL/min; from 1% to 30% MeOH in 5 min; >99.5:0.5 er (major enantiomer t_R = 1.46 min; minor enantiomer t_R = 1.90 min).

$[\alpha]^{25}_D$ = –298.6 (*c* = 1.0, $CHCl_3$).

(–)-3bd

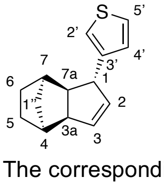

The corresponding compound was prepared following general procedure A using 3-thiopheneboronic acid. The mixture was stirred at 65 °C for 14 h. Purification by flash chromatography (hexane) afforded the product (–)-**3bd** as a colorless solid (45 mg, 52% yield) as a single diastereomer (dr >20:1). SFC analysis showed an enantiomeric excess of >99%.

^{1}H NMR ($CDCl_3$, 400 MHz): δ (ppm) 7.24 (dd, *J* = 4.8, 3.0 Hz, 1H, C(5')–H), 6.95 – 6.85 (m, 2H, C(2')–H and C(4')–H), 5.73 (app. s, 2H, C(3)–H and C(2)–H), 3.82 – 3.80 (m, 1H, C(1)–H), 3.19 – 3.14 (m, 1H, C(3a)–H), 2.41 – 2.37 (m, 1H, C(7a)–H), 2.36 – 2.25 (m, 2H, C(4)–H and C(7)–H), 1.45 (s, 2H, C(1'')–H_2), 1.42 – 1.15 (m, 4H, C(5)–H_2 and C(6)–H_2).

^{13}C NMR ($CDCl_3$, 100 MHz) δ (ppm) 148.5 (C(1')), 134.1 (C(2)), 133.6 (C(3)), 127.5 (C(4')), 125.6 (C(5')), 118.9 (C(2')), 53.2 (C(7a)), 52.6 (C(3a)), 46.3 (C(1)), 41.1 (C(7) and C(1'')), 39.3 (C(4)), 25.3, 22.9 (C(5) and C(6)).

IR ($CHCl_3$ film) 3042 (w), 2947 (s), 2871 (m), 1451 (w), 842 (m), 765 (s), 679 (w), 642 (w) cm^{-1}.

HRMS (GC-MS EI): m/z calcd for $C_{14}H_{16}S^+$ $[M]^+$ 216.0967 found 216.0973.

SFC Chiralpak® IG; 1500 psi, 30°C; flow: 1.5 mL/min; from 1% to 30% MeOH in 5 min; >99.5:0.5 er (minor enantiomer t_R = 2.47 min; major enantiomer t_R = 2.53 min).

$[\alpha]^{25}_D$ = –294.8 (*c* = 1.0, $CHCl_3$).

(–)-3be

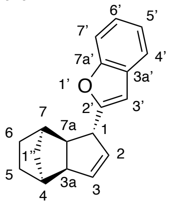

The corresponding compound was prepared following general procedure A using 2-benzofuranyl-boronic acid. The mixture was stirred at 65 °C for 14 h. Purification by flash chromatography (hexane/CH_2Cl_2 = 100/0 to 95/5) afforded the product (–)-**3be** as a colorless solid (61 mg, 61 % yield) as a single diastereomer (dr >20:1). SFC analysis showed an enantiomeric excess of 99%.

^{1}H NMR ($CDCl_3$, 400 MHz): δ (ppm) 7.49 – 7.44 (m, 1H, C(4')–H), 7.42 – 7.40 (m, 1H, C(7')–H), 7.24 – 7.10 (m, 2H, C(5')–H and C(6')–H), 6.32 (s, 1H, C(3')–H) 5.84 (dtd, *J* = 5.0, 2.1, 0.7 Hz, 1H, (C(3)–H), 5.78 (dt, *J* = 5.7, 2.1 Hz, 1H, C(2)–H), 4.03 – 3.73 (m, 1H, C(1)–H), 3.23 – 3.17 (m, 1H, C(3a)–H), 2.65 (m, 1H, C(7a)–H), 2.40 – 2.38 (m, 1H, C(7)–H), 2.36 (tt, *J* = 3.5, 1.2 Hz, 1H, C(4)–H), 1.52 – 1.46 (m, 2H, C(1'')–H_2), 1.45 – 1.25 (m, 4H, C(5)–H_2 and C(6)–H_2).

^{13}C NMR ($CDCl_3$, 100 MHz) δ (ppm) 163.2 (C(2)), 155.0 (C(7a')), 136.1 (C(3)), 130.1 (C(2)), 129.1 (C(3a'), 123.2 (C(6')), 122.5 (C(5')), 120.4 (C(4')), 110.9 (C(7')), 100.7 (C(3')), 52.8 (C(3a)), 50.5 (C(7a)), 44.9 (C(1)), 41.1 (C(7)), 41.1 (C(1'')), 39.3 (C(4)), 25.3, 22.9 (C(5) and C(6)).

IR ($CHCl_3$ film) 3045 (w), 2949 (s), 2872 (m), 1585 (w), 1453 (s), 1253 (m), 1163 (w), 955 (w), 930 (w), 852 (w), 794 (m), 768 (m), 745 (s), 684 (w) cm^{-1}.

HRMS (GC-MS EI): m/z calcd for $C_{18}H_{18}O^+$ $[M]^+$ 250.1352 found 250.1361.

SFC Chiralpak® IG; 1500 psi, 30°C; flow: 1.5 mL/min; from 1% to 30% MeOH in 5 min; 99.5:0.5 er (minor enantiomer t_R = 3.15 min; major enantiomer t_R = 3.64 min).

m.p. 68 – 69 °C.

$[\alpha]^{25}_D$ = –322.4 (*c* = 1.0, $CHCl_3$).

(–)-3ca

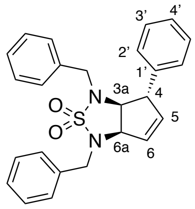

The corresponding compound was prepared following general procedure A using phenylboronic acid. The mixture was stirred at 80 °C for 4 h. Purification by flash chromatography (hexane/EtOAc = 90/10 to 70/30) afforded the product (–)-**3ca** as a colorless solid (142 mg, 85 % yield) as a single diastereomer (d.r. >20:1). SFC analysis showed an enantiomeric excess of 98%.

Determination of the relative stereochemistry:

- The dihedral angle between C(3a)–H and C(4)–H is close to 90°, therefore a small *J*-coupling is expected. The dihedral angle between C(3a)–H and C(6a)–H is close to zero, therefore a larger *J*-coupling is expected. The observed *J*-coupling between C(3a)–H and C(6a)–H is 7.9 Hz. C(4)–H (app. p, *J* = 2.3 Hz) has a significantly smaller *J*-coupling to C(3a)–H, therefore indicating the *trans* stereochemistry between C(3a)–H and C(4)–H.

^{1}H NMR ($CDCl_3$, 400 MHz): δ (ppm) 7.47 – 7.30 (m, 5H, 5x C(Ar)–H), 7.28 – 7.21 (m, 5H, 5x C(Ar)–H), 7.19 – 7.10 (m, 3H, 3x C(Ar)–H), 6.84 – 6.52 (m, 2H, 2x C(Ar)–H), 5.80 (ddd, *J* = 5.8, 2.4, 1.1 Hz, 1H, C(5)–H), 5.52 (dt, *J* = 5.8, 2.1 Hz, 1H, C(6)–H), 4.51 (d, *J* = 14.7 Hz, 1H, 1x PhC*H*$_2$), 4.43 (d, *J* = 14.1 Hz, 1H, 1x PhC*H*$_2$), 4.43 – 4.40 (m, 1H, C(6a)–H), 4.23 (d, *J* = 14.0 Hz, 1H, 1x PhC*H*$_2$), 4.16 (d, *J* = 14.6 Hz, 1H, 1x PhC*H*$_2$), 3.98 (app. p, *J* = 2.3 Hz, 1H, C(4)–H), 3.78 (dd, *J* = 7.9, 2.9 Hz, 1H, C(3a)–H).

^{13}C NMR ($CDCl_3$, 100 MHz) δ (ppm) 141.4 (C(1''), 138.1 (C(5)), 135.3, 135.3 (2x $C(Ar)_{quart}$), 129.1, 129.0, 128.9, 128.8, 128.7, 128.4, 128.2, 128.1, 127.5, 127.2 (15x C(Ar)–H and C(6)), 66.9 (C(6a)), 65.9 (C(3a)), 56.7 (C(4)), 50.7, 50.4 (2x $PhCH_2$).

IR ($CHCl_3$ film) 3030 (w) 2913 (w), 1495 (w), 1453 (w) 1306 (m), 1208 (w), 1159 (s), 1095 (m), 1068 (m), 1030 (w), 961 (w), 912 (w), 866 (w), 801 (w), 748 (w), 700 (s) cm^{-1}.

HRMS (ESI): m/z calcd for $C_{25}H_{24}O_2N_2NaS^+$ $[M + Na]^+$ 439.14507 found 439.14550.

SFC Chiralpak® IG; 1500 psi, 30°C; flow: 1.5 mL/min; from 1% to 30% MeOH in 5 min; 99:1 er (major enantiomer t_R = 5.77 min; minor enantiomer t_R = 6.00 min).

m.p. 96 – 97 °C.

$[\alpha]^{25}_D$ = –124.9 (*c* = 1.0, $CHCl_3$).

(–)-3b

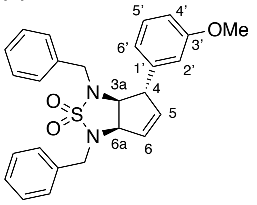

The corresponding compound was prepared following general procedure A using 3-methoxyphenyl-boronic acid. The mixture was stirred at 80 °C for 4 h. Purification by flash chromatography (hexane/EtOAc = 90/10 to 70/30) afforded the product (–)-**3cb** as an off-white solid (150 mg, 84% yield) as a single diastereomer (dr >20:1). SFC analysis showed an enantiomeric excess of 98%.

^{1}H NMR ($CDCl_3$, 400 MHz): δ (ppm) 7.46 – 7.33 (m, 5H, 5x C(Ar)–H), 7.28 (s, 5H, 5x C(Ar)–H), 7.12 (dd, *J* = 8.3, 7.6 Hz, 1H, C(5')–H), 6.72 (ddd, *J* = 8.3, 2.6, 1.0 Hz, 1H, C(4')–H), 6.36 (dt, *J* = 7.6, 1.3 Hz, 1H, C(6')–H), 6.30 (dd, *J* = 2.6, 1.6 Hz, 1H, C(2')–H), 5.81 (ddd, *J* = 5.9, 2.4, 1.1 Hz, 1H, C(5)–H), 5.54 (dt, *J* = 5.8, 2.0 Hz, 1H, C(6)–H), 4.54 (d, *J* = 14.7 Hz, 1H, 1x PhC*H*$_2$), 4.45 (d, *J* = 14.0 Hz, 1H, 1x PhC*H*$_2$), 4.45 – 4.41 (m, 1H, C(6a)–H), 4.25 (d, *J* = 14.1 Hz, 1H, 1x PhC*H*$_2$), 4.19 (d, *J* = 14.7 Hz, 1H, 1x PhC*H*$_2$), 3.97 (app. p, *J* = 2.3 Hz, 1H, C(4)–H), 3.82 (dd, *J* = 7.9, 2.8 Hz, 1H, C(3a)–H), 3.72 (s, 3H, CH_3).

^{13}C NMR ($CDCl_3$, 100 MHz) δ (ppm) 159.9 (C(3')), 142.9 (C(5)), 137.8 (C(1')), 135.3, 135.3 (2x $C(Ar)_{quart}$), 129.8 (C(5')), 129.0, 128.9, 128.7, 128.3, 128.2, 128.1 (10x C(Ar)–H and C(6)), 119.8 (C(6'')), 113.4 (C(2')), 112.3 (C(4')), 66.8 (C(6a)), 65.9 (C(3a)), 56.6 (C(4)), 55.3 (CH_3), 50.7, 50.3 (2x $PhCH_2$).

IR ($CHCl_3$ film) 2914 (w), 1602 (w), 1491 (w), 1455 (w), 1307 (m), 1264 (m), 1156 (s), 104 (w), 1068, 1048 (w), 963 (w), 777 (w), 748 (m), 700 cm^{-1}.

HRMS (ESI): m/z calcd for $C_{26}H_{26}O_3N_2NaS^+$ $[M + Na]^+$ 469.15563 found 469.15560.

SFC Chiralpak® IA; 1500 psi, 30°C; flow: 1.5 mL/min; from 1% to 30% MeOH in 5 min; 99:1 er (minor enantiomer t_R = 4.61 min; major enantiomer t_R = 4.77 min).

m.p. 83 – 84 °C. **$[\alpha]^{25}_D$ =** –115.3 (*c* = 1.0, $CHCl_3$).

(–)-3cc

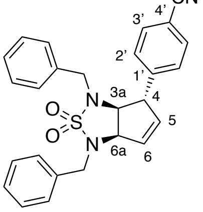

The corresponding compound was prepared following general procedure A using 4-cyanophenylboronic acid. The mixture was stirred at 80 °C for 4 h. Purification by flash chromatography (hexane/EtOAc = 90/10 to 70/30) afforded the product (–)-**3cc** as a off-white solid (93 mg, 52% yield) as a single diastereomer (dr >20:1). SFC analysis showed an enantiomeric excess of 96%.

^{1}H NMR ($CDCl_3$, 400 MHz): δ (ppm) 7.49 – 7.33 (m, 7H, 5x C(Ar)–H and 2x C(3')–H), 7.32 – 7.20 (m, 5H, 5x C(Ar)–H), 6.77 – 6.68 (m, 2H, 2x C(2')–H), 5.76 (ddd, *J* = 5.9, 2.3, 1.2 Hz, 1H, C(5)–H), 5.61 (dt, *J* = 5.9, 2.1 Hz, 1H, C(6)–H), 4.58 (d, *J* = 14.4 Hz, 1H, 1x PhC*H*$_2$), 4.46 (ddd, *J* = 8.1, 3.2, 1.9 Hz, 1H, C(6a)–H), 4.42 (d, *J* = 14.0 Hz, 1H, 1x PhC*H*$_2$), 4.28 (d, *J* = 14.0 Hz, 1H, 1x PhC*H*$_2$), 4.04 (d, *J* = 14.4 Hz, 1H, 1x PhC*H*$_2$), 4.00 (app. p, *J* = 2.4 Hz, 1H, C(4)–H), 3.72 (dd, *J* = 8.1, 3.1 Hz, 1H, C(3a)–H).

^{13}C NMR ($CDCl_3$, 100 MHz) δ (ppm) 146.8 (C(1')), 136.9 (C(5)), 135.1, 135.0 (2x $C(Ar)_{quart}$), 132.5 (C(3')), 129.2, 129.1, 129.0, 128.9, 128.5, 128.4 (10x C(Ar)–H and C(5)), 128.2 (C(2')), 118.6 (CN), 111.1 (C(4')), 66.9 (C(6a)), 65.7 (C(3a)), 57.1 (C(4)), 51.7, 50.2 (2x $PhCH_2$).

IR ($CHCl_3$ film) 3033 (w), 2917 (w), 2227 (w), 1607 (w), 1499 (w), 1454 (w), 1308 (m), 1208 (w), 1160 (s), 1095 (w), 1068 (w), 1028 (w), 962 (w), 869 (w), 835 (w), 748 (m), 700 cm^{-1}.

HRMS (ESI): m/z calcd for $C_{26}H_{23}O_2N_3NaS^+$ $[M + Na]^+$ 464.14032 found 464.14066.

SFC Chiralpak® IB; 1500 psi, 30°C; flow: 1.5 mL/min; from 1% to 30% MeOH in 5 min, then from 30% to 50% MeOH in 0.5 min, then hold 50% MeOH for 1.5 min; 98:2 er (minor enantiomer t_R = 4.46 min; major enantiomer t_R = 4.56 min).

m.p. 121 – 122 °C.

$[\alpha]^{25}_D$ = –162.9 (*c* = 1.0, $CHCl_3$).

(–)-3cd

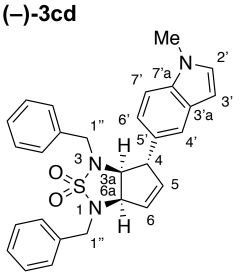

The corresponding compound was prepared following general procedure A using (1-methyl-1*H*-indol-5-yl)boronic acid. The mixture was stirred at 80 °C for 4 h. Purification by flash chromatography (hexane/EtOAc = 90/10 to 70/30) afforded the product **(–)-3cd** as a off-white foam (147 mg, 78 % yield) as a single diastereomer (dr >20:1). SFC analysis showed an enantiomeric excess of 99%.

^{1}H NMR ($CDCl_3$, 400 MHz): δ (ppm) 7.51 – 7.35 (m, 5H, 5x C(Ar)–H), 7.34 – 7.27 (m, 5H, 5x C(Ar)–H), 7.14 (d, *J* = 8.4 Hz, 1H, C(7')–H), 7.03 (d, *J* = 3.1 Hz, 1H, C(2')–H), 6.95 (d, *J* = 1.7 Hz, 1H, C(4')–H), 6.60 (dd, *J* = 8.4, 1.7 Hz, 1H, C(6')–H), 6.35 (dd, *J* = 3.1, 0.9 Hz, 1H, C(3')–H), 5.86 (ddd, *J* = 5.9, 2.4, 1.1 Hz, 1H, C(5)–H), 5.52 (dt, *J* = 5.8, 2.0 Hz, 1H, C(6)–H), 4.59 (d, *J* = 14.8 Hz, 1H, 1x PhC*H*$_2$), 4.48 (dq, *J* = 7.8, 1.9 Hz, 1H, C(6a)–H), 4.46 (d, *J* = 13.8 Hz, 1H, 1x PhC*H*$_2$), 4.26 (d, *J* = 14.1 Hz, 1H, 1x PhC*H*$_2$), 4.18 (d, *J* = 14.8 Hz, 1H, 1x PhC*H*$_2$), 4.08 (p, *J* = 2.3 Hz, 1H, C(4)–H), 3.86 (dd, *J* = 7.9, 2.5 Hz, 1H, C(3a)–H), 3.75 (s, 3H, CH_3).

^{13}C NMR ($CDCl_3$, 100 MHz) δ (ppm) 138.7 (C(5)), 136.0, 135.7, 135.5, 132.1 (4x $C(Ar)_{quart}$), 129.6, 129.1, 128.9, 128.8, 128.7, 128.3, 128.1, 127.4 (10x C(Ar)–H, C(2') and $C(Ar)_{quart}$), 121.1 (C(6')), 119.6 (C(4')), 109.5 (C(7')), 100.9 (C(3')), 67.6 (C(6a)), 66.0 (C(3a)), 56.9 (C(4)), 50.2, 50.3 (2x $PhCH_2$), 33.0 (CH_3).

IR ($CHCl_3$ film) 3031 (w), 2918 (w), 1513 (w), 1494 (w), 1455 (w), 1305 (m), 1246 (w), 1155 (s), 1094 (w), 1070 (w), 1028 (w), 963 (w), 801 (w), 749 (m), 726 (m), 699 cm^{-1}.

HRMS (ESI): m/z calcd for $C_{28}H_{28}O_2N_3S^+$ $[M + H]^+$ 470.18967 found 470.18955.

SFC Chiralpak® IB; 1500 psi, 30°C; flow: 1.5 mL/min; from 1% to 30% MeOH in 5 min, then from 30% to 50% MeOH in 0.5 min, then hold 50% MeOH for 1.5 min; 99.5:0.5 er (major enantiomer t_R = 5.35 min; minor enantiomer t_R = 5.96 min).

m.p. 107 – 108 °C.

$[\alpha]^{25}_D$ = –129.1 (*c* = 1.0, $CHCl_3$).

(–)-3ce

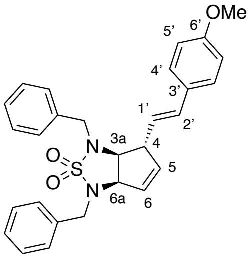

The corresponding compound was prepared following general procedure B using (*E*)-(4-methoxystyryl)boronic acid. The mixture was stirred at 80 °C for 4 h. Purification by flash chromatography (hexane/EtOAc = 90/10 to 70/30) afforded the product **(–)-3ce** as a brown viscous oil (138 mg, 70% yield) as a single diastereomer (dr >20:1). SFC analysis showed an enantiomeric excess of 97%.

^{1}H NMR ($CDCl_3$, 400 MHz): δ (ppm) 7.52 – 7.28 (m, 10H, 10x C(Ar)–H), 7.16 – 7.04 (m, 2H, 2x C(4')–H), 6.96 – 6.75 (m, 2H, 2x C(5')–H), 5.98 (d, *J* = 15.8 Hz, 1H, C(2')–H), 5.74 (ddd, *J* = 5.8, 2.4, 1.1 Hz, 1H, C(5)–H), 5.49 (dd, *J* = 15.8, 8.2 Hz, 1H, C(1')–H), 5.40 (dt, *J* = 5.9, 2.0 Hz, 1H, C(6)–H), 4.54 (d, *J* = 14.4 Hz, 1H, 1x PhC*H*$_2$), 4.44 (d, *J* = 14.1 Hz, 2H, 2x PhC*H*$_2$), 4.34 (dtd, *J* = 8.0, 1.9, 1.1 Hz, 1H, C(6a)–H), 4.22 (d, *J* = 14.2 Hz, 1H, 1x PhC*H*$_2$), 3.80 (s, 3H), 3.67 (dd, *J* = 8.0, 3.0 Hz, 1H, C(3a)–H), 3.53 (dddd, *J* = 8.2, 3.0, 2.1, 0.9 Hz, 1H, C(4)–H).

^{13}C NMR ($CDCl_3$, 100 MHz) δ (ppm) 159.4 (C(6')), 137.5 (C(5)), 135.4, 135.3 (2x $C(Ar)_{quart}$), 130.9 (C(2')), 129.5 (C(3')), 129.4, 129.1, 128.9, 128.8, 128.3 (10x C(Ar)–H), 128.0 (C(6)), 127.5 (C(4')), 126.9 (C(1')), 114.1 (C(5')), 65.7 (C(6a)), 64.6 (C(3a)), 55.4 (CH_3), 53.7 (C(4)), 50.5, 50.5 (2x $PhCH_2$).

IR ($CHCl_3$ film) 3032 (w), 2910 (w), 1607 (m), 1511 (s), 1496 (w), 1456 (w), 1299 (m), 1249 (s), 1208 (w), 1158 (s), 1096 (m), 1068 (m), 1029 (m), 966 (m), 849 (w), 803 (m), 742 (m), 700 (m) cm^{-1}.

HRMS (ESI): m/z calcd for $C_{28}H_{29}O_3N_2S^+$ $[M + H]^+$ 473.18934 found 473.18967.

SFC Chiralpak® IB; 1500 psi, 30°C; flow: 1.5 mL/min; from 1% to 30% MeOH in 5 min, then from 30% to 50% MeOH in 0.5 min; 98.5:1.5 er (major enantiomer t_R = 5.11 min; minor enantiomer t_R = 5.26 min).

$[\alpha]^{25}_D$ = –163.9 (*c* = 1.0, $CHCl_3$).

(–)-3da

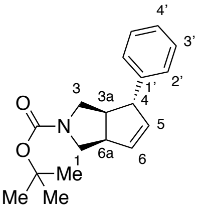

The corresponding compound was prepared following general procedure A using phenylboronic acid. The mixture was stirred at 80 °C for 4 h. Purification by flash chromatography (hexane/Et_2O = 90/10 to 70/30) afforded the product **(–)-3da** as a colorless oil that solidified in the freezer (104 mg, 91% yield) as a single diastereomer (dr >20:1) containing amall amounts (~2-3%) of unreacted starting material. SFC analysis showed an enantiomeric excess of >99%.

Determination of the relative stereochemistry:

- Overlapping peaks and the rotameric broadening of the peaks makes assignment of the relative stereochemistry very difficult.
- High temperature (353 K) NMR in $C_6D_5CD_3$ allowed for *J* coupling analysis. The dihedral angle between C(3a)–H and C(4)–H is close to 90°, therefore a small *J*-coupling is expected. The dihedral angle between C(3a)–H and C(6a)–H is close to zero, therefore a larger *J*-coupling is expected. The observed *J*-coupling between C(3a)–H and C(6a)–H is 7.0 Hz. C(3a)–H has a significantly smaller *J*-coupling to C(4)–H (not resolved), therefore also supporting the *trans* stereochemistry between C(3a)–H and C(4)–H.

^{1}H NMR ($CDCl_3$, 500 MHz): δ (ppm) 7.30 (t, *J* = 7.5 Hz, 2H, 2x C(3')–H), 7.21 (app. t, *J* = 7.3, 1.3 Hz, 1H, C(4')–H), 7.16 (dd, *J* = 8.2, 1.4 Hz, 2H, 2x C(2')–H), 5.82 (br. s, 1H, C(6)–H), 5.80 – 5.76 (br. m, 1H, C(5)–H), 3.76 – 3.64 (br. m, 2H, C(4)–H and 1x C–*H*$_2$), 3.58 – 3.23 (br. m, 4H, C(6a)–H and 3x C–*H*$_2$), 2.77 (br. s, 1H, C(3a)–H), 1.47 (s, 9H, 3x CH_3).

^{1}H NMR ($C_6D_5CD_3$, 500 MHz; 353 K; aromatic peaks due to overlapp not reported): 5.48 (q, *J* = 2.6 Hz, 1H, C(5)–H), 5.44 (dq, *J* = 3.6, 1.5 Hz, 1H, C(6)–H), 3.53 (dd, *J* = 9.1, 6.0 Hz, 1H, 1x C(3)–*H*$_2$), 3.42 (br. s, 1H, C(4)–H), 3.36 – 3.20 (br. m, 3H, 3x C–*H*$_2$), 3.06 – 2.95 (m, 1H, C(6a)–H), 2.43 (dtd, *J* = 8.1, 6.8 Hz, 2.5 Hz, 2.8 Hz, 1H, C(3a)–H), 1.47 (s, 9H, 3x CH_3).

^{13}C NMR ($CDCl_3$, 100 MHz) δ (ppm) 154.6 (C=O), 144.3 (C(1')), 134.4, 134.3 (2x rotameric C(6)), 134.1, 133.9 (2x rotameric C(5)), 128.7 (2x C(3')), 127.3 (2x C(2')), 126.6 (C(4')), 79.3 (*C*(CH_3)$_3$), 57.9 (C(4)), 52.5, 52.1 (2x rotameric CH_2), 51.0, 50.3 (2x rotameric C(3a)), 49.9, 49.7 (2x rotameric CH_2), 48.8 (C(6a)), 28.7 (3x CH_3).

IR ($CHCl_3$ film) 3055 (w), 2876 (w), 1694 (s), 1479 (w), 1453 (w), 1400 (m), 1365 (w), 1169 (m), 1114 (m), 1039 (w), 1027 (w), 980 (w), 772 (w), 717 (w), 700 (w) cm^{-1}.
HRMS (ESI): m/z calcd for $C_{18}H_{23}O_2NNa^+$ $[M + Na]^+$ 308.1620 found 308.16238.

SFC Chiralpak® IE; 1500 psi, 30°C; flow: 1.5 mL/min; from 1% to 30% MeOH in 5 min; >99.5:0.5 er (major enantiomer t_R = 2.99 min; minor enantiomer t_R = 3.12 min).

m.p. 47 – 48 °C.

$[\alpha]^{25}_D$ = –275.7 (*c* = 1.0, $CHCl_3$).

(–)-3db

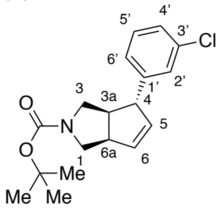

The corresponding compound was prepared following general procedure A using 3-chlorophenylboronic acid. The mixture was stirred at 80 °C for 4 h. Purification by flash chromatography (hexane/Et_2O = 90/10 to 60/40) afforded the product (–)-**3db** as a pale yellow oil (121 mg, 94% yield) as a single diastereomer (dr >20:1) containing amall amounts (~2-3%) of unreacted starting material. SFC analysis showed an enantiomeric excess of 99%.

^{1}H NMR ($CDCl_3$, 500 MHz): δ (ppm) 7.22 (app. t, *J* = 7.7 Hz, 1H, C(5')–H), 7.18 (app. dt, *J* = 8.0, 1.6 Hz, 1H, C(6')–H), 7.13 (app. t, *J* = 1.9 Hz, 1H, C(2')–H), 7.04 (dt, *J* = 7.4, 1.6 Hz, 1H, C(4')–H), 5.85 (br. s, 1H, C(6)–H), 5.75 – 5.73 (br. m, 1H, C(5)–H), 5.74 (br. ddd, *J* = 5.5, 2.6, 1.2 Hz, 1H, C(6)–H), 3.72 – 3.64 (br. m, 2H, C(4)–H and 1x C–H_2), 3.58 – 3.07 (br. m, 4H, C(6a)–H and 3x C–H_2), 2.74 (br. s, 1H, C(3a)–H), 1.46 (s, 9H, 3x CH_3).

^{13}C NMR ($CDCl_3$, 125 MHz) δ (ppm) 154.6 (C=O), 146.4 (C(1')), 135.2, 134.8 (2x rotameric C(6)), 134.6 (C(3')), 133.6, 133.2 (2x rotameric C(5)), 130.0 (C(5')), 127.4 (C(2')), 126.8 (C(6')), 125.5 (C(4')), 79.5 (*C*(CH_3)$_3$), 57.5 (C(4)), 52.4, 52.0 (2x rotameric CH_2), 50.9, 50.1 (2x rotameric C(3a)), 49.8, 49.6 (2x rotameric CH_2), 48.7 (C(6a)), 28.7 (3x CH_3).

IR ($CHCl_3$ film) 2973 (w), 1693 (s), 1585 (w), 1474 (w), 1401 (s), 1247 (w), 1170 (m), 1114 (m), 880 (w), 776 (w), 694 (w) cm^{-1}.

HRMS (ESI): m/z calcd for $C_{18}H_{22}O_2NClNa^+$ $[M + Na]^+$ 342.12313 found 342.12326.

SFC Chiralpak® ID; 1500 psi, 30°C; flow: 1.5 mL/min; from 1% to 30% MeOH in 5 min; 99.5:0.5 er (major enantiomer t_R = 2.44 min; minor enantiomer t_R = 2.75 min).

$[\alpha]^{25}_D$ = –255.1 (*c* = 1.0, $CHCl_3$).

(–)-3dc

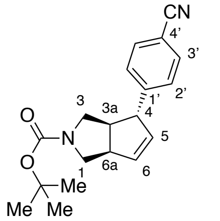

The corresponding compound was prepared following general procedure A using 4-cyanophenylboronic acid. The mixture was stirred at 80 °C for 4 h. Purification by flash chromatography (hexane/Et_2O = 90/10 to 30/70) afforded the product (–)-**3dc** as a colourless oil (93 mg, 75% yield) as a single diastereomer (dr >20:1). SFC analysis showed an enantiomeric excess of 97%.

^{1}H NMR ($CDCl_3$, 500 MHz): δ (ppm) 7.58 (d, *J* = 8.3 Hz, 2H, 2x C(3')–H), 7.26 (d, *J* = 8.3 Hz, 2H, 2x C(2')–H), 5.89 (br. s, 1H, C(6)–H), 5.79 – 5.65 (br. m, 1H, C(5)–H), 3.76 (br. s, 1H, C(4)–H), 3.69 (dd, *J* = 11.3, 8.7 Hz, 1H, 1x C–H_2), 3.58 – 3.23 (br. m, 4H, C(6a)–H and 3x C–H_2), 2.73 (br. s, 1H, C(3a)–H), 1.46 (s, 9H, 3x CH_3).

^{13}C NMR ($CDCl_3$, 125 MHz) δ (ppm) 154.6 (C=O), 149.8 (C(1')), 135.8, 135.5 (2x rotameric C(6)), 133.0 (1x rotameric C(5)), 132.6 (1x rotameric C(5) and 2x C(3')), 128.1 (2x C(2')), 119.0 (CN), 110.6 (C(4')), 79.6 (*C*(CH_3)$_3$), 57.9 (C(4)), 52.3, 52.0, 50.8, 50.1, 49.7, 48.8 (C(3a), rotameric CH_2 and C(6a)), 28.6 (3x CH_3).

IR ($CHCl_3$ film) 2975 (w), 2227 (w), 1691 (s), 1607 (w), 1503 (w), 1478 (w), 1454 (w), 1400 (m), 1365 (m), 1250 (w), 1168 (m), 1116 (m), 884 (w), 830 (w), 771 (w), 702 (w) cm^{-1}.

HRMS (ESI): m/z calcd for $C_{19}H_{22}O_2N_2Na^+$ $[M + Na]^+$ 333.15735 found 333.15709.

SFC Chiralpak® IC; 1500 psi, 30°C; flow: 1.5 mL/min; from 1% to 30% MeOH in 5 min; 98.5:1.5 er (major enantiomer t_R = 4.64 min; minor enantiomer t_R = 4.80 min).

$[\alpha]^{25}_D$ = –343.2 (*c* = 1.0, $CHCl_3$).

(–)-3dd

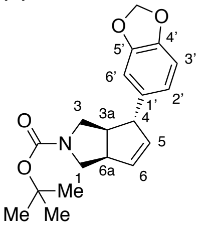

The corresponding compound was prepared following general procedure A using benzo[*d*][1,3] dioxol-5-ylboronic acid. The mixture was stirred at 80 °C for 4 h. Purification by flash chromatography (hexane/Et_2O = 90/10 to 70/30) afforded the product (–)-**3dd** as a colorless oil (98 mg, 74% yield) as a single diastereomer (dr >20:1). SFC analysis showed an enantiomeric excess of 99%.

^{1}H NMR ($CDCl_3$, 500 MHz): δ (ppm) 6.73 (d, *J* = 7.6 Hz, 1H, C(2')–H), 6.63 – 6.61(overlapping m, 2H, C(3')–H and C(6')–H), 5.92 (s, 2H, CH_2O_2), 5.80 (br. s, 1H, C(6)–H), 5.73 (dt, *J* = 5.5, 1.7 Hz, 1H, C(5)–H), 3.77 – 3.56 ((br. m, 2H, C(4)–H and 1x C–H_2)), 3.55 – 3.18 (br. m, 4H, C(6a)–H and 3x C–H_2), 2.70 (br. s, 1H, C(3a)–H), 1.46 (s, 9H, 3x CH_3).

^{13}C NMR ($CDCl_3$, 125 MHz) δ (ppm) 154.6 (C=O), 148.0 (C(5')), 146.3 (C(4')), 138.3 (C(1')), 134.4, 134.1 (C(5) and C(6)), 120.1 (C(2')), 108.3 (C(6')), 107.6 (C(3')), 101.0 (CH_2O_2), 79.4 (*C*(CH_3)$_3$), 57.6 (C(4)), 52.4, 52.0 (2x rotameric CH_2), 51.2, 50.4 (2x rotameric C(3a)), 50.2, 49.8 (2x rotameric CH_2), 49.6, 48.6 (2x rotameric C(6a)), 28.7 (3x CH_3).

IR ($CHCl_3$ film) 2975 (w), 2880 (w), 1693 (s), 1503 (w), 1487 (m), 1441 (w), 1400 (m), 1365 (w), 1246 (m), 1170 (m), 1114 (m), 1039 (m), 937 (w), 881 (w), 802 (w) cm^{-1}.

HRMS (ESI): m/z calcd for $C_{19}H_{23}O_4NNa^+$ $[M + Na]^+$ 352.15193 found 352.15154.

SFC Chiralpak® IC; 1500 psi, 30°C; flow: 1.5 mL/min; from 1% to 30% MeOH in 5 min; 99.5:0.5 er (major enantiomer t_R = 3.46 min; minor enantiomer t_R = 3.70 min).

$[\alpha]^{25}_D$ = –284.2 (*c* = 1.0, $CHCl_3$).

(–)-3de

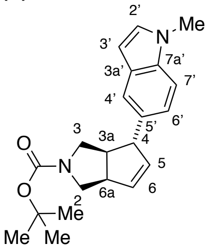

The corresponding compound was prepared following general procedure A using (1-methyl-1*H*-indol-5-yl)boronic acid. The mixture was stirred at 80 °C for 4 h. Purification by flash chromatography (hexane/Et_2O = 90/10 to 30/70) afforded the product (–)-**3de** as a colourless oil that solidified at –20 °C (86 mg, 64% yield) as a single diastereomer (dr >20:1). SFC analysis showed an enantiomeric excess of 99%.

^{1}H NMR ($CDCl_3$, 500 MHz): δ (ppm) 7.41 (d, *J* = 1.7 Hz, 1H, C(4')–H), 7.26 (d, *J* = 8.4 Hz, 1H, C(7')–H), 7.07 – 7.01 (m, 2H, C(2')–H and C(6')–H), 6.43 (dd, *J* = 3.0, 0.9 Hz, 1H, C(3')–H), 5.83 (br. s, 2H, C(6)–H and C(5)–H), 3.82 (br. s, 1H, C(4)–H), 3.78 (s, 3H, NCH_3), 3.70 (dd, *J* = 11.2, 8.7 Hz, 1H), 3.58 – 3.23 (br. m, 4H, C(6a)–H and 3x C–H_2), 2.81 (br. s, 1H, C(3a)–H), 1.48 (s, 9H, 3x CH_3).

^{13}C NMR ($CDCl_3$, 125 MHz) δ (ppm) 154.7 (C=O), 135.8 (C(3a')), 135.2 (1x rotameric C(6) and C(5')), 134.8 (1x rotameric C(6)), 133.7 (1x rotameric C(6)), 133.4 (2x rotameric C(5)), 129.3 (C(6')), 128.8 (C(3')), 121.3 (C(2')), 118.9 (C(4')), 109.4 (C(7')), 100.8 (C(7a')), 79.3 (*C*(CH_3)$_3$), 58.0 (C(4)), 52.6, 52.2 (2x rotameric CH_2), 51.6, 50.9 (2x rotameric C(3a)), 50.4, 50.0 (2x rotameric CH_2), 49.7, 48.7 (2x rotameric C(6a)), 33.0 (NCH_3), 28.7 (3x CH_3).

IR ($CHCl_3$ film) 2969 (w), 1691 (s), 1484 (w), 1401 (s), 1247 (w), 1170 (m), 1114 (m), 881 (w), 767 (w), 725 (w) cm^{-1}.

m.p. 82 – 84 °C.

HRMS (ESI): m/z calcd for $C_{21}H_{27}O_2N_2^+$ $[M + H]^+$ 339.20670 found 339.20646.

SFC Chiralpak® IF; 1500 psi, 30°C; flow: 1.5 mL/min; from 1% to 30% MeOH in 5 min, then from 30% to 50% MeOH in 0.5 min; 99.5:0.5 er (major enantiomer t_R = 4.54 min; minor enantiomer t_R = 5.23 min).

$[\alpha]^{25}_D$ = –293.7 (*c* = 1.0, $CHCl_3$).

(–)-3ea and (–)-3ea*

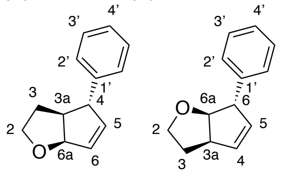

The corresponding compound was prepared following general procedure A using phenylboronic acid. The mixture was stirred at 65 °C for 15 h. The unpurified reaction mixture showed a regioisomeric ratio of (–)-**3ea** (dr 4:1) and (–)-**3ea*** (dr 5:1) of 1.00:1.04. Purification by flash chromatography (hexane/Et_2O = 90/10 to 70/30) first afforded the product (–)-**3ea*** (28 mg, 37% yield) as single diatereomer (dr >20:1) and later fractions gave a mixture of (–)-**3ea** and the minor diastereomer of (–)-**3ea*** (ratio 1.00:0.30:0.25) (40 mg, 54% yield) as a colourless oil. SFC analysis showed an enantiomeric excess of 98% for (–)-**3ea*** and 99% for (–)-**3ea***.

Analytical data for Fraction 1 ((–)-3ea*)

Determination of the relative stereochemistry:

- The dihedral angle between C(6a)–H and C(6)–H is close to 90°, therefore a small *J*-coupling is expected. The dihedral angle between C(3a)–H and C(6a)–H is close to zero, therefore a larger *J*-coupling is expected. The observed *J*-coupling between C(3a)–H and C(6a)–H is 6.0 Hz. C(6)–H (s) has a significantly smaller *J*-coupling to C(6a)–H, therefore also supporting the *trans* stereochemistry between C(6a)–H and C(6)–H.
- Consequently, the minor diastereomer shows for C(6a)–H similar *J* coupling to C(3a)–H and C(6)–H (app. t, *J* = 5.9 Hz), therefore supporting the *cis* stereochemistry between C(6a)–H and C(6)–H.

^{1}H NMR ($CDCl_3$, 400 MHz): δ (ppm) 7.34 – 7.27 (m, 2H, 2x C(3')–H), 7.24 – 7.18 (m, 1H, C(4')–H), 7.18 – 7.11 (m, 2H, 2x C(2')–H), 5.82 (dt, *J* = 4.9, 2.3 Hz, 1H, C(5)–H), 5.76 (dt, *J* = 5.7, 1.9 Hz, 1H, C(6)–H), 4.42 (d, *J* = 6.0 Hz, 1H, C(6a)–H), 3.89 (s, 1H, C(6)–H), 3.83 (ddd, *J* = 8.2, 7.4, 3.5 Hz, 1H, 1x C(2)–H_2), 3.75 (td, *J* = 8.8, 5.8 Hz, 1H, 1x C(2)–H_2), 3.51 (ddq, *J* = 8.3, 6.1, 2.1 Hz, 1H, C(3a)–H), 2.01 (dtd, *J* = 12.1, 8.9, 7.4 Hz, 1H, 1x C(3)–H_2), 1.80 (ddt, *J* = 11.9, 5.8, 3.1 Hz, 1H, 1x C(3)–H_2).

^{13}C NMR ($CDCl_3$, 100 MHz) δ (ppm) 143.0 (C(1')), 133.6 (C(5)), 133.4 (C(5)), 128.7 (2x C(3')), 127.6 (2x C(2')), 126.6 (C(4')), 90.2 (C(6a)), 67.1 (C(2)), 59.6 (C(6)), 49.9 (C(3a)), 31.2 (C(3)).

IR ($CHCl_3$ film) 3040 (w), 2936 (m), 2861 (m), 1600 (w), 1491 (w), 1450 (w), 1209 (w), 1069 (s), 1020 (w), 928 (w), 871 (w), 756 (m), 700 (m) cm^{-1}.

HRMS (ESI): m/z calcd for $C_{13}H_{15}O^+$ $[M + H]^+$ 187.11174 found 187.11202.

SFC Chiralpak® IC; 1500 psi, 30°C; flow: 1.5 mL/min; from 1% to 30% MeOH in 5 min; 99:1 er (minor enantiomer t_R = 1.72 min; major enantiomer t_R = 2.23 min).

$[\alpha]^{25}_D$ = –315.8 (*c* = 0.5, $CHCl_3$).

Analytical data for Fraction 2 ((–)-3ea with minor diastereomers)

Determination of the relative stereochemistry:

- The dihedral angle between C(6a)–H and C(4)–H is close to 90°, therefore a small *J*-coupling is expected. The dihedral angle between C(3a)–H and C(6a)–H is close to zero, therefore a larger *J*-coupling is expected. The observed mulitplicity for C(3a)–H is a ddq (*J* = 8.3, 6.1, 2.1 Hz). The *J*-coupling between C(3a)–H and C(6a)–H is 6.1 Hz. The *J* coupling between C(3a)–H and C(3)–H is 8.3 Hz. The *J* coupling between C(3a)–H and C(4)–H is significantly smaller, therefore also supporting the *trans* stereochemistry between C(3a)–H and C(4)–H.

^{1}H NMR ($CDCl_3$, 500 MHz; major diastereomer): δ (ppm) 7.33 – 7.28 (m, 2H, 2x C(3')–H), 7.25 – 7.19 (m, 1H, C(4')–H), 7.17 – 7.10 (m, 2H, C(2')–H), 5.95 (dd, *J* = 5.8, 2.3 Hz, 1H, C(5)–H), 5.87 (dt, *J* = 5.6, 2.1 Hz, 1H, C(6)–H), 5.30 (dt, *J* = 7.0, 2.2 Hz, 1H, C(6a)–H), 3.85 (ddd, *J* = 8.5, 6.9, 3.8 Hz, 1H, 1x C(2)–H_2), 3.76 – 3.63 (m, 2H, 1x C(2)–H_2 and C(4)–H), 2.80 – 2.72 (m, 1H, C(3a)–H), 2.15 – 2.02 (m, 1H, 1x C(3)–H_2), 1.84 (ddt, *J* = 12.5, 5.6, 3.6 Hz, 1H, 1x C(3)–H_2).

^{13}C NMR ($CDCl_3$, 125 MHz; major diastereomer) δ (ppm) 145.0 (C(1')), 138.4 (C(5)), 131.2 (C(6)), 128.8 (2x C(3')), 127.2 (2x C(2')), 126.6 (C(4')), 88.8 (C(6a)), 66.3 (C(2)), 59.2 (C(4)), 50.5 (C(3a)), 34.6 (C(3)).

IR ($CHCl_3$ film) 3058 (w), 3026 (w), 2940 (w), 2862 (w), 1602 (w), 1492 (w), 1453 (w), 1360 (w), 1058 (m), 1031 (w), 928 (w), 906 (w), 867 (w), 770 (m), 748 (m), 700 (s) cm^{-1}.

HRMS (ESI): m/z calcd for $C_{13}H_{15}O^+$ $[M + H]^+$ 187.11174 found 187.11197.

SFC Chiralpak® IC; 1500 psi, 30°C; flow: 1.5 mL/min; from 1% to 30% MeOH in 5 min; 99.5:0.5 er (major enantiomer t_R = 1.96 min; minor enantiomer t_R = 2.02 min).

$[\alpha]^{25}_D$ = –210.8 (*c* = 0.5, $CHCl_3$).

1.3. Derivatization Experiments

(–)-4

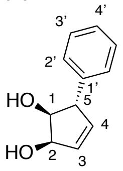

A solution of (–)-**3aa** (492 mg, 2.00 mmol) in AcOH (4.4 mL) and H_2O (1.9 mL) was stirred for 24 h at 40 °C. The reaction mixture was concentrated under reduced pressure and purification by flash chromatography (cyclohexane/EtOAc = 70/30 to 0/100) afforded the product (–)-**4** as a colorless solid (347 mg, 98% yield) as a single diastereomer (dr >20:1).

^{1}H NMR ($CDCl_3$, 400 MHz): δ (ppm) 7.38 – 7.29 (m, 2H, 2x C(3')–H), 7.28 – 7.22 (m, 1H, C(4')–H), 7.22 – 7.15 (m, 2H, 2x C(2')–H), 6.14 – 5.83 (m, 2H, C(3)–H and C(4)–H), 4.69 (tt, *J* = 5.8, 1.5 Hz, 1H, C(2)–H), 4.03 (dt, *J* = 7.1, 5.3 Hz, 1H, C(1)–H), 3.85 (dd, *J* = 4.9, 1.3 Hz, 1H, C(5)–H), 2.79 (d, *J* = 7.1 Hz, 1H, C(1)–OH), 2.27 (d, *J* = 6.0 Hz, 1H, C(2)–OH).

^{13}C NMR ($CDCl_3$, 100 MHz) δ (ppm) 141.8 (C(1')), 138.3 (C(4)), 132.1 (C(3)), 128.8 (2x C(3')), 127.4 (2x C(2')), 127.0 (2x C(4')), 79.9 (C(1)), 75.4 (C(2)), 57.1 (C(5)).

IR ($CHCl_3$ film) 3341 (br. s), 3060 (m), 2916 (m) 1600 (w), 1494 (w), 1443 (w), 1417 (w), 1318 (m), 1207 (m), 1105 (s), 1033 (m), 965 (w), 857 (w), 755 (s), 700 (s) cm^{-1}.

m.p. 45 – 47 °C.

HRMS (ESI): m/z calcd for $C_{11}H_{12}O_2Na^+$ $[M + Na]^+$ 199.07295 found 199.07305.

$[\alpha]^{25}_{D}$ = –282.1 (*c* = 1.0, $CHCl_3$).

(–)-5

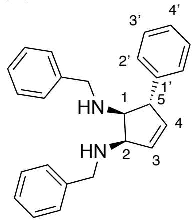

A round bottom flask equipped with a reflux condensor was charged with (–)-**3ca** (63 mg, 150 µmol) and Et_2O (1.8 mL). A solution of lithium aluminium hydride (4.0 M; 250 µL, 900 µmol) was added and the reaction mixture was stirred while heating to reflux for 16 h. H_2O (40 µL), an aq. sol. of NaOH (1N; 80 µL) and again H_2O (40 µL) were added subsequently. The mixture was concentrated under reduced pressure and purification by flash chromatography (EtOAc/MeOH = 98/2) afforded the product (–)-**5** as a pale yellow oil (49 mg, 93% yield) as a single diastereomer (dr >20:1).

^{1}H NMR ($CDCl_3$, 400 MHz): δ (ppm) 7.37 – 7.13 (m, 15H, 15x C(Ar)–H), 6.06 (dt, *J* = 5.9, 2.3 Hz, 1H, C(3)–H), 5.87 (ddd, *J* = 5.9, 2.0, 0.9 Hz, 1H, C(4)–H), 3.86 (d, *J* = 13.1 Hz, 1H, 1x PhC*H*$_2$), 3.80 – 3.74 (m, 2H, 1x PhC*H*$_2$ and C(2)–H), 3.73 – 3.69 (m, 2H, 1x PhC*H*$_2$ and C(5)–H), 3.66 (d, *J* = 13.4 Hz, 1H, 1x PhC*H*$_2$), 3.10 (app. t, *J* = 6.5 Hz, 1H, C(1)–H), 1.93 (s, 2H, 2x NH).

^{13}C NMR ($CDCl_3$, 100 MHz) δ (ppm) 143.4, 141.0, 140.7 (3x $C(Ar)_{quart}$), 136.0 (C(4)), 133.8 (C(3)), 128.6, 128.5, 128.4, 128.1, 127.7, 127.0, 126.9, 126.6 (15x C(Ar)–H), 68.8 (C(1)), 62.7, 56.8, 52.9, 52.8 (2x $PhCH_2$)

IR ($CHCl_3$ film) 3305 (w), 3029 (w), 2915 (w), 2845 (w), 1959 (w), 1491 (w), 1452 (m), 1202 (w), 1137 (w), 1134 (w), 1081 (w), 1029 (w), 980 (w), 741 (s), 664 (s) cm^{-1}.

HRMS (ESI): m/z calcd for $C_{25}H_{27}N_2^+$ $[M + H]^+$ 355.21688 found 355.21656.

$[\alpha]^{25}_{D}$ = –216.4 (*c* = 0.5, $CHCl_3$).

(–)-6

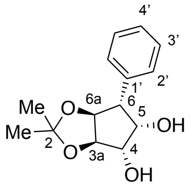

N-Methylmorpholine *N*-oxide (70.2 mg, 600 µmol) and $K_2OsO_6{\cdot}H_2O$ (5.9 mg, 16 µmol) were added to a solution of (–)-**3aa** (86.4 mg, 400 µmol) in acetone (650 µL) and H_2O (160 µL) and the mixture was stirred for 18 h at 23 °C. A sat. solution of $Na_2S_2O_3$ (2 mL) was added. After stirring the mixture for 20 min, the aqueous layer was extracted with EtOAc (3x 5 mL) and the combined organic layer was dried over $MgSO_4$. Purification by flash chromatography (hexane/EtOAc = 30/70) afforded the product **(–)-6** as a yellow oil (91 mg, 91% yield) with a dr of 4:1.

Determination of the relative stereochemistry:

- *Cis* stereochemistry between C(6a)–H and C(3a)–H and was set in the previous reaction step.
- *Trans* stereochemistry between C(6a)–H and C(6)–H was set in the previous reaction step.
- Strong NOE between C(4)–H and C(5)–H indicates *cis* stereochemistry between C(4)–H and C(5)–H.
- Strong NOE between C(6)–H and C(5)–H indicates *cis* stereochemistry between C(6)–H and C(5)–H.
- Very weak NOE between C(3a)–H and C(4)–H indicates *trans* stereochemistry between C(3a)–H and C(4)–H.
- For the minor diastereomer: Absence of NOE C(6)–H and C(5)–H indicates *trans* stereochemistry between C(6)–H and C(5)–H and NOE between C(3a)–H and C(4)–H indicates *cis* stereochemistry between C(3a)–H and C(4)–H

^{1}H NMR ($CDCl_3$, 500 MHz; major diastereomer): δ (ppm) 7.38 (app. d, *J* = 4.4 Hz, 4H, 2x C(3')–H and 2x C(2')–H), 7.32 – 7.25 (m, 1H, C(4')–H), 5.06 (t, *J* = 7.0 Hz, 1H, (C(6a)–H), 4.67 (dt, *J* = 7.3, 3.8 Hz, 1H, C(3a)–H), 4.40 (dt, *J* = 6.3, 3.0 Hz, 1H, C(5)–H), 4.25 (q, *J* = 4.2 Hz, 1H, C(4)–H), 3.27 (dd, *J* = 6.5, 4.0 Hz, 1H, C(6)–H), 3.01 (t, *J* = 4.4 Hz, 1H, OH), 2.45 (d, *J* = 3.0 Hz, 1H, OH), 1.54 (s, 3H, CH_3), 1.36 (s, 3H, CH_3)

^{13}C NMR ($CDCl_3$, 100 MHz; major diastereomer) δ (ppm) 137.3 (C(1')), 128.8, 128.7 (2x C(2') and 2x C(3')), 127.2 (C(4')), 113.2 (C(2)), 86.1 (C(3a)), 83.2 (C(6a)), 79.2 (C(4)), 78.0 (C(5)), 54.3 (C(6)), 27.3 (CH_3), 24.7 (CH_3).

IR ($CHCl_3$ film) 3428 (br. m), 2980 (s), 1494 (w), 1457 (w), 1381 (s), 1259 (m), 1209 (m), 1155 (m), 1063 (s), 955 (m), 866 (w), 749 (w), 700 (m) cm^{-1}.

HRMS (ESI): m/z calcd for $C_{14}H_{18}O_4Na^+$ $[M + Na]^+$ 273.10973 found 273.10972.

$[\alpha]^{25}_D$ = –55.1 (*c* = 1.0, $CHCl_3$).

1.4. Mechanistic Studies

$[Rh(cod)OH]_2$ (9.2 mg, 0.020 mmol, 2.5 mol%) and (*S*)-Segphos (29.4 mg, 4.8 mmol 6.0 mol%) were added to a flame dried, septum sealed, 10 mL round bottom flask under an argon atmosphere and dissolved in THF (1.4 mL). CsOH (50 wt% aq. sol., 140 µL, 0.80 mmol, 1.00 eq) was added and the mixture was stirred while hearting at 65 °C. After 30 min the mixture was then cooled to 40 °C at heating and stirring was maintained at 40 °C. A solution of phenylboronic acid (196 mg, 1.60 mmol, 2.00 eq) and allylic chloride (±)-**1a** (122 µL, 0.80 mmol, 1.00 eq) in THF (1.8 mL) was added via syringe. Samples of (~50 µL) were taken *via* syringe, then diluted with hexane (1 mL), filtered, concentrated in vacuo (200 mbar, 40 ºC) and then analyzed by ^{1}H NMR spectroscopy (without internal standard), followed by SFC, followed by GC.

The conversion was determined by integration and taking the mean of the two protons C(3a)–H and C(4)–H in the starting material and the product and calculating the ratio [**3aa**]/([**1a**]+[**3aa**]).
*(As we do not see any significant side-products derived from **1a** during the reaction, we believe that this is a good approximation for conversion.)*

The ee of the product (–)-**3aa** was determined by super critical super fluid chromatography (SFC):
Chiralpak® IF; 1500 psi, 30°C; flow: 1.5 mL/min; from 1% to 30% MeOH in 5 min; (major enantiomer t_R = 1.58 min; minor enantiomer t_R = 1.64 min).

The ee of the allylic chloride (±)-**1a** was determined by gas chromatography (GC):
Lipodex E column; initial temperature 60 °C, initial hold time 0 min, progress rate 1 °C/min, final temperature 190 °C; flow rate 2.8 mL/min; minor enantiomer t_R = 12.83 min; major enantiomer t_R = 13.53 min).

2. References

[1] R. Uson, L. A. Oro, J. A. Cabeza, E. Bryndza, M. P. Stepro in *Inorg. Synth. Vol. 23* (Ed.: S. Kirschner), John Wiley & Sons, Inc., Hoboken, NJ, USA, **1985**, pp. 126–130.
[2] S. P. Fletcher, M. Sidera, Oxford University Innovation WO2016/198836 (**2016**).
[3] M. E. Jung, J. A. Berliner, D. Angst, D. Yue, L. Koroniak, A. D. Watson, R. Li, *Org. Lett.* **2005**, 7, 3933–3935.
[4] B. M. Trost, M. T. Sorum, *Org. Process Res. Dev*. **2003**, 7, 432–435.
[5] N. Palani, A. Chadha, K. K. Balasubramanian, *J. Org. Chem*. **1998**, 63, 5318–5323.
[6] C. Wu, P. D. Bartlett, *J. Org. Chem.* **1985**, 50, 733–740.
[7] R. I. McDonald, S. S. Stahl, *Angew. Chem. Int. Ed.* **2010**, *49*, 5529–5532; Angew. Chem. **2010**, *122*, 5661–5664.
[8] M. Rönn, J. E. Bäckvall, P. G. Andersson, *Tetrahedron Lett*. **1995**, 36, 7749–7752.
[9] M. Sidera, S. P. Fletcher, *Nat. Chem.* **2015**, 7, 935–939.
[10] P. Schäfer, T. Palacin, M. Sidera, S. P. Fletcher, *Nat. Commun.* **2017**, 8, 15762.
[11] J.-C. Fiaud, J.-Y. Legros, *J. Org. Chem.* **1987**, *52*, 1907–1911.

3. NMR spectra

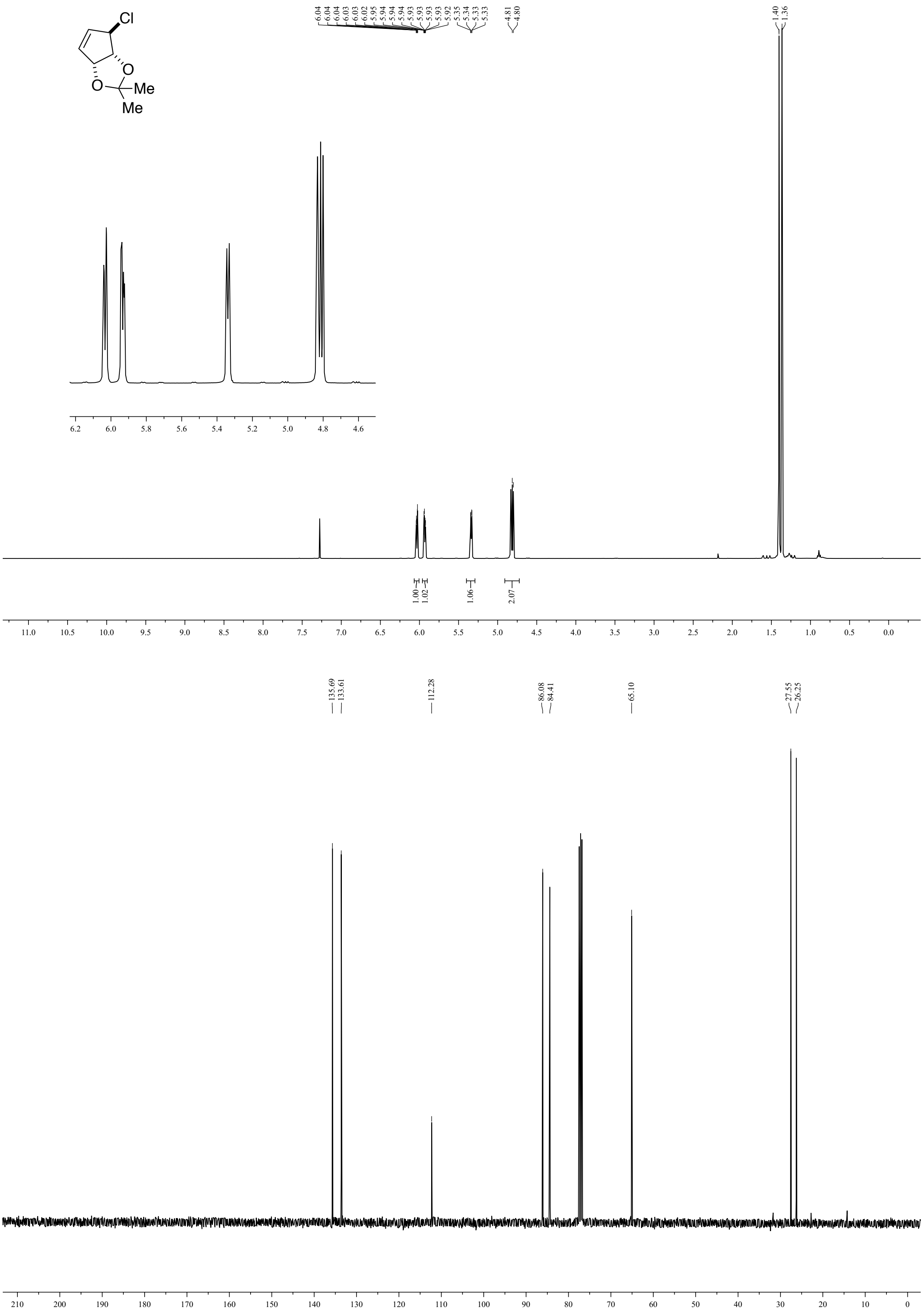

Figure S1: ^{1}H-NMR (top) and ^{13}C-NMR (bottom) of (±)-**1a**.

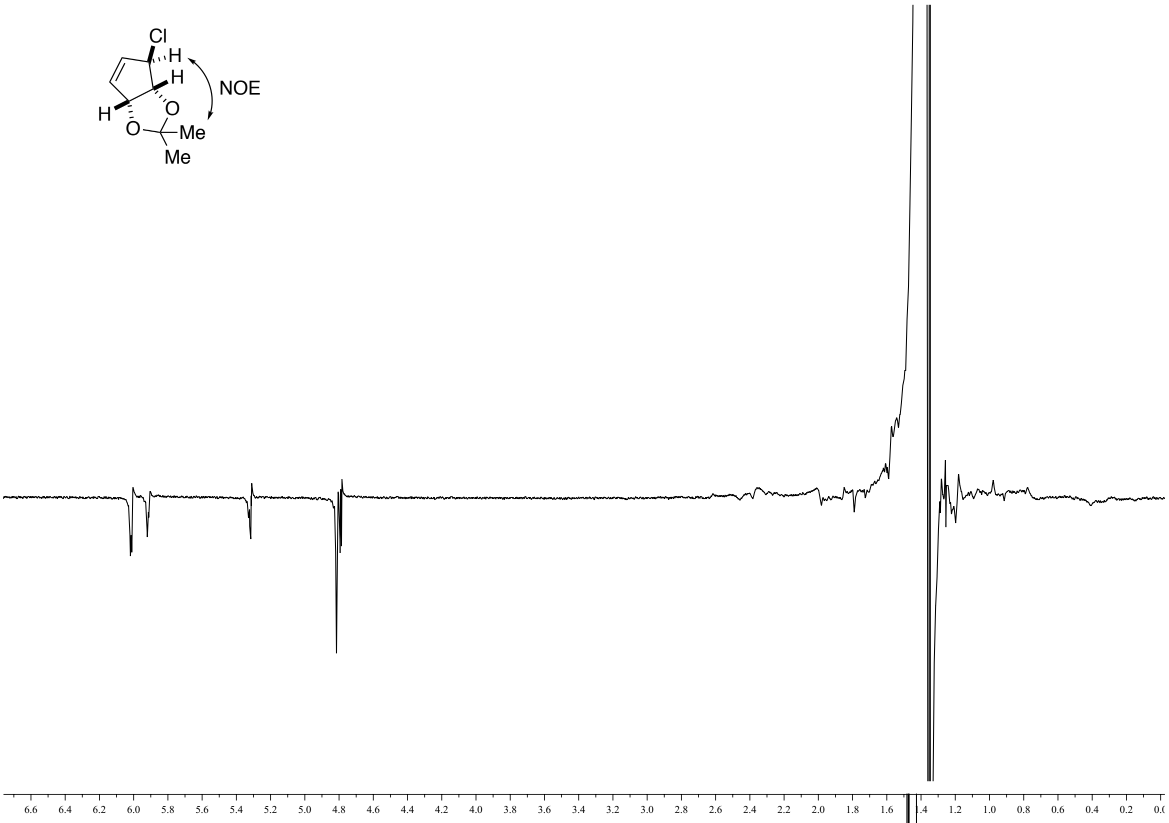

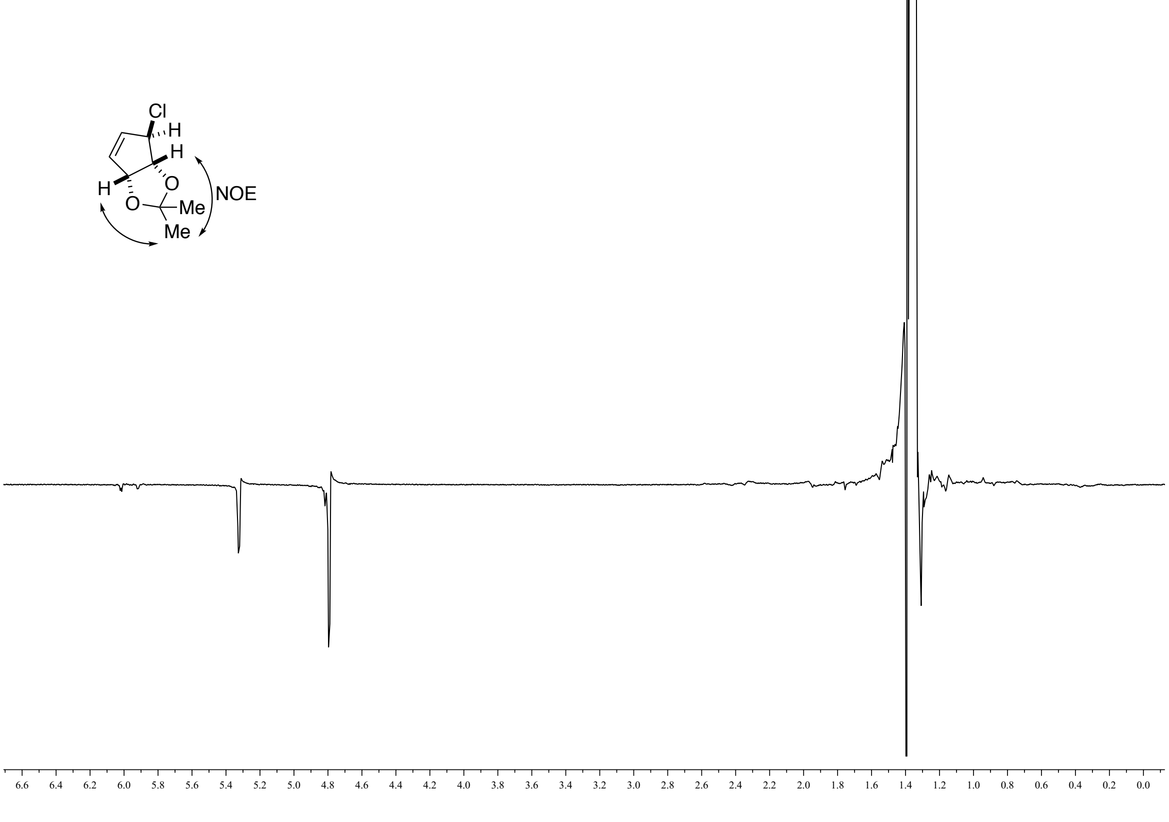

Figure S2: 1D NOEs of (±)-**1a**.

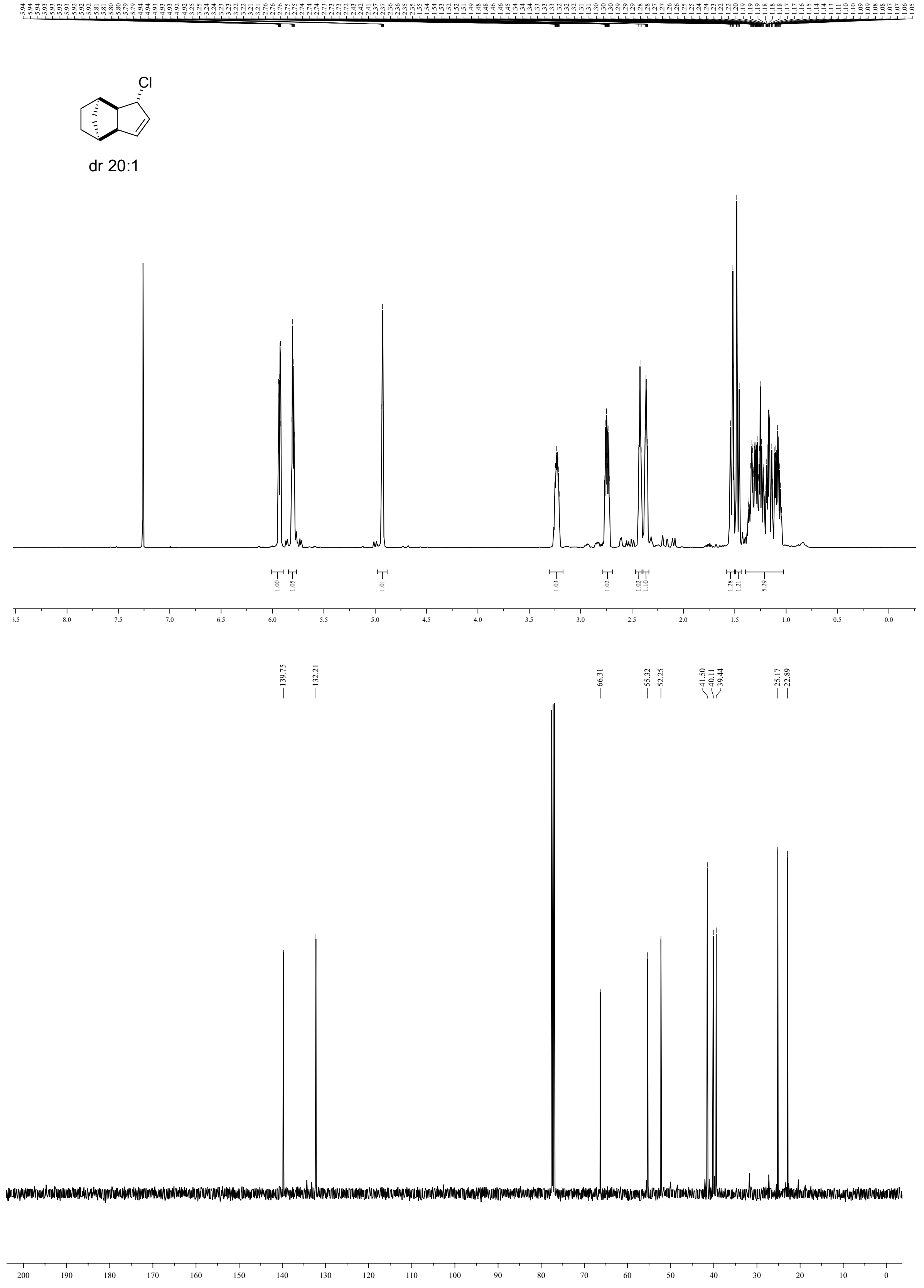

Figure S3: ^{1}H-NMR (top) and ^{13}C-NMR (bottom) of (±)-**1b**.

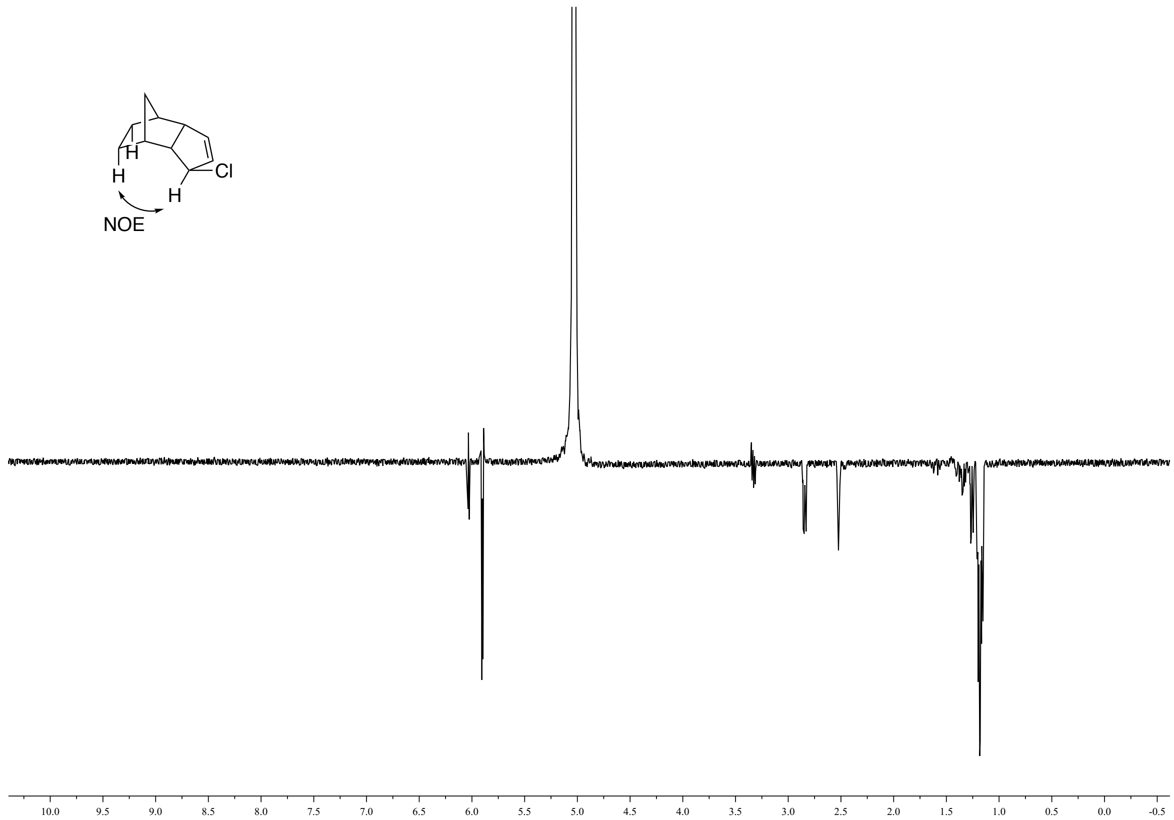

Figure S4: 1D NOE of (±)-**1b**.

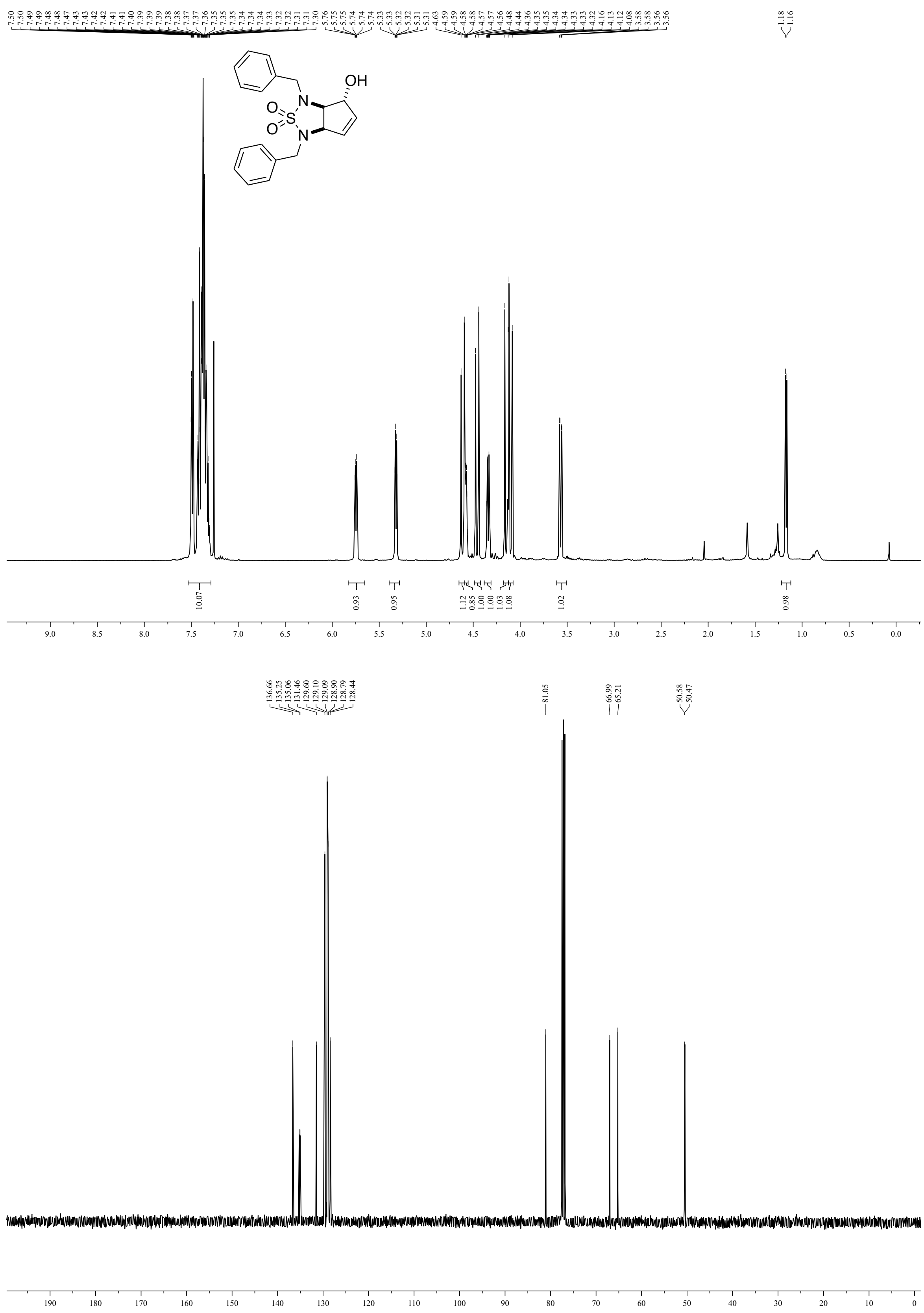

Figure S5: ^{1}H-NMR (top) and ^{13}C-NMR (bottom) of (±)-**S9**.

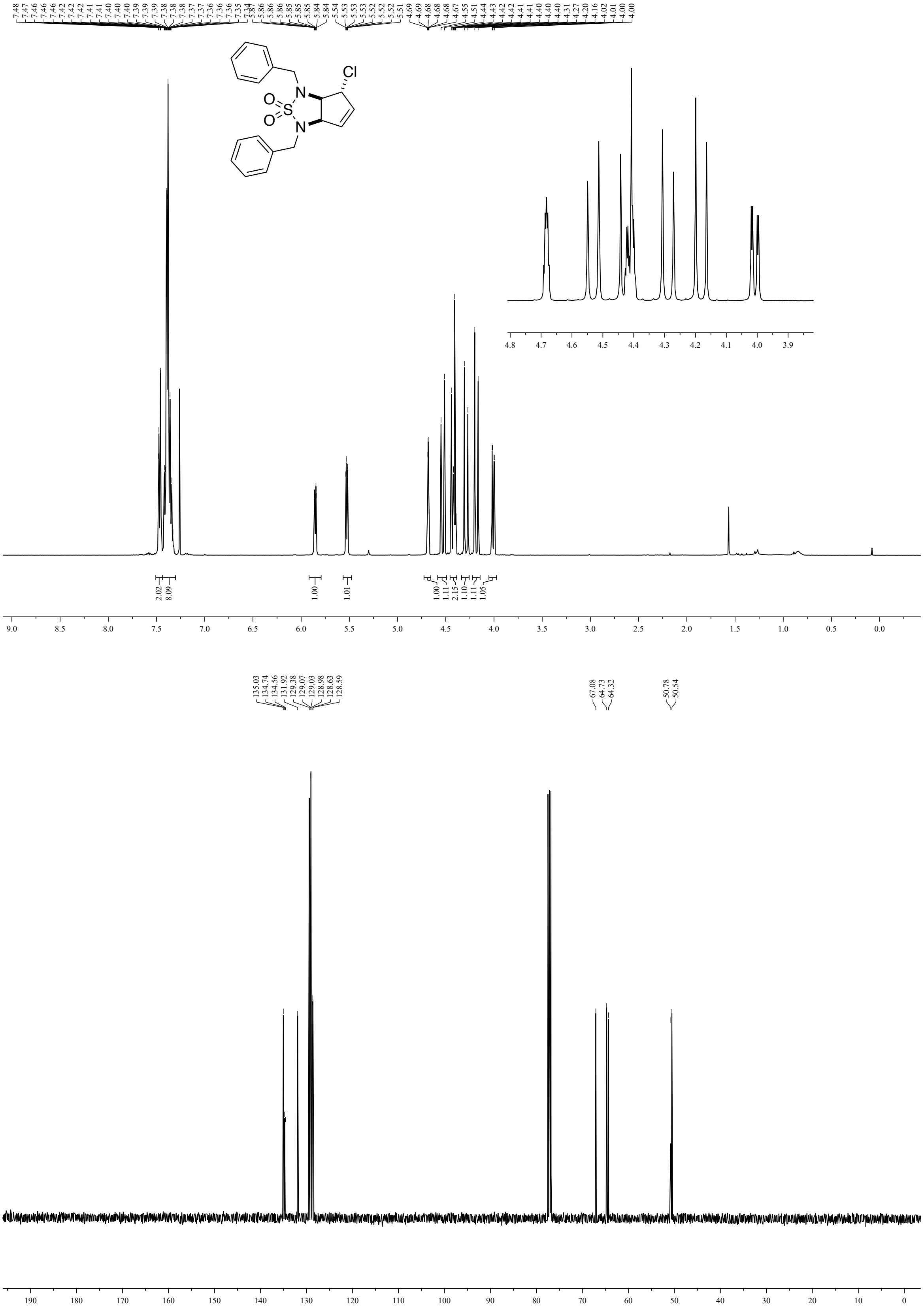

Figure S6: ^{1}H-NMR (top) and ^{13}C-NMR (bottom) of (±)-**1c**.

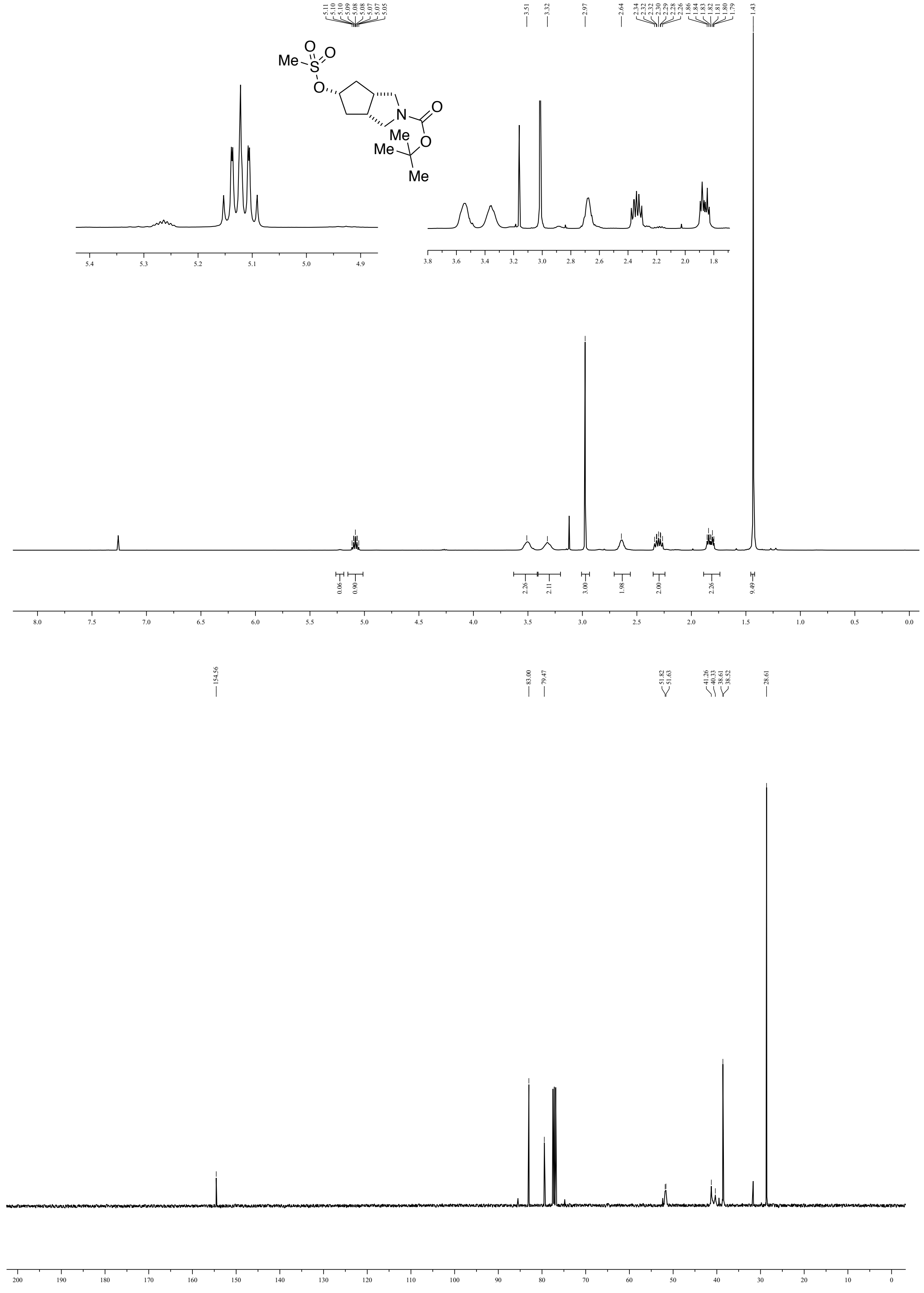

Figure S7: ^{1}H-NMR (top) and ^{13}C-NMR (bottom) of **S11**.

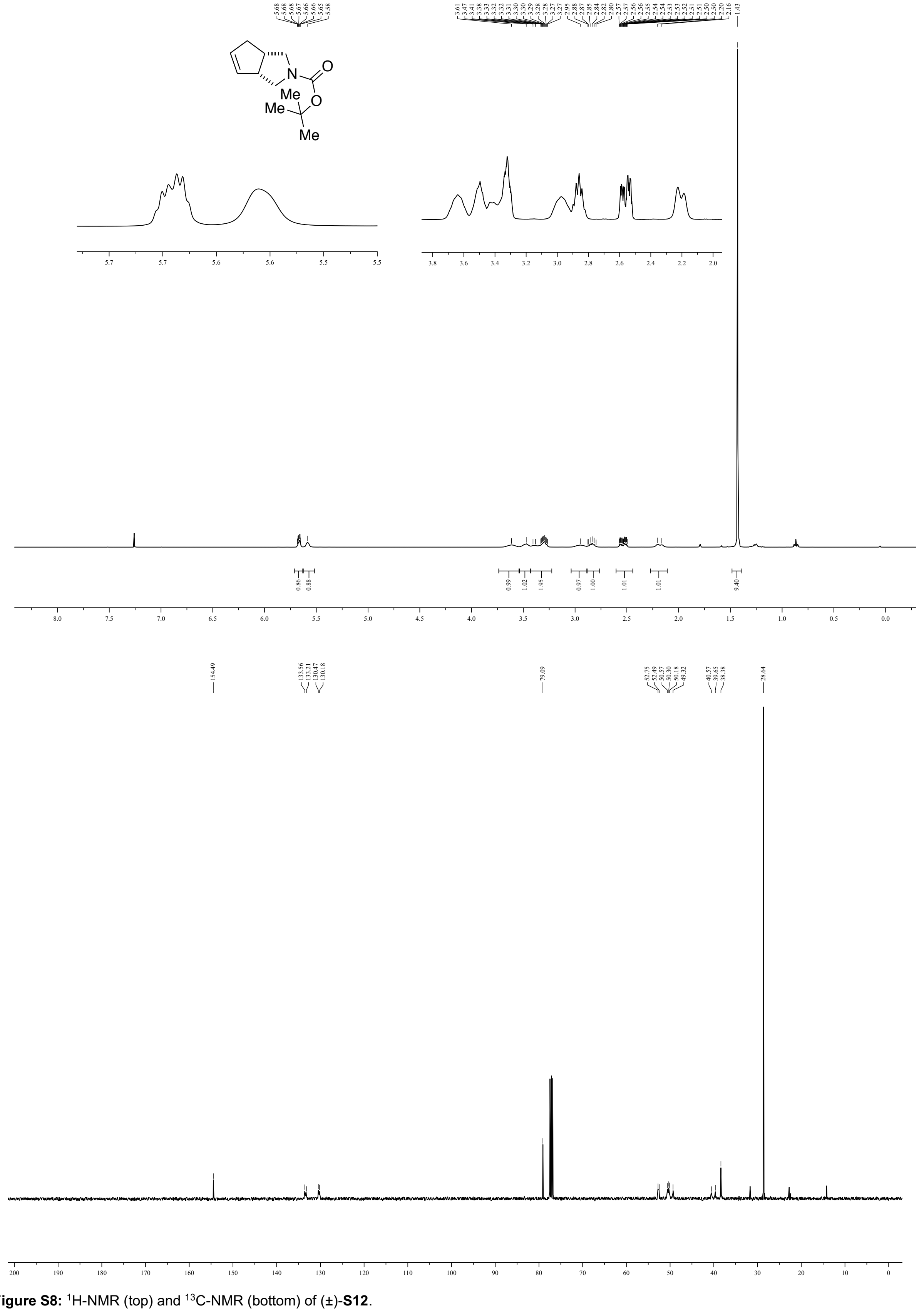

Figure S8: ^{1}H-NMR (top) and ^{13}C-NMR (bottom) of (±)-**S12**.

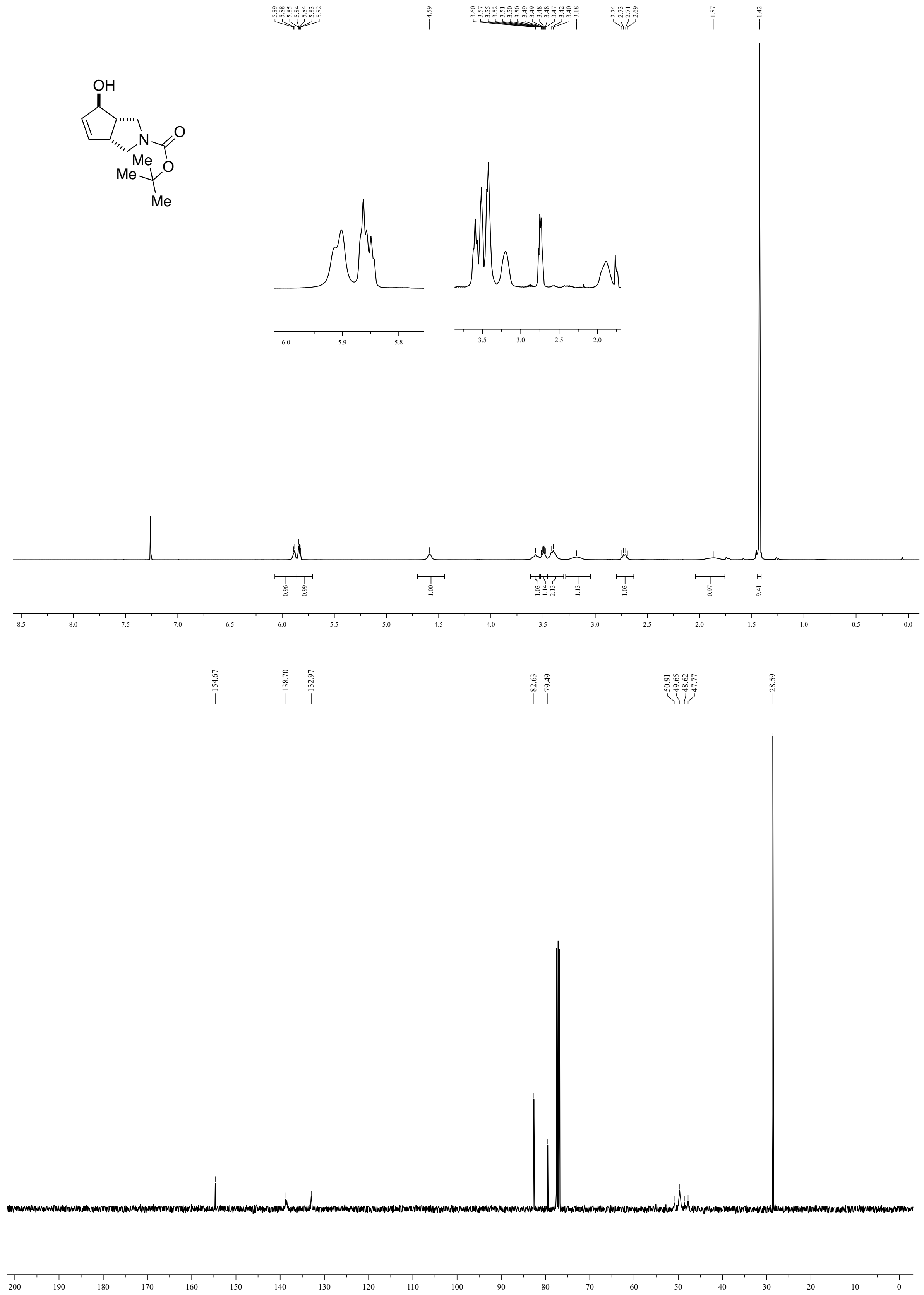

Figure S9: ^{1}H-NMR (top) and ^{13}C-NMR (bottom) of (±)-**S13**.

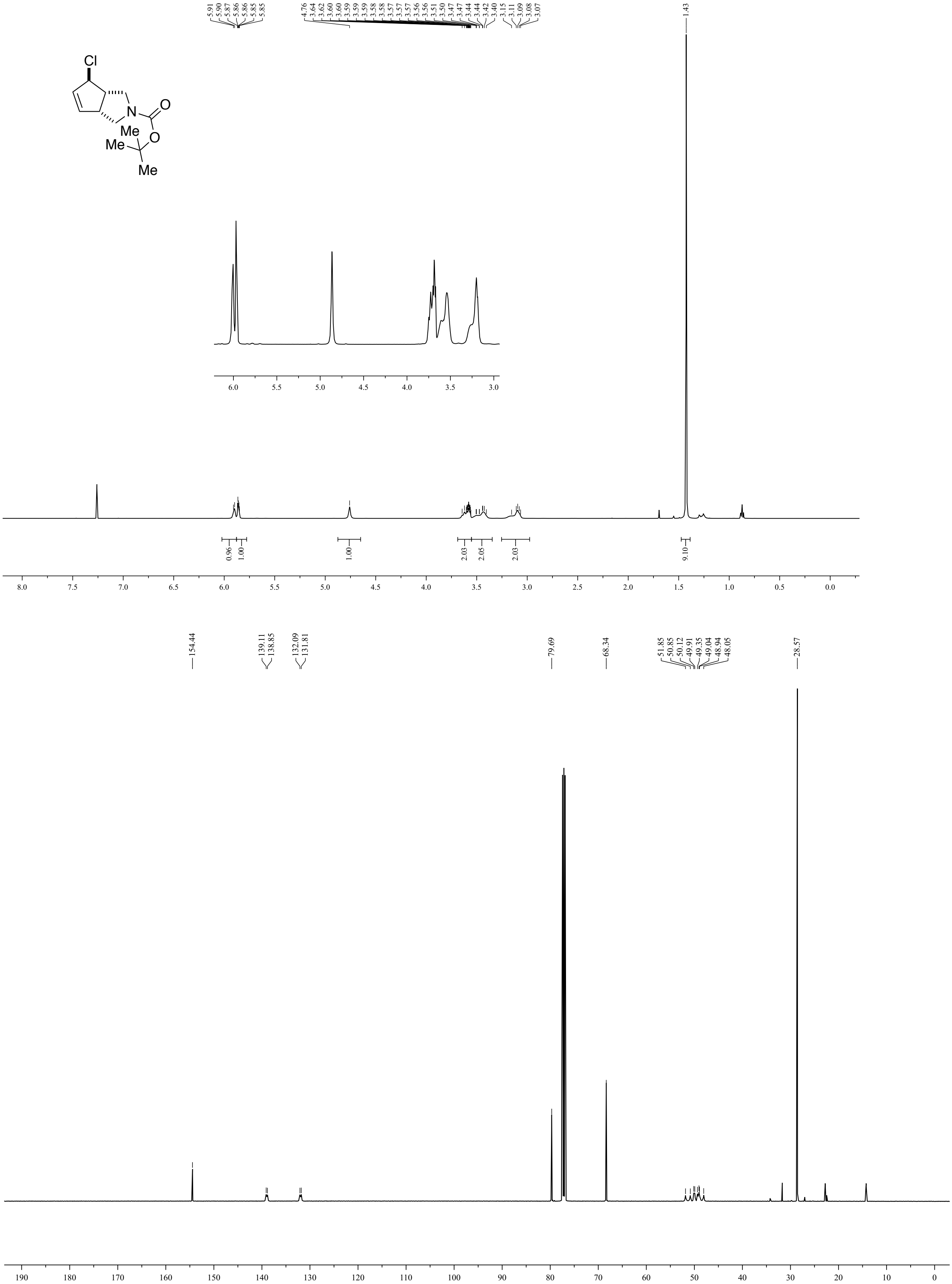

Figure S10: ^{1}H-NMR (top) and ^{13}C-NMR (bottom) of (±)-**1d**.

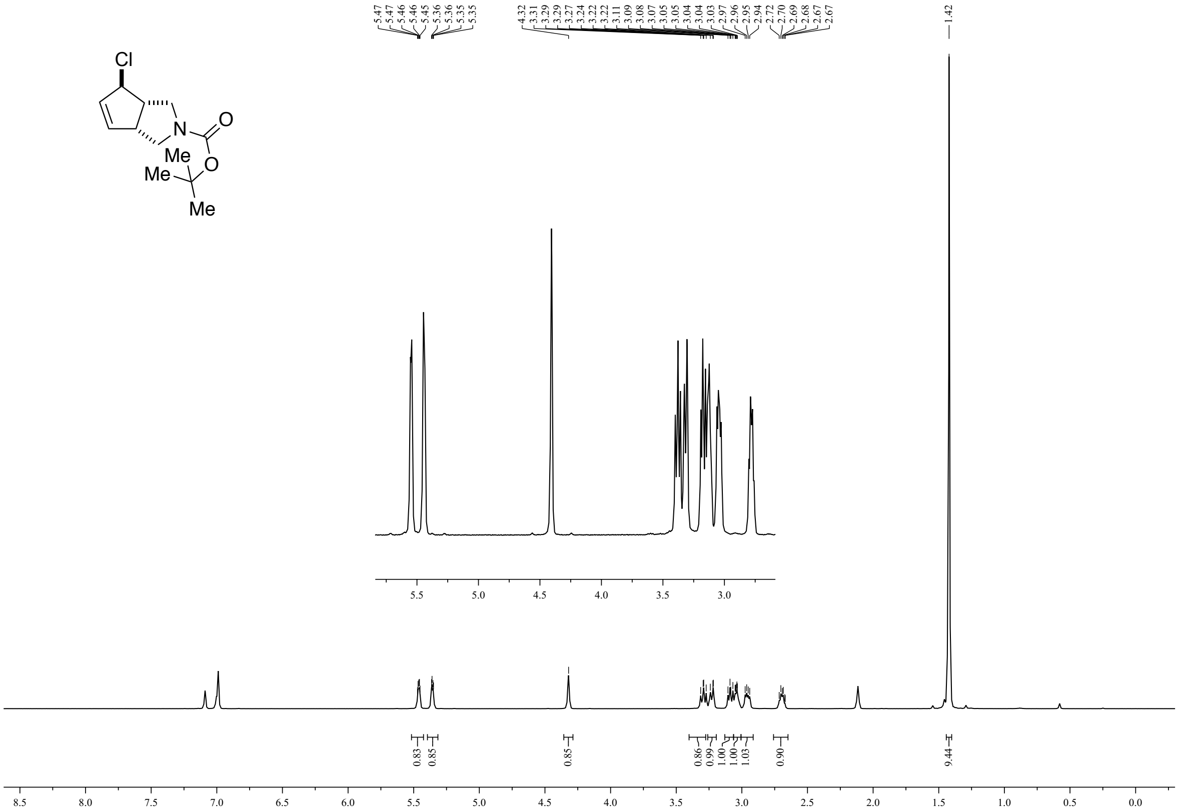

Figure S11: High-temperature (363 K) NMR of (±)-**1d**.

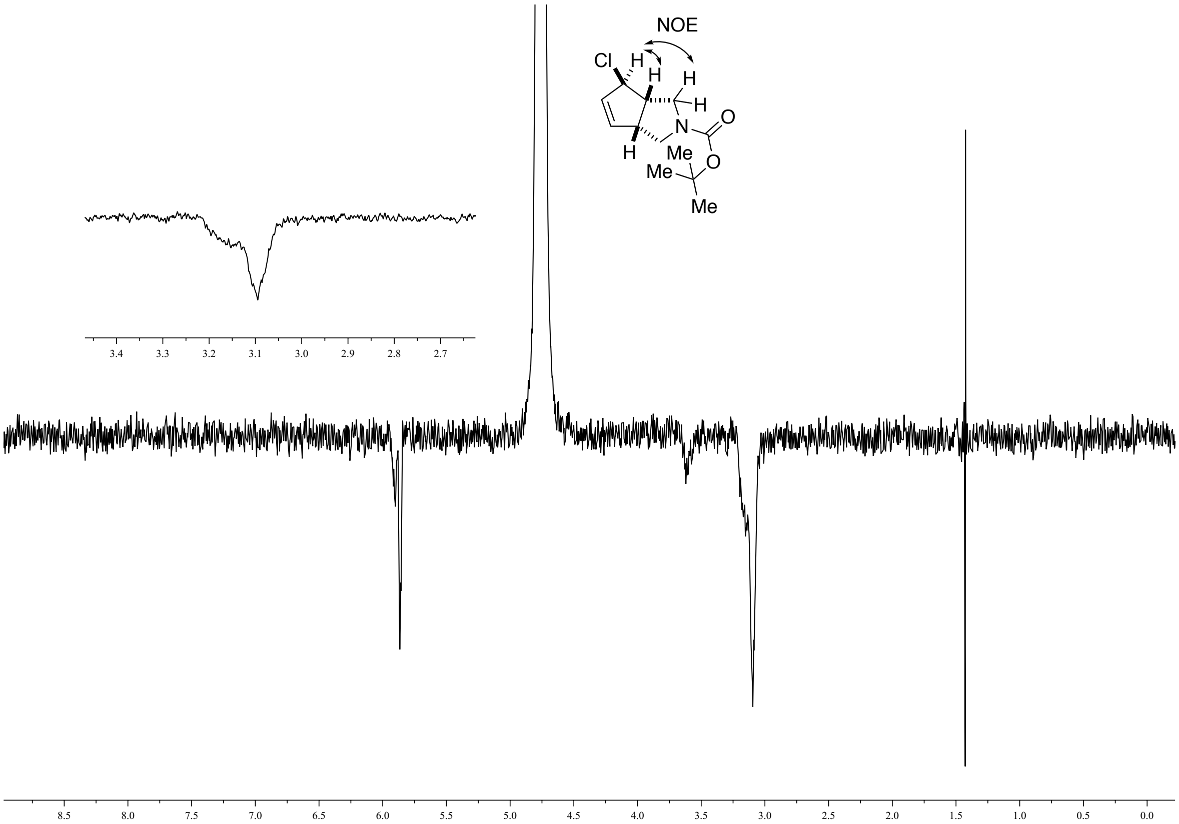

Figure S12: 1D NOE of (±)-**1d**.

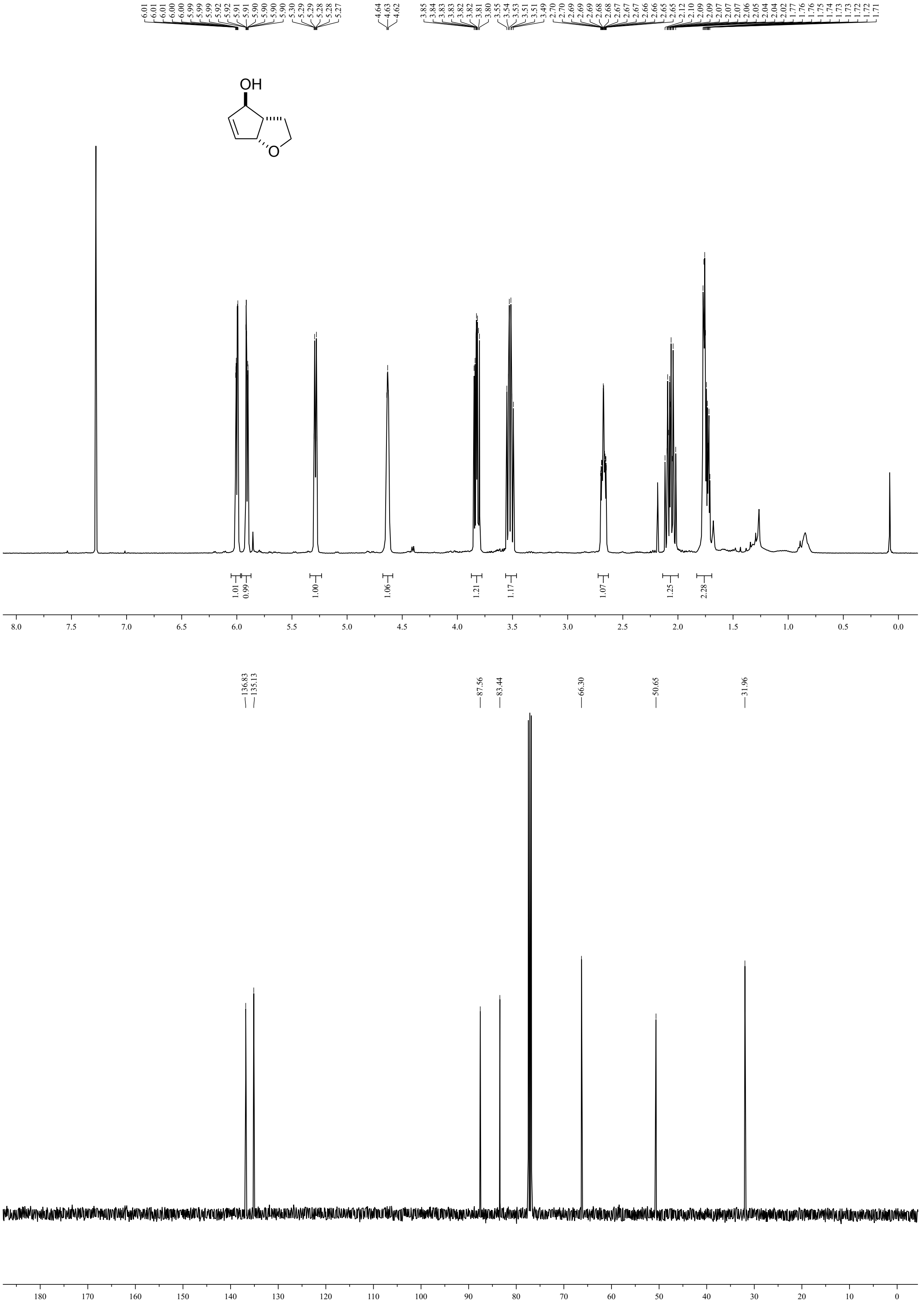

Figure S13: ^{1}H-NMR (top) and ^{13}C-NMR (bottom) of (±)-**S15**.

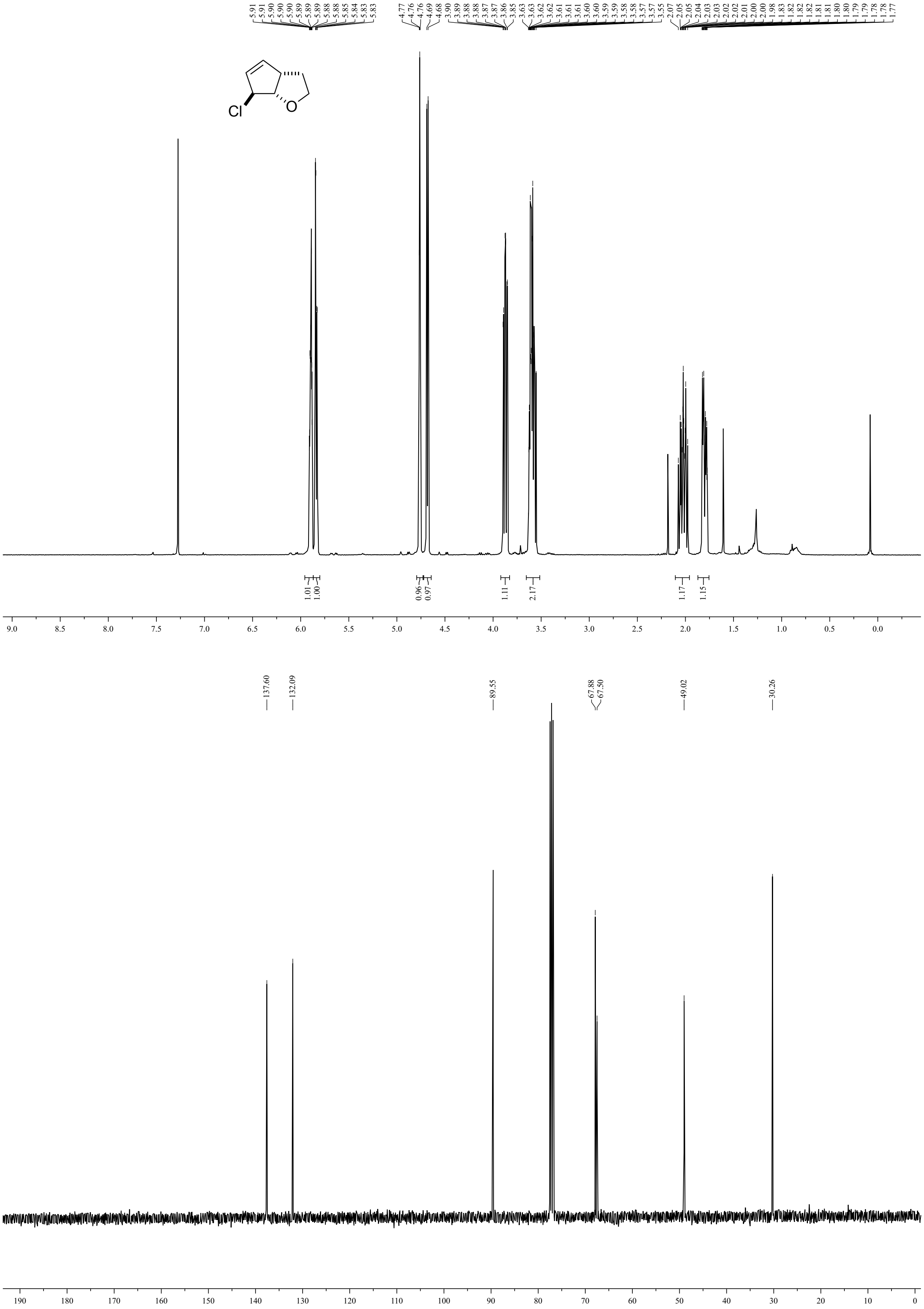

Figure S14: ^{1}H-NMR (top) and ^{13}C-NMR (bottom) of (±)-**1e**.

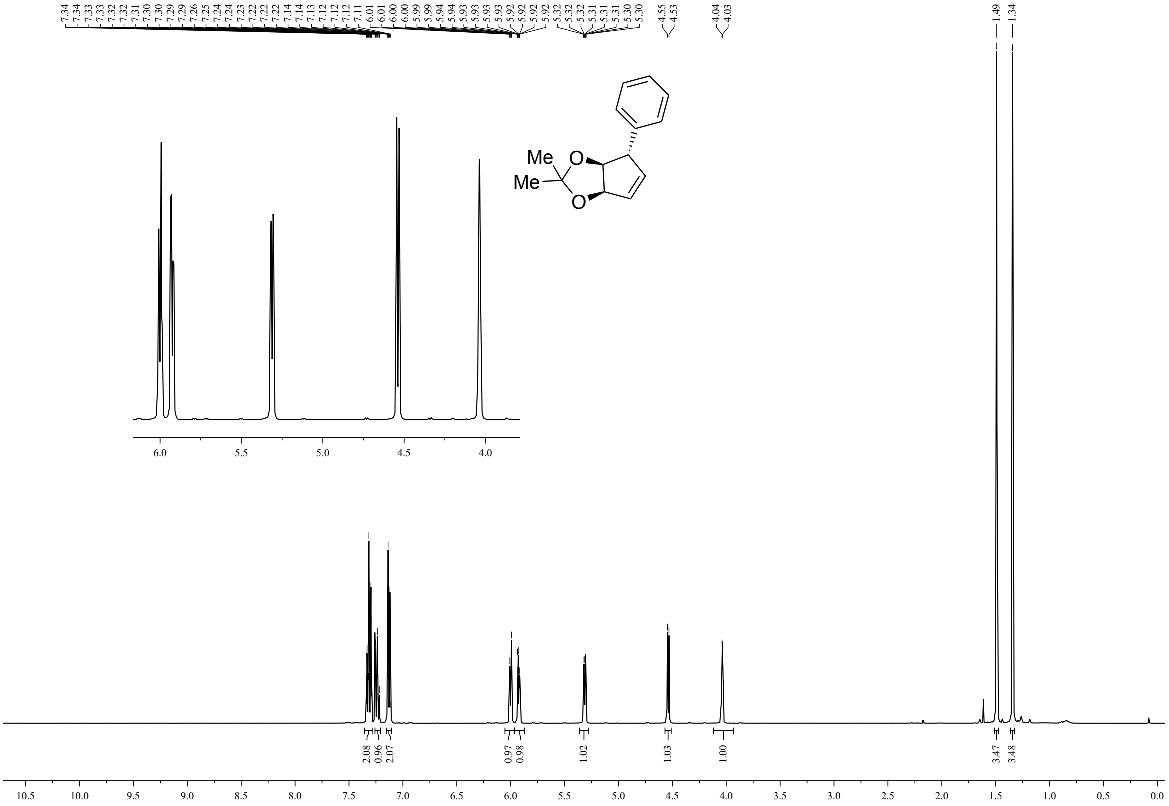

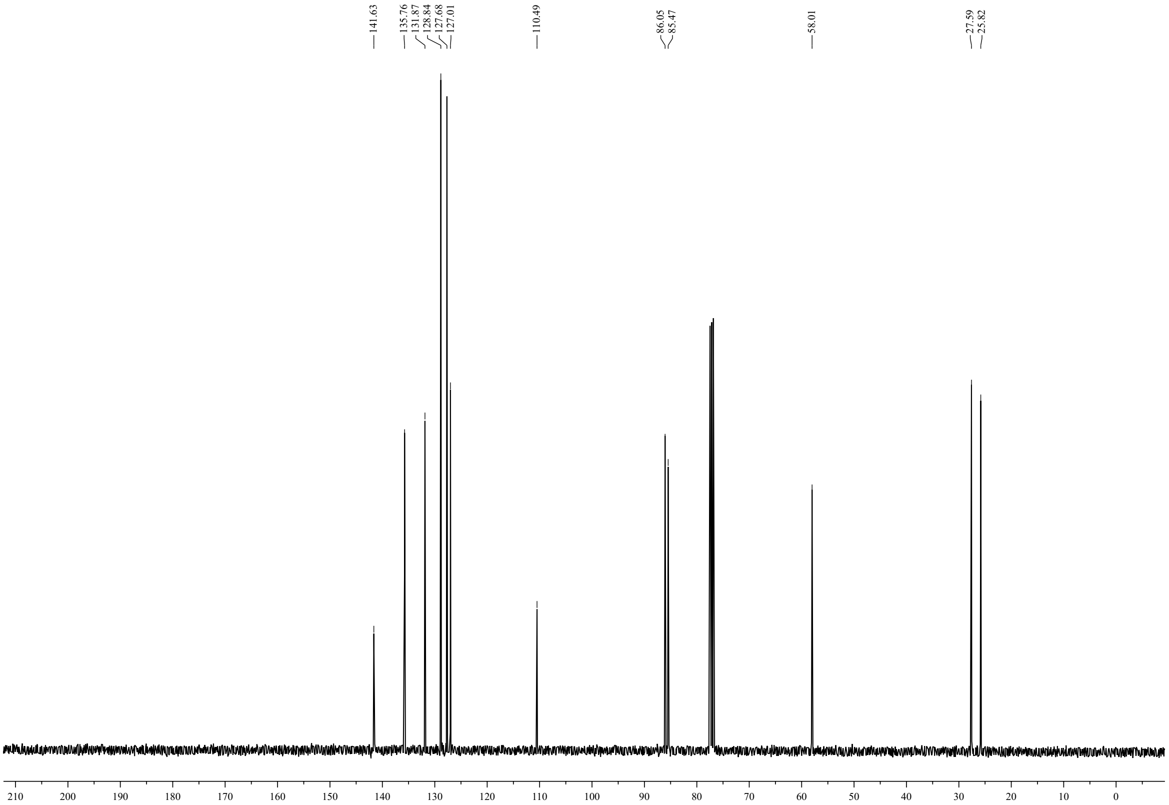

Figure S15: ^{1}H-NMR (top) and ^{13}C-NMR (bottom) of (–)-**3aa**.

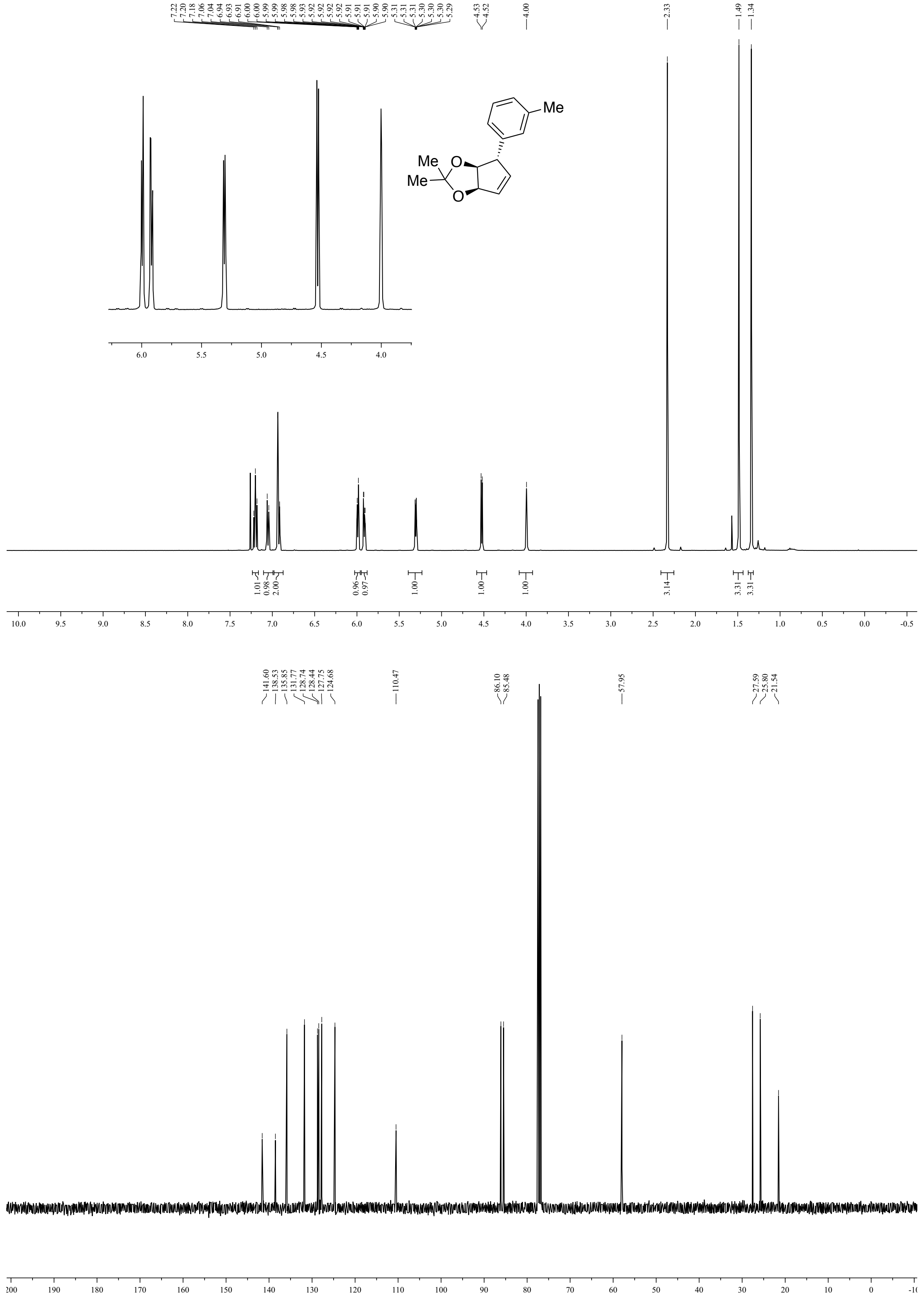

Figure S16: ^{1}H-NMR (top) and ^{13}C-NMR (bottom) of (–)-**3ab**.

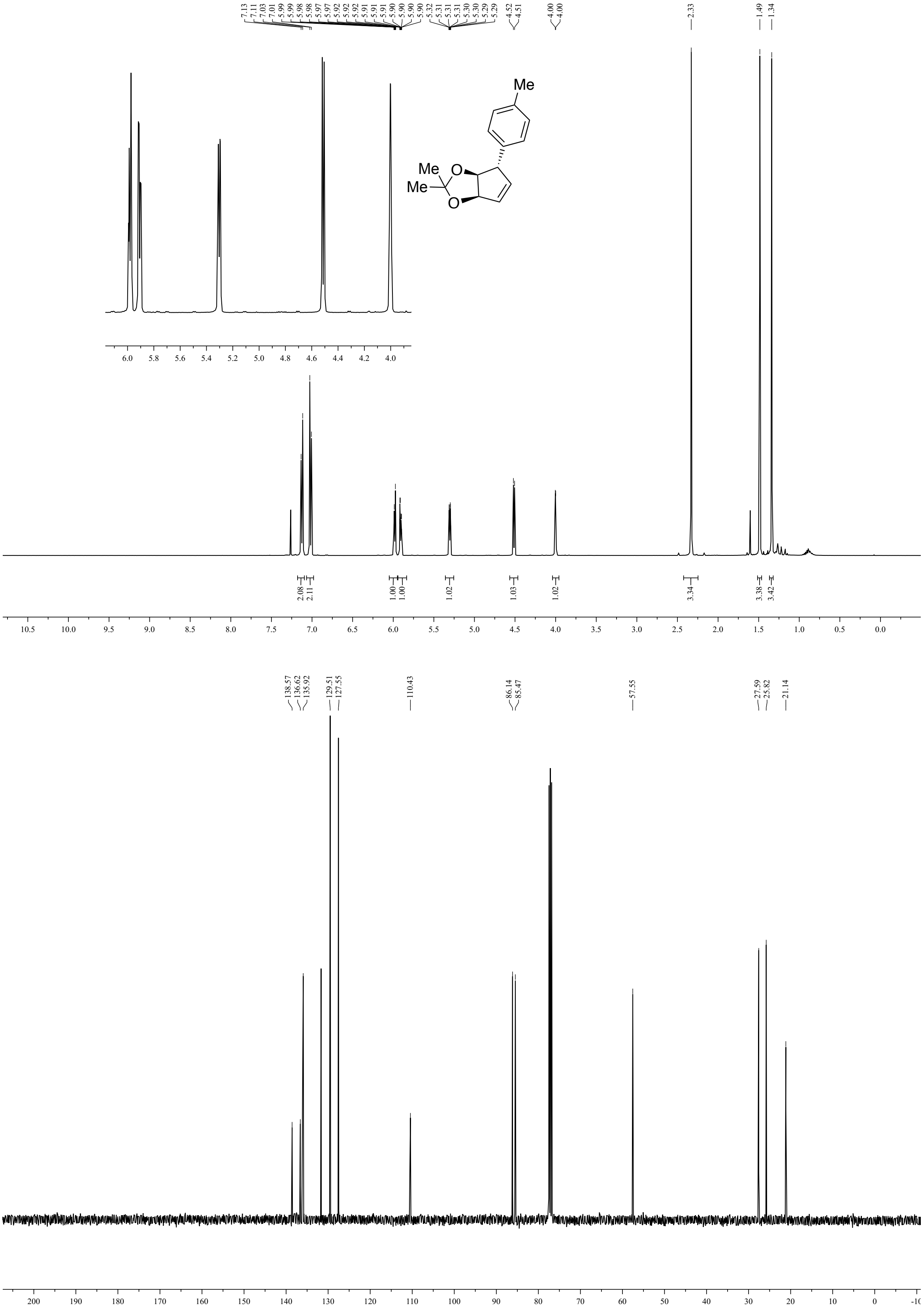

Figure S17: ^{1}H-NMR (top) and ^{13}C-NMR (bottom) of (–)-**3ac**.

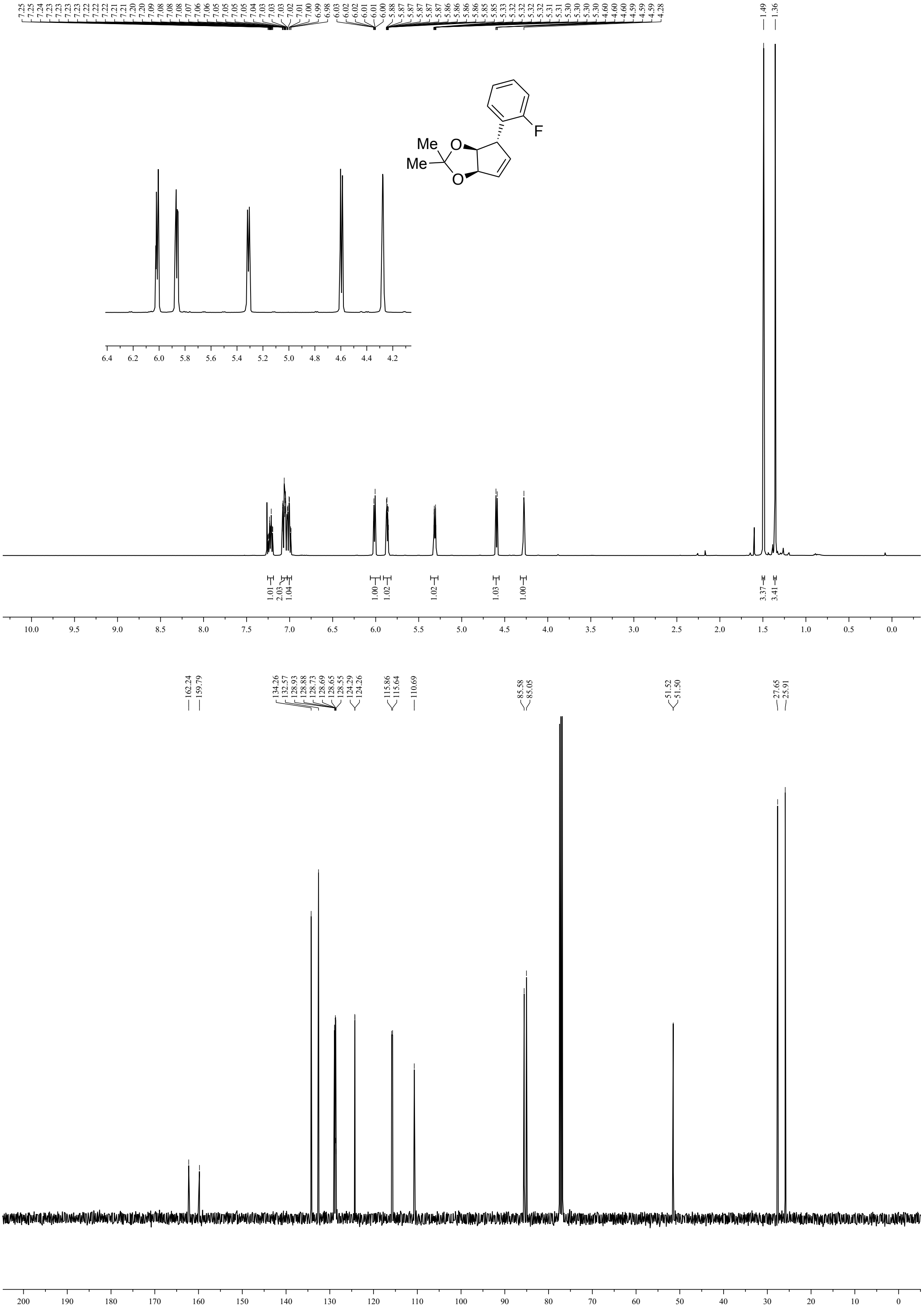

Figure S18: ^{1}H-NMR (top) and ^{13}C-NMR (bottom) of (–)-**3ad**.

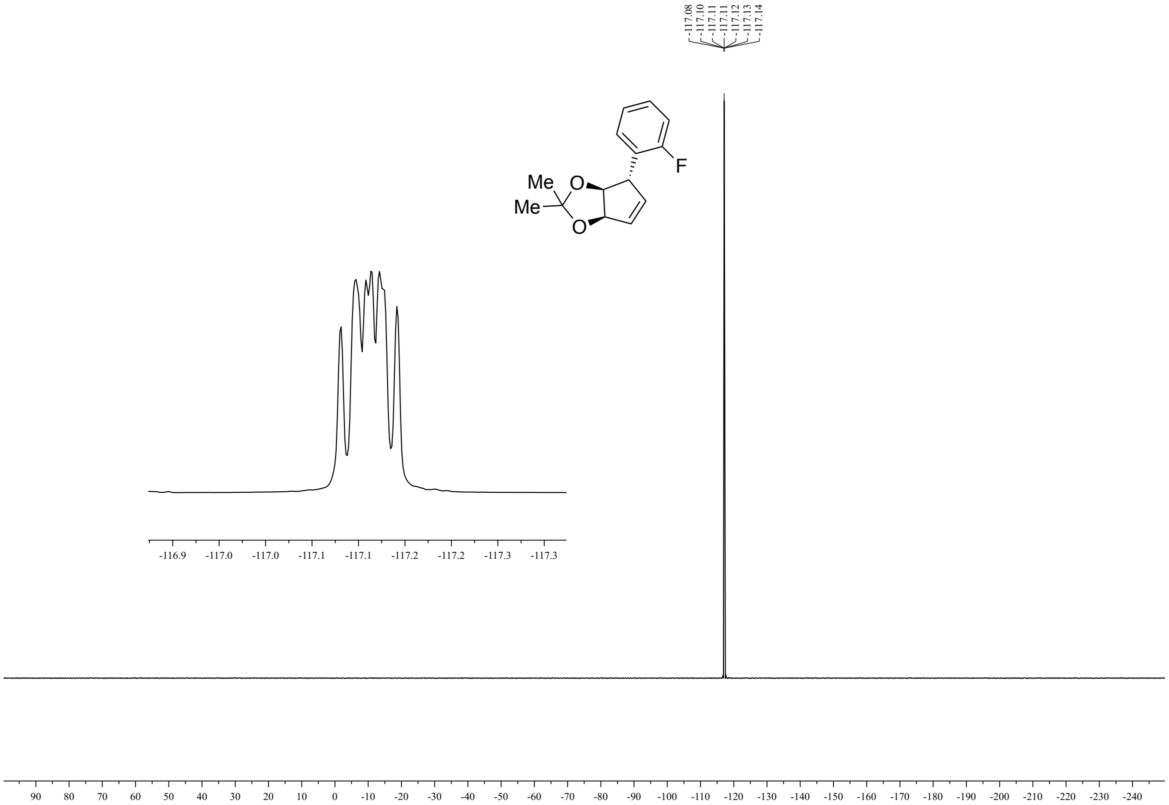

Figure S19: ^{19}F-NMR of (–)-**3ad**.

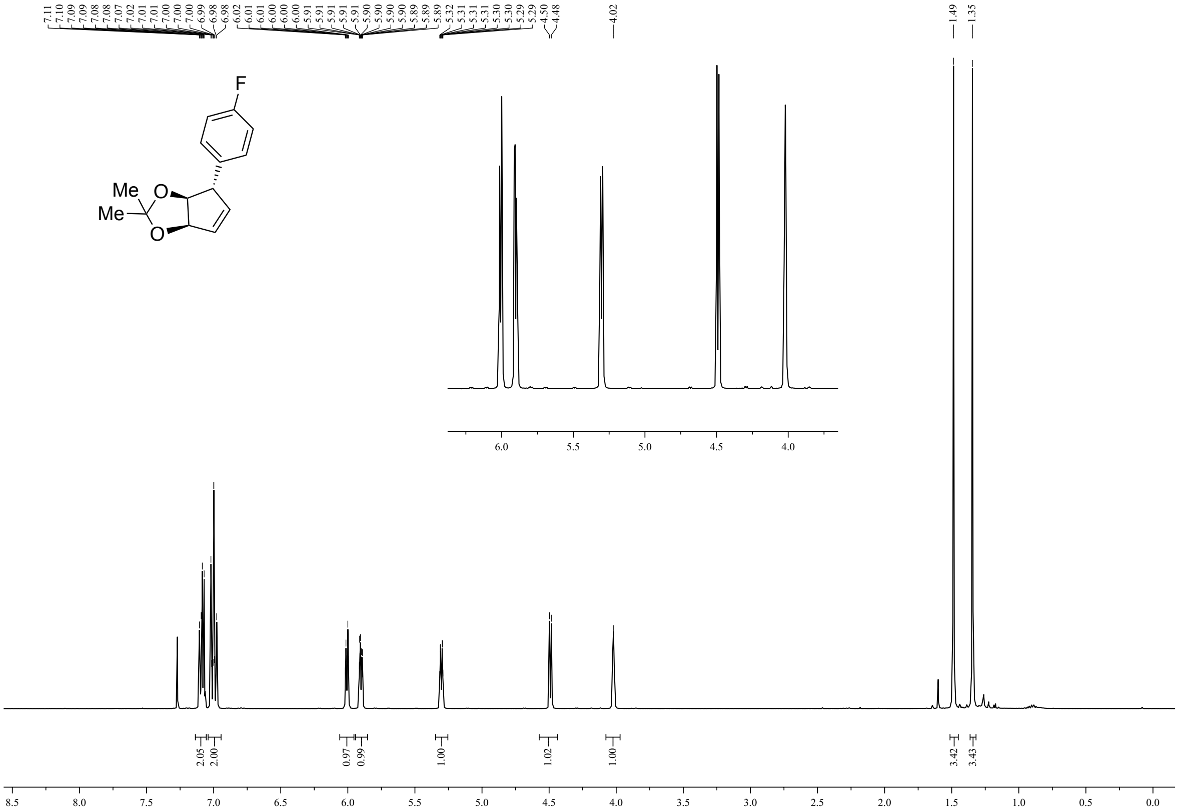

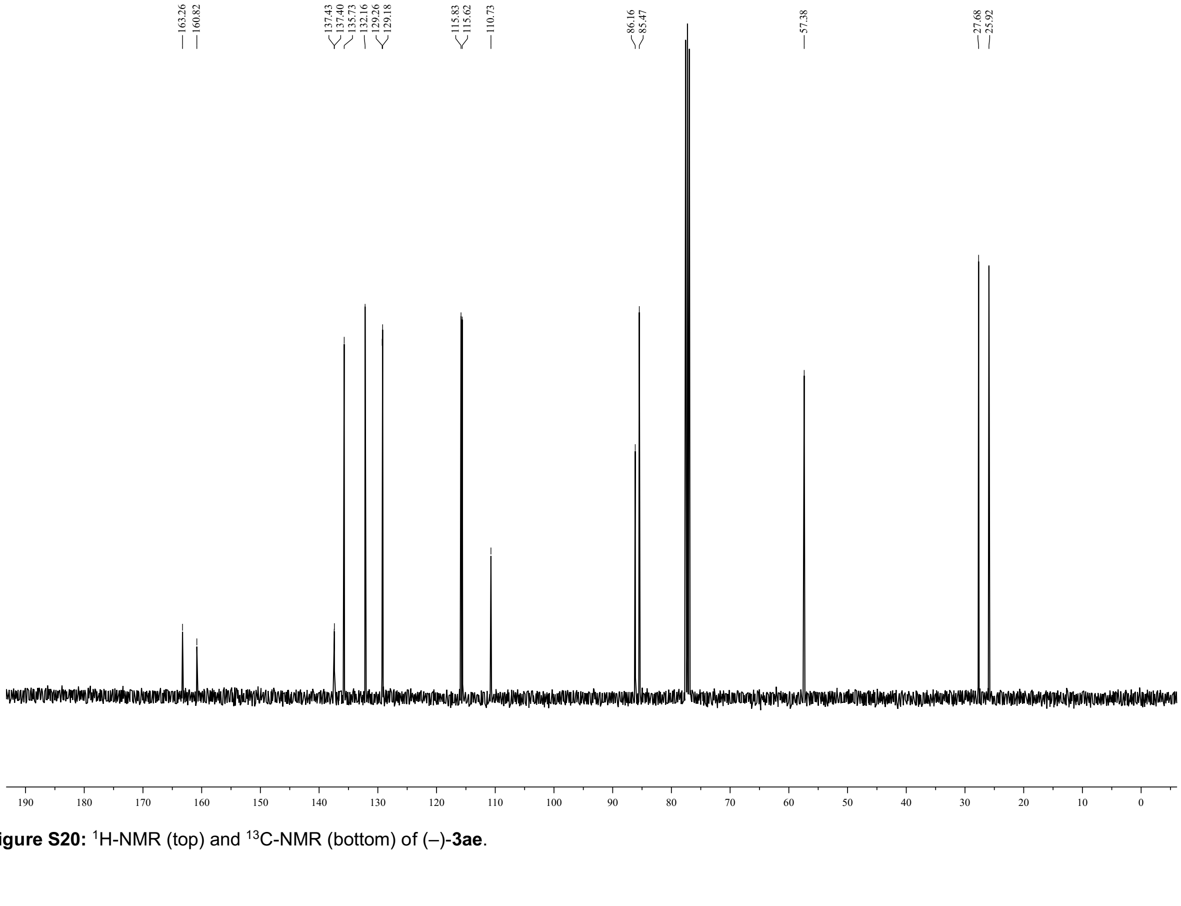

Figure S20: ^{1}H-NMR (top) and ^{13}C-NMR (bottom) of (–)-**3ae**.

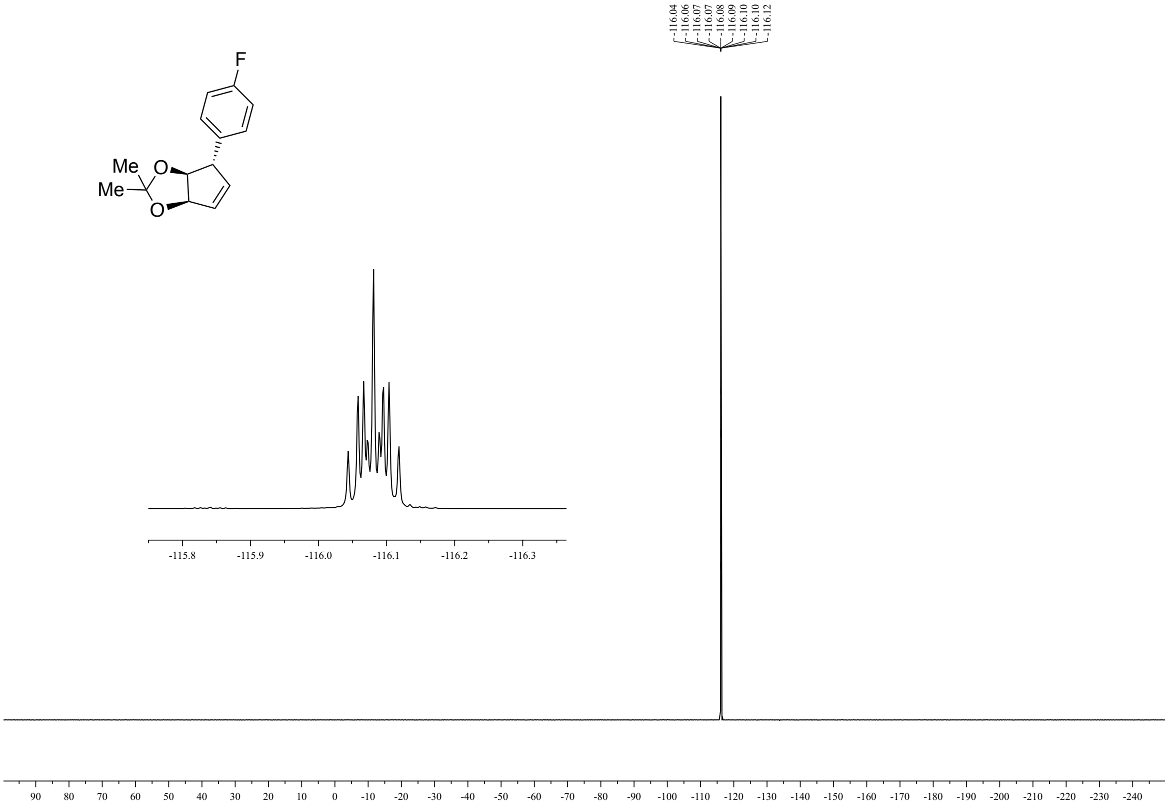

Figure S21: ^{19}F-NMR of (–)-**3ae**.

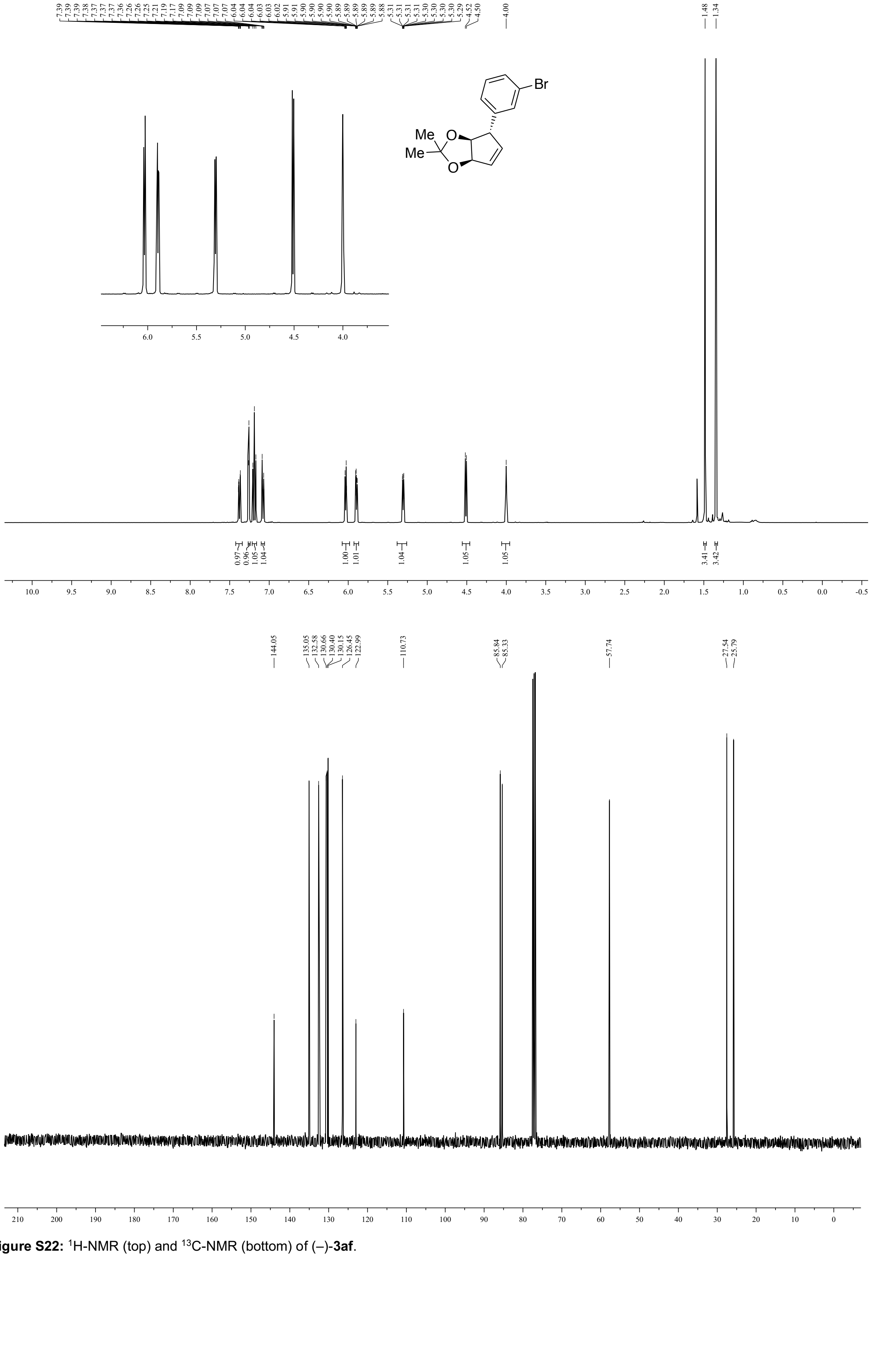

Figure S22: ^{1}H-NMR (top) and ^{13}C-NMR (bottom) of (–)-**3af**.

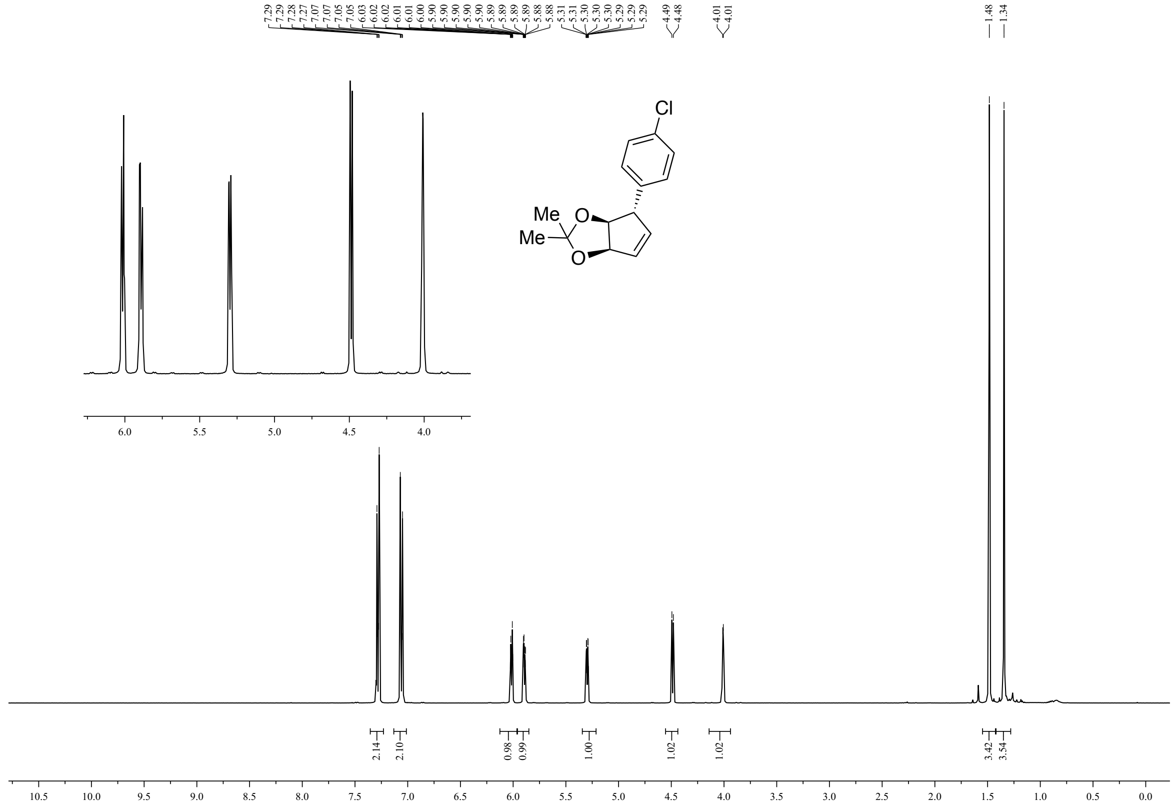

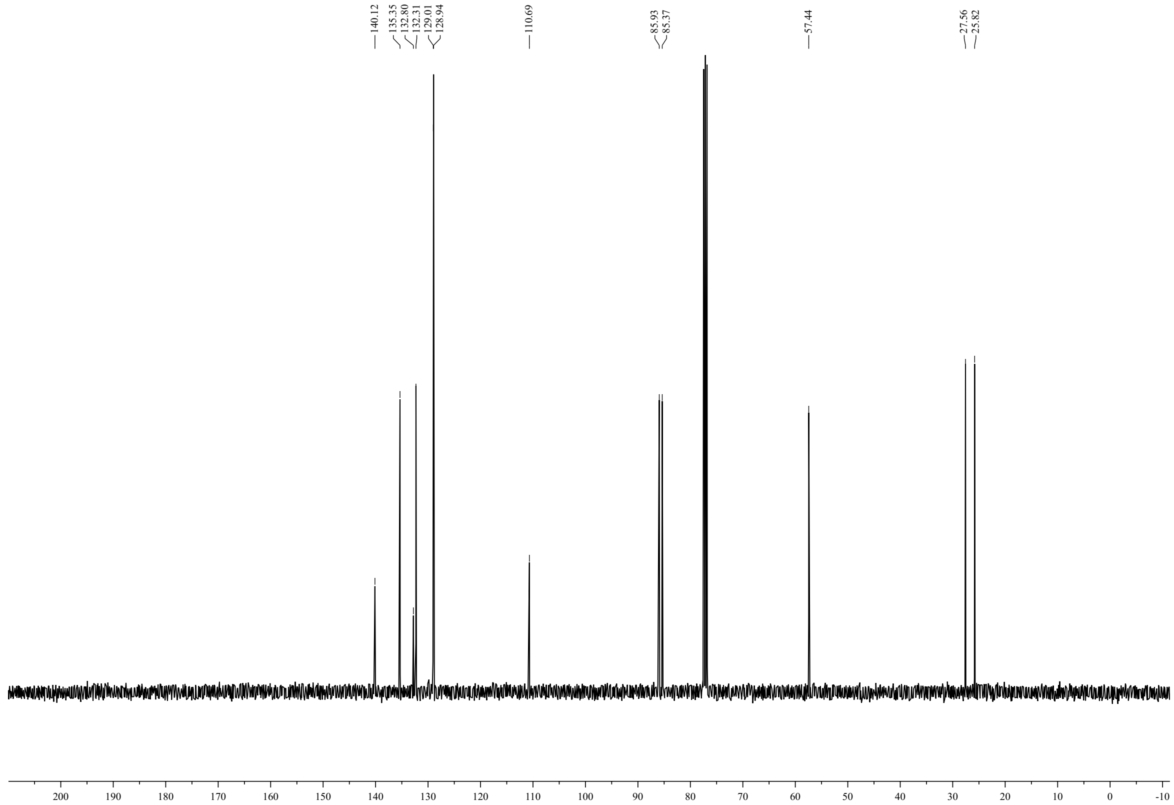

Figure S23: ^{1}H-NMR (top) and ^{13}C-NMR (bottom) of (–)-**3ag**.

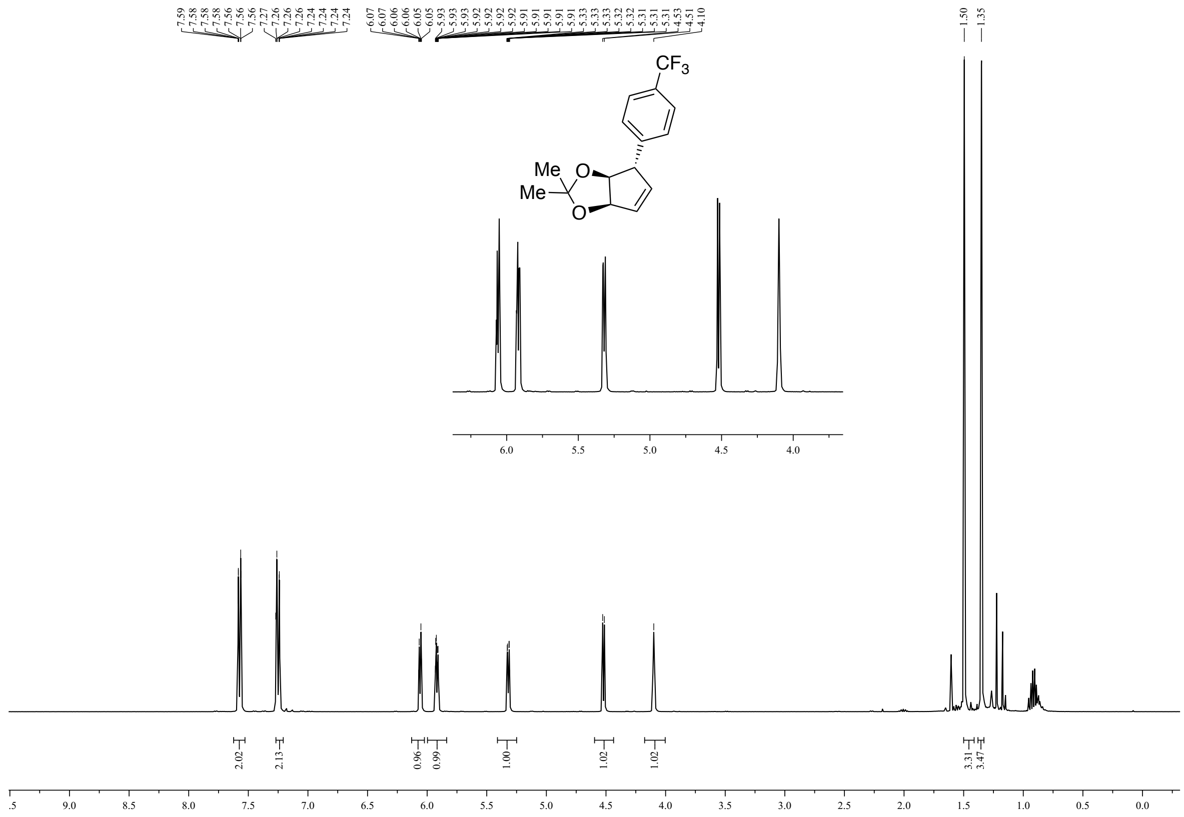

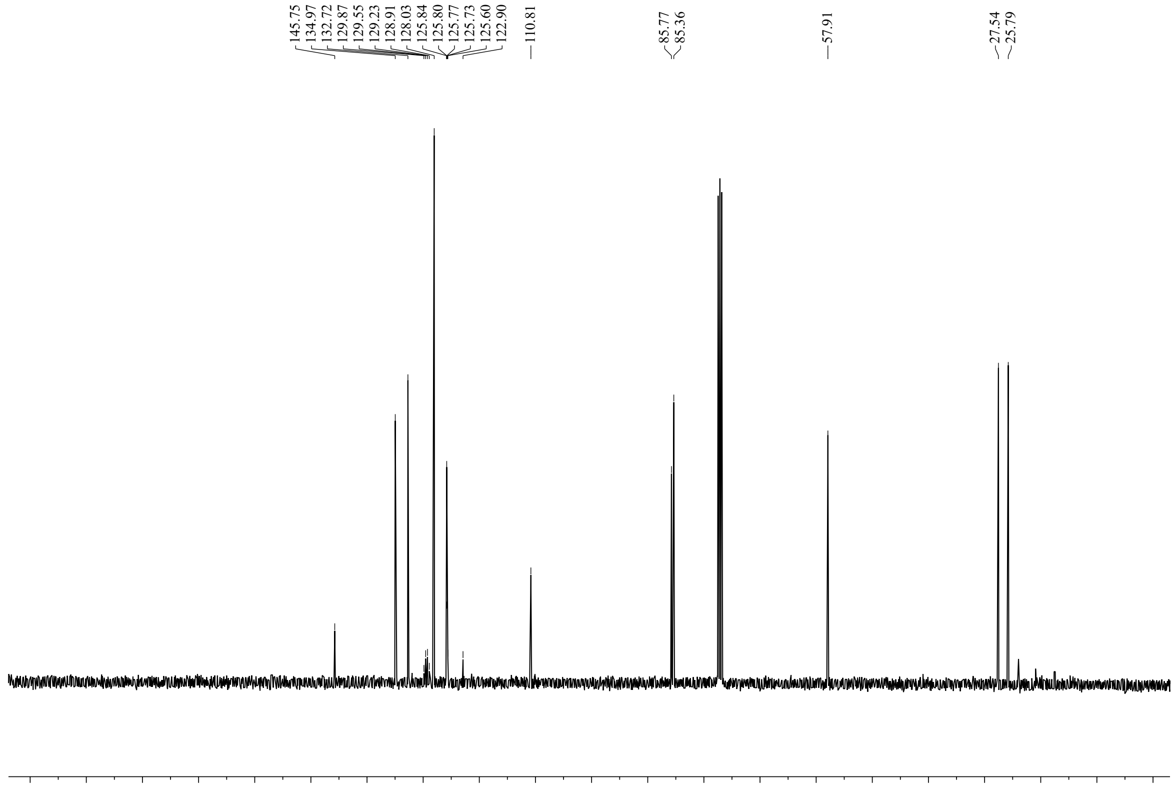

Figure S24: ^{1}H-NMR (top) and ^{13}C-NMR (bottom) of (–)-**3ah**.

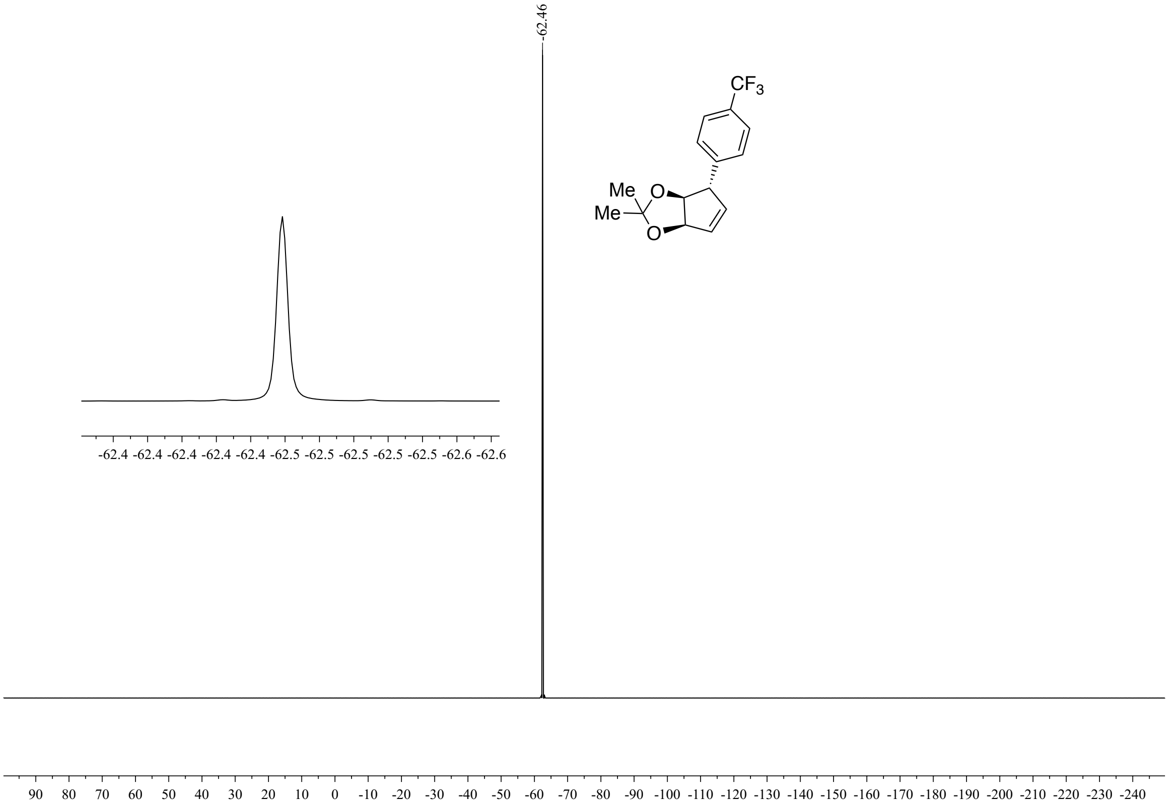

Figure S25: ^{19}F-NMR of (–)-**3ah**.

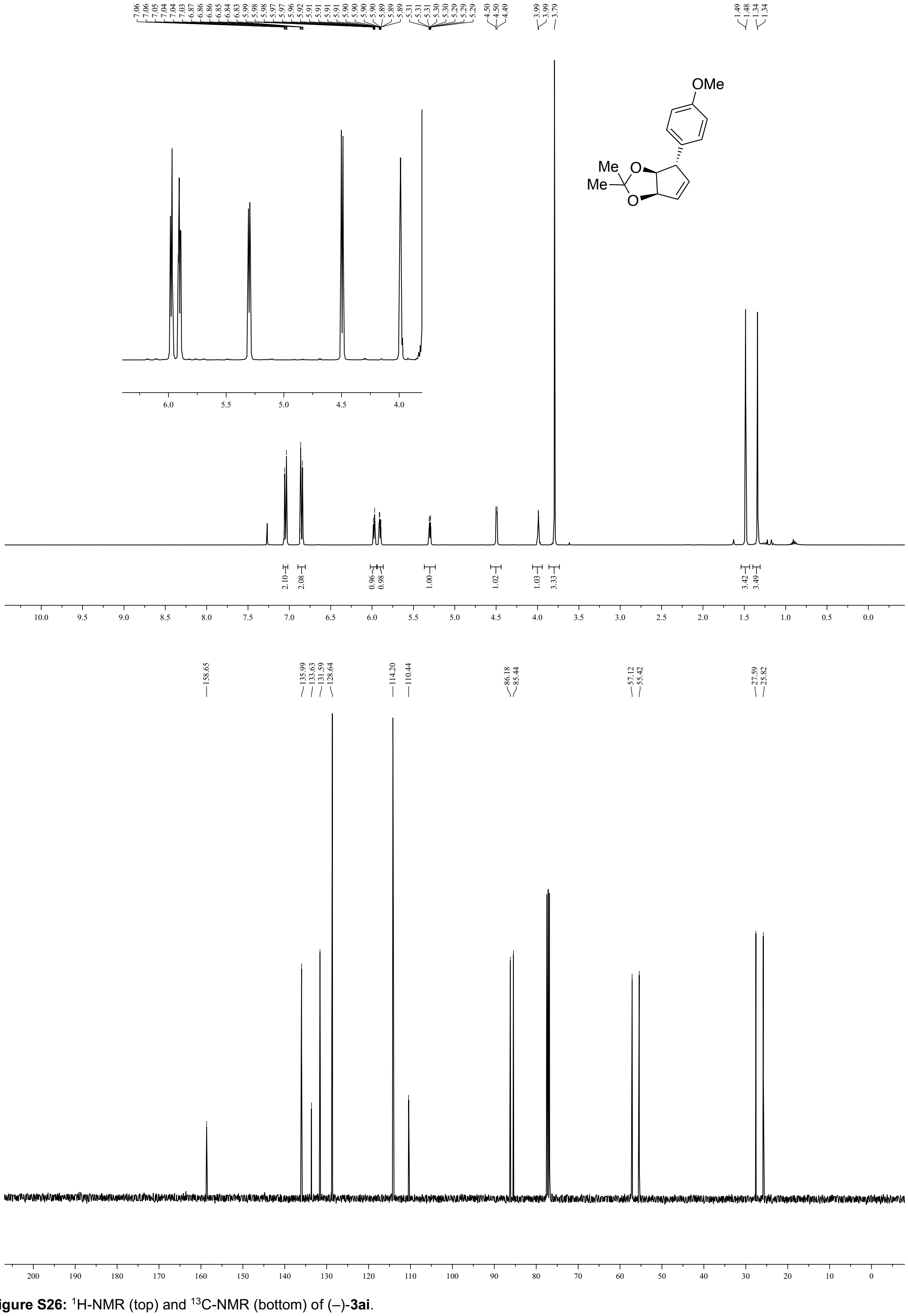

Figure S26: ^{1}H-NMR (top) and ^{13}C-NMR (bottom) of (–)-**3ai**.

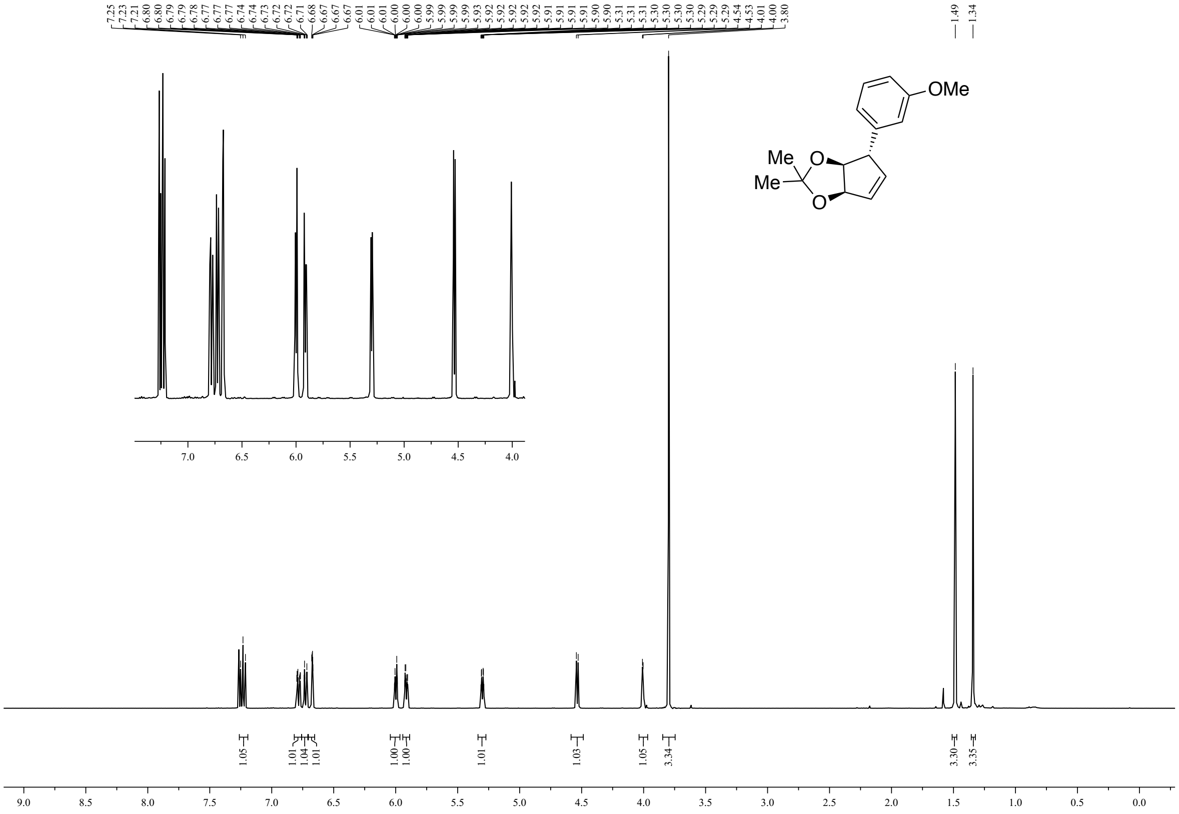

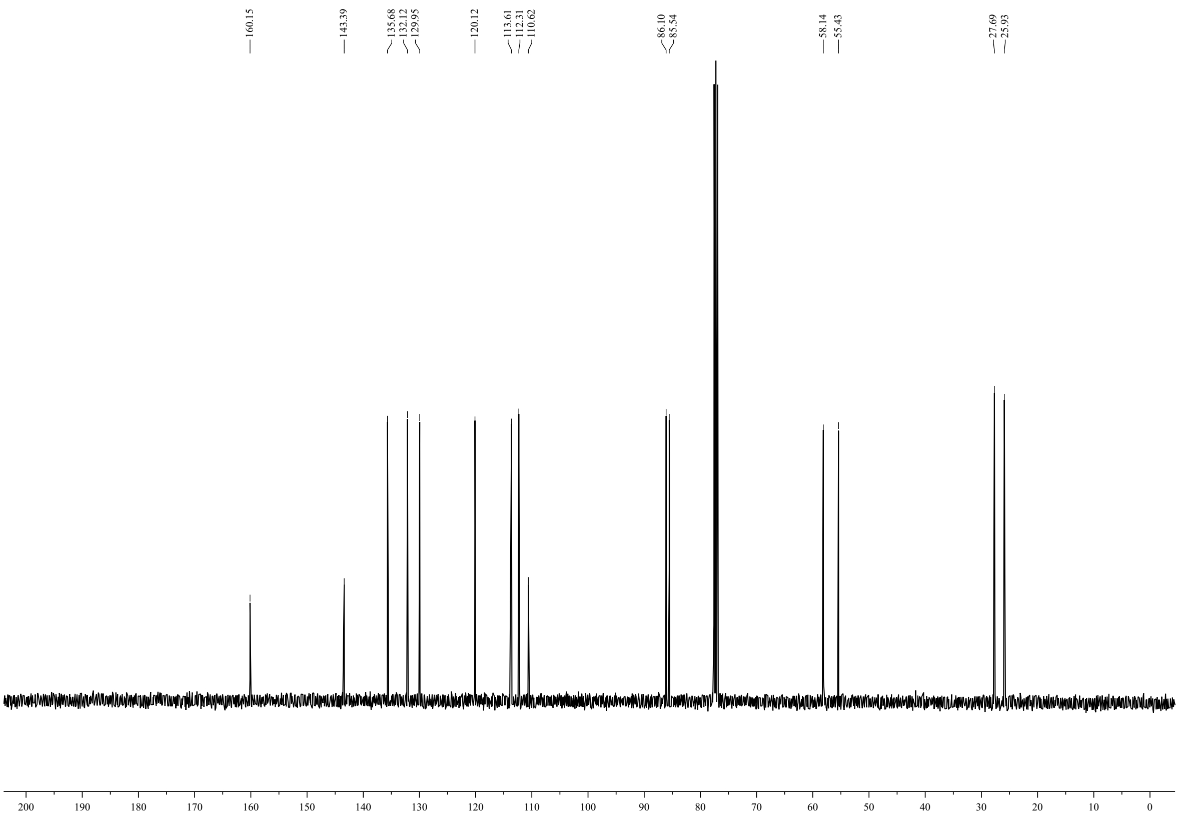

Figure S27: ^{1}H-NMR (top) and ^{13}C-NMR (bottom) of (–)-**3aj**.

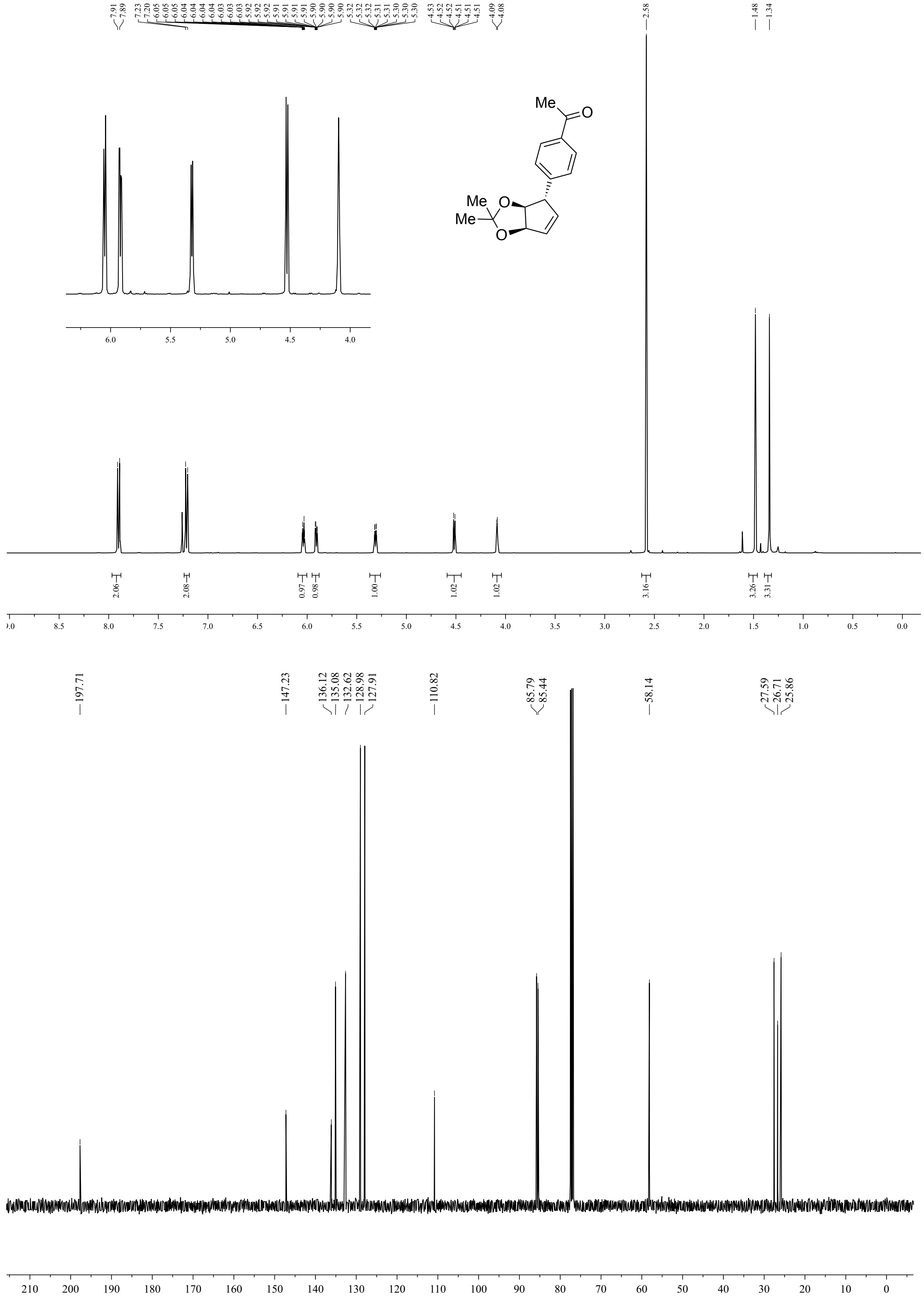

Figure S28: ^{1}H-NMR (top) and ^{13}C-NMR (bottom) of (–)-**3ak**.

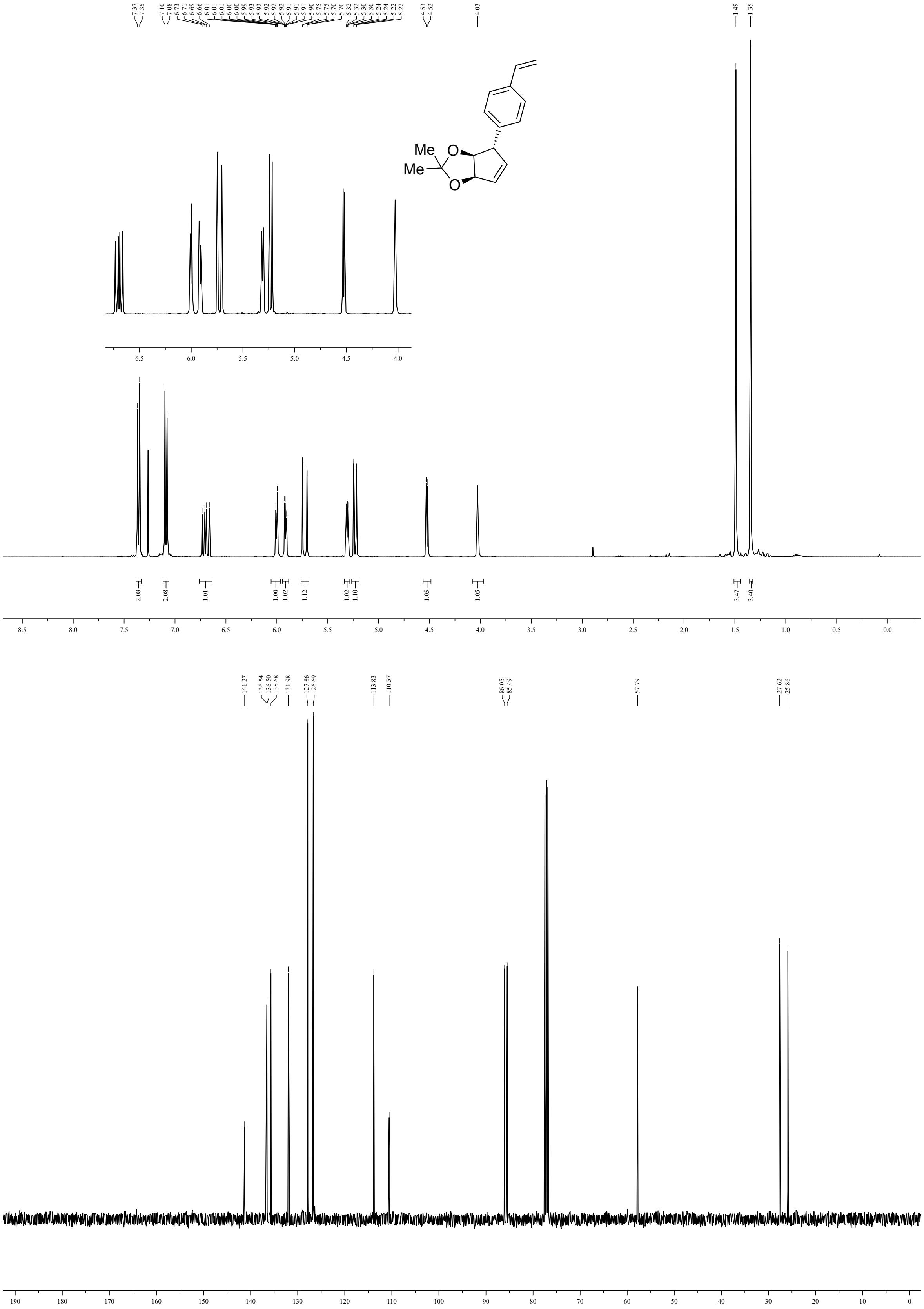

Figure S29: ^{1}H-NMR (top) and ^{13}C-NMR (bottom) of (–)-**3al**.

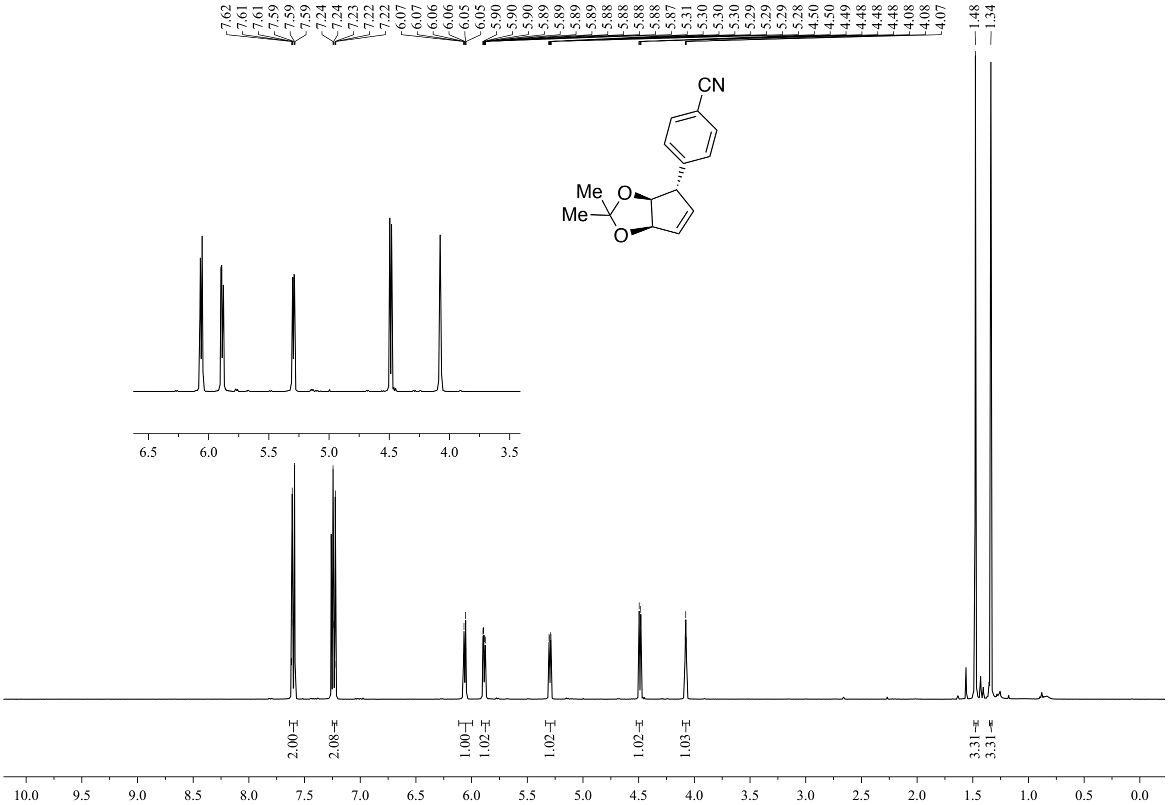

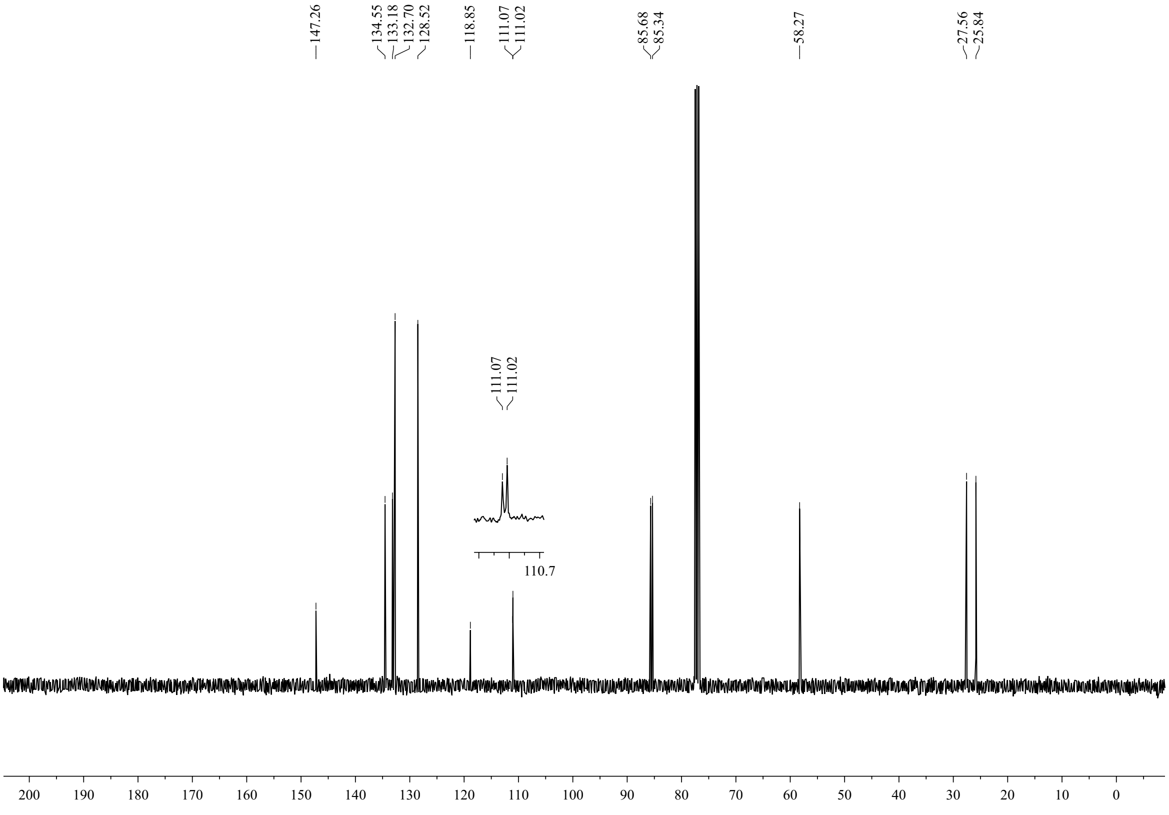

Figure S30: ^{1}H-NMR (top) and ^{13}C-NMR (bottom) of (–)-**3am**.

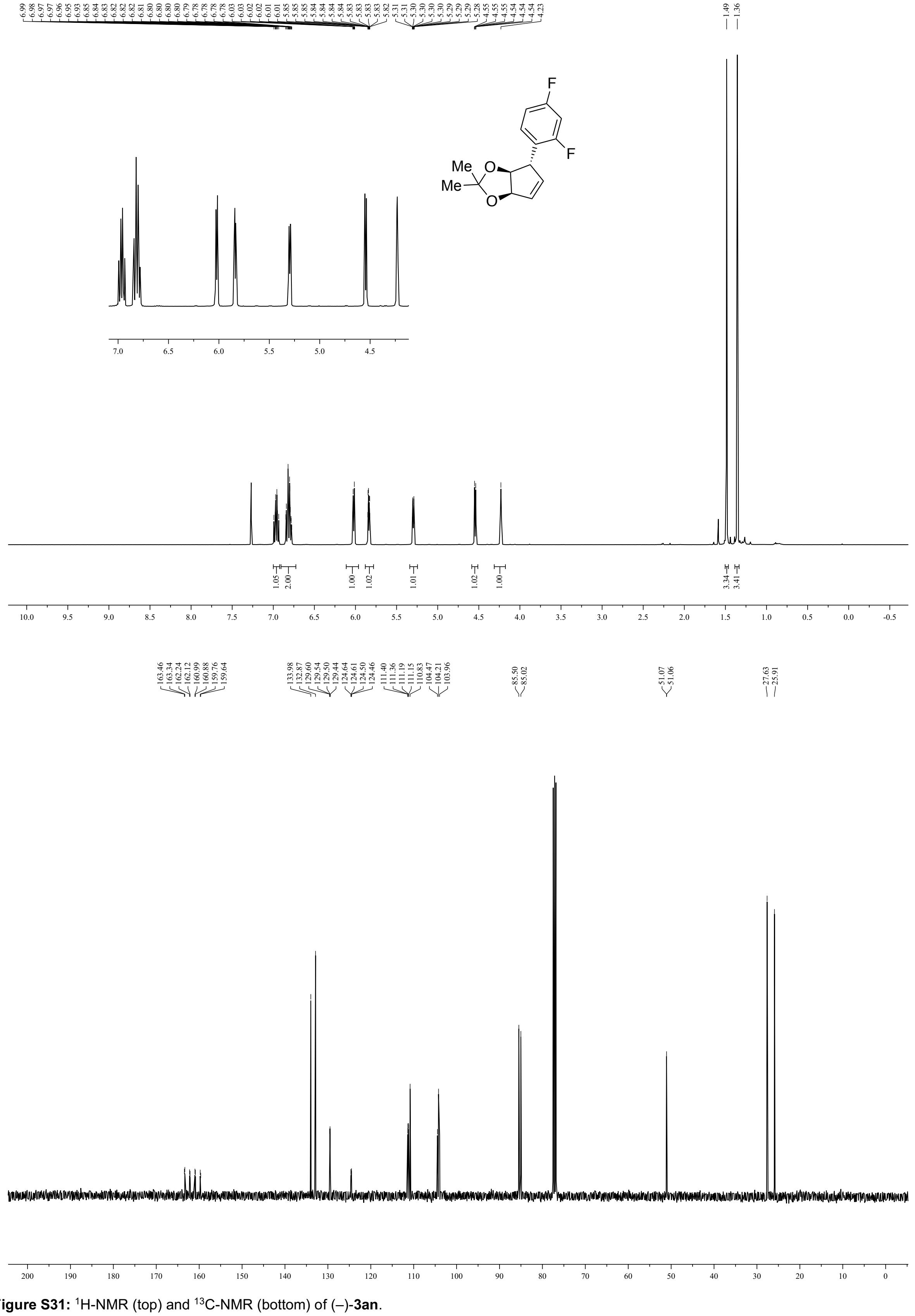

Figure S31: ^{1}H-NMR (top) and ^{13}C-NMR (bottom) of (–)-**3an**.

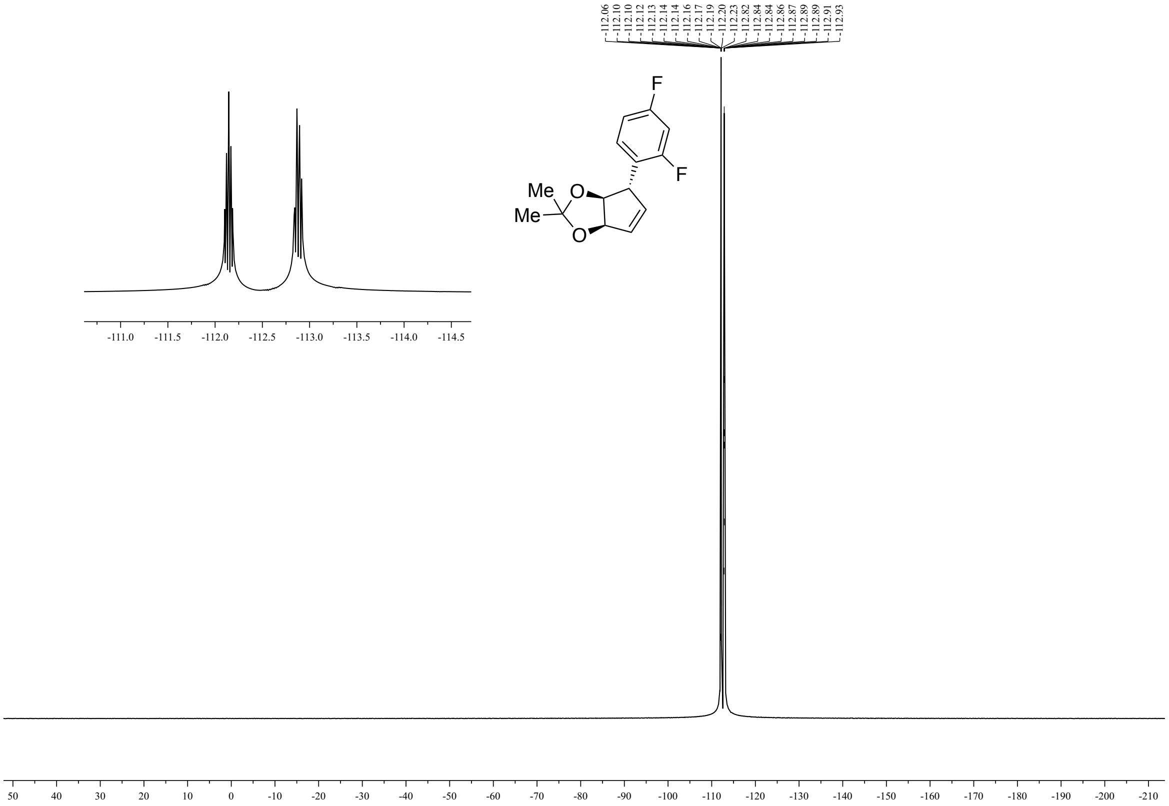

Figure S32: ^{19}F-NMR of (–)-**3an**.

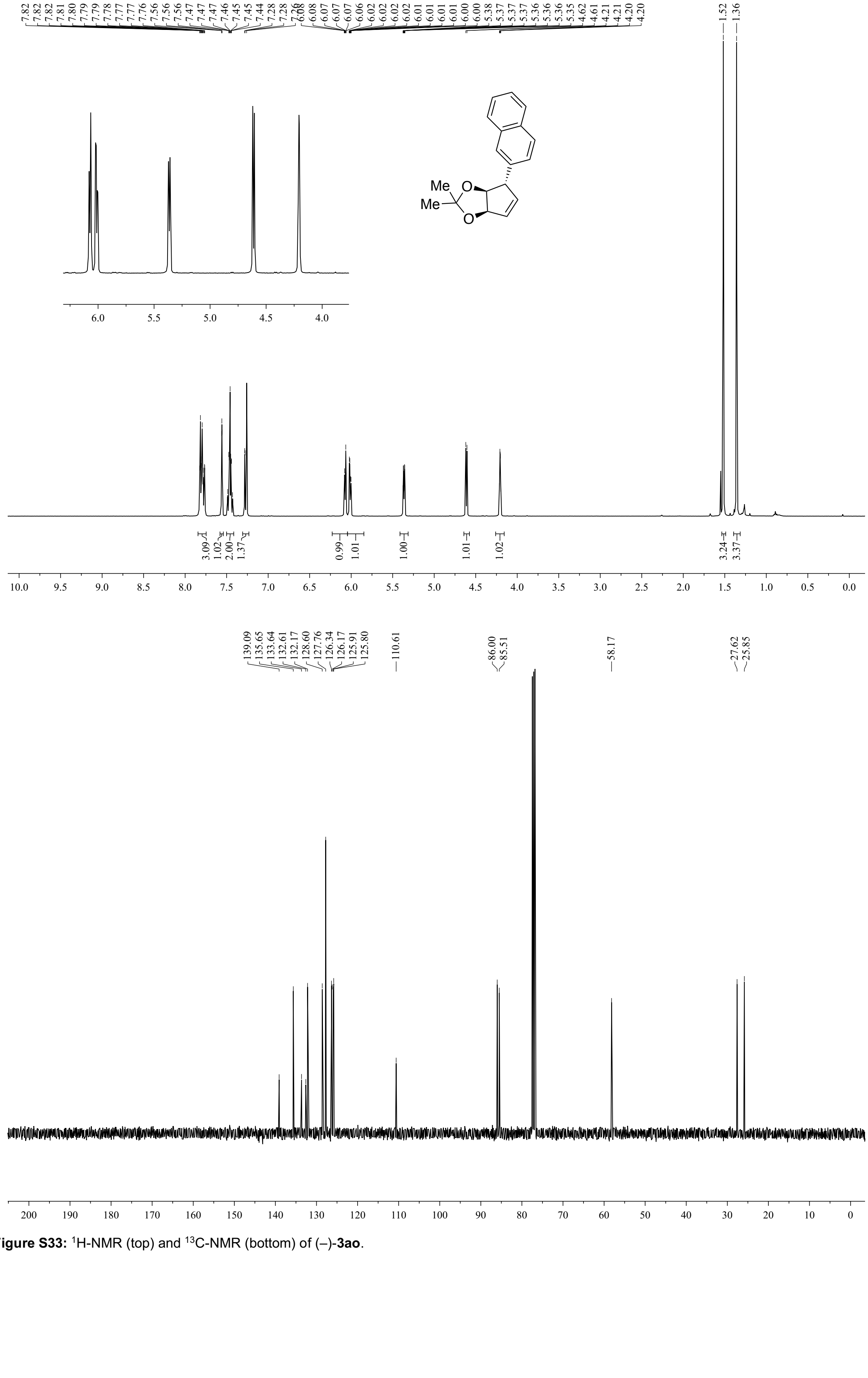

Figure S33: ^{1}H-NMR (top) and ^{13}C-NMR (bottom) of (–)-**3ao**.

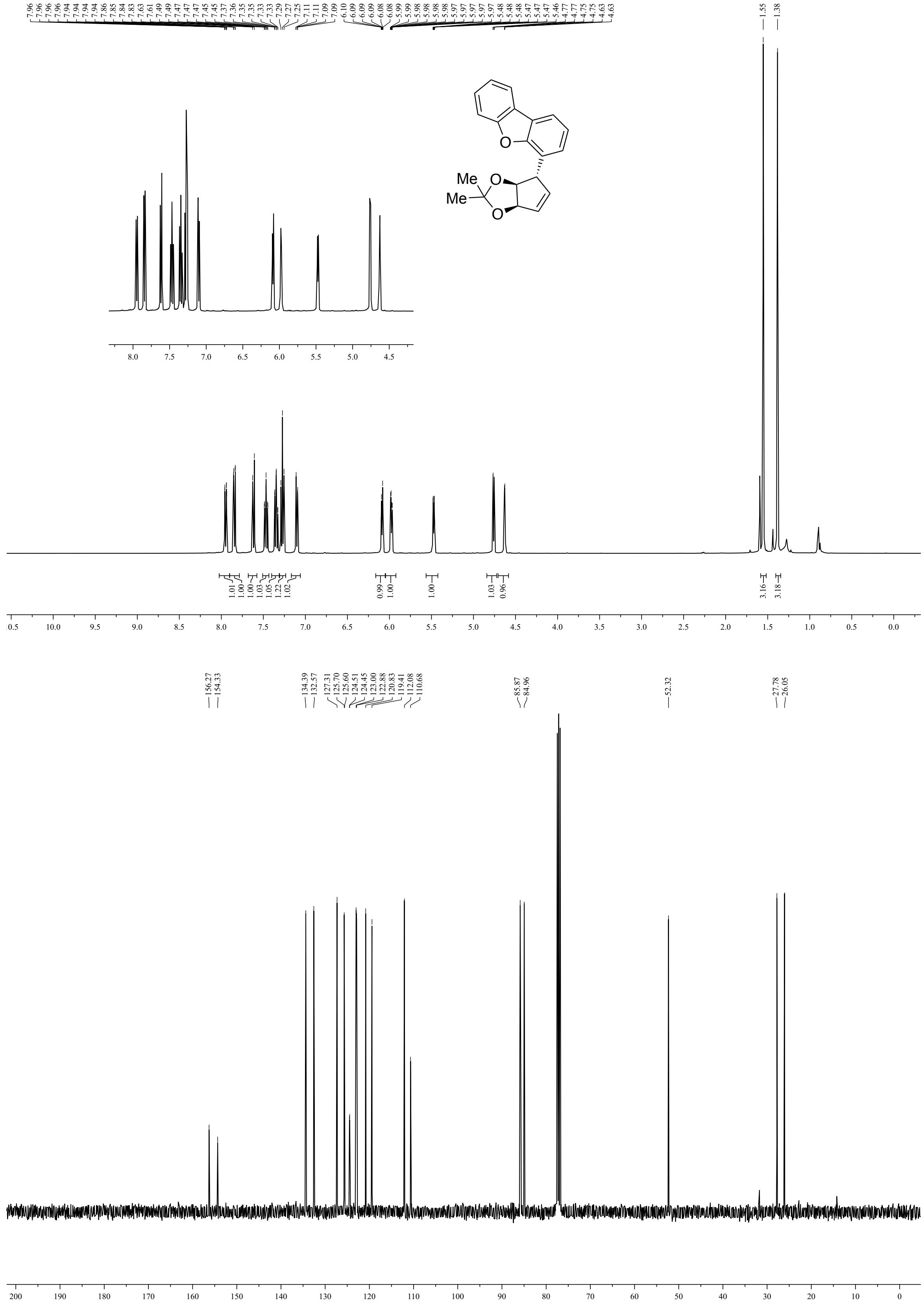

Figure S34: ^{1}H-NMR (top) and ^{13}C-NMR (bottom) of (–)-**3ap**.

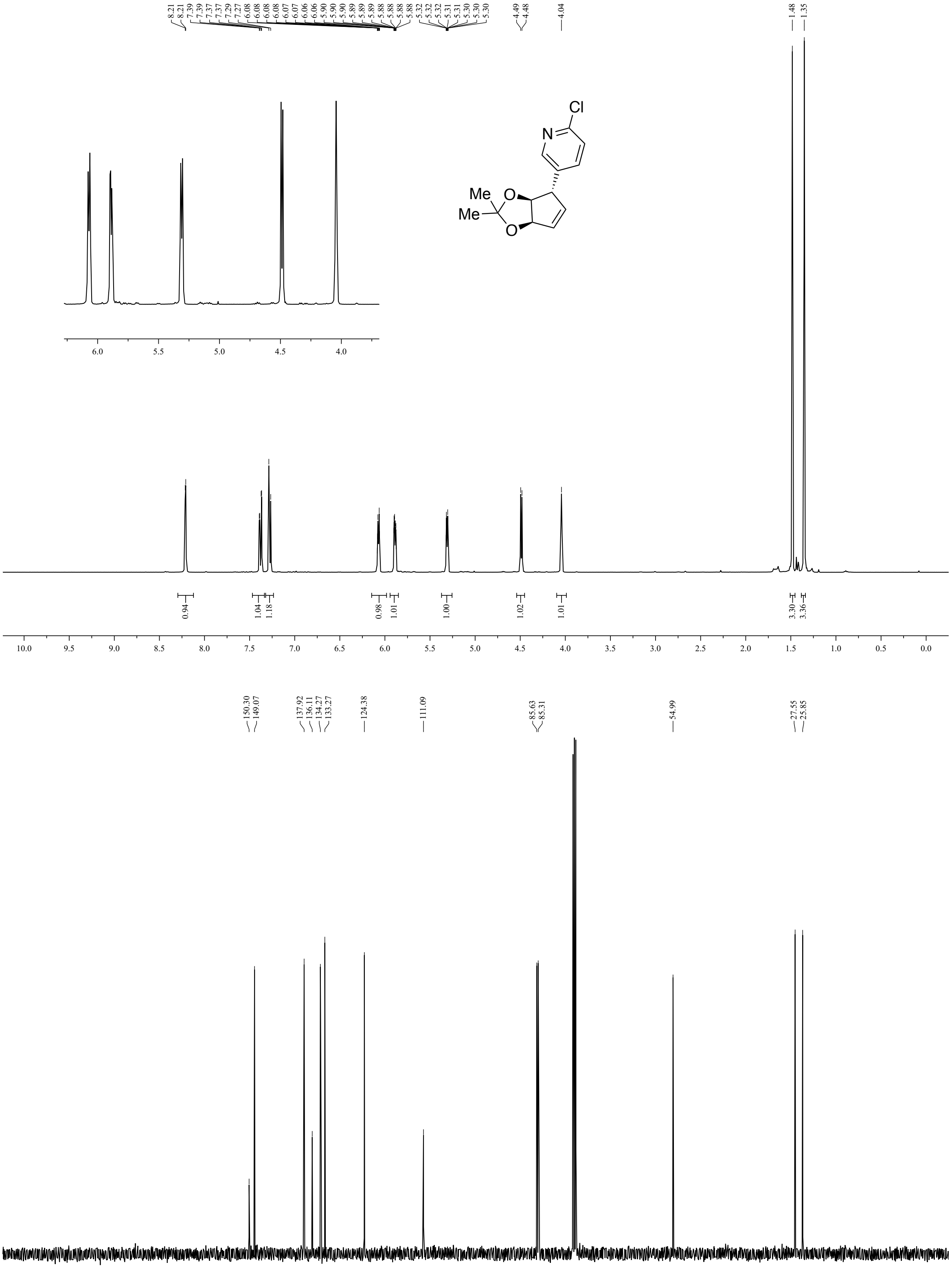

Figure S35: ^{1}H-NMR (top) and ^{13}C-NMR (bottom) of (–)-**3aq**.

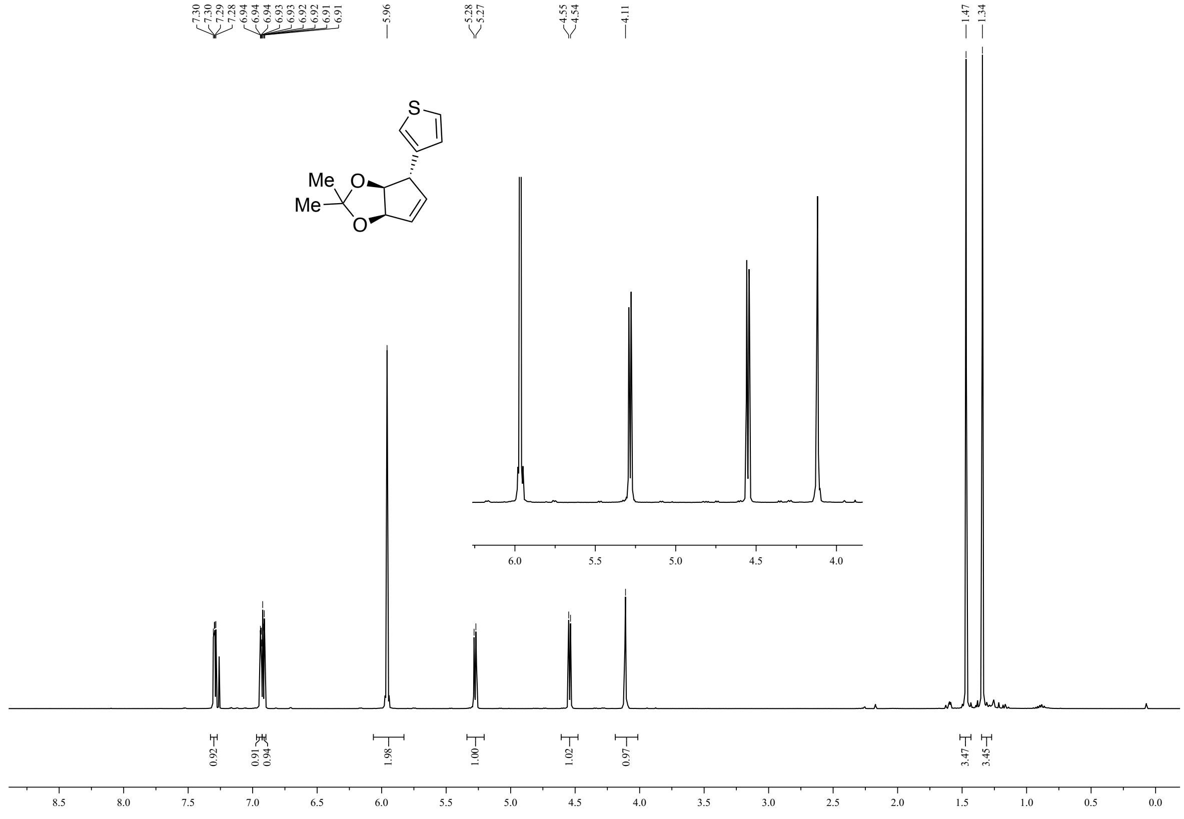

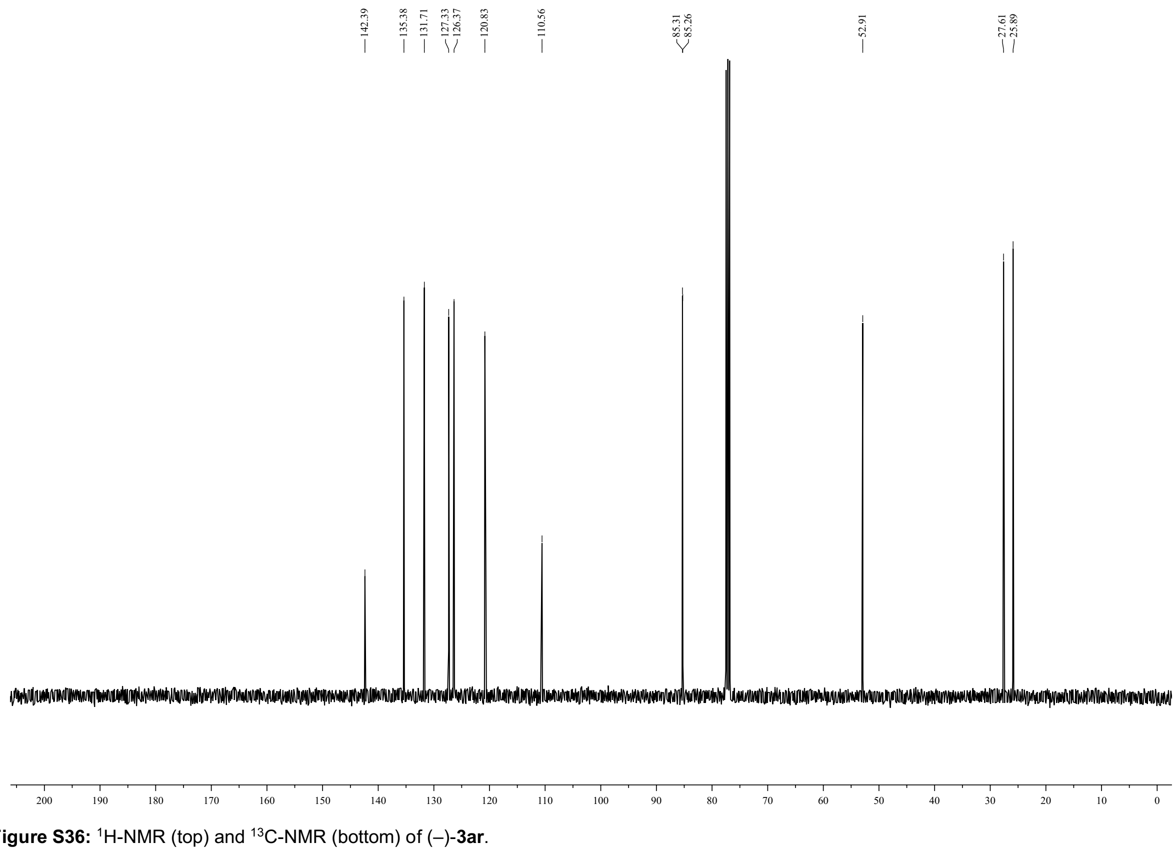

Figure S36: ^{1}H-NMR (top) and ^{13}C-NMR (bottom) of (–)-**3ar**.

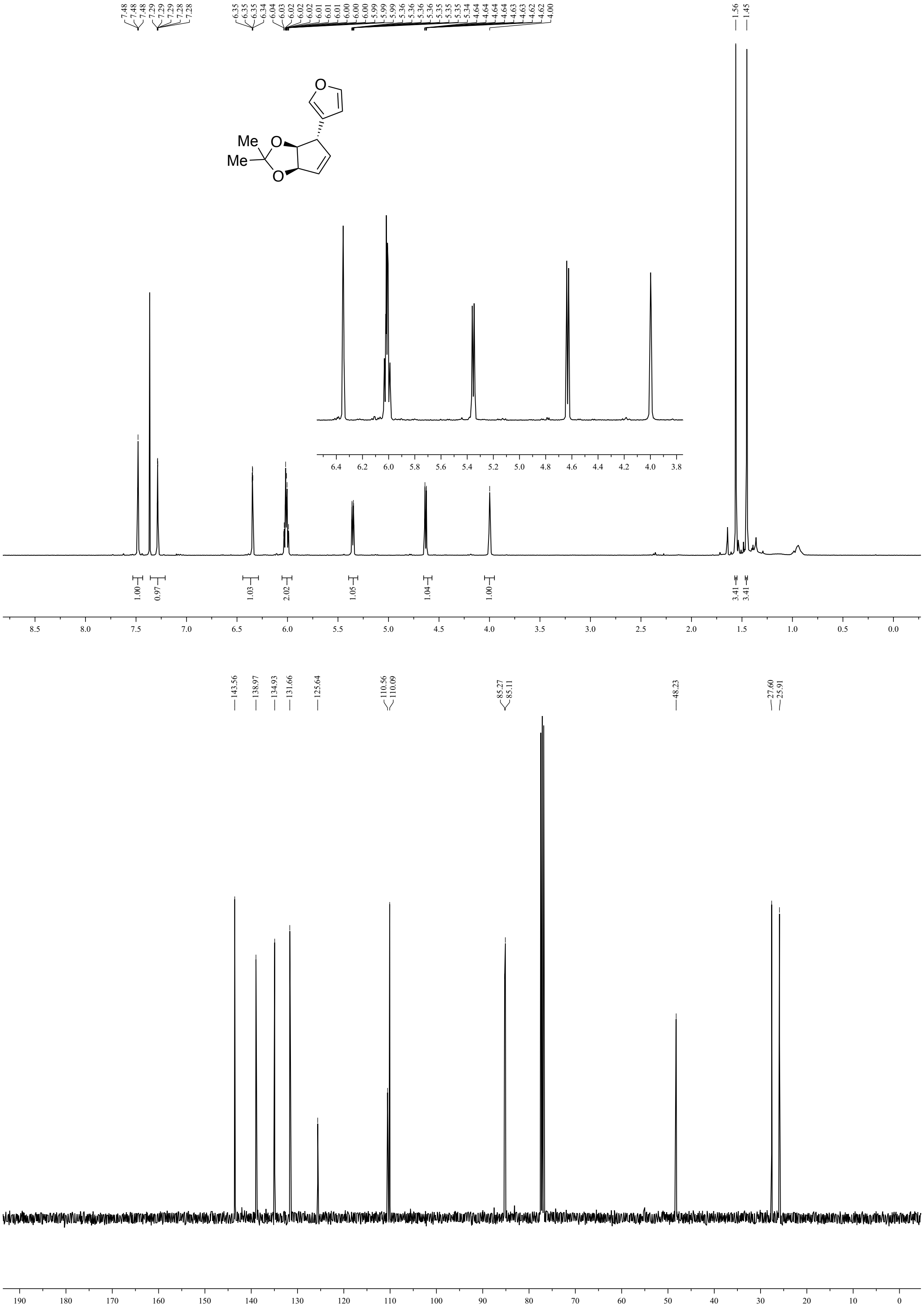

Figure S37: ^{1}H-NMR (top) and ^{13}C-NMR (bottom) of (–)-**3as**.

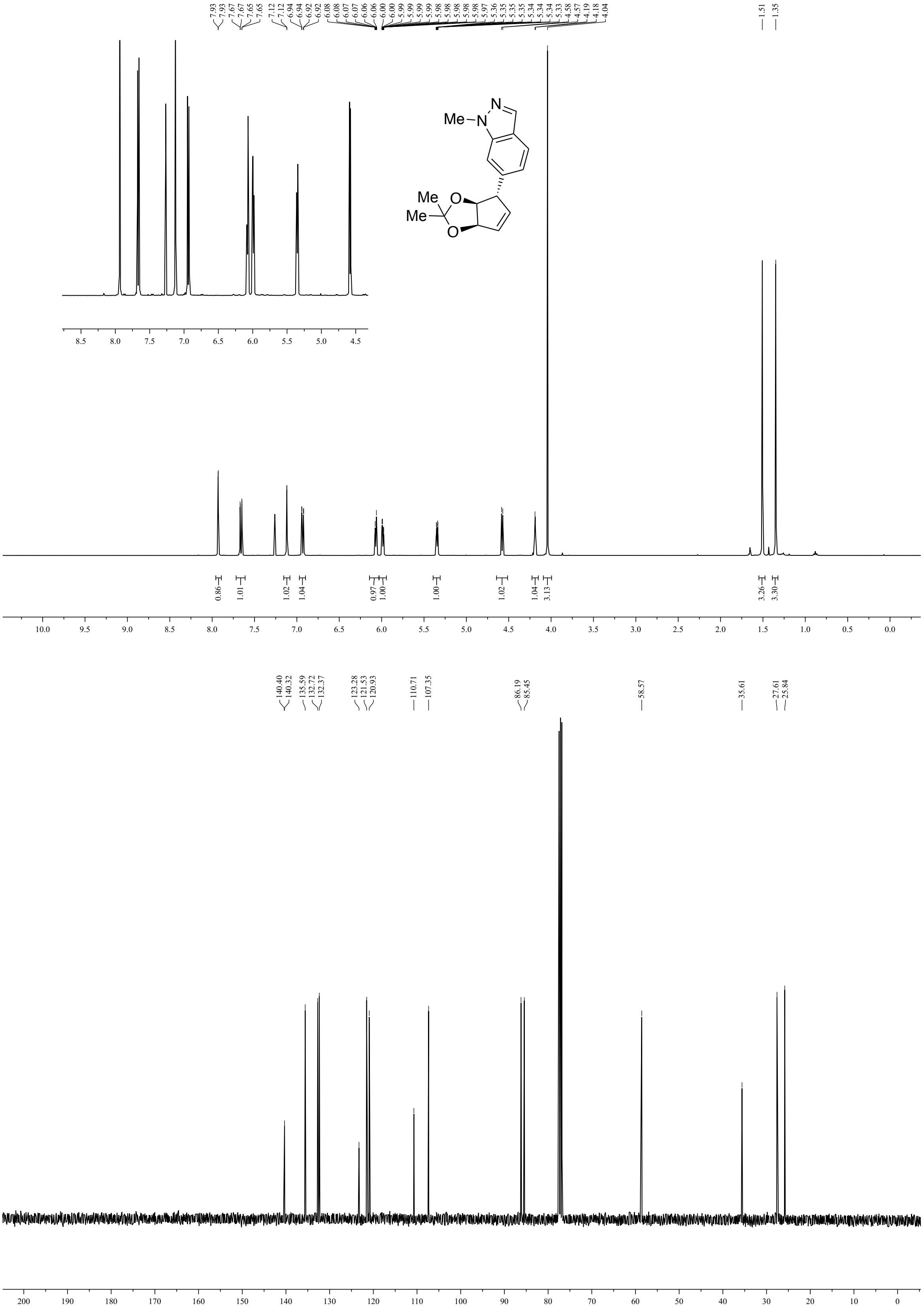

Figure S38: ^{1}H-NMR (top) and ^{13}C-NMR (bottom) of (–)-**3at**.

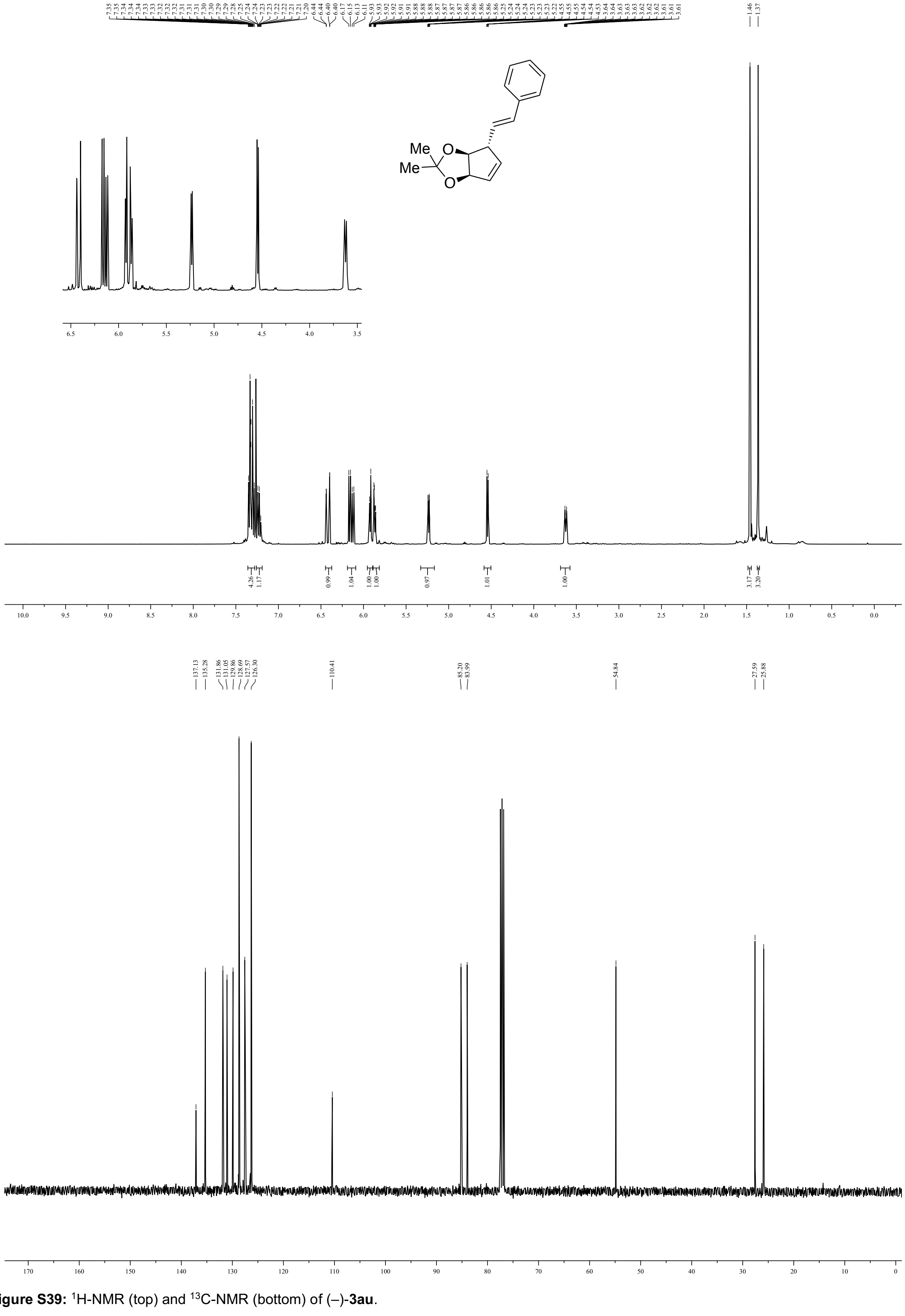

Figure S39: ^{1}H-NMR (top) and ^{13}C-NMR (bottom) of (–)-**3au**.

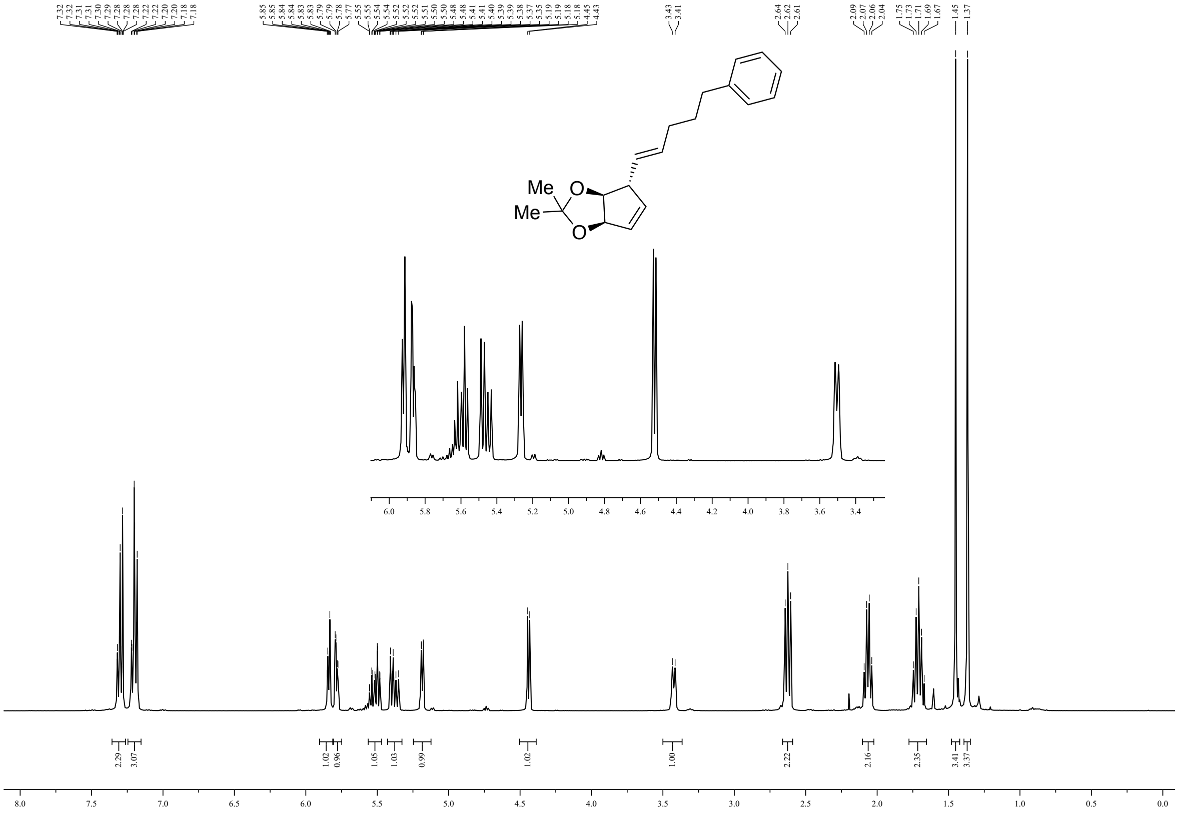

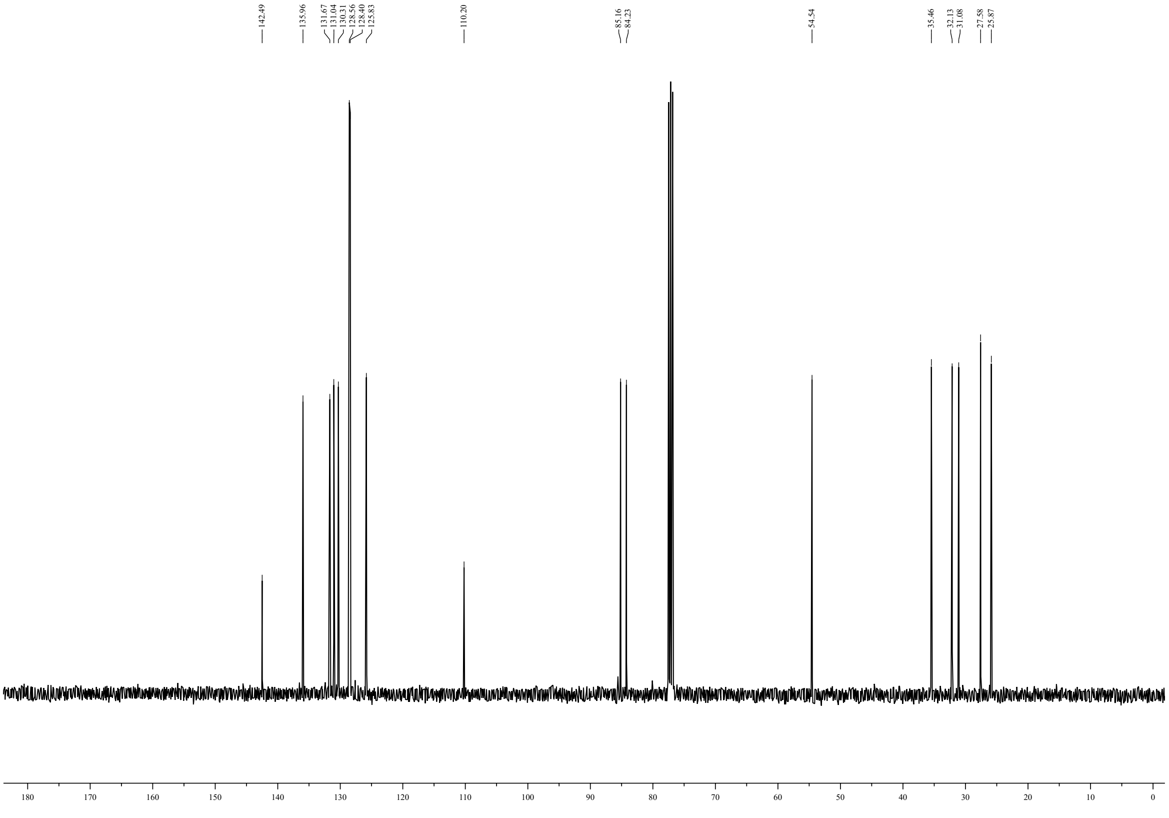

Figure S40: ^{1}H-NMR (top) and ^{13}C-NMR (bottom) of (–)-**3av**.

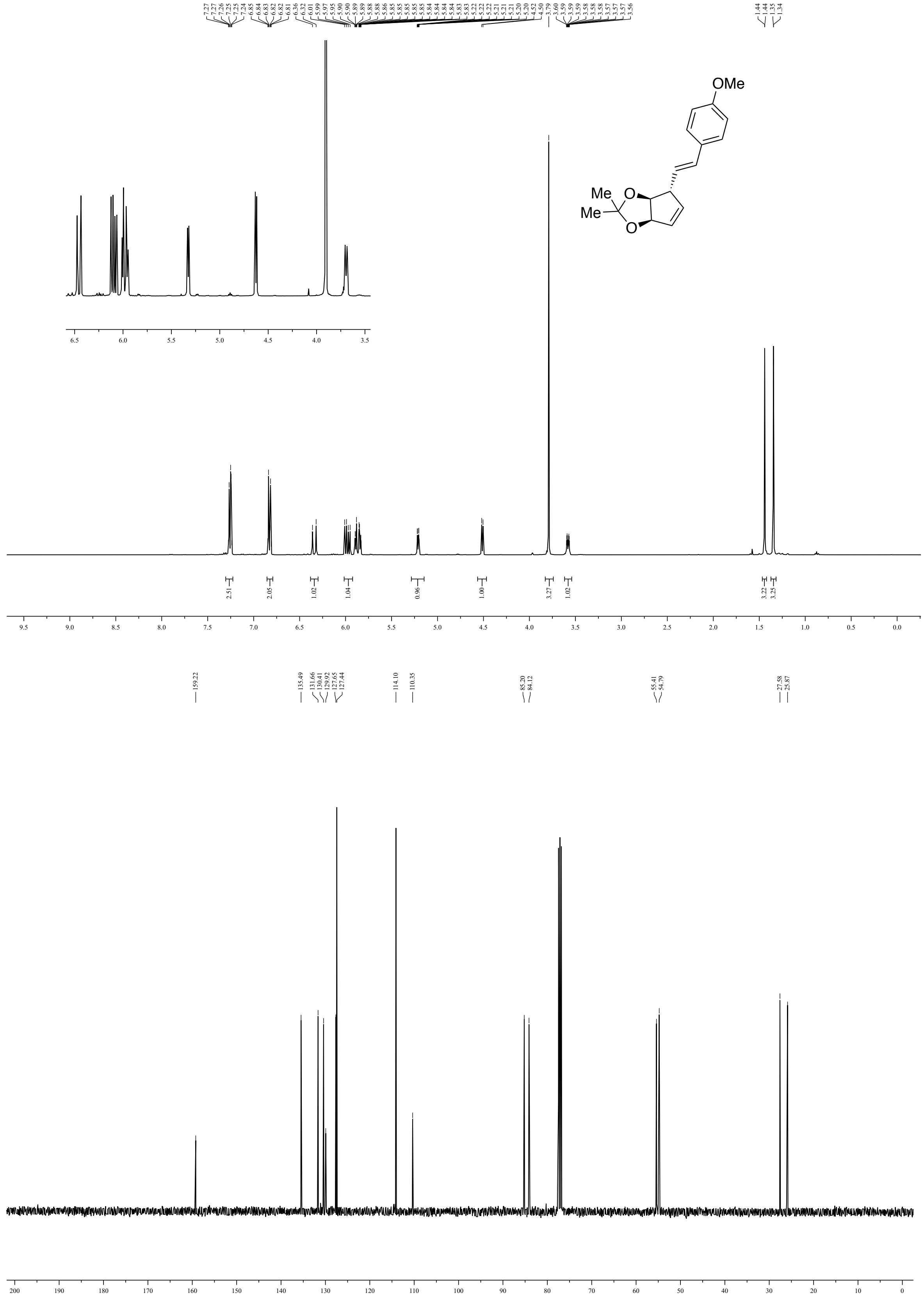

Figure S41: ^{1}H-NMR (top) and ^{13}C-NMR (bottom) of (–)-**3aw**.

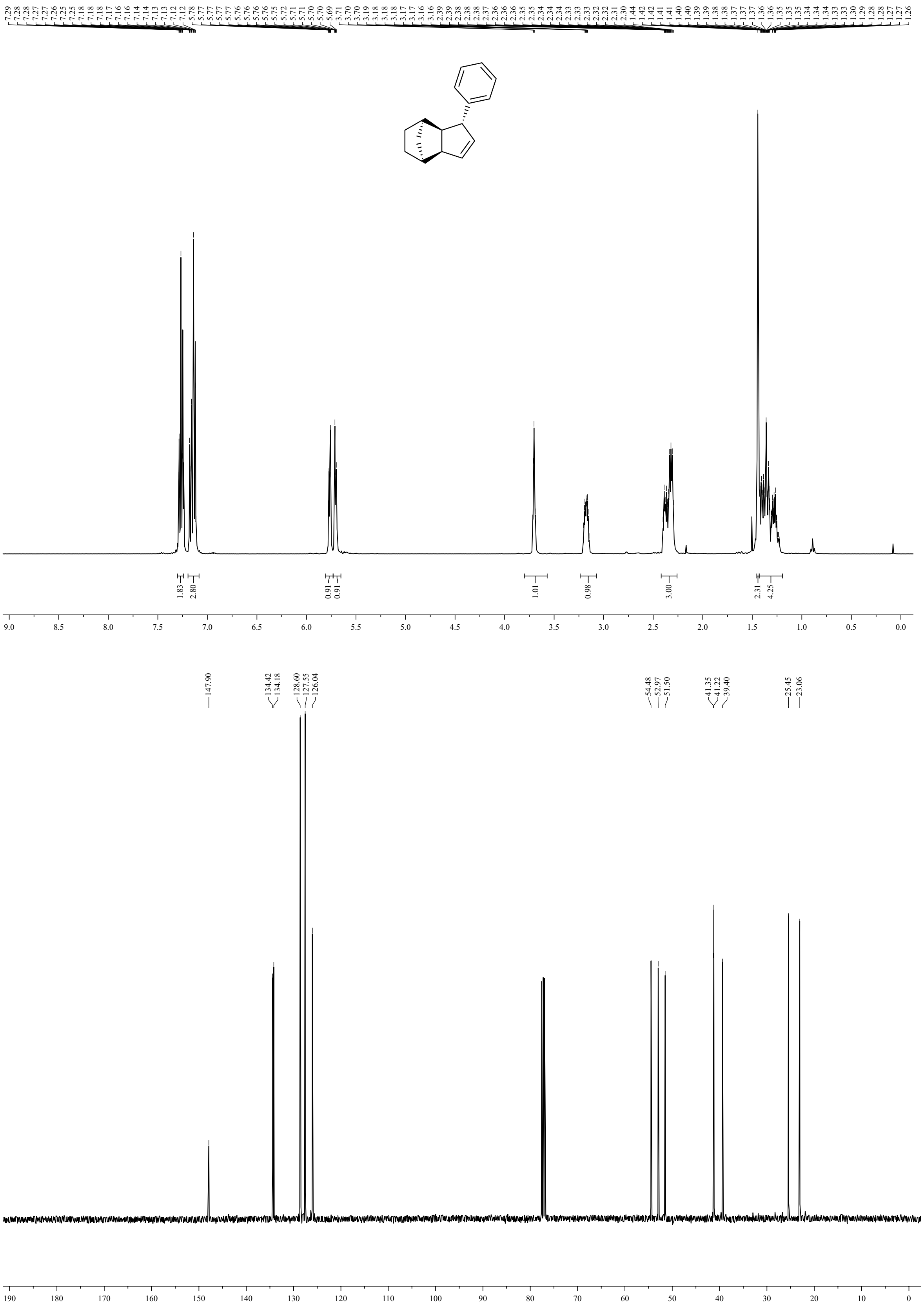

Figure S42: ^{1}H-NMR (top) and ^{13}C-NMR (bottom) of (–)-**3ba**.

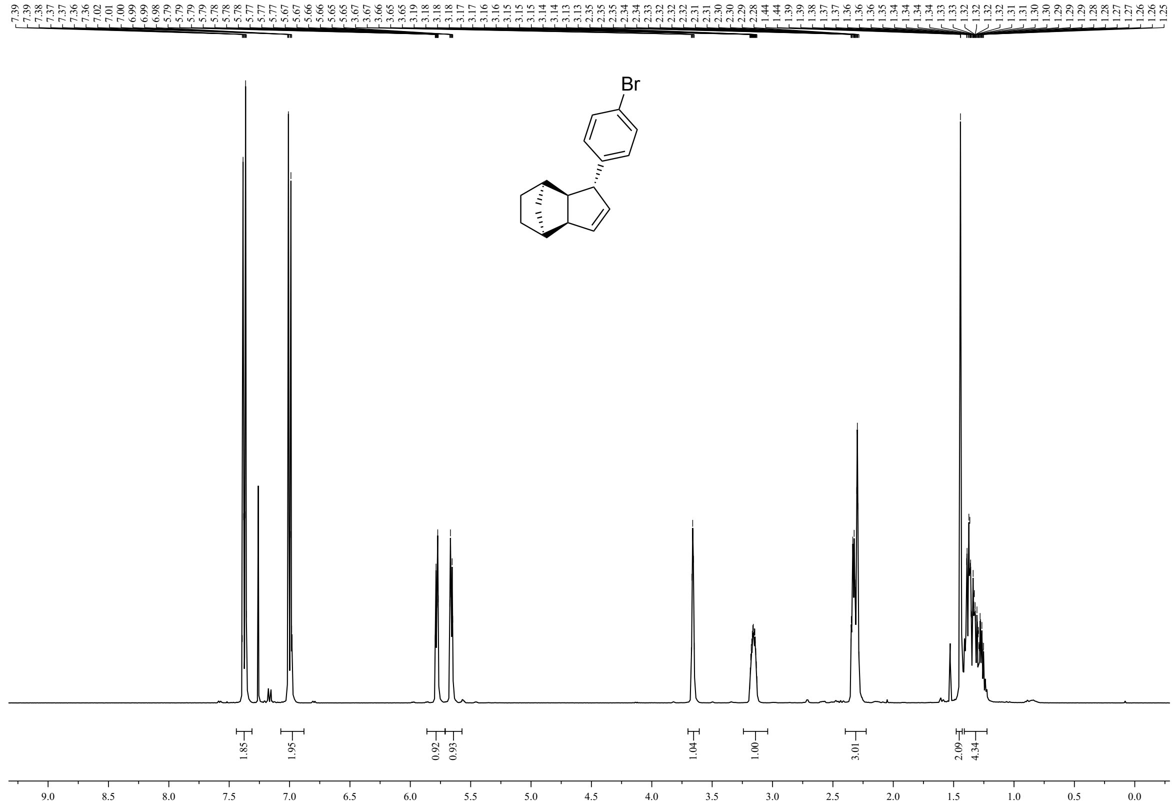

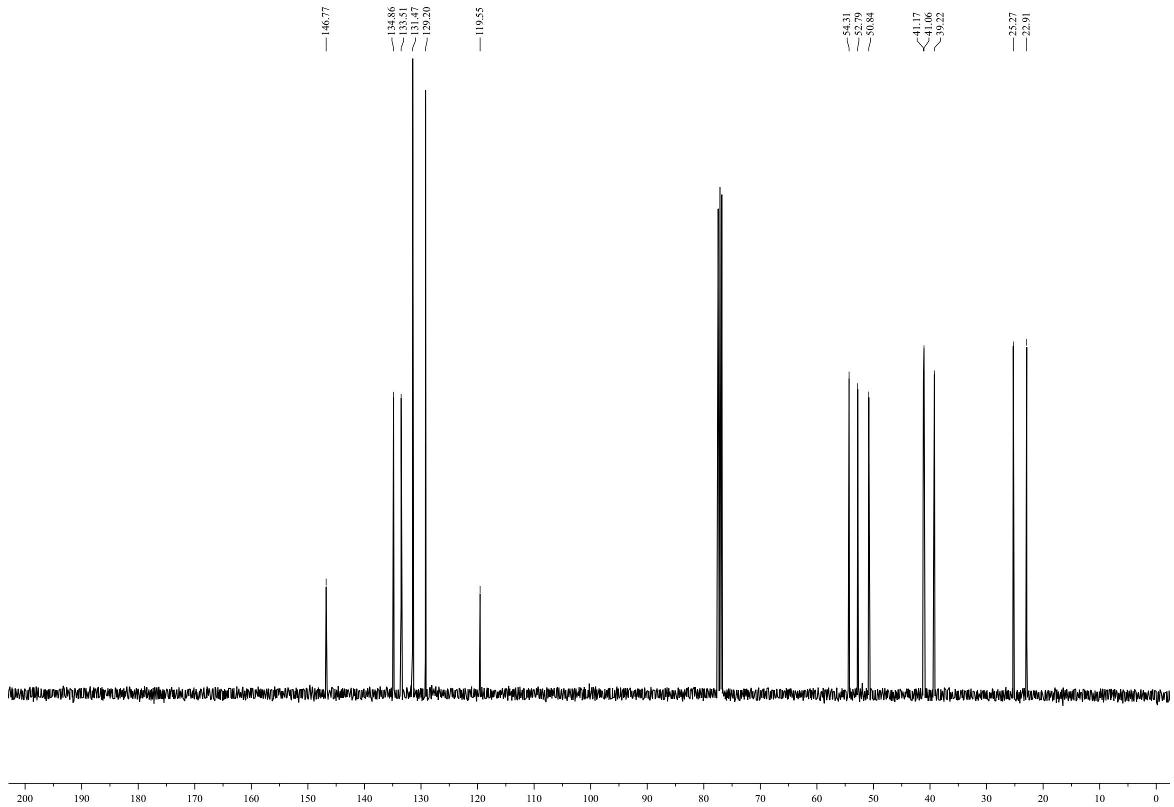

Figure S43: ^{1}H-NMR (top) and ^{13}C-NMR (bottom) of (–)-**3bb**.

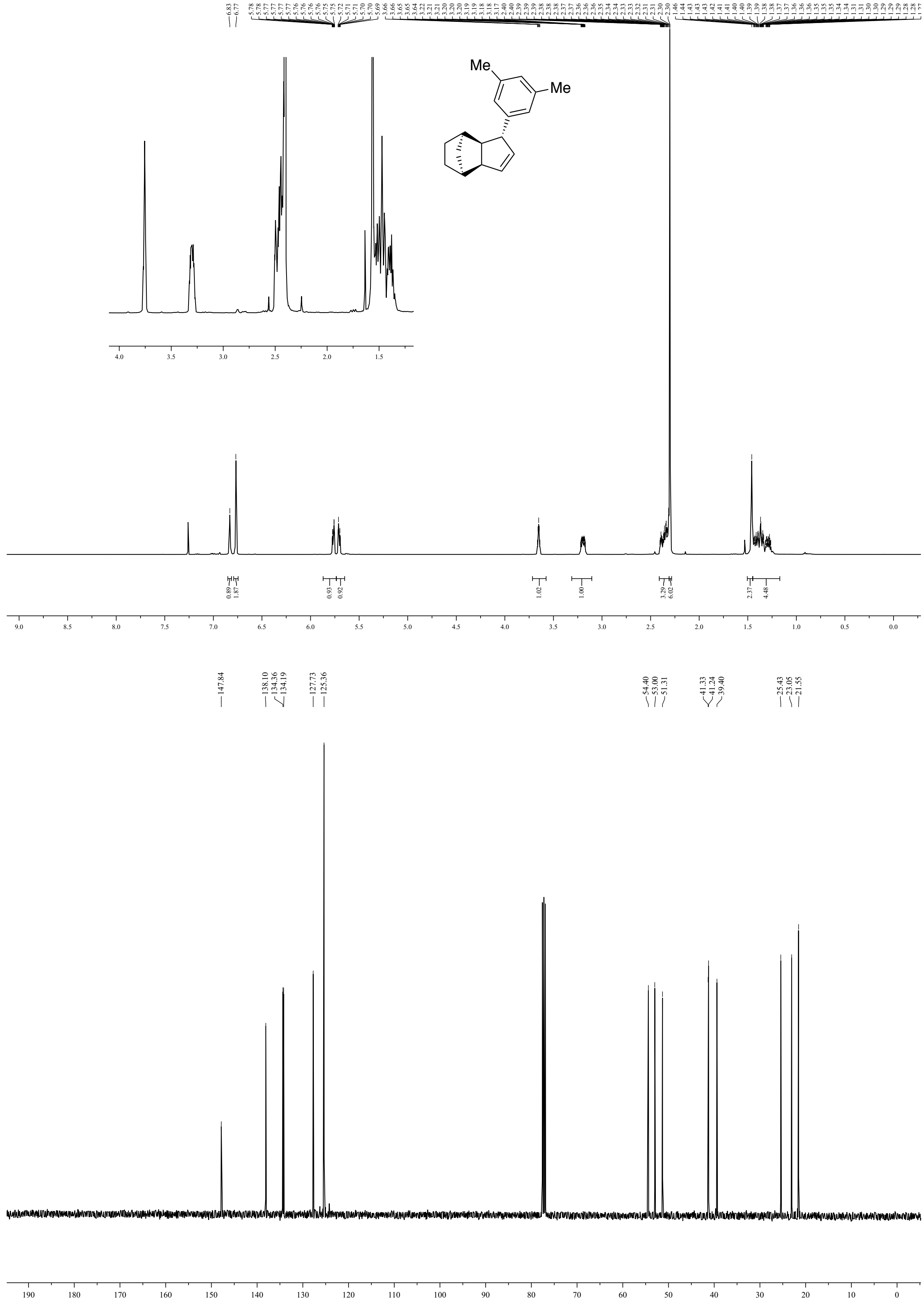

Figure S44: ^{1}H-NMR (top) and ^{13}C-NMR (bottom) of (–)-**3bc**.

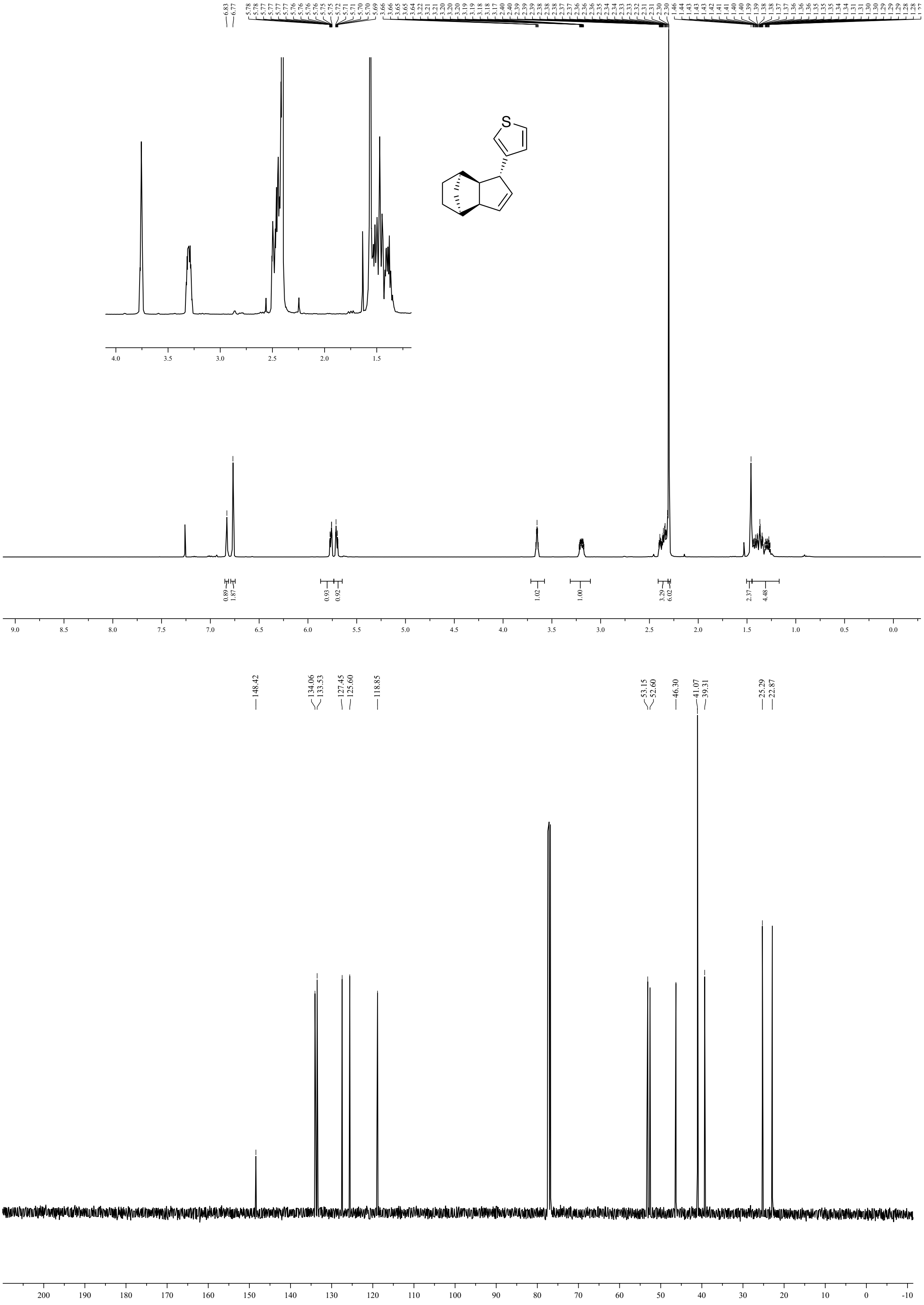

Figure S45: ^{1}H-NMR (top) and ^{13}C-NMR (bottom) of (–)-**3bd**.

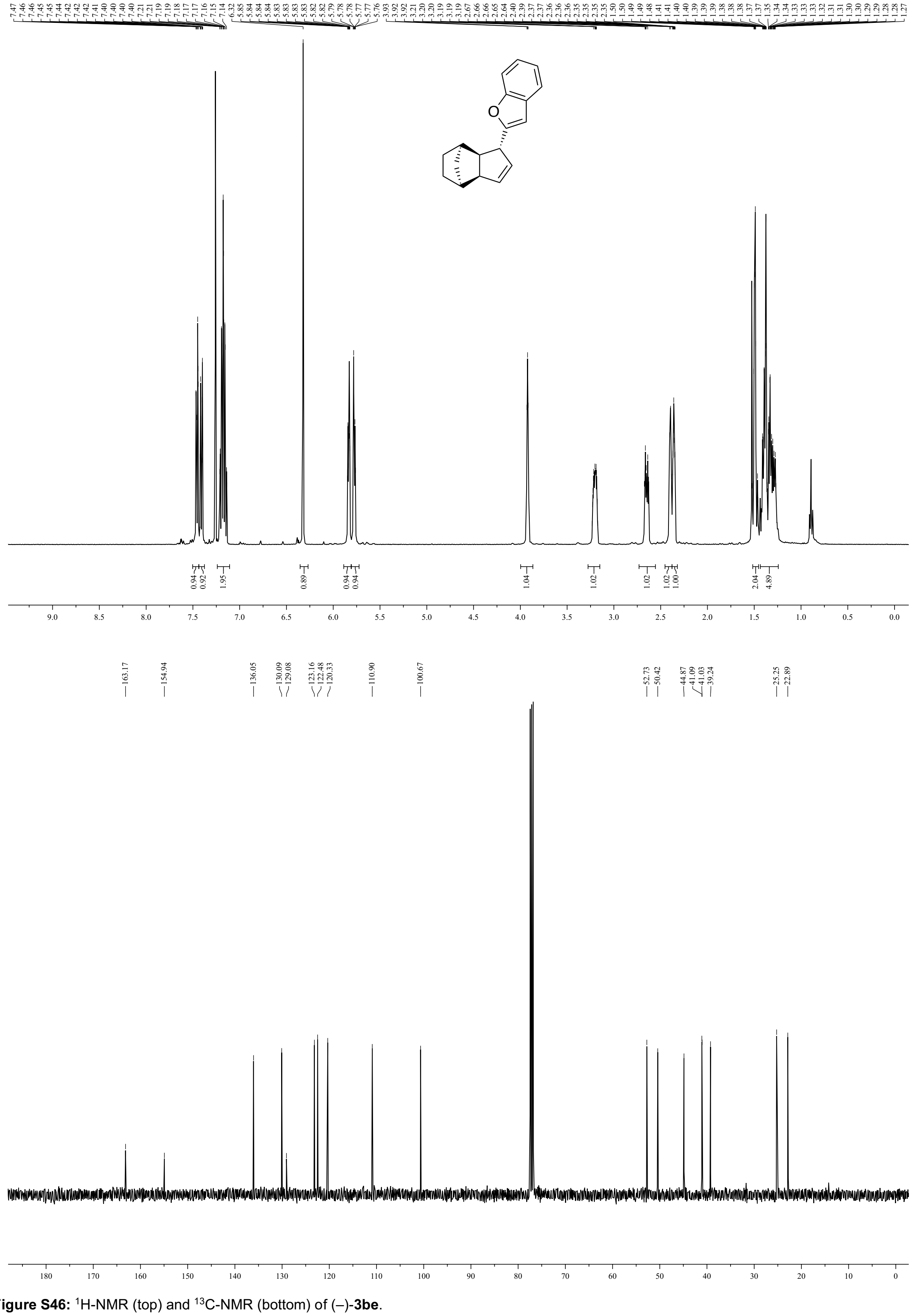

Figure S46: ^{1}H-NMR (top) and ^{13}C-NMR (bottom) of (–)-**3be**.

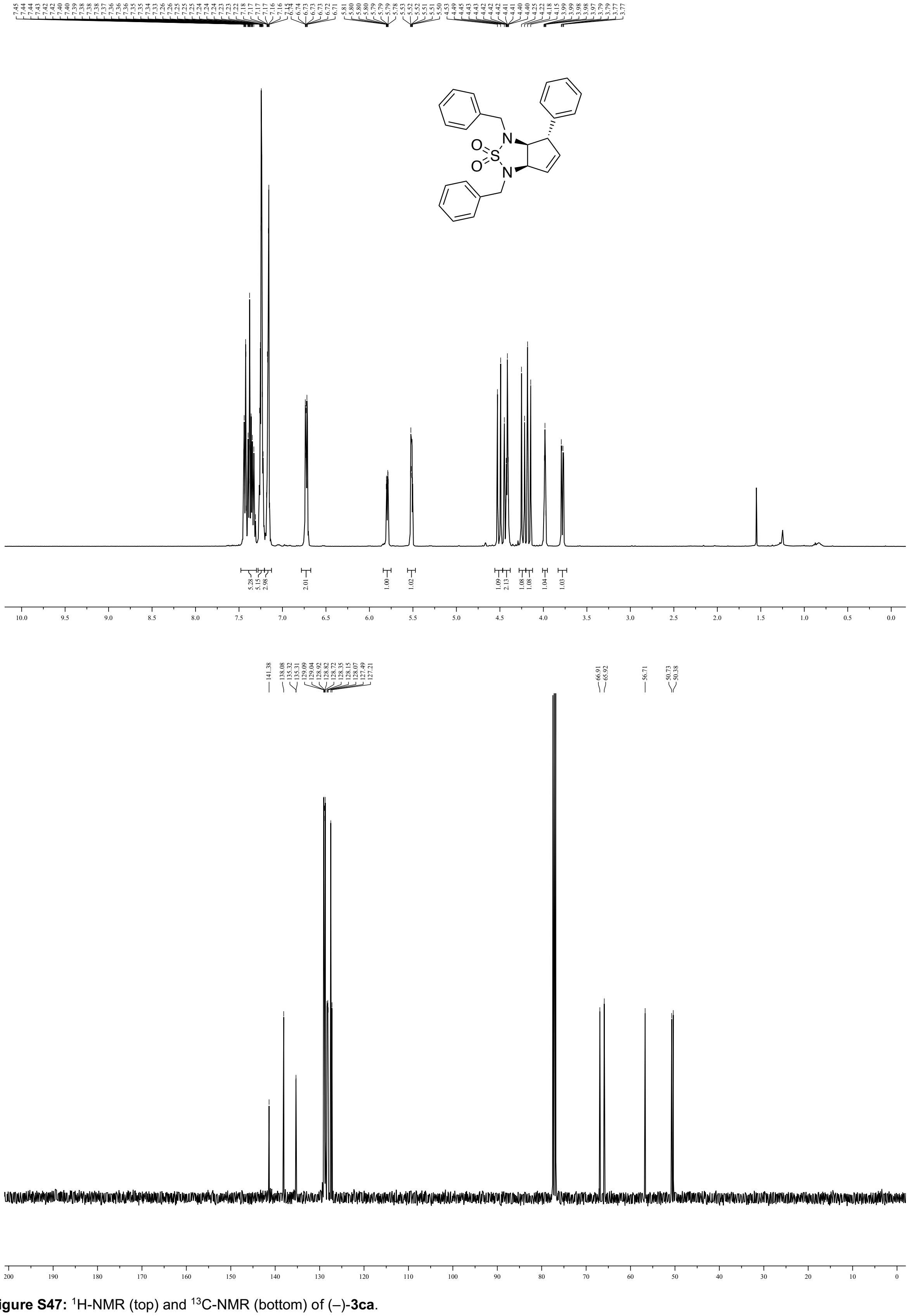

Figure S47: ^{1}H-NMR (top) and ^{13}C-NMR (bottom) of (–)-**3ca**.

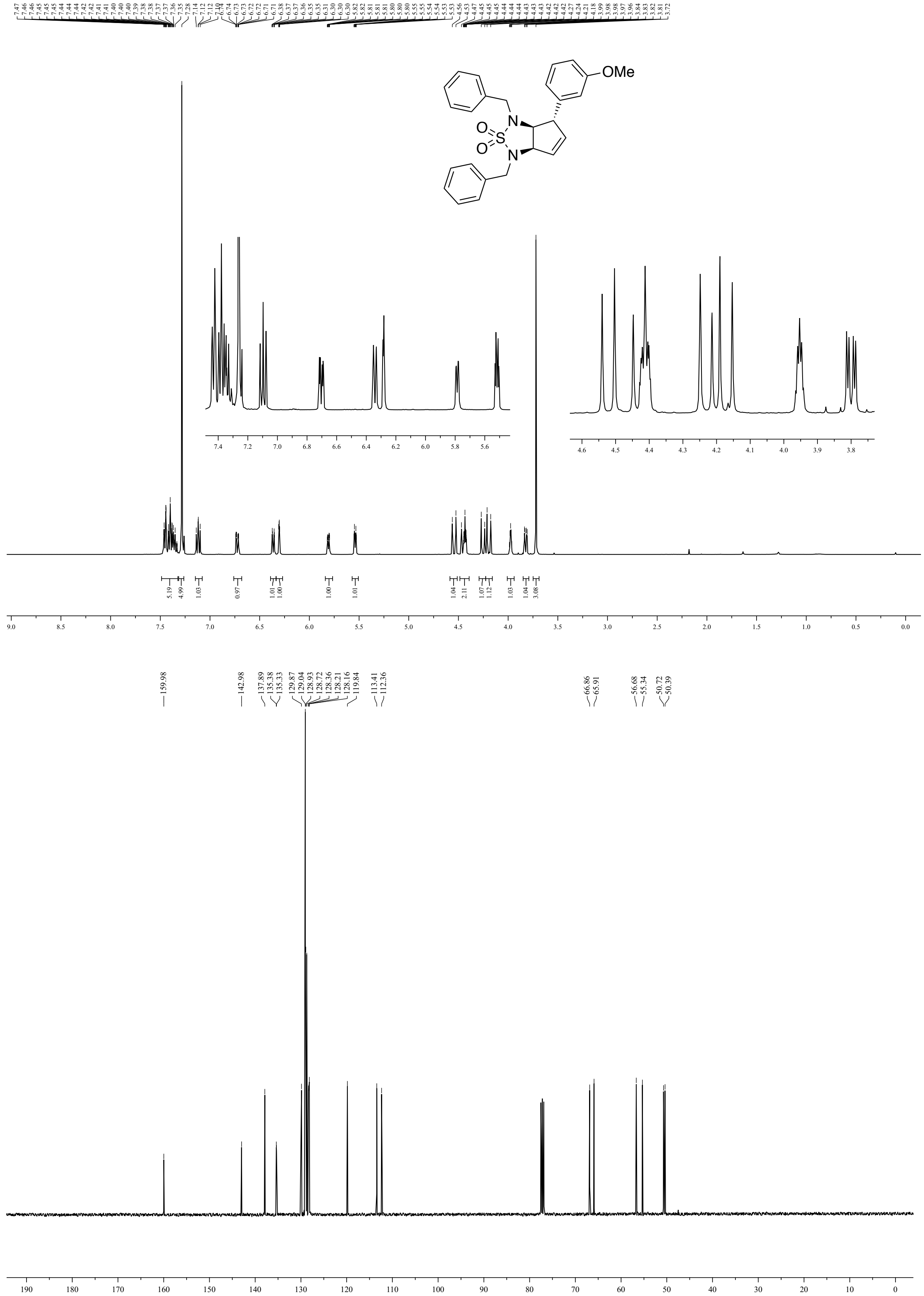

Figure S48: ^{1}H-NMR (top) and ^{13}C-NMR (bottom) of (–)-**3cb**.

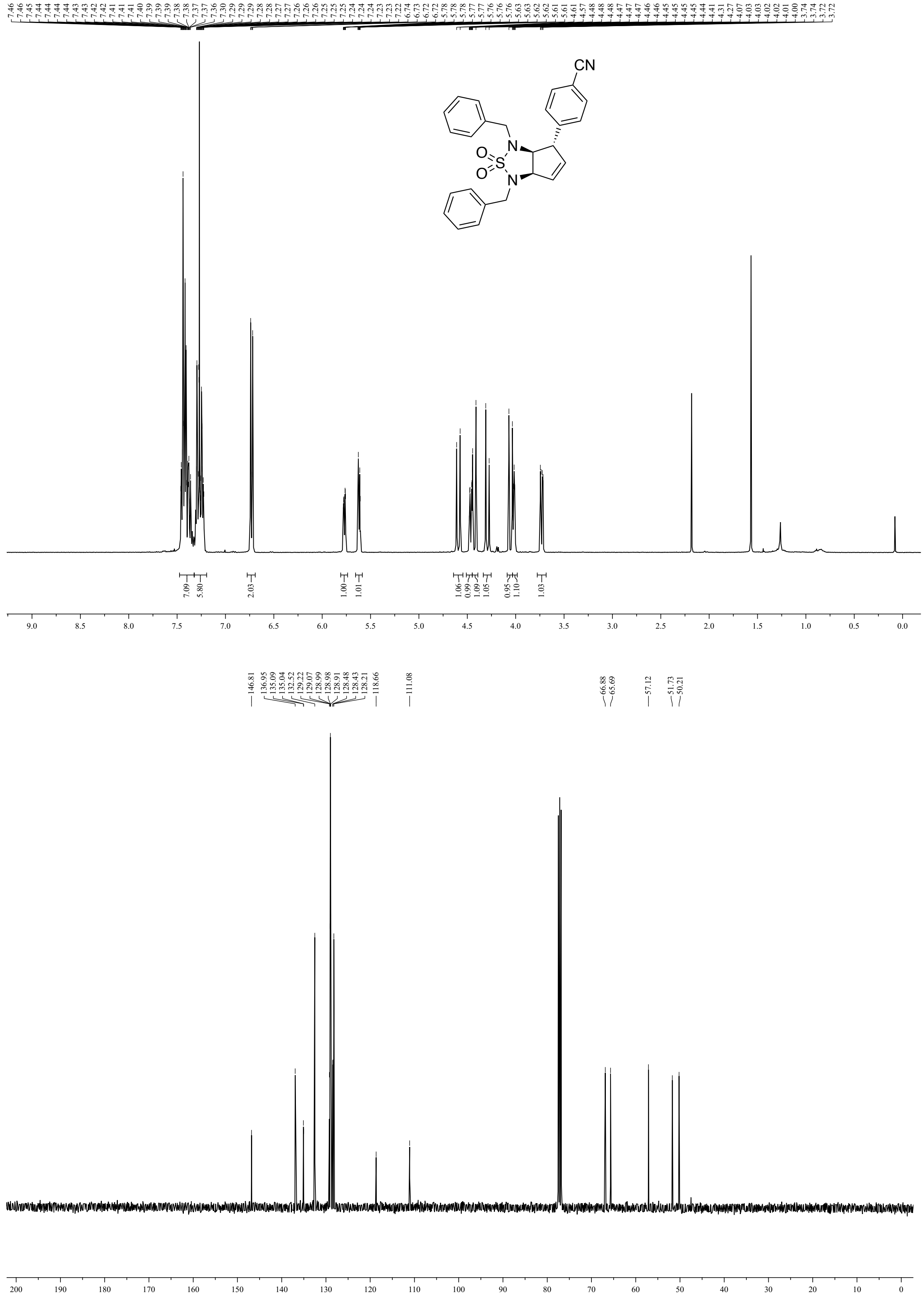

Figure S49: ^{1}H-NMR (top) and ^{13}C-NMR (bottom) of (–)-**3cc**.

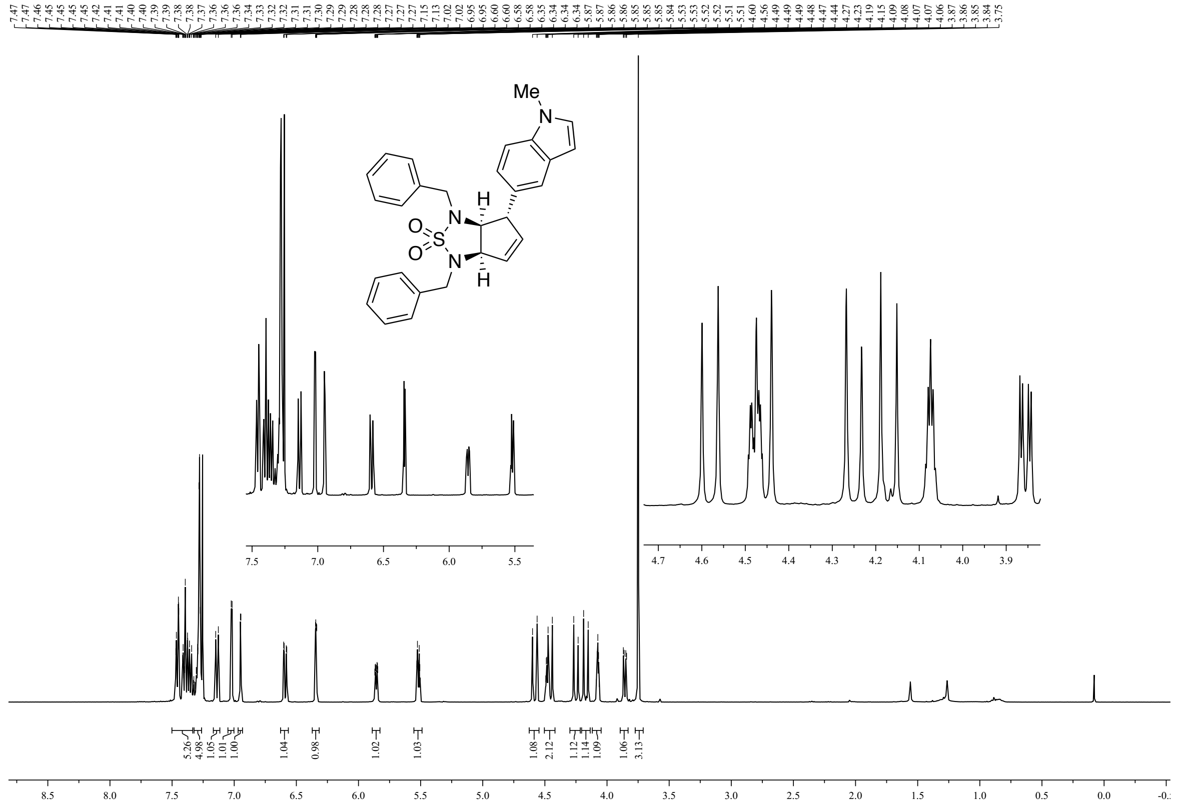

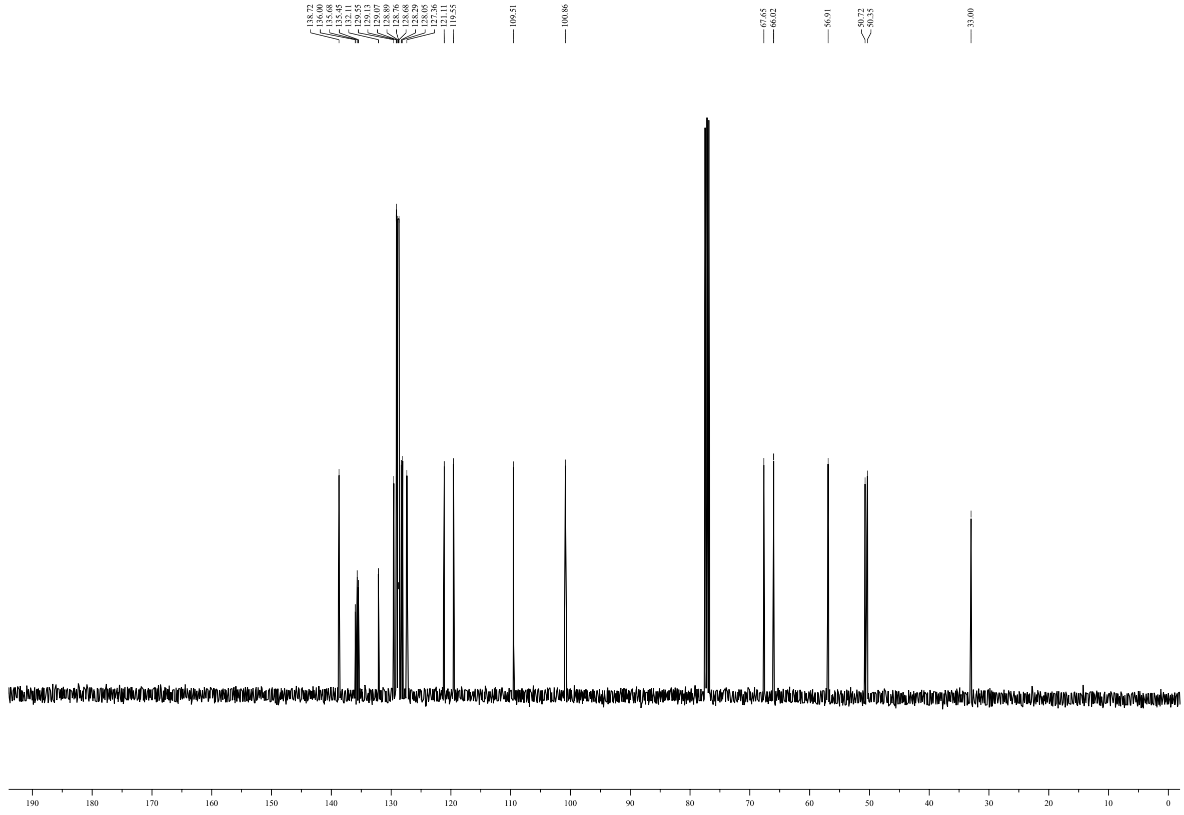

Figure S50: ^{1}H-NMR (top) and ^{13}C-NMR (bottom) of (–)-**3cd**.

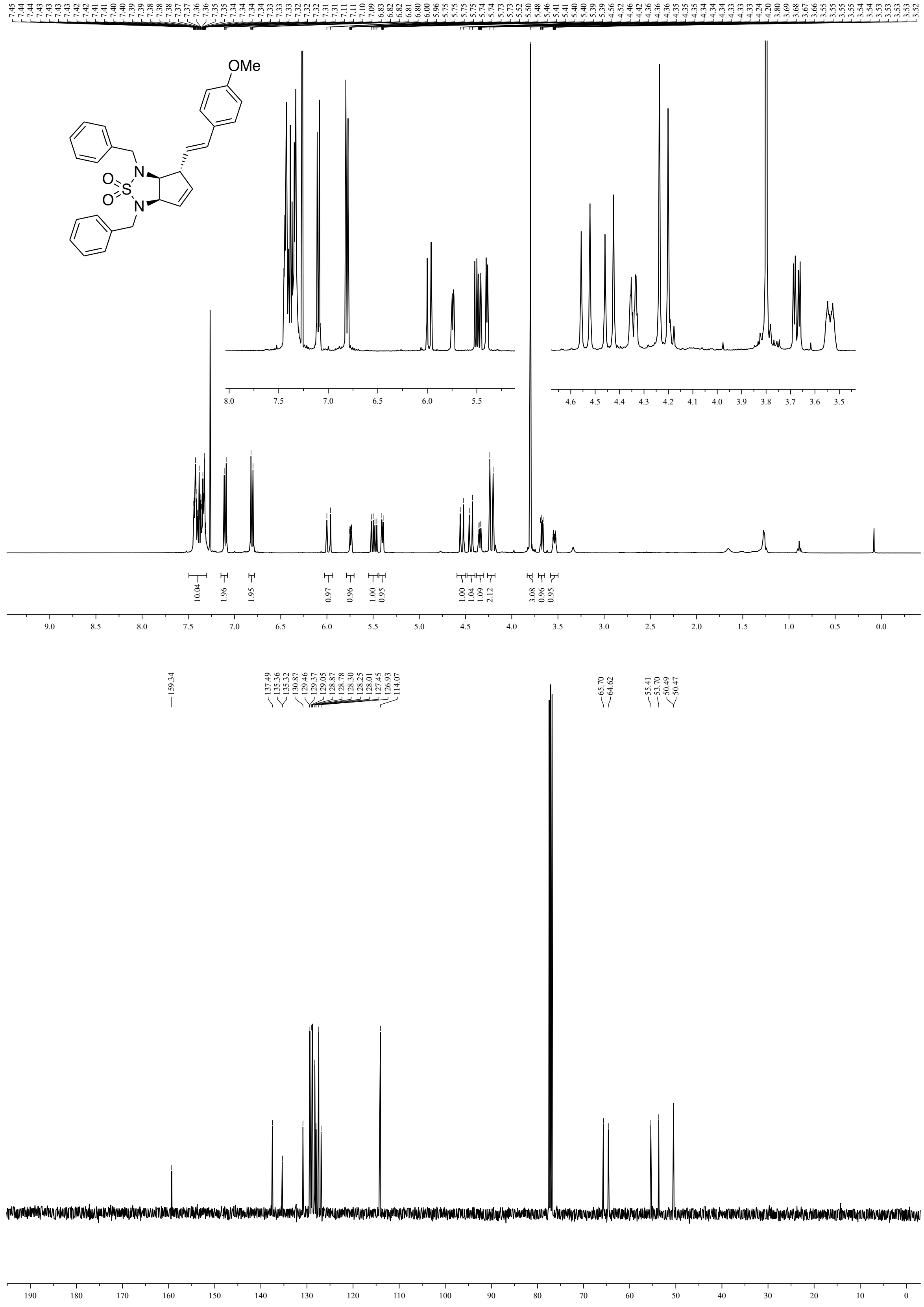

Figure S51: ^{1}H-NMR (top) and ^{13}C-NMR (bottom) of (–)-**3ce**.

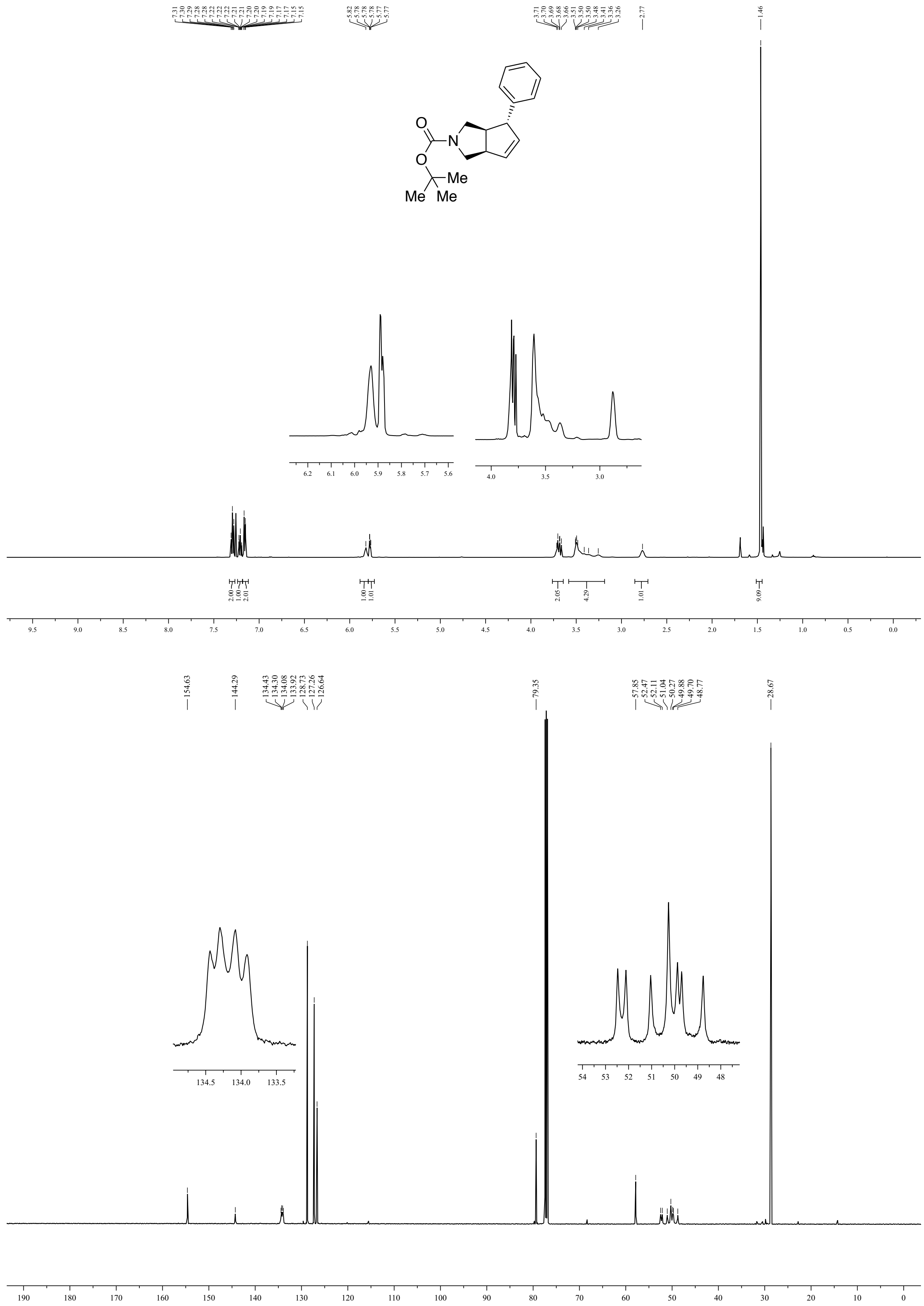

Figure S52: ^{1}H-NMR (top) and ^{13}C-NMR (bottom) of (–)-**3da**.

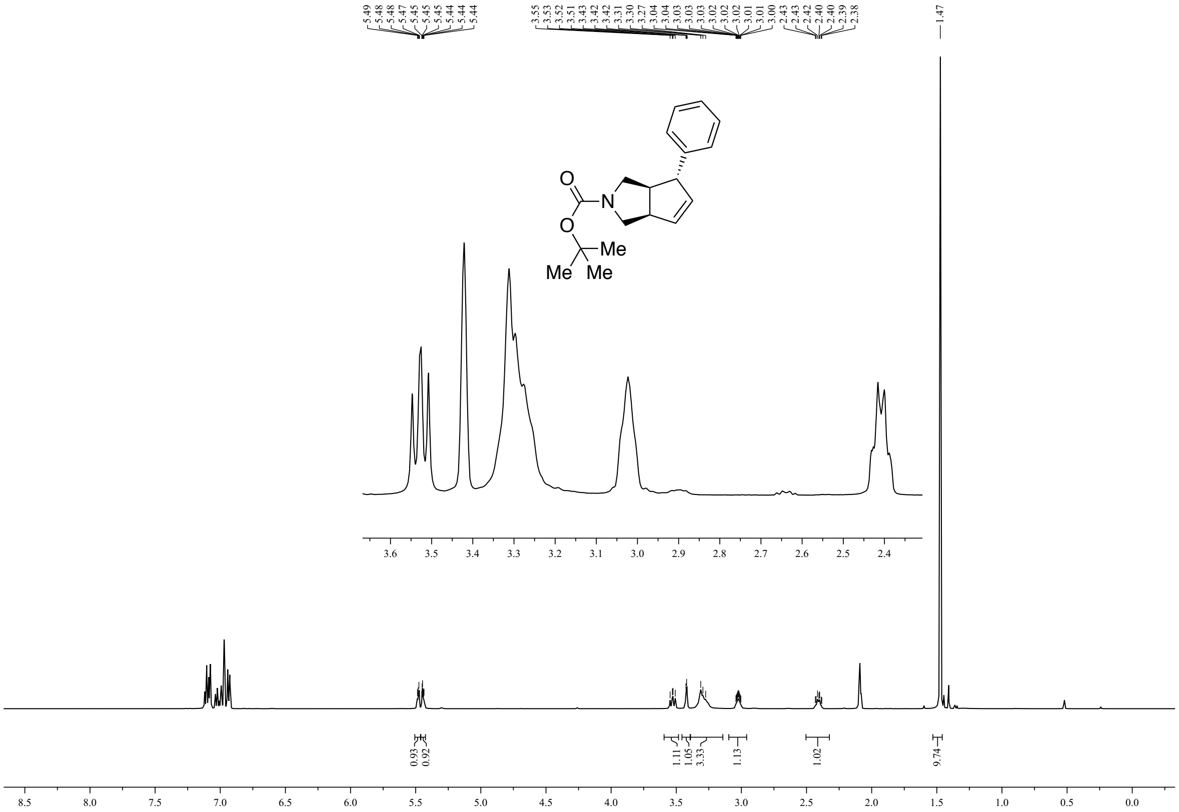

Figure S53: High-temperature NMR of (–)-**3da**.

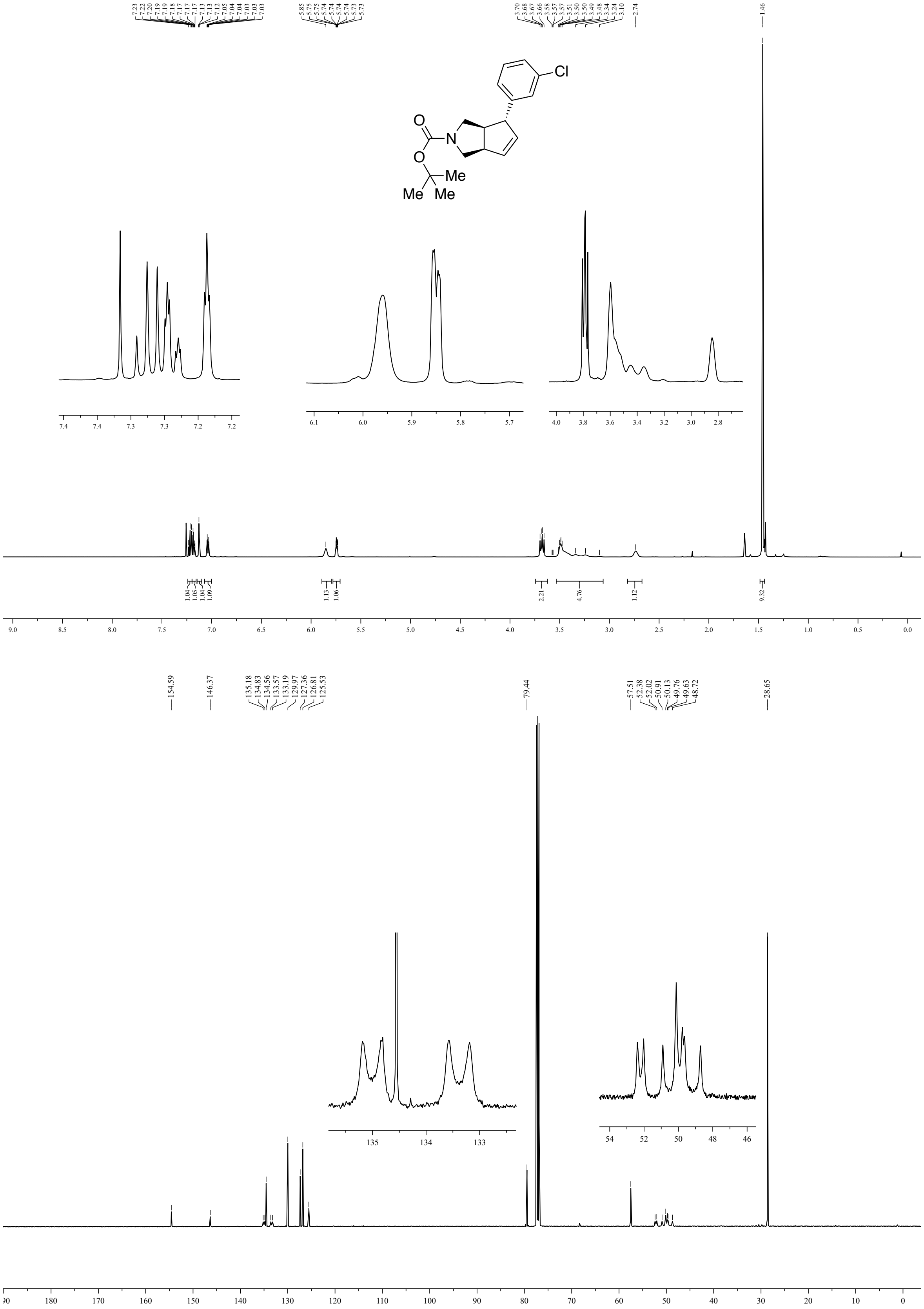

Figure S54: ^{1}H-NMR (top) and ^{13}C-NMR (bottom) of (–)-**3db**.

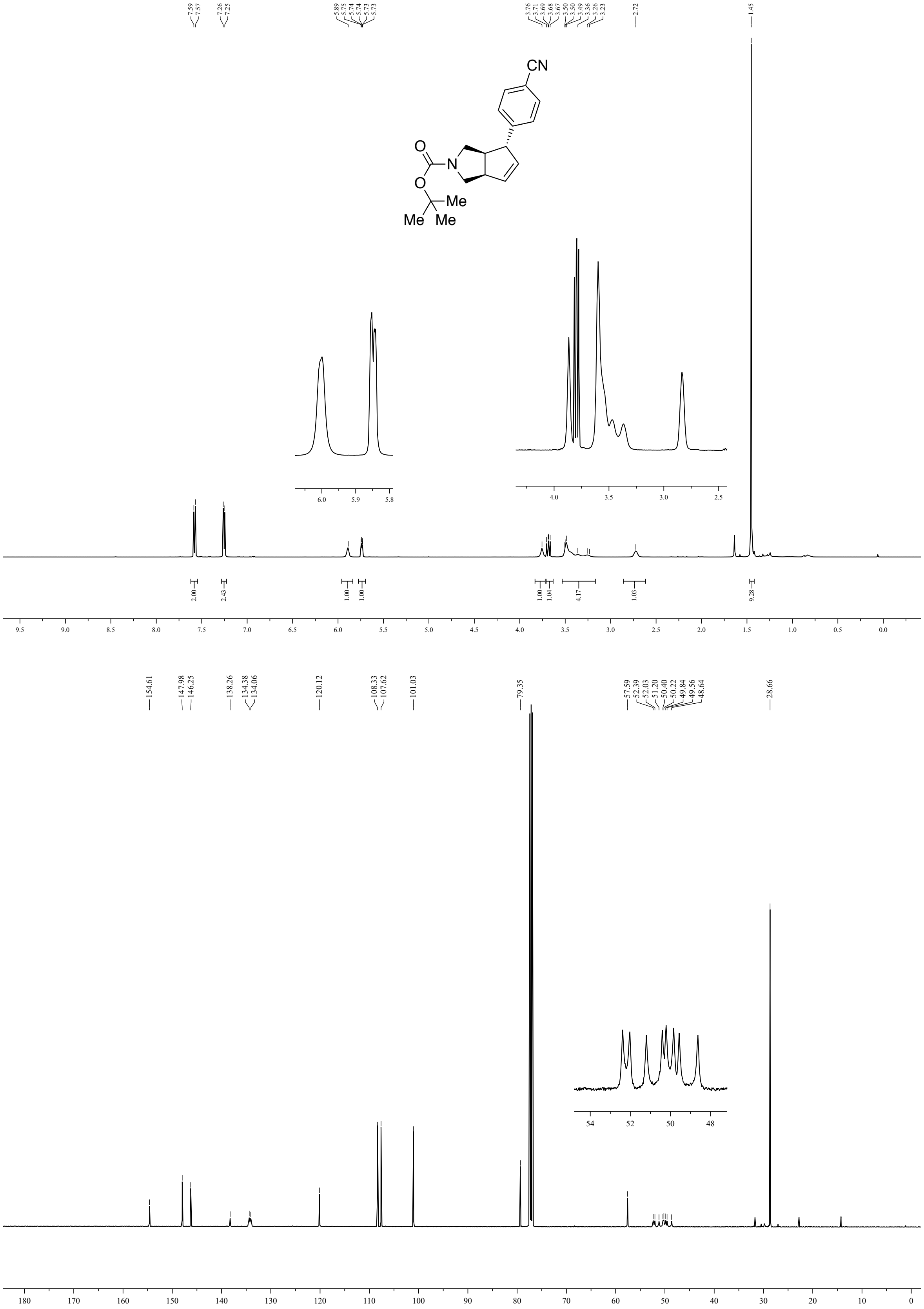

Figure S55: ^{1}H-NMR (top) and ^{13}C-NMR (bottom) of (–)-**3dc**.

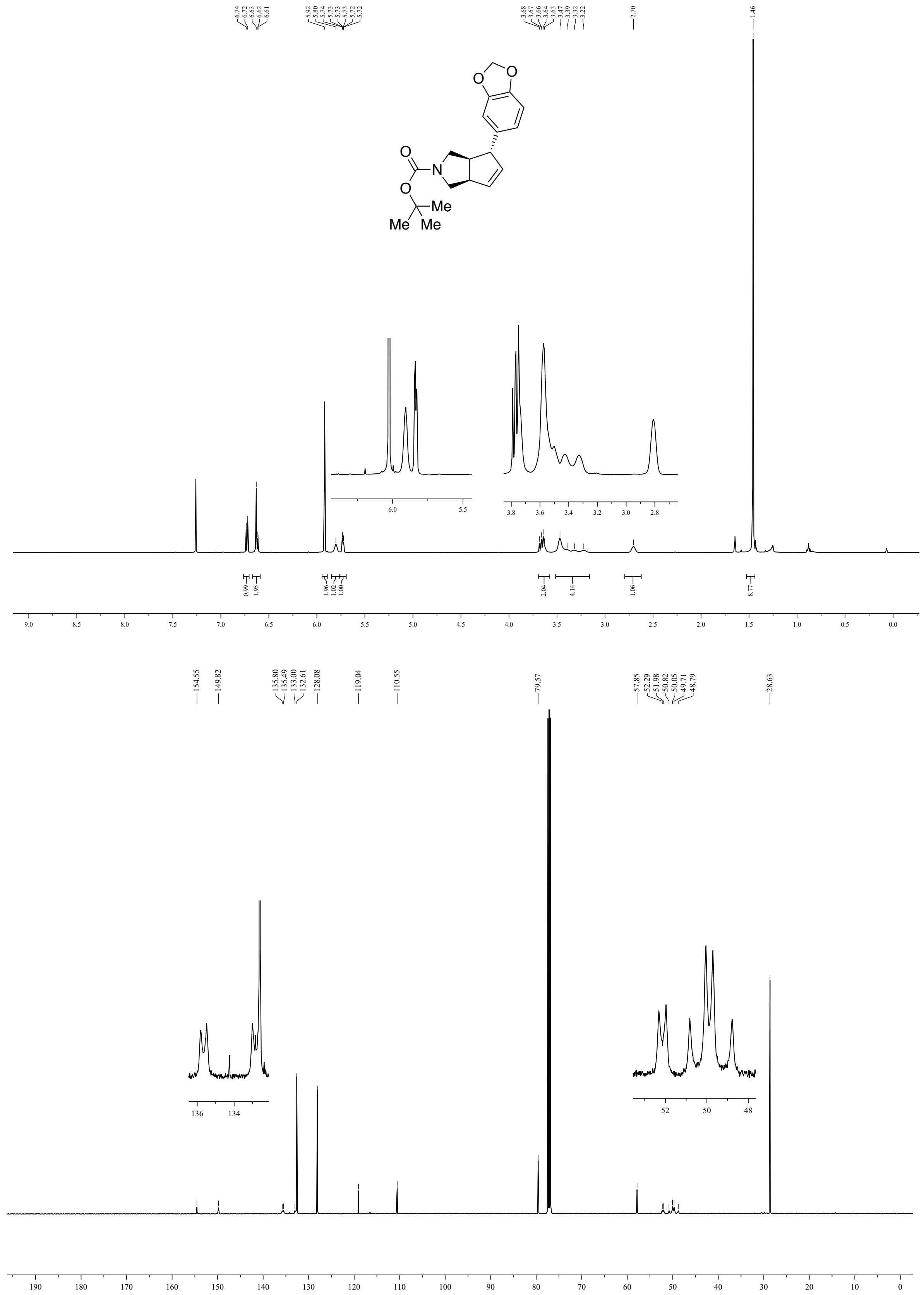

Figure S56: ^{1}H-NMR (top) and ^{13}C-NMR (bottom) of (–)-**3dd**.

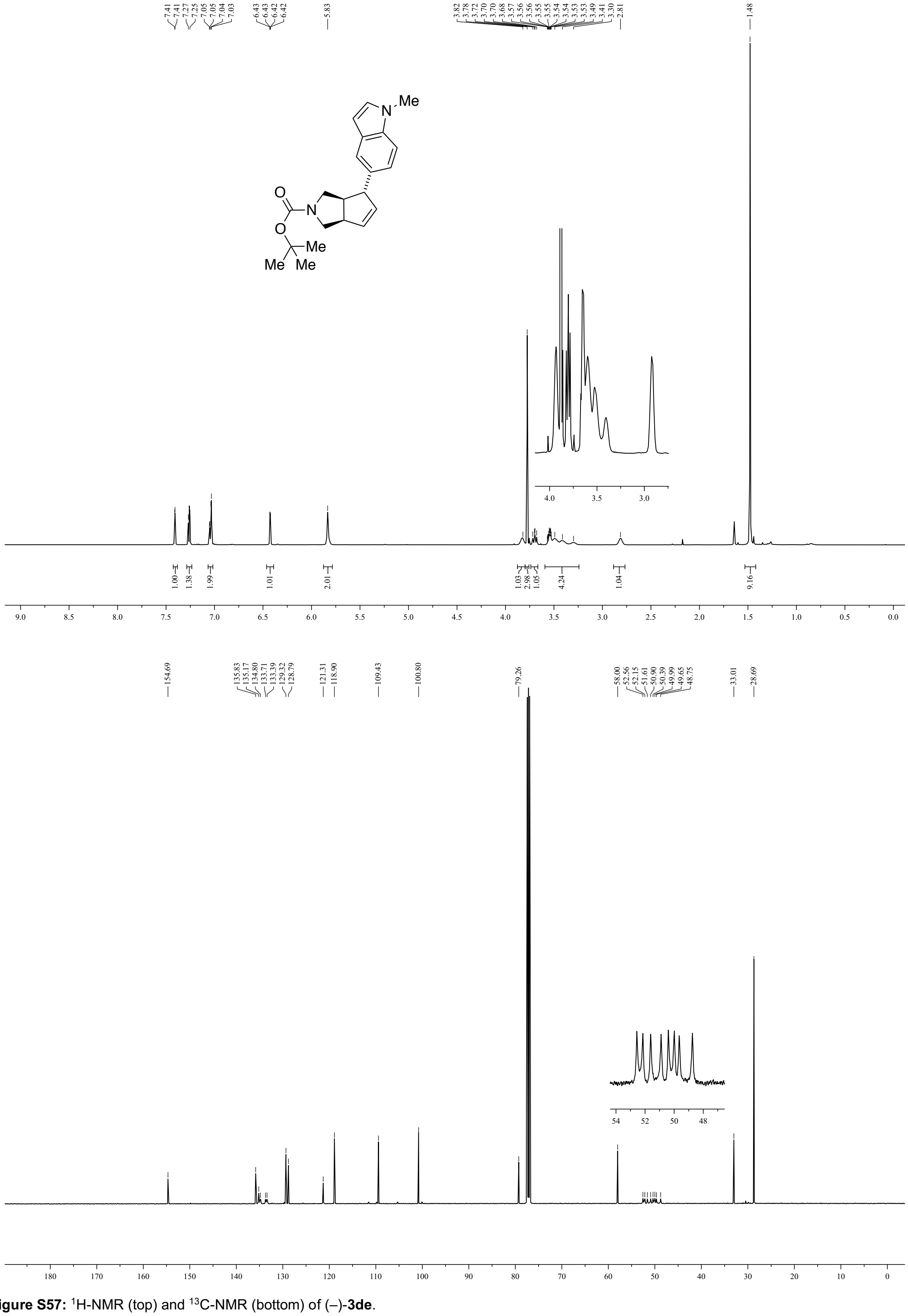

Figure S57: ^{1}H-NMR (top) and ^{13}C-NMR (bottom) of (–)-**3de**.

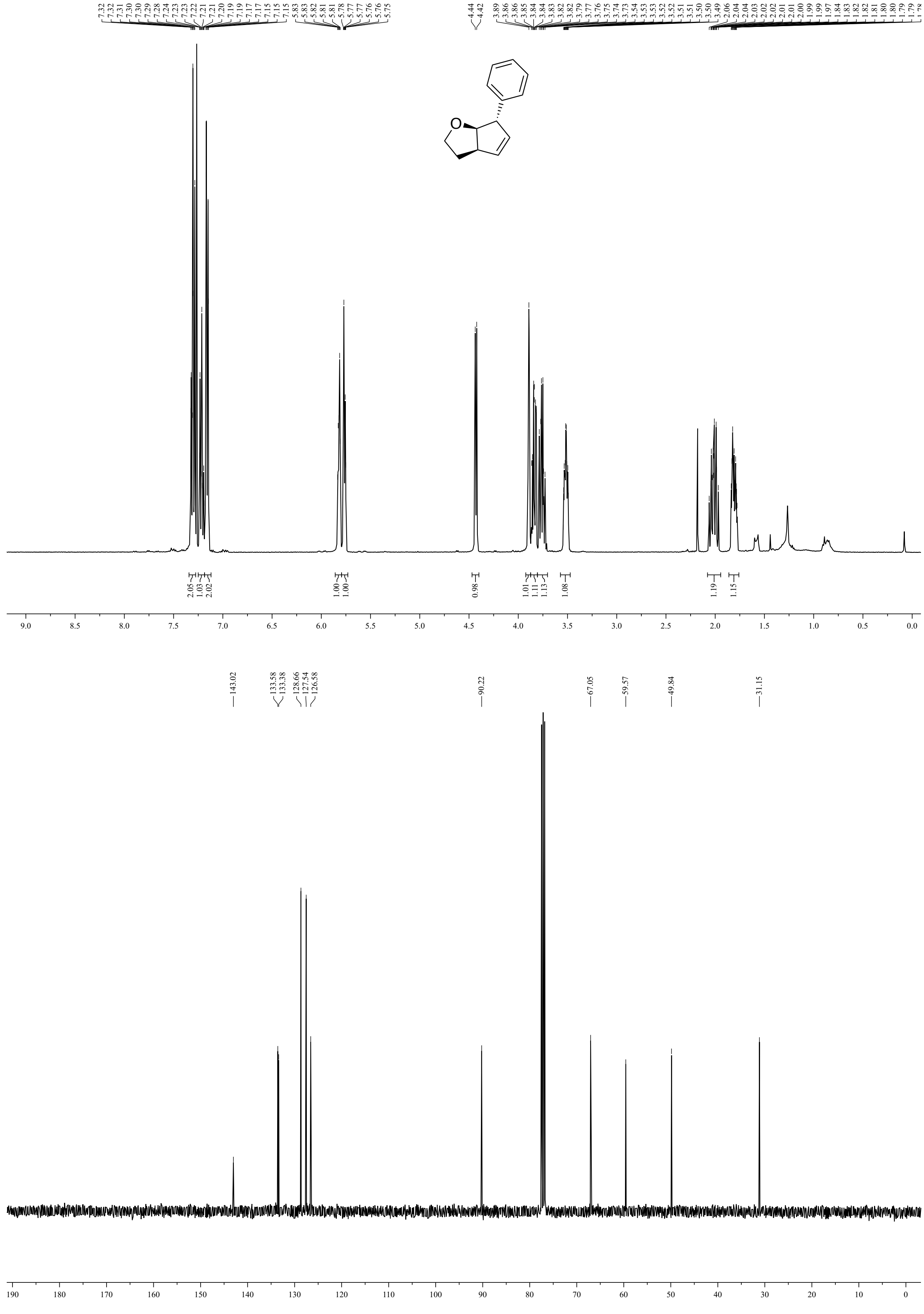

Figure S58: ^{1}H-NMR (top) and ^{13}C-NMR (bottom) of (–)-**3ea***.

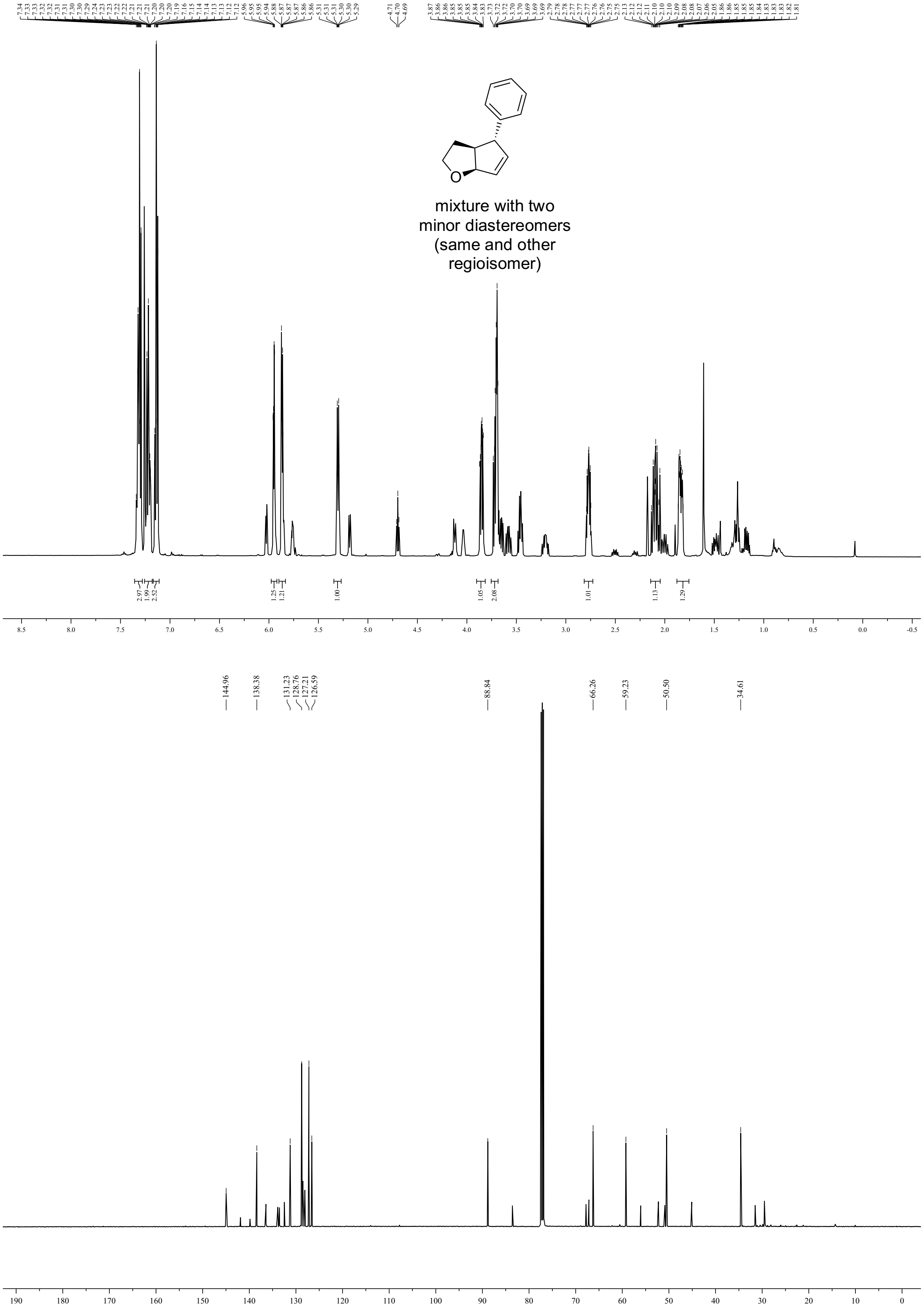

Figure S59: ^{1}H-NMR (top) and ^{13}C-NMR (bottom) of (–)-**3ea**.

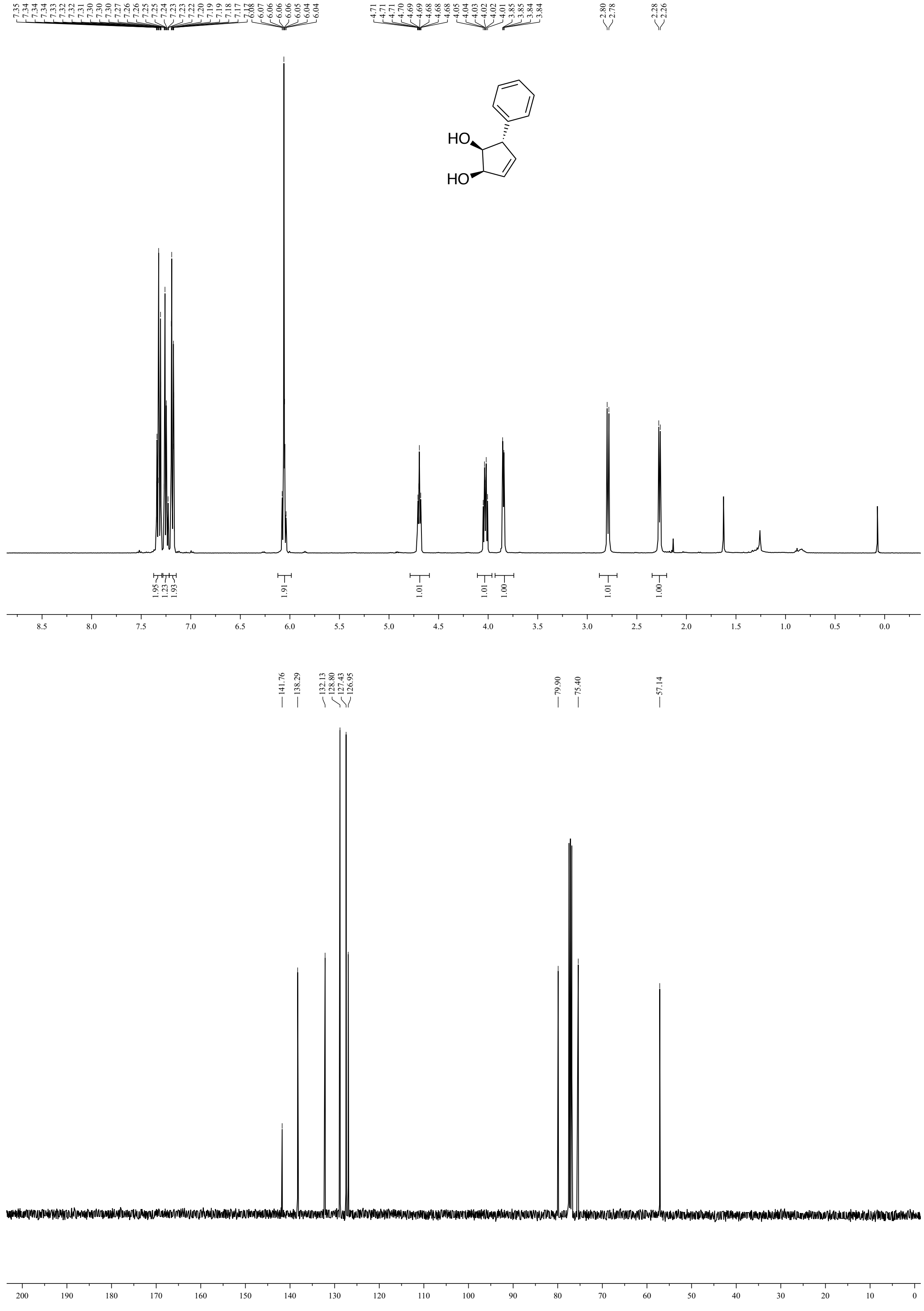

Figure S60: ^{1}H-NMR (top) and ^{13}C-NMR (bottom) of (–)-**4**.

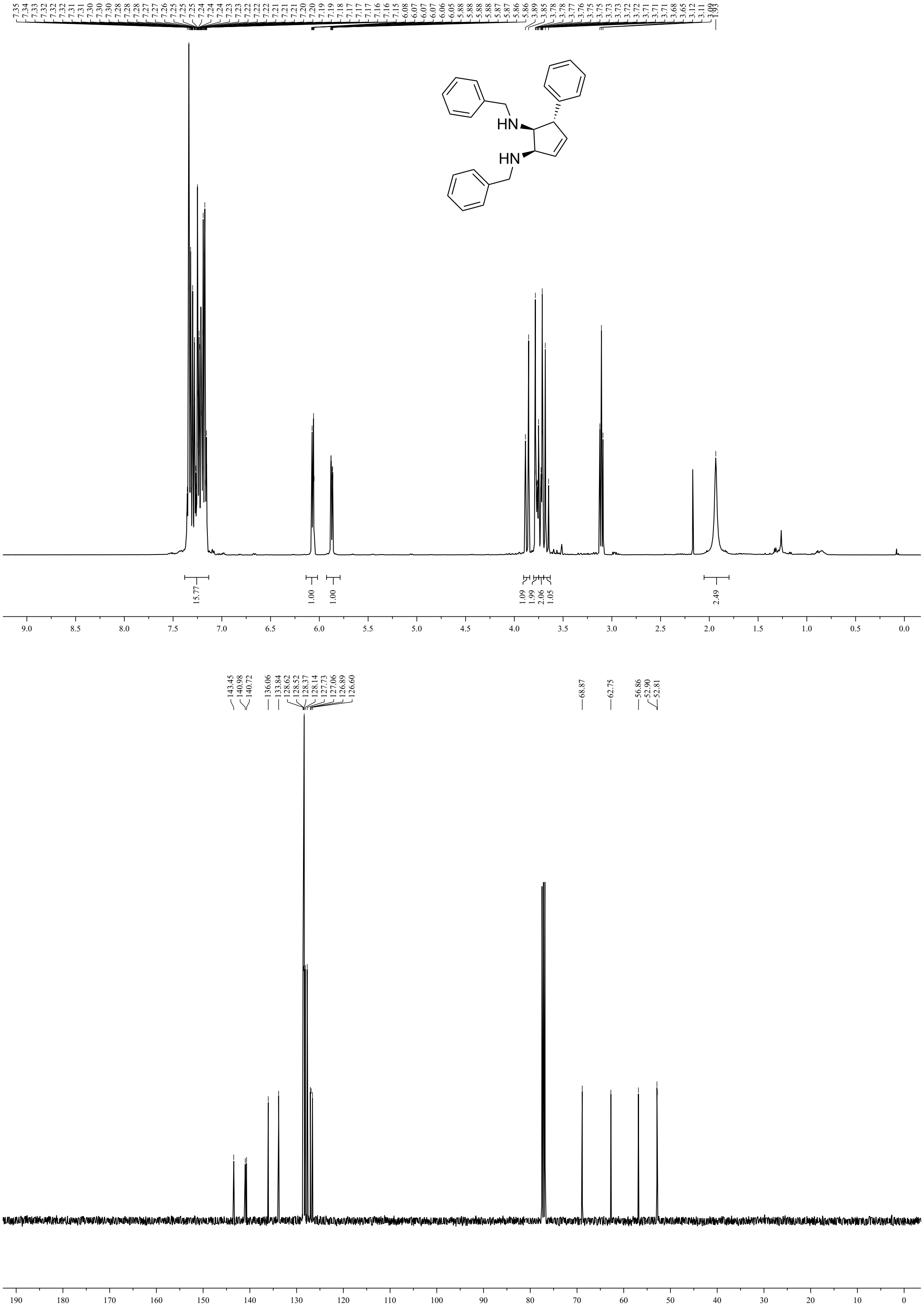

Figure S61: ^{1}H-NMR (top) and ^{13}C-NMR (bottom) of (–)-**5**.

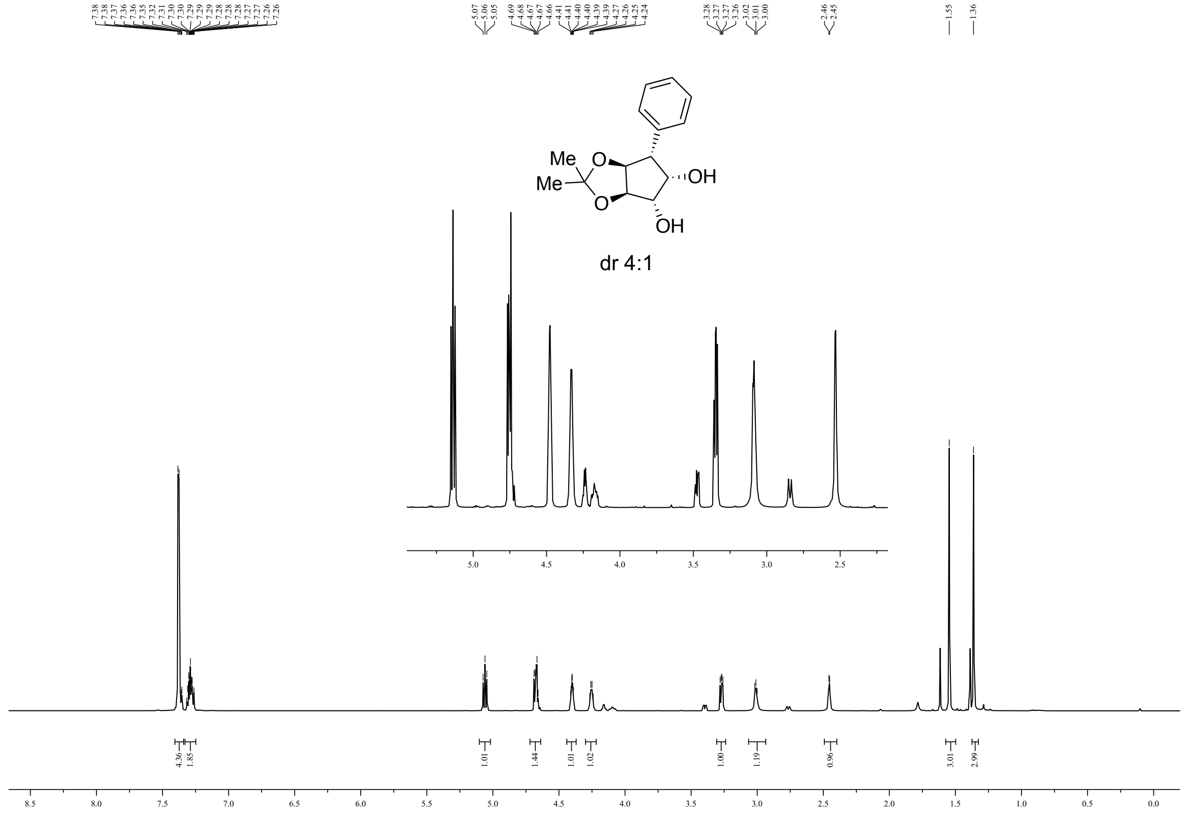

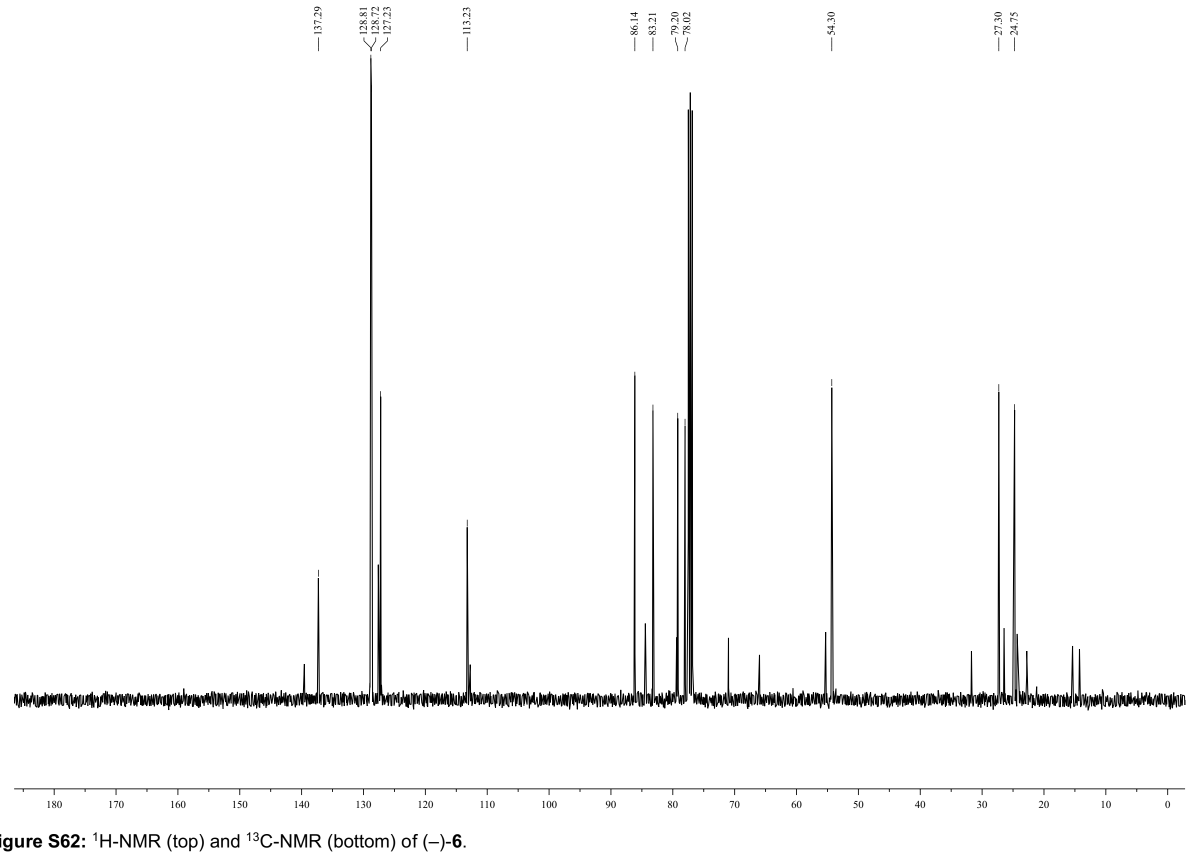

Figure S62: ^{1}H-NMR (top) and ^{13}C-NMR (bottom) of (–)-**6**.

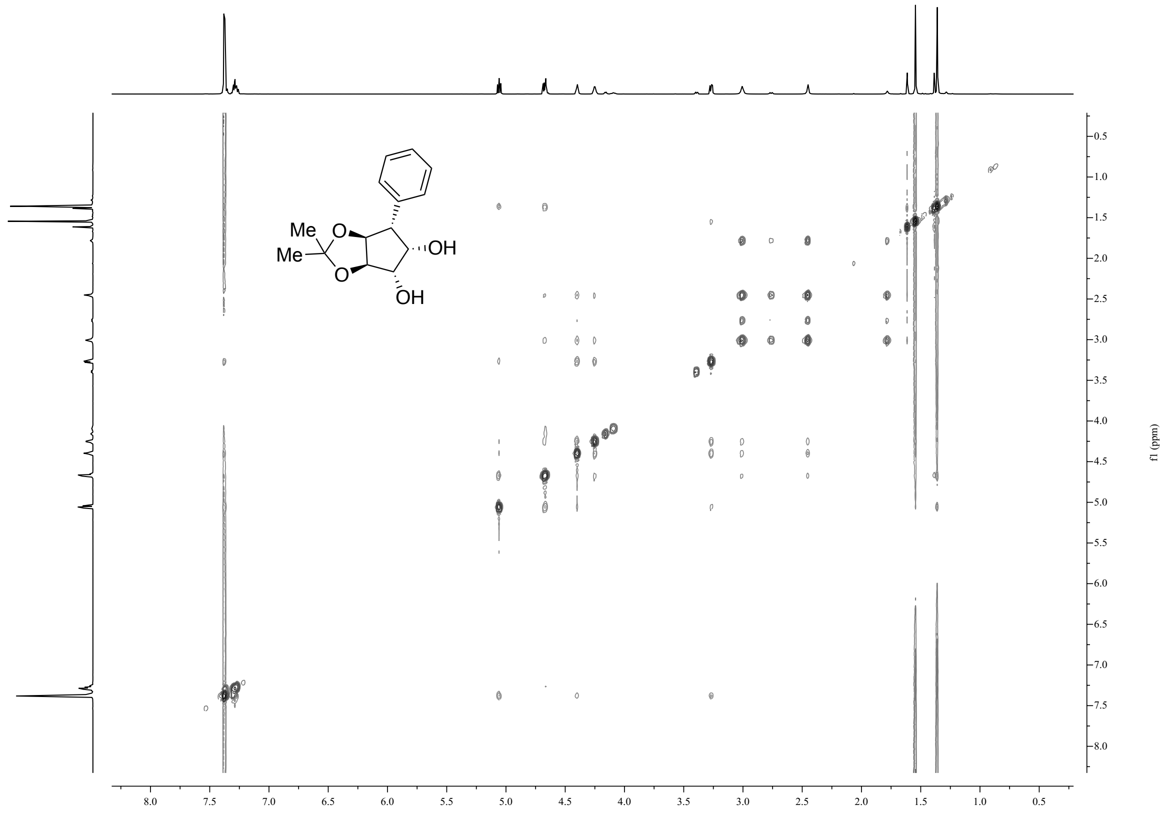

Figure S63: 2D NOE of (–)-**6**.

4. SFC traces

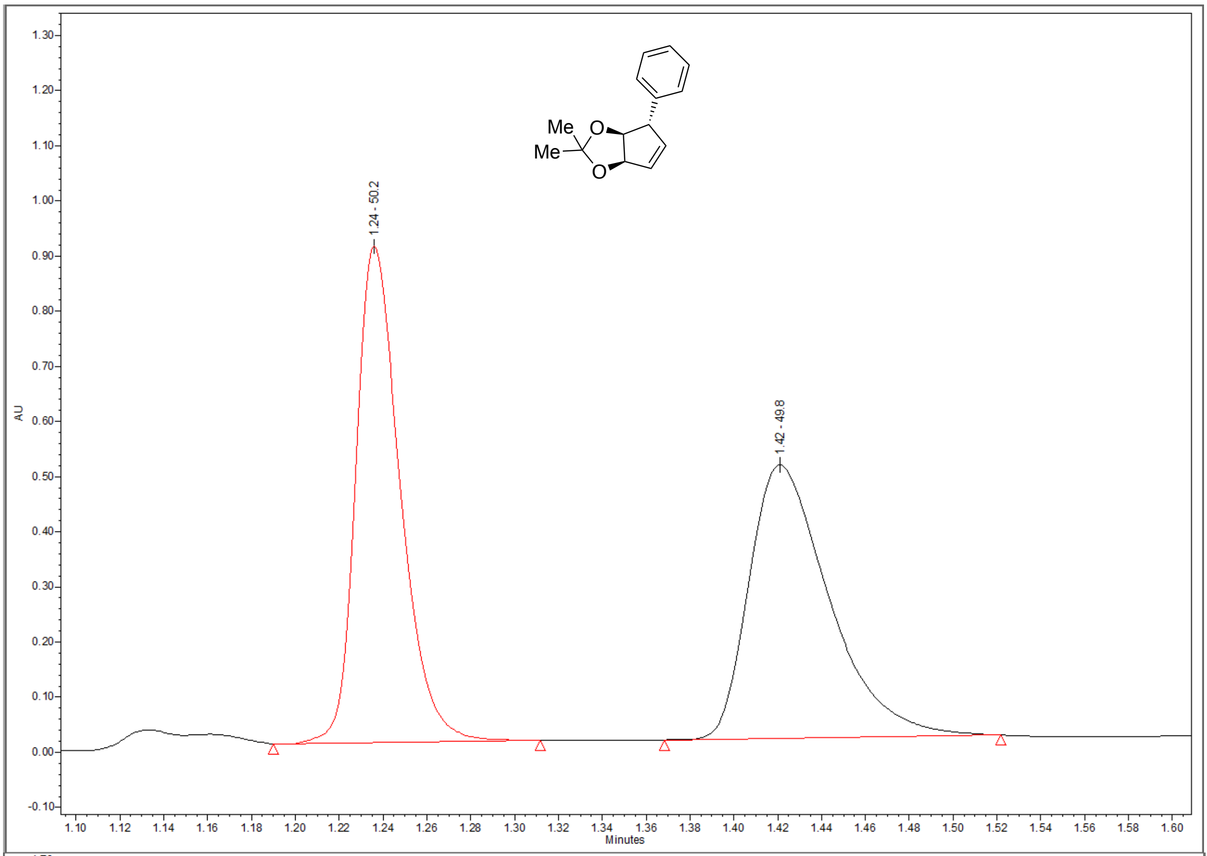

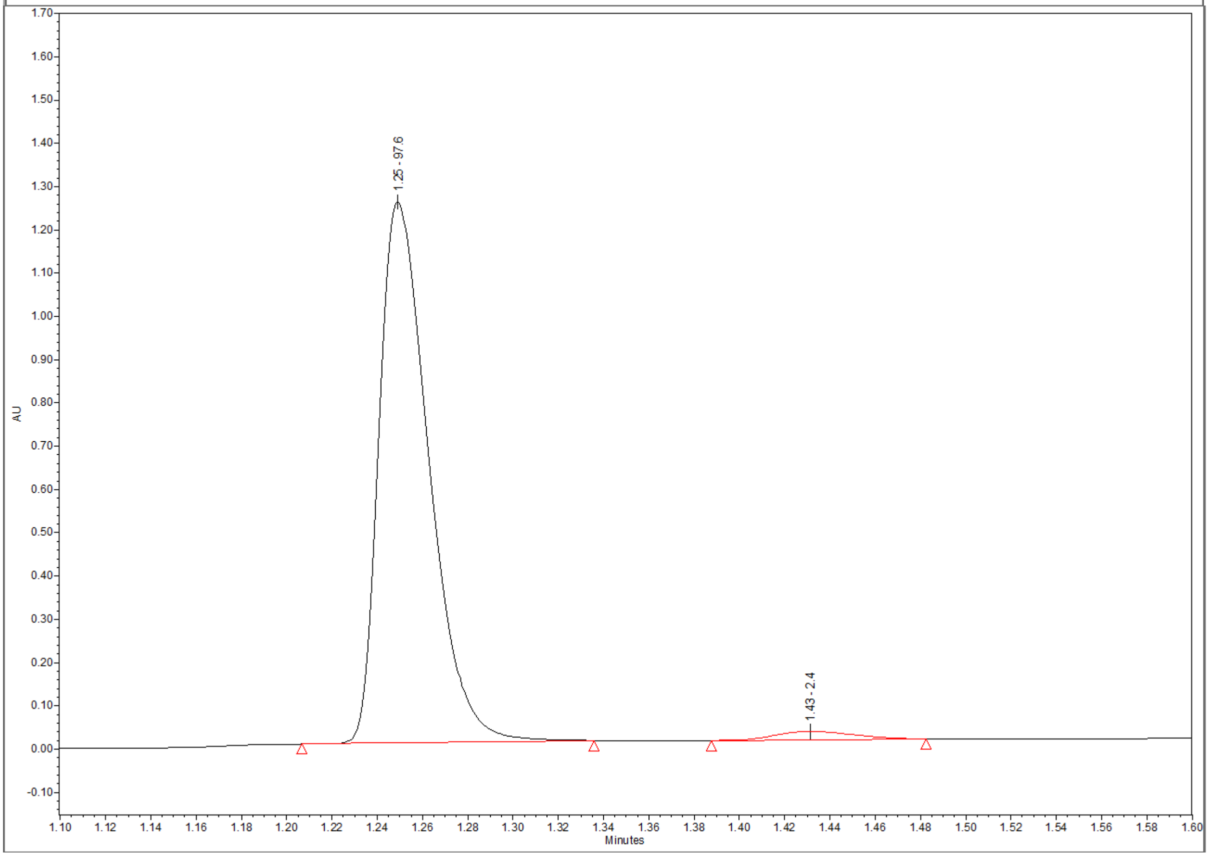

Figure S64: SFC traces of the racemic (±)-**3aa** (top) and enantioenriched (–)-**3aa** (bottom).

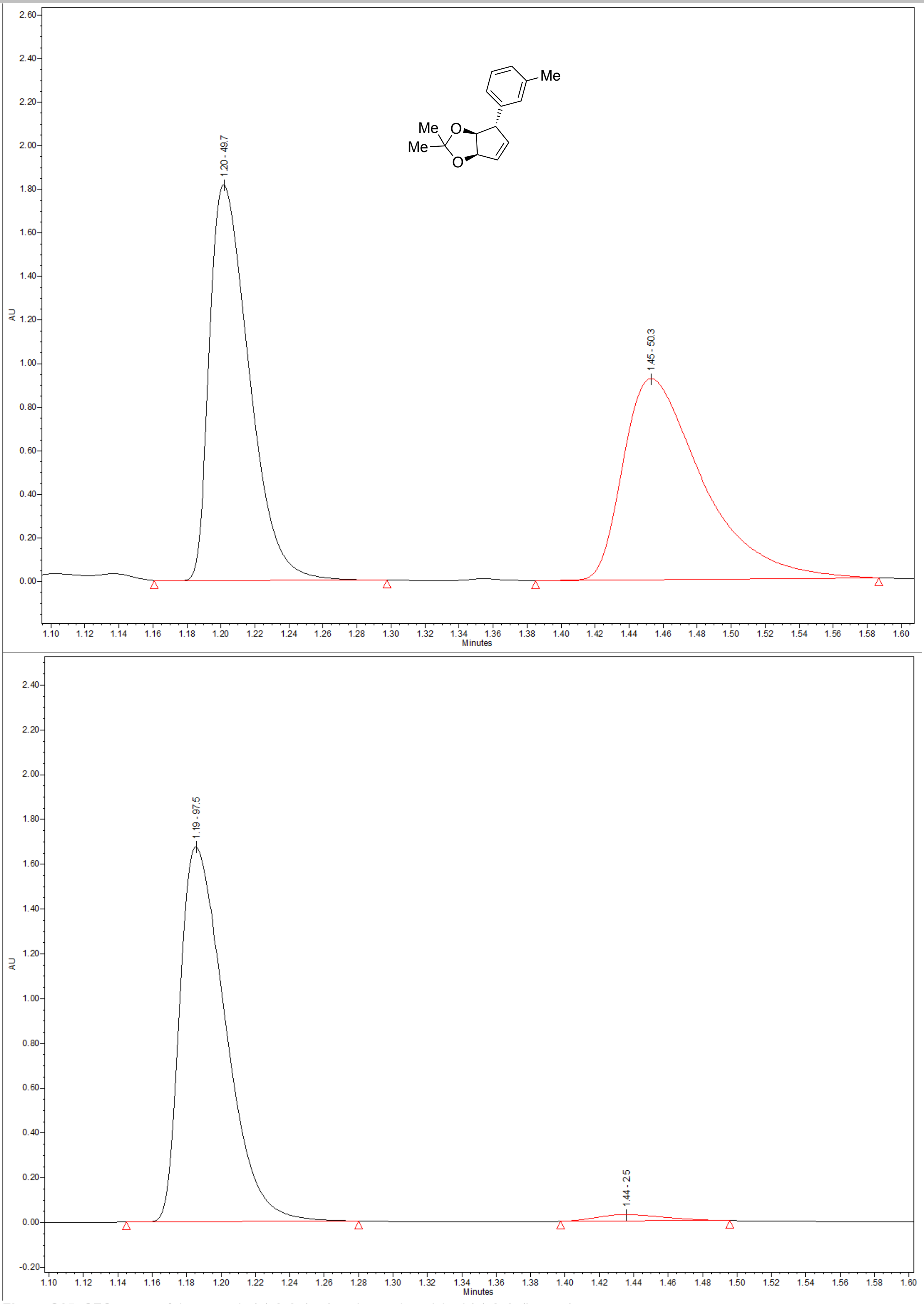

Figure S65: SFC traces of the racemic (±)-**3ab** (top) and enantioenriched (–)-**3ab** (bottom).

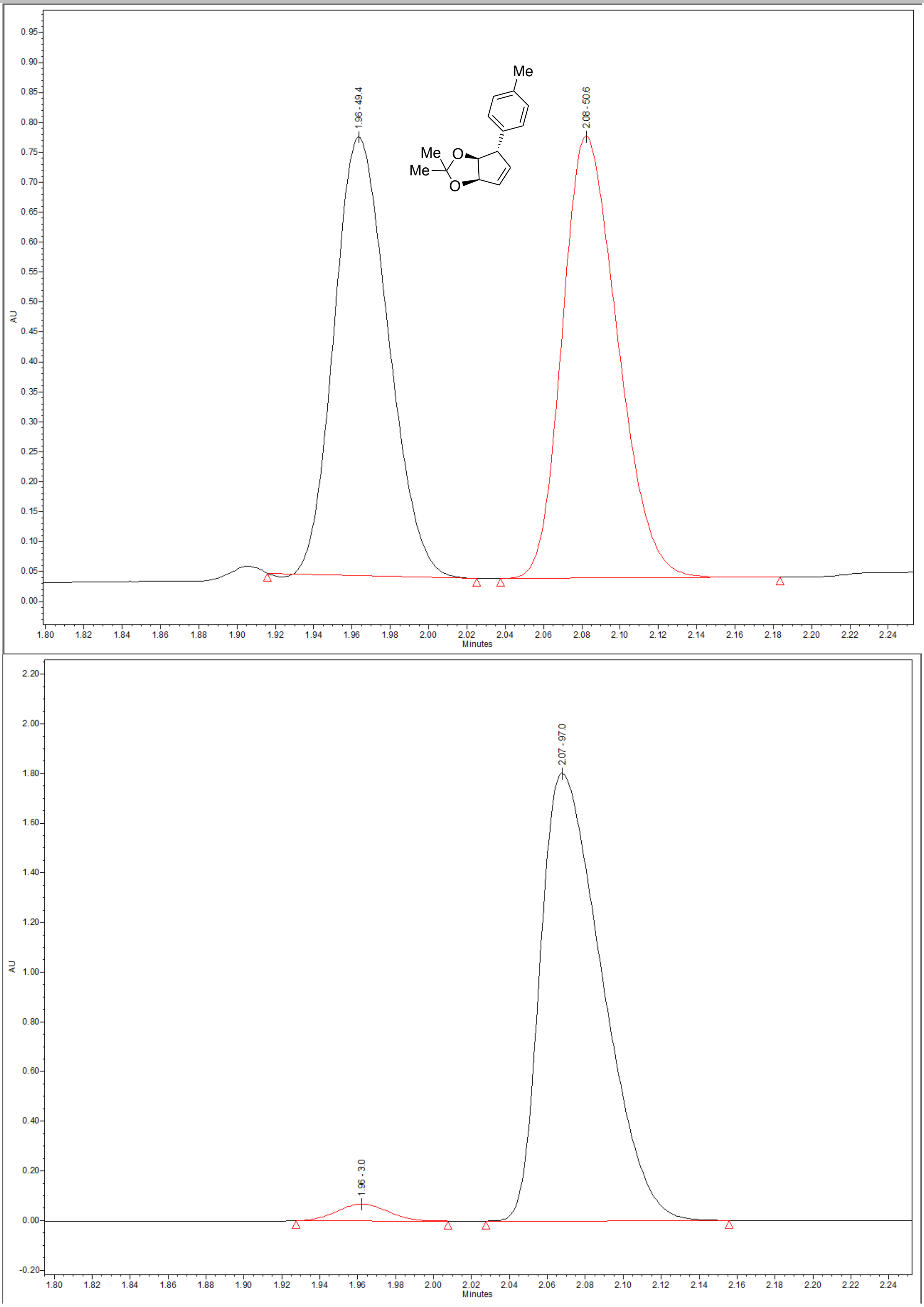

Figure S66: SFC traces of the racemic (±)-**3ac** (top) and enantioenriched (–)-**3ac** (bottom).

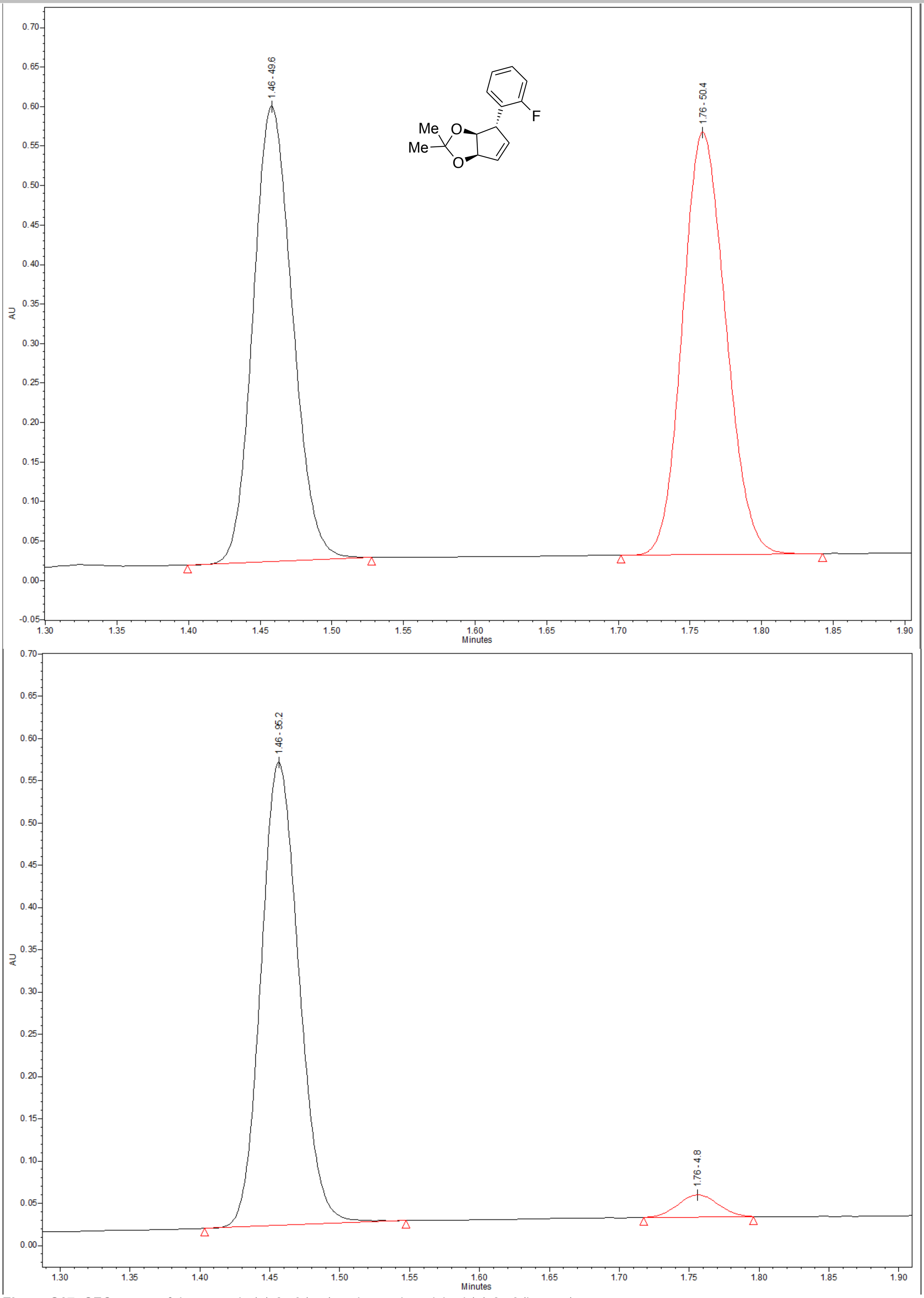

Figure S67: SFC traces of the racemic (±)-**3ad** (top) and enantioenriched (–)-**3ad** (bottom).

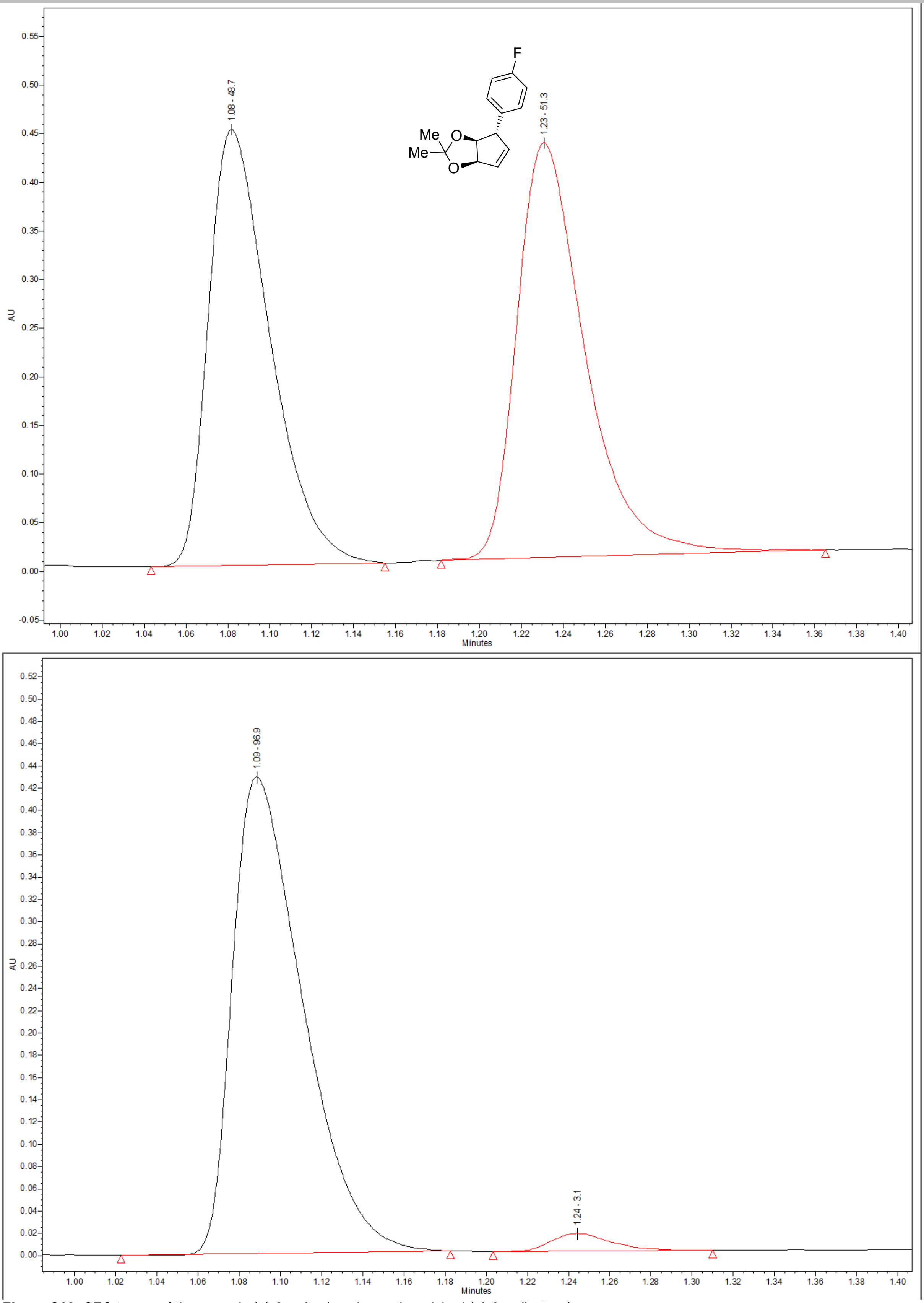

Figure S68: SFC traces of the racemic (±)-**3ae** (top) and enantioenriched (–)-**3ae** (bottom).

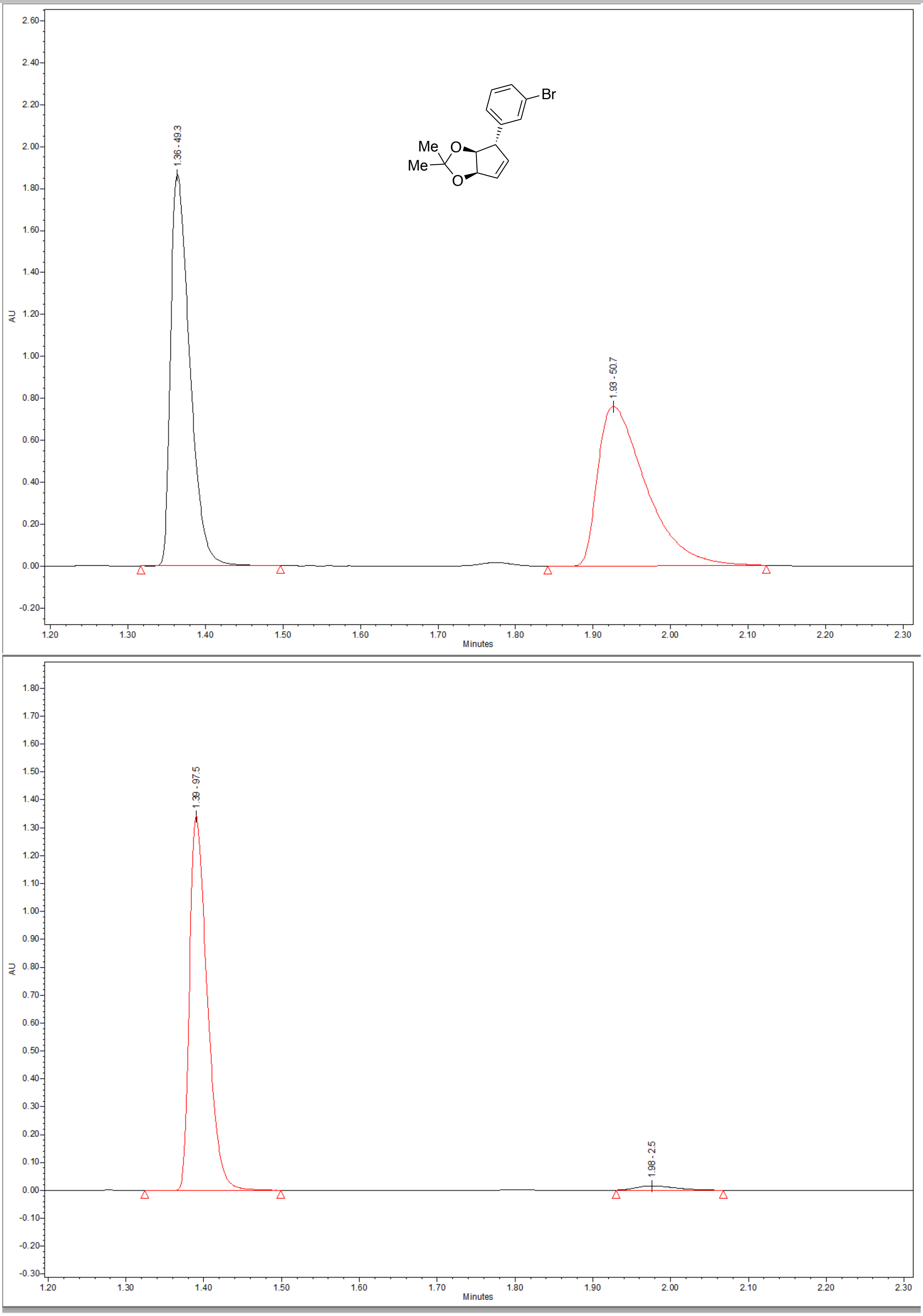

Figure S69: SFC traces of the racemic (±)-**3af** (top) and enantioenriched (–)-**3af** (bottom).

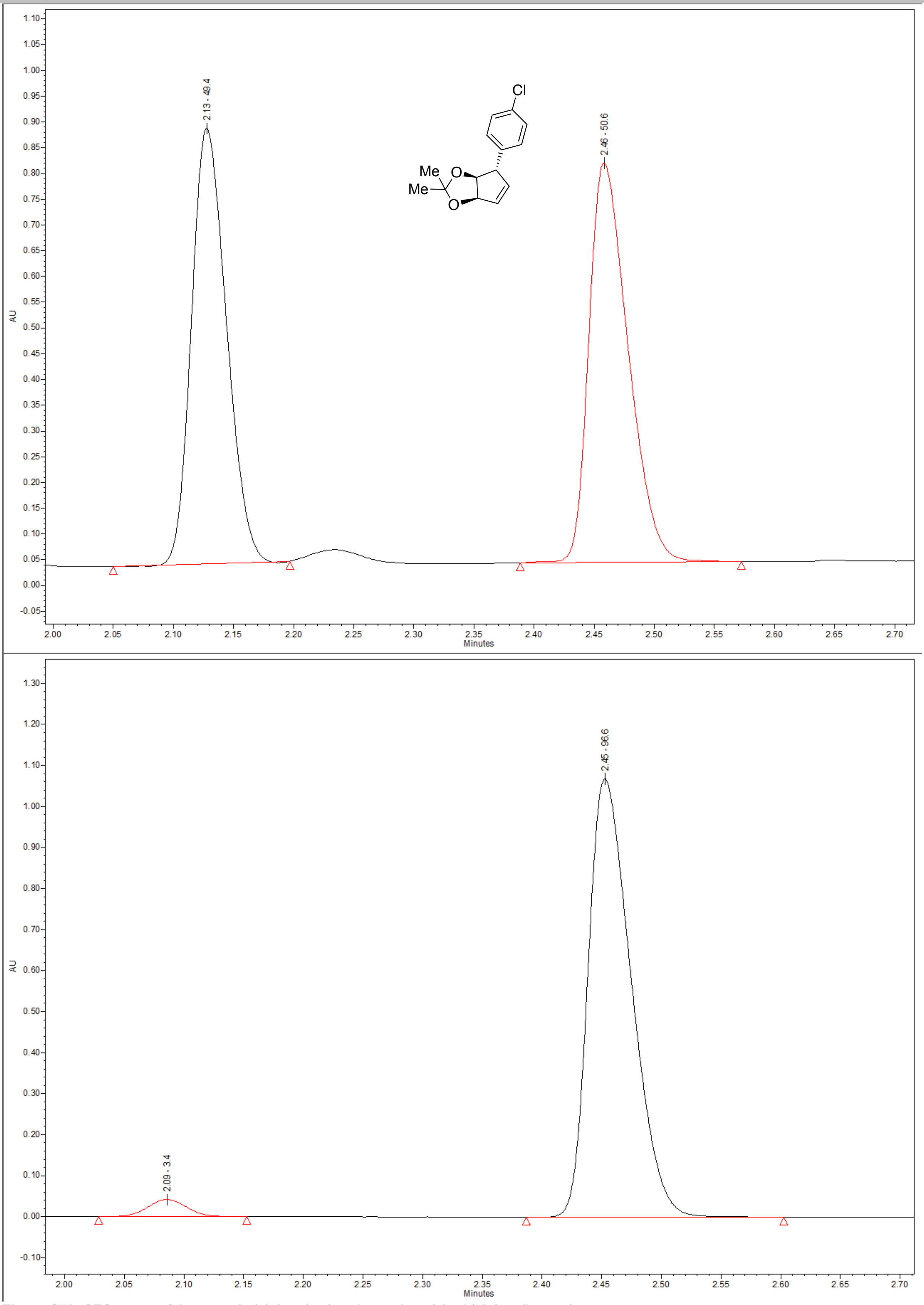

Figure S70: SFC traces of the racemic (±)-**3ag** (top) and enantioenriched (–)-**3ag** (bottom).

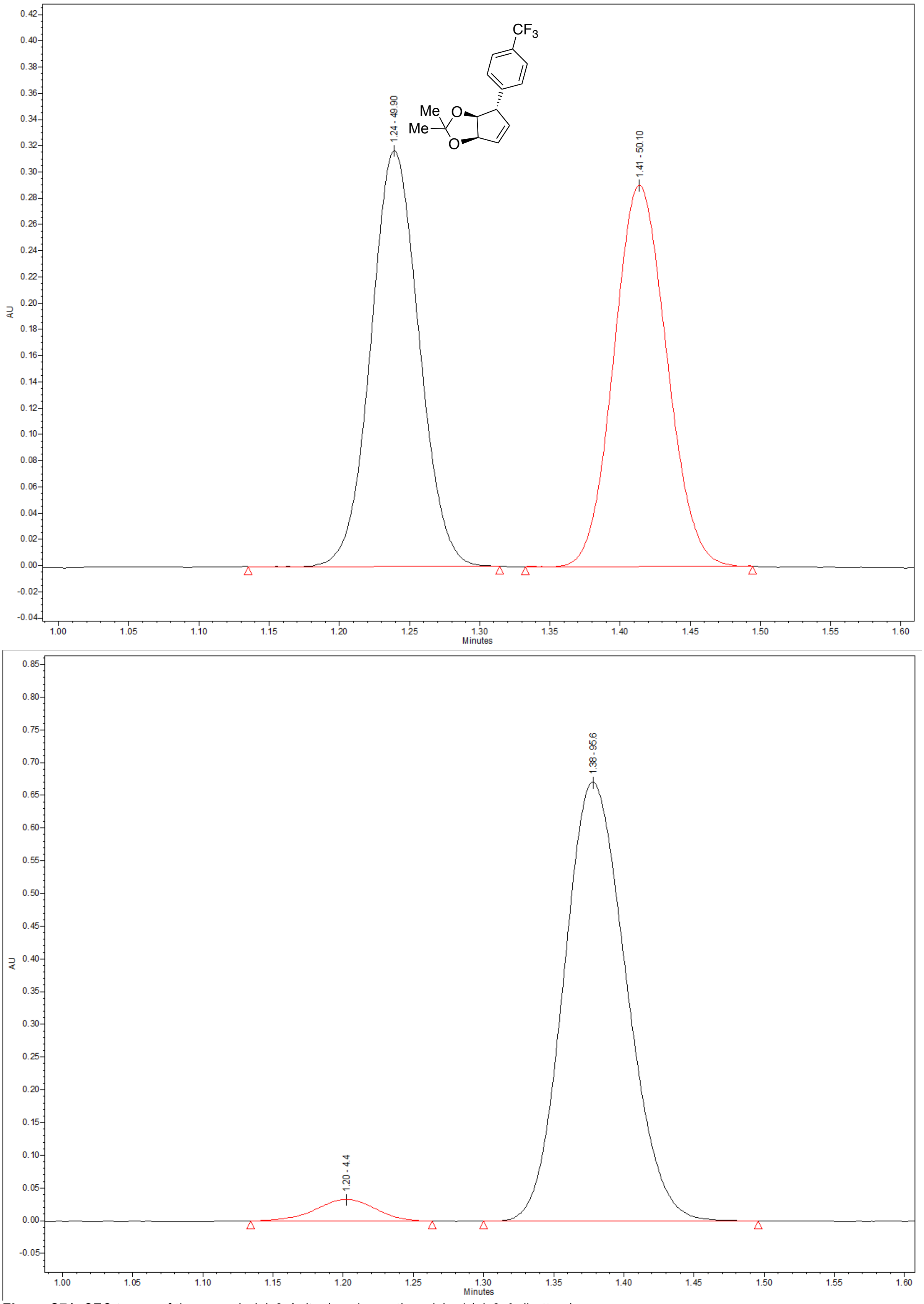

Figure S71: SFC traces of the racemic (±)-**3ah** (top) and enantioenriched (–)-**3ah** (bottom).

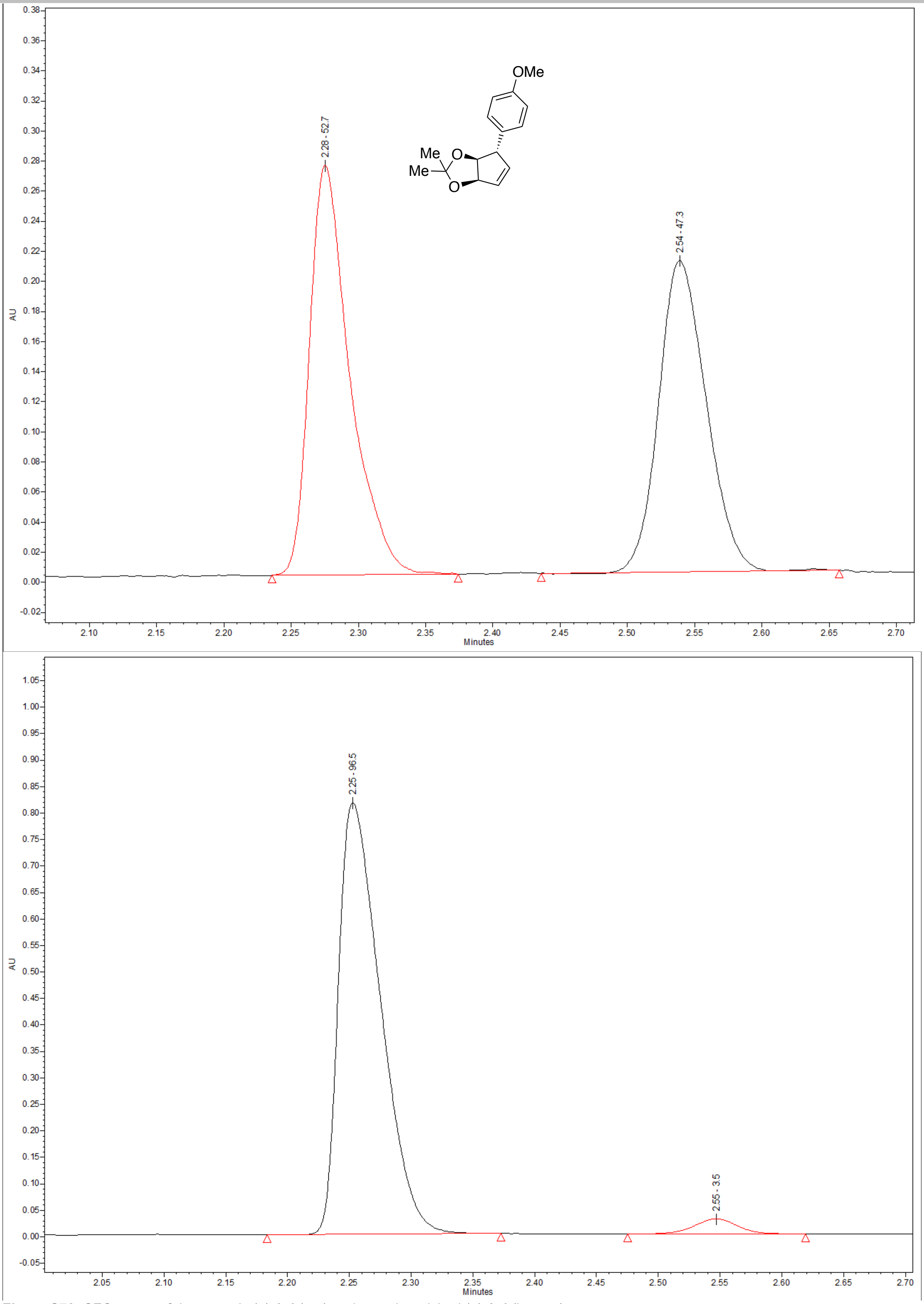

Figure S72: SFC traces of the racemic (±)-**3ai** (top) and enantioenriched (–)-**3ai** (bottom).

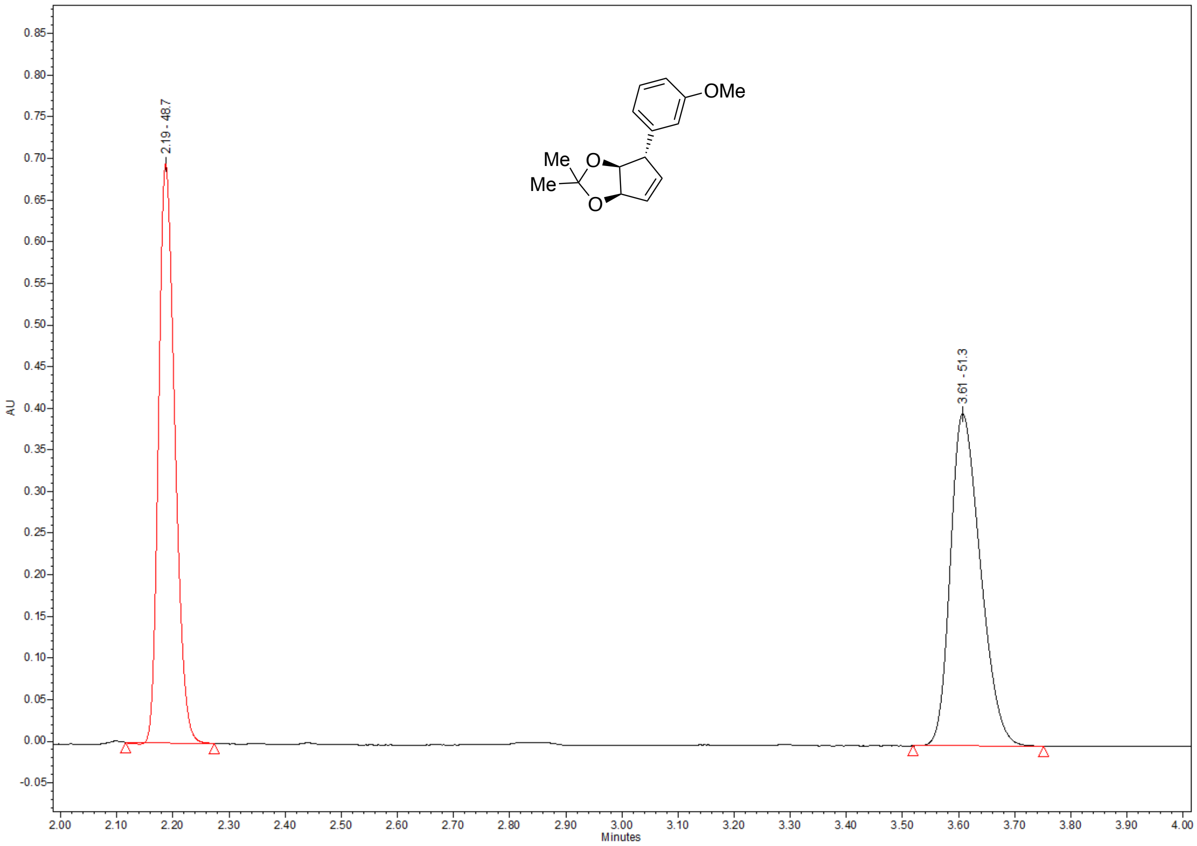

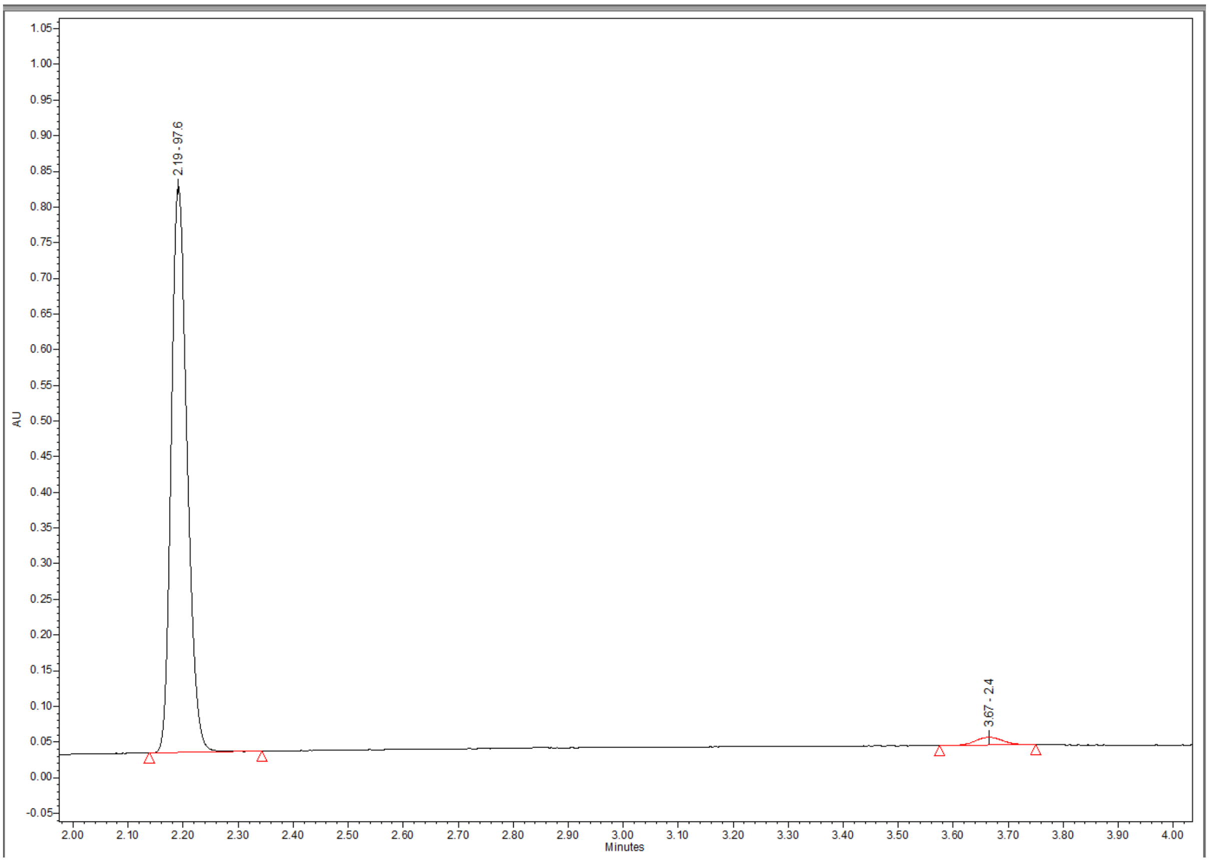

Figure S73: SFC traces of the racemic (±)-**3aj** (top) and enantioenriched (–)-**3aj** (bottom).

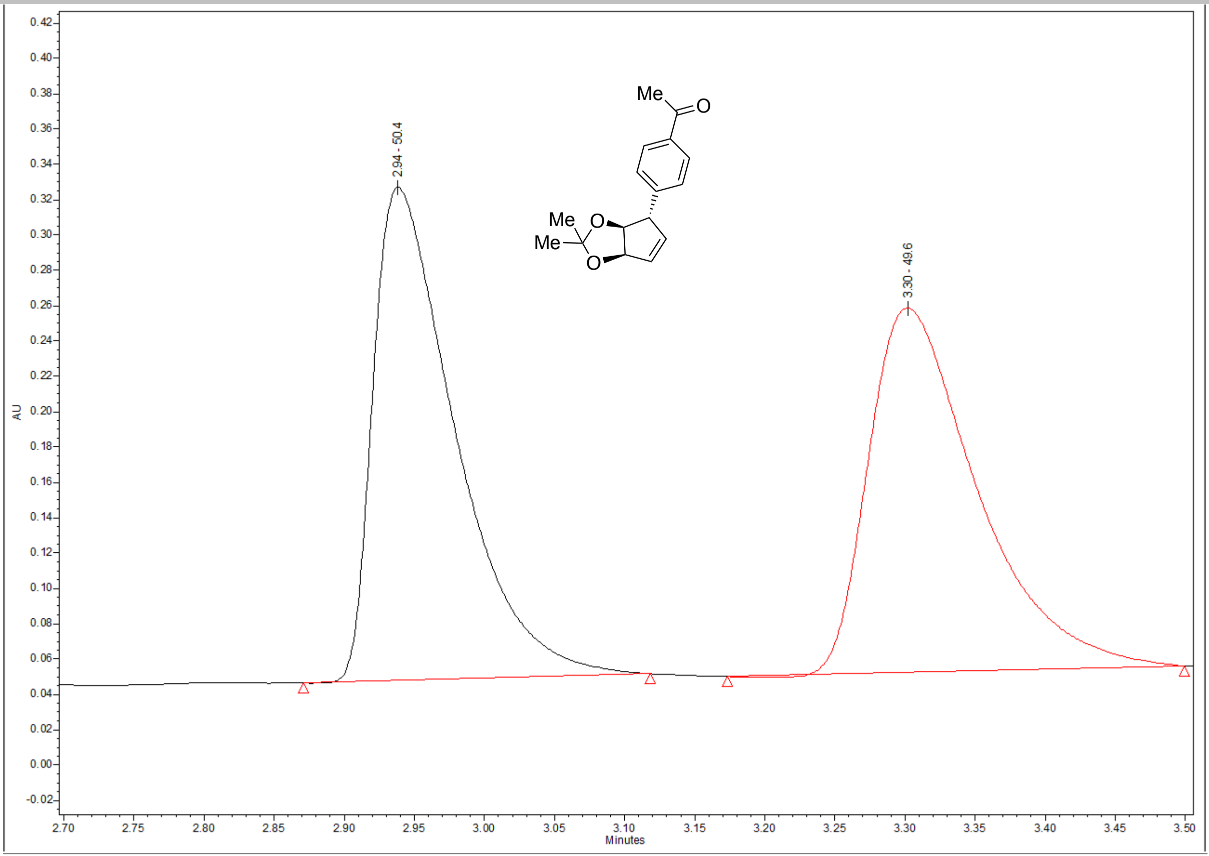

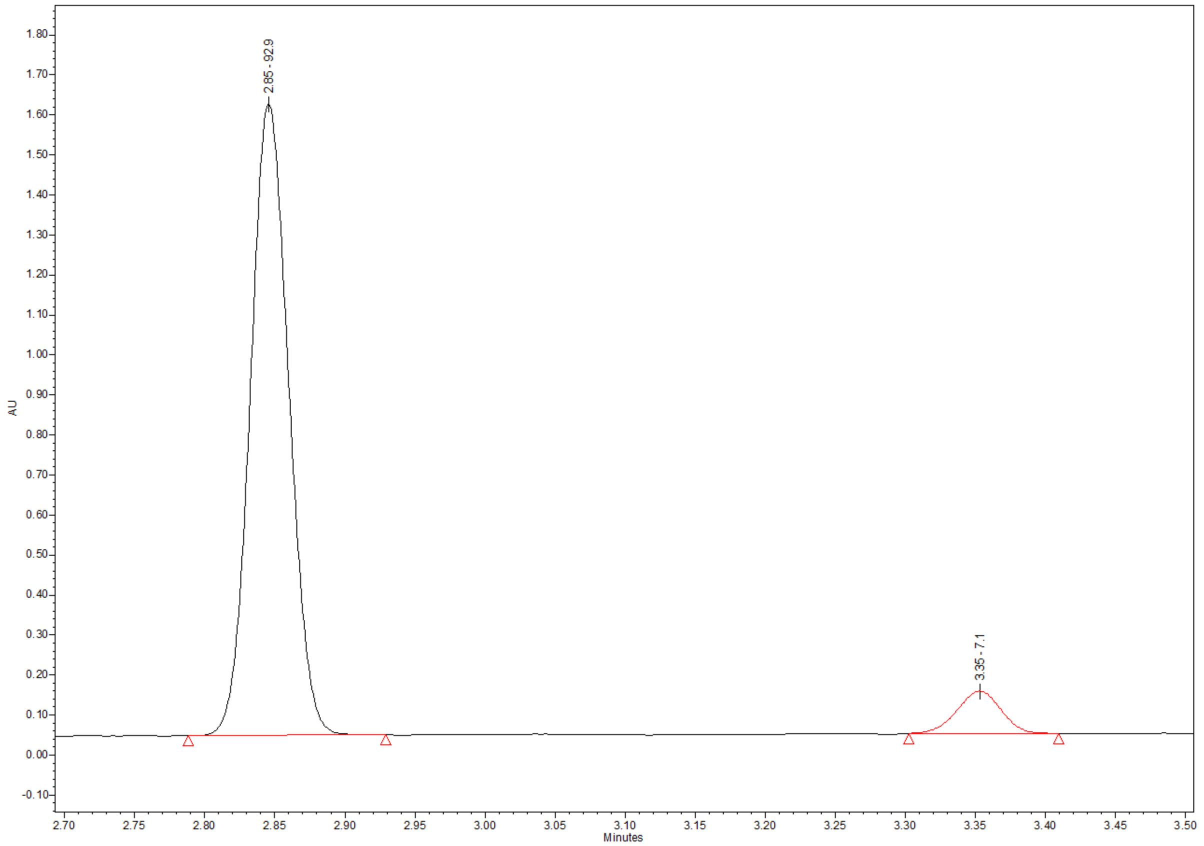

Figure S74: SFC traces of the racemic (±)-**3ak** (top) and enantioenriched (–)-**3ak** (bottom).

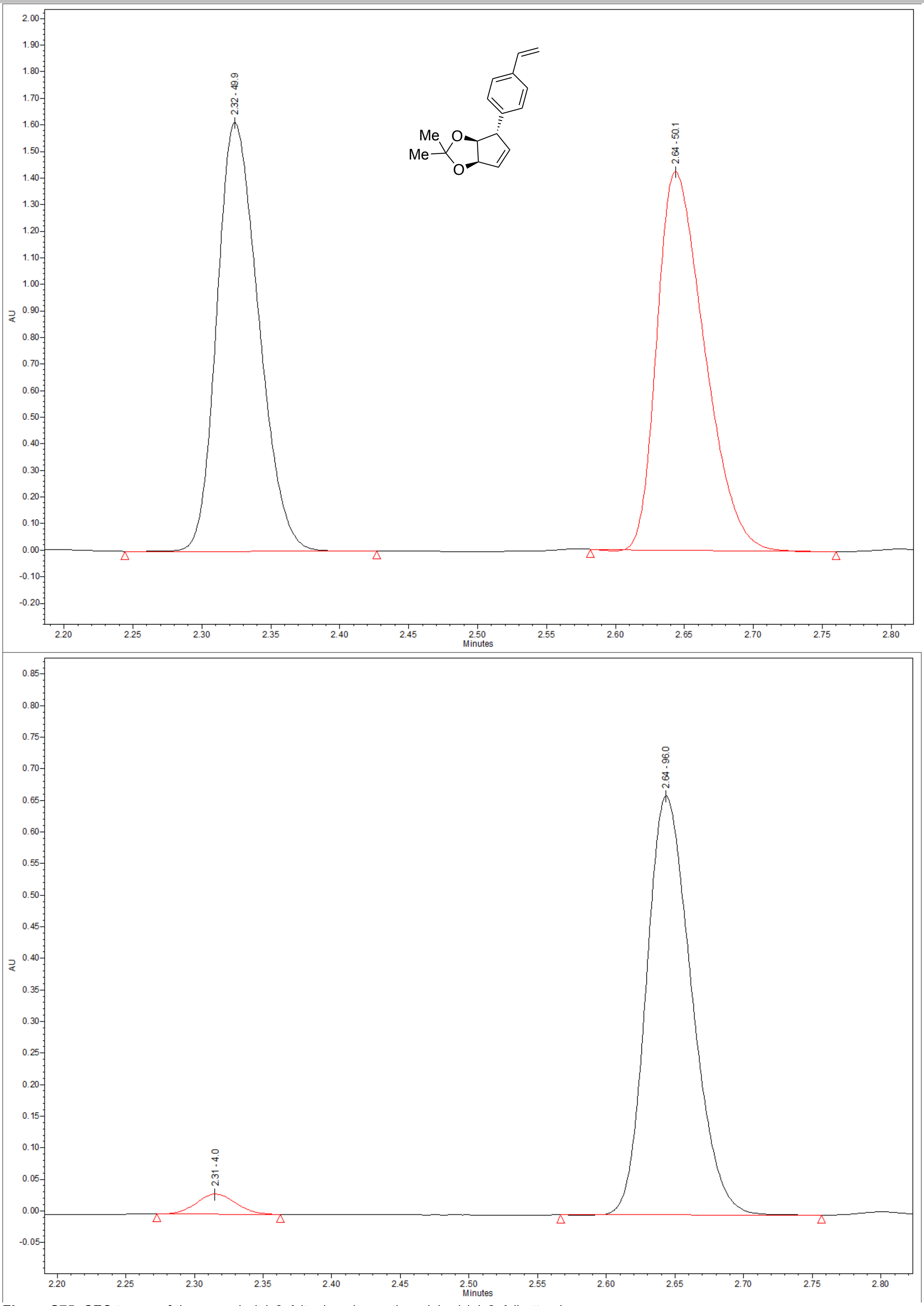

Figure S75: SFC traces of the racemic (±)-**3al** (top) and enantioenriched (–)-**3al** (bottom).

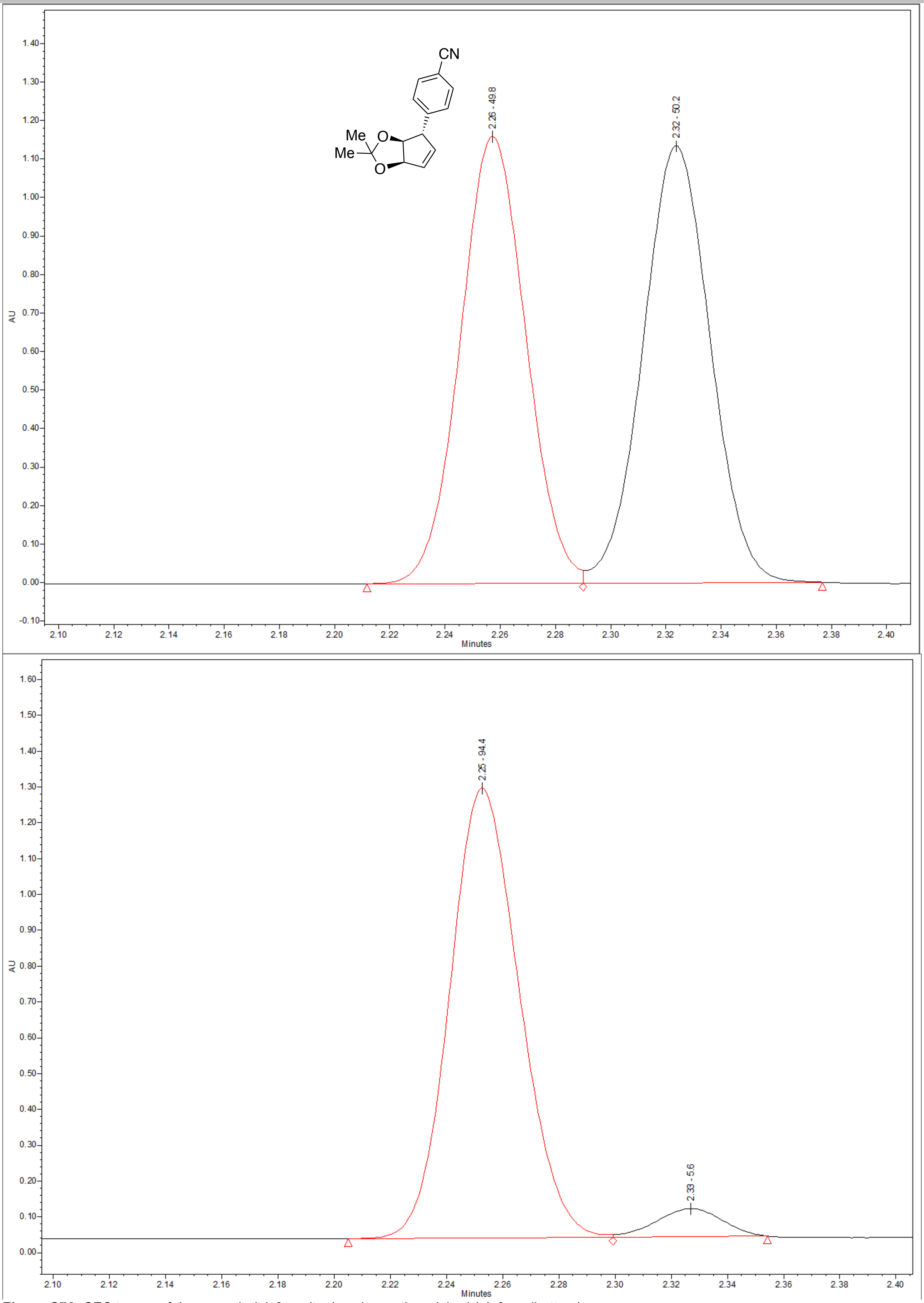

Figure S76: SFC traces of the racemic (±)-**3am** (top) and enantioenriched (–)-**3am** (bottom).

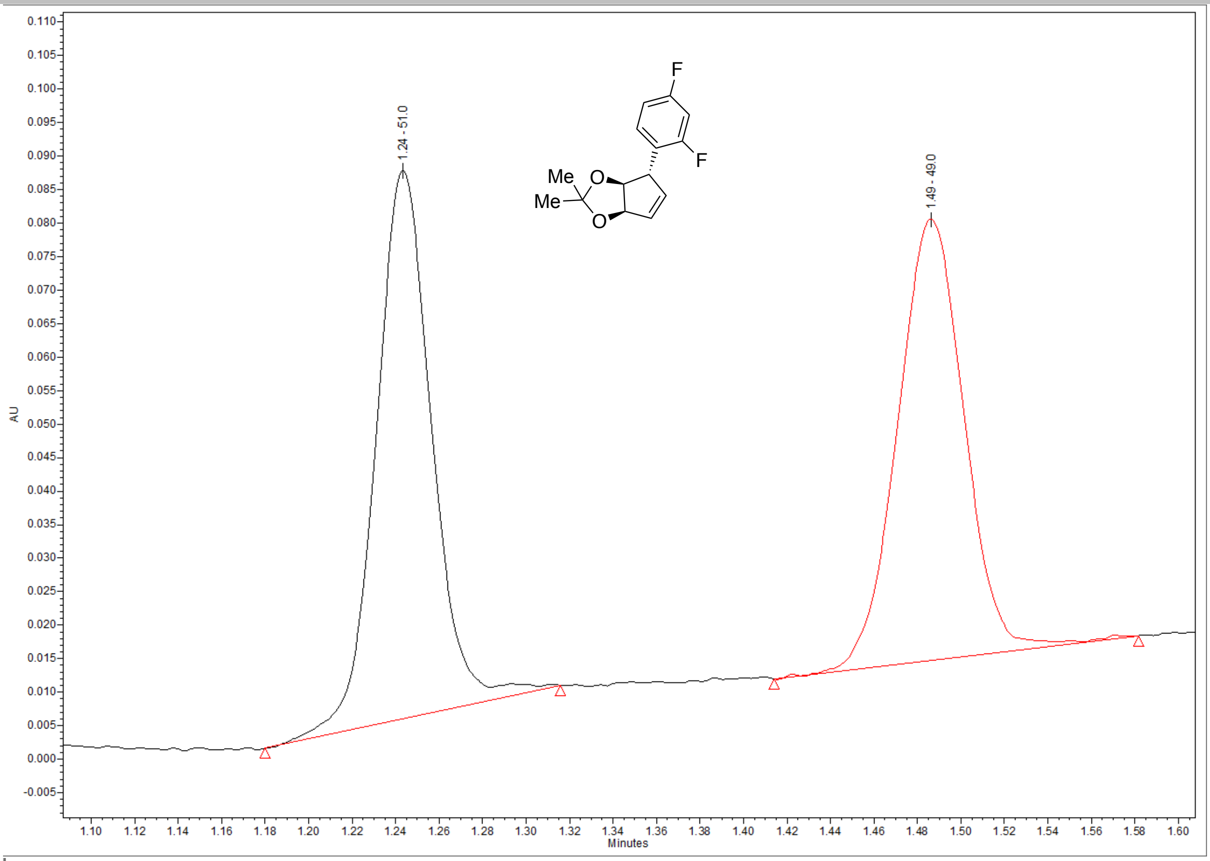

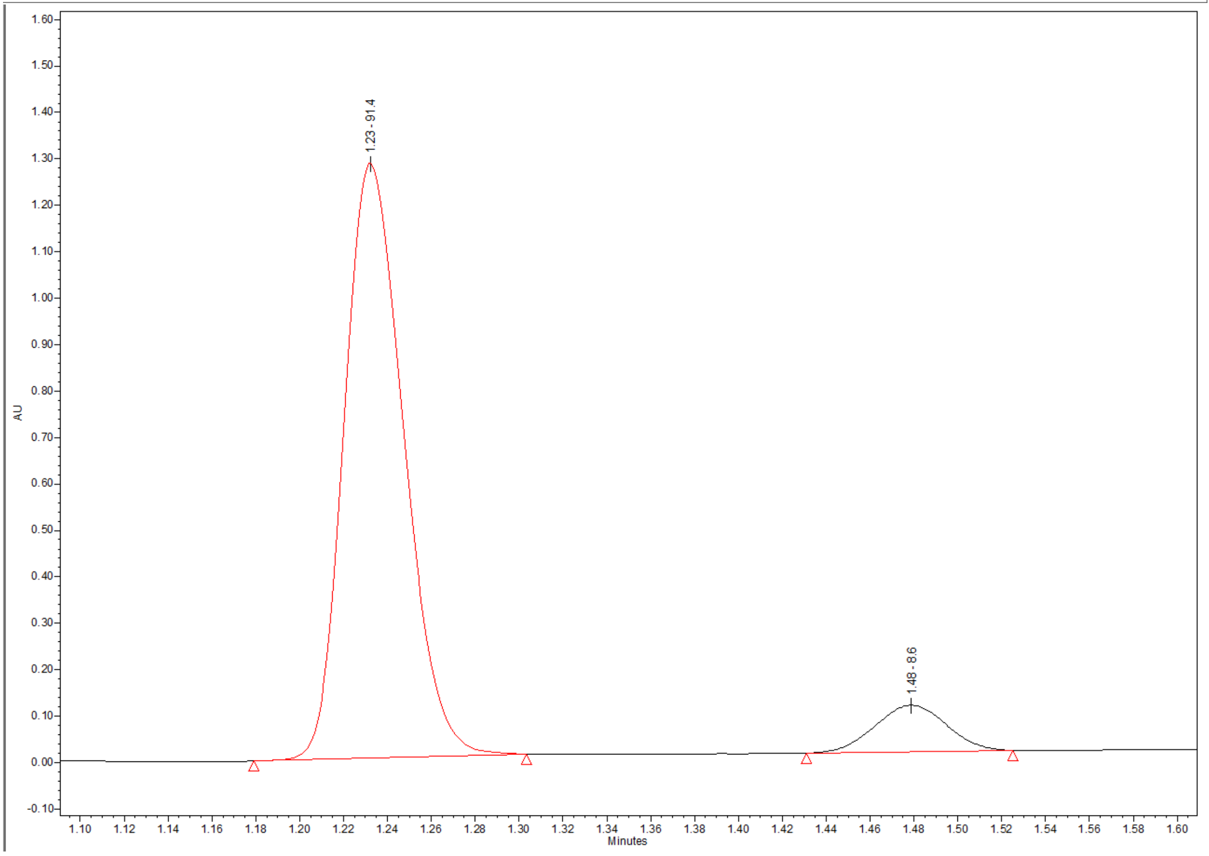

Figure S77: SFC traces of the racemic (±)-**3an** (top) and enantioenriched (–)-**3an** (bottom).

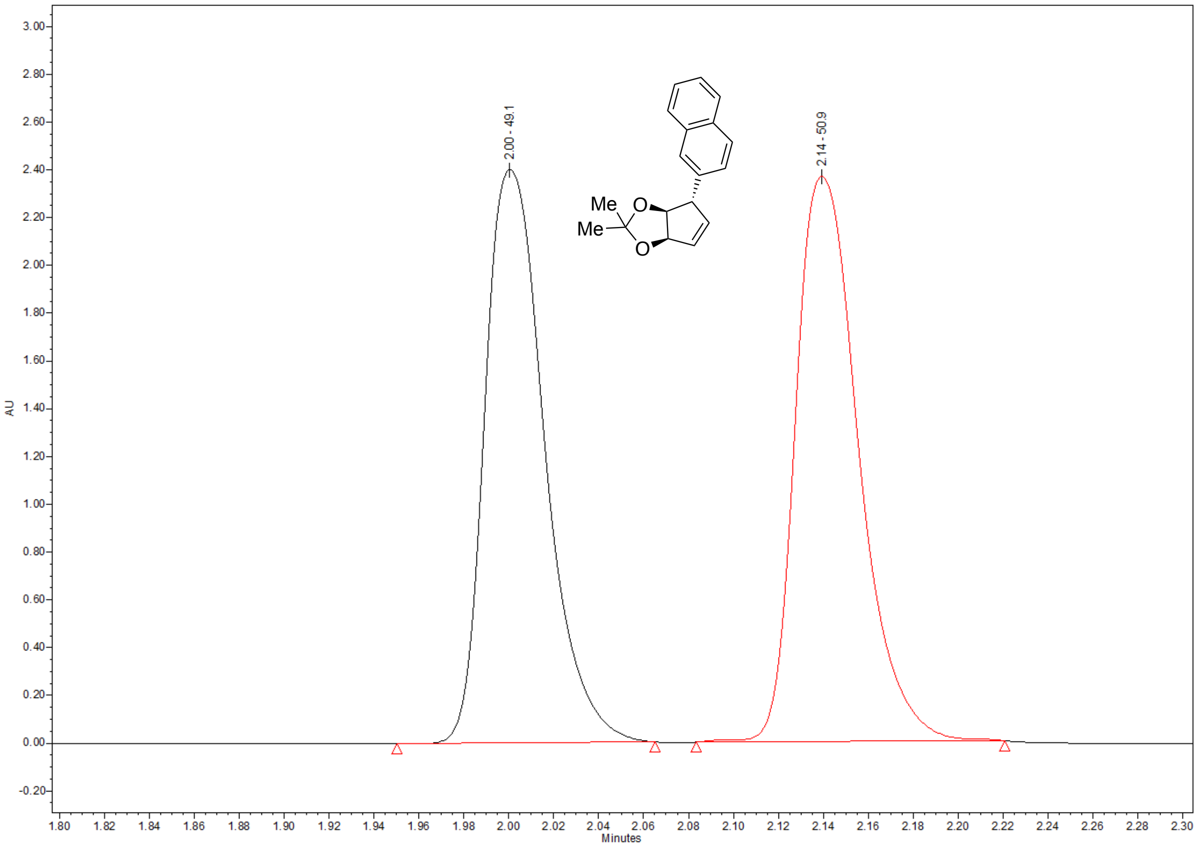

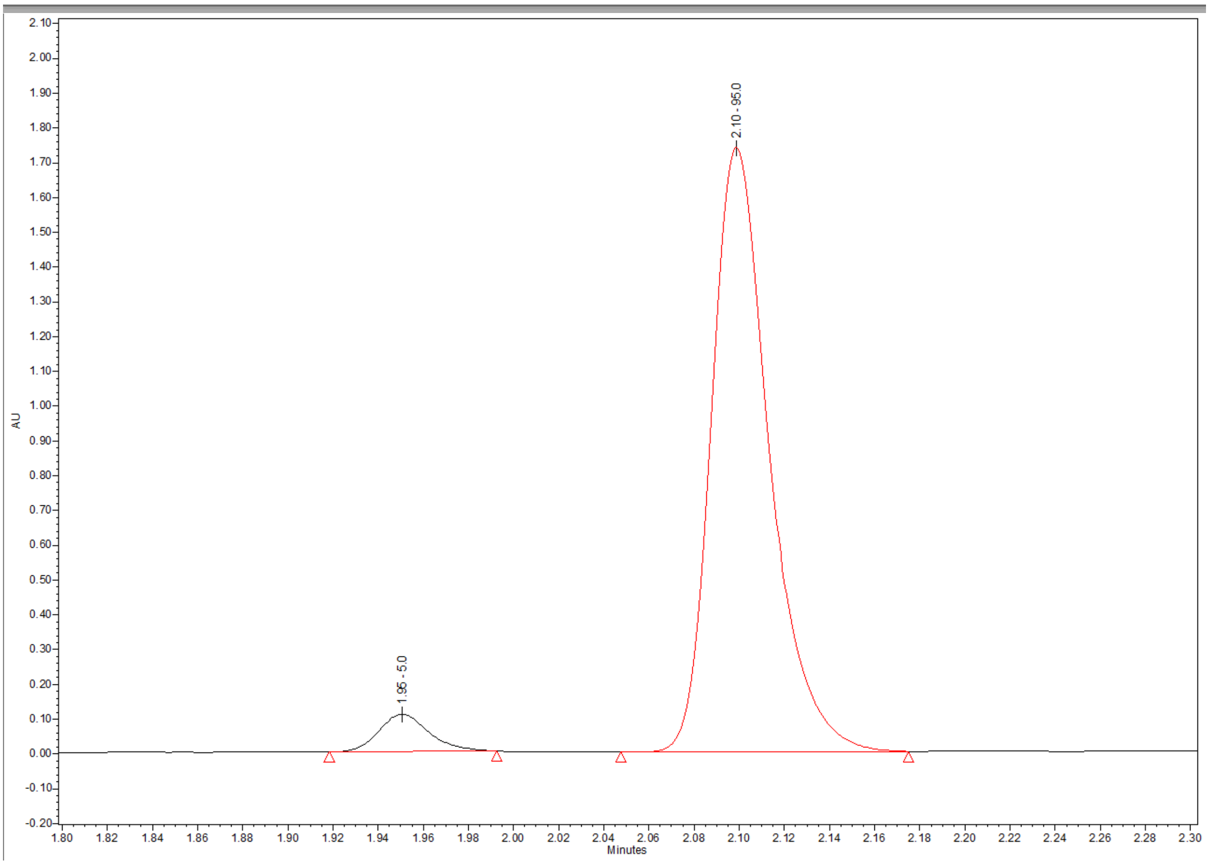

Figure S78: SFC traces of the racemic (±)-**3ao** (top) and enantioenriched (–)-**3ao** (bottom).

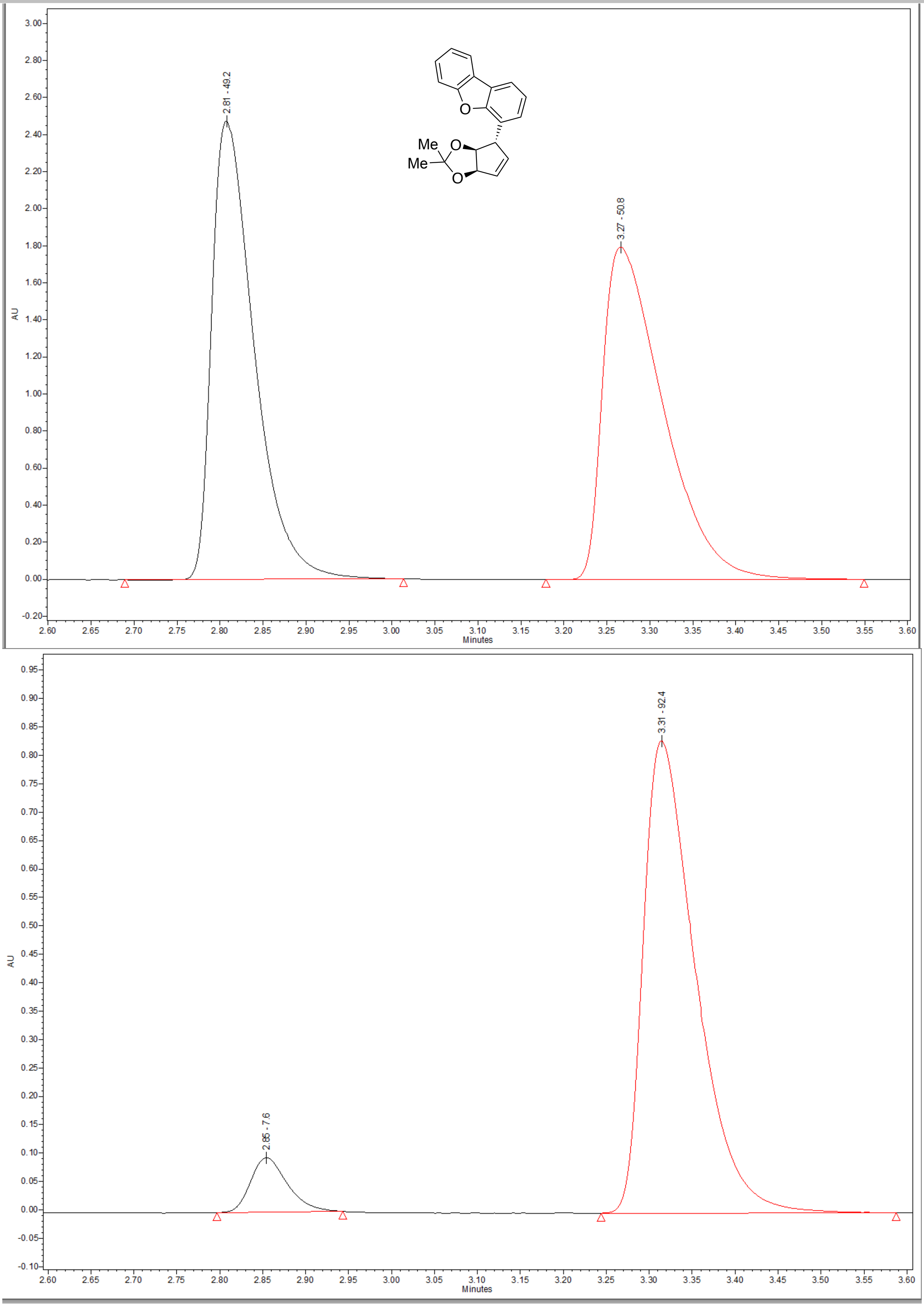

Figure S79: SFC traces of the racemic (±)-**3ap** (top) and enantioenriched (–)-**3ap** (bottom).

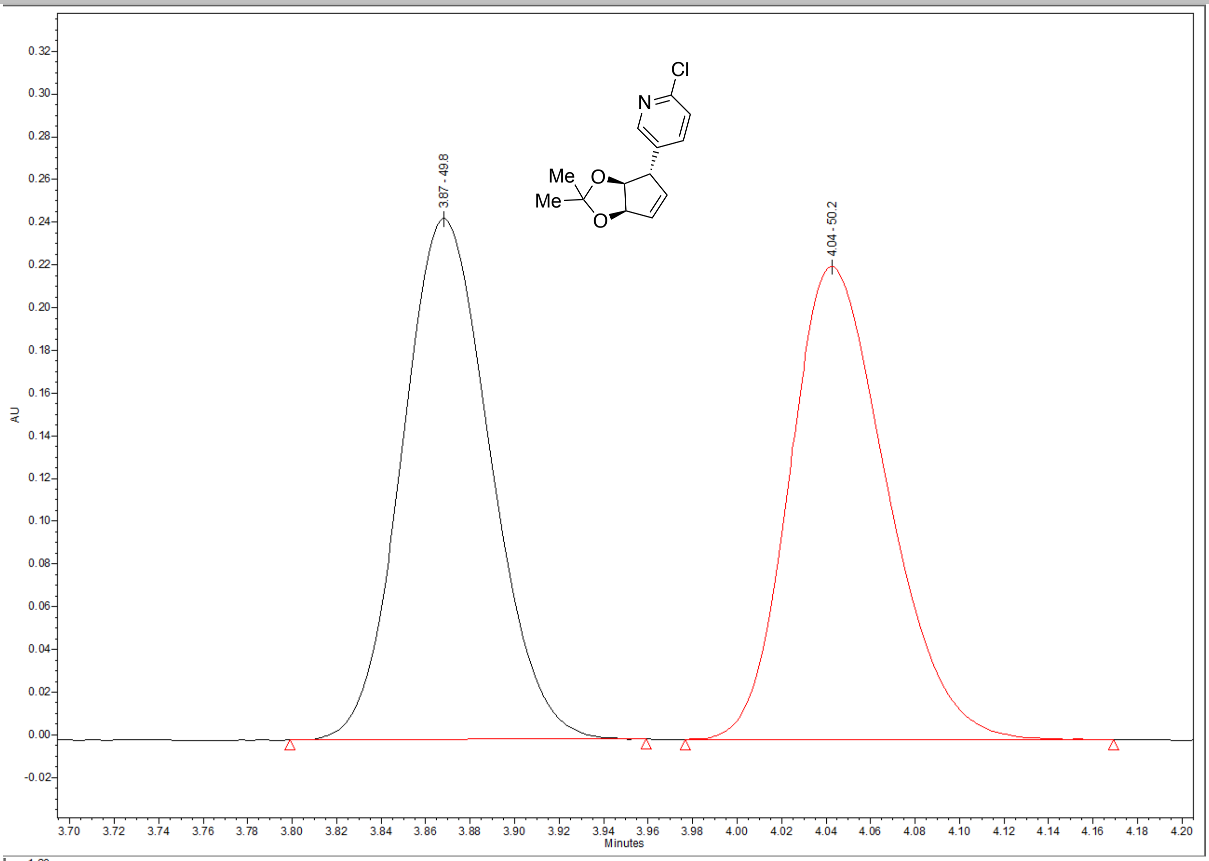

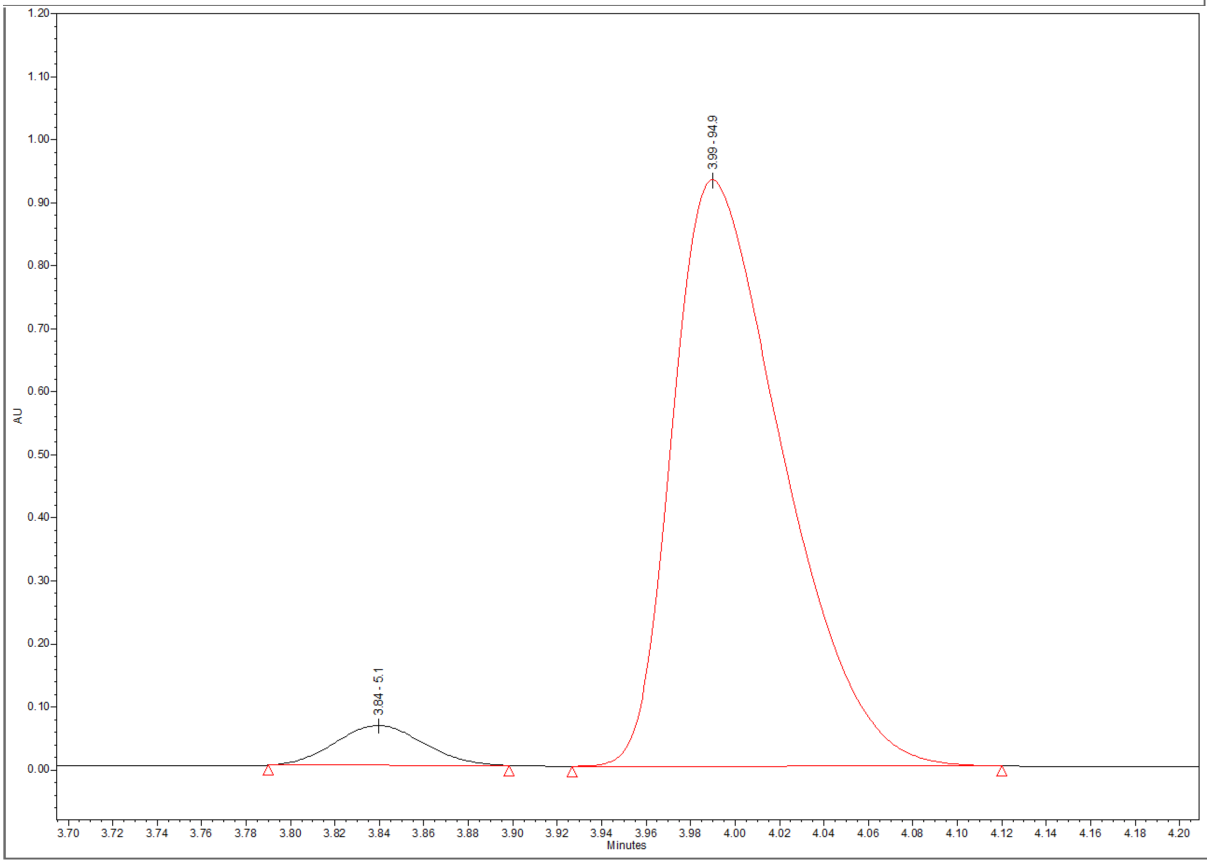

Figure S80: SFC traces of the racemic (±)-**3aq** (top) and enantioenriched (–)-**3aq** (bottom).

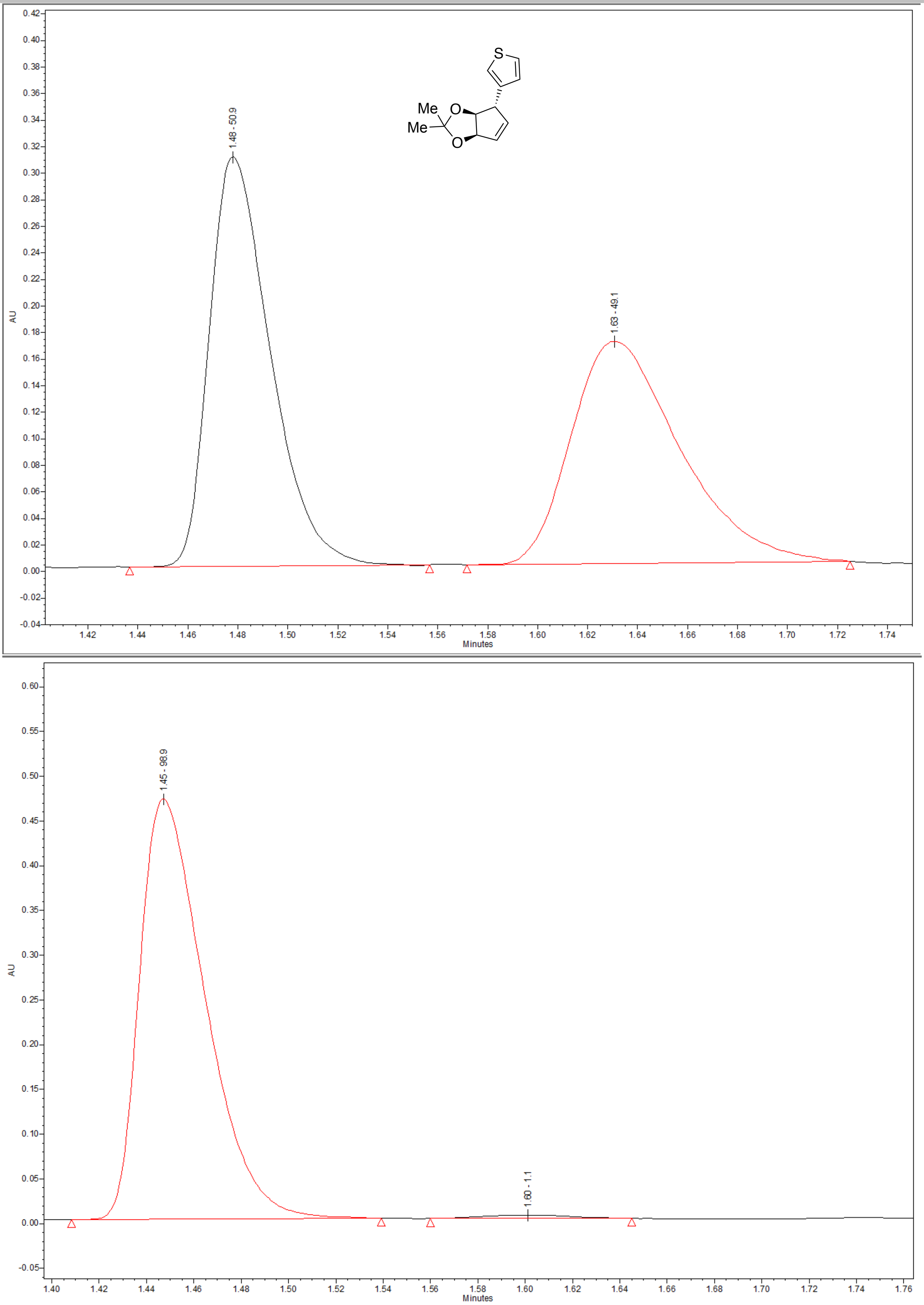

Figure S81: SFC traces of the racemic (±)-**3ar** (top) and enantioenriched (–)-**3ar** (bottom).

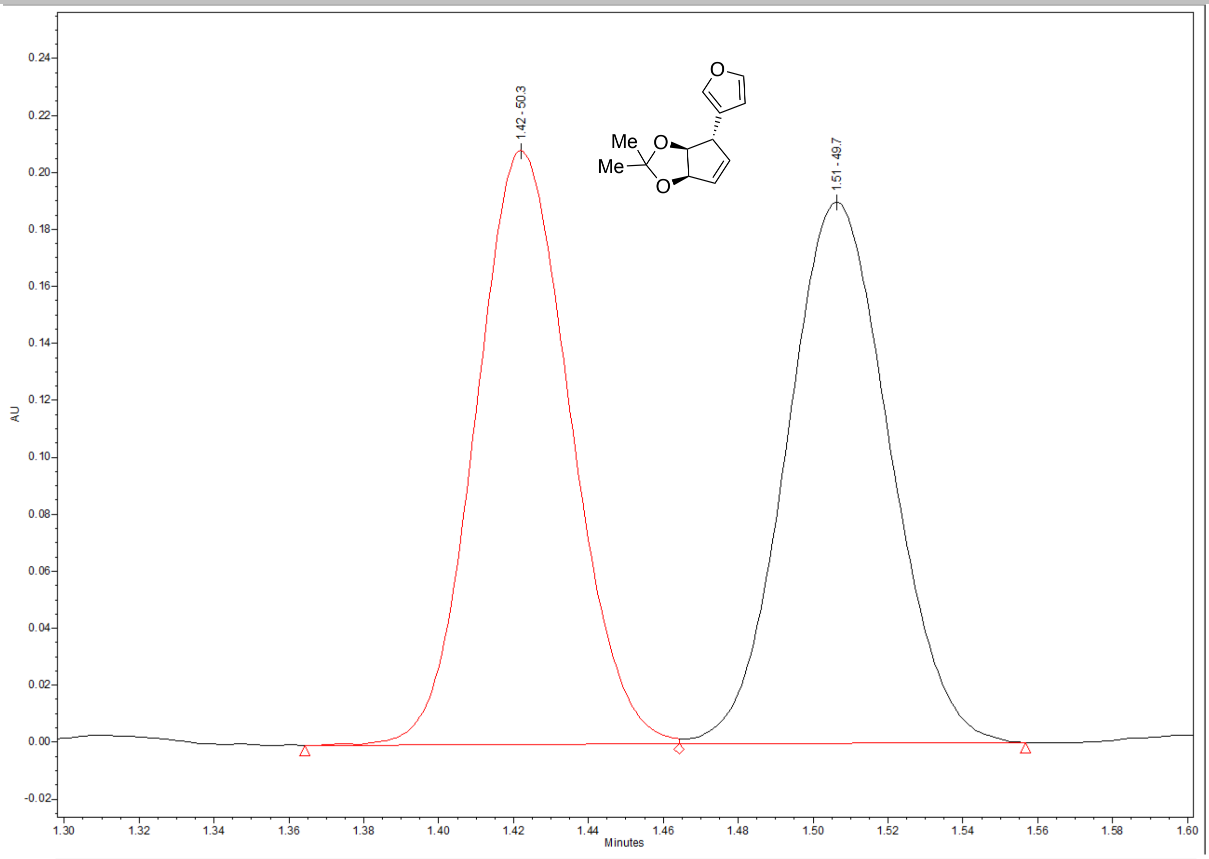

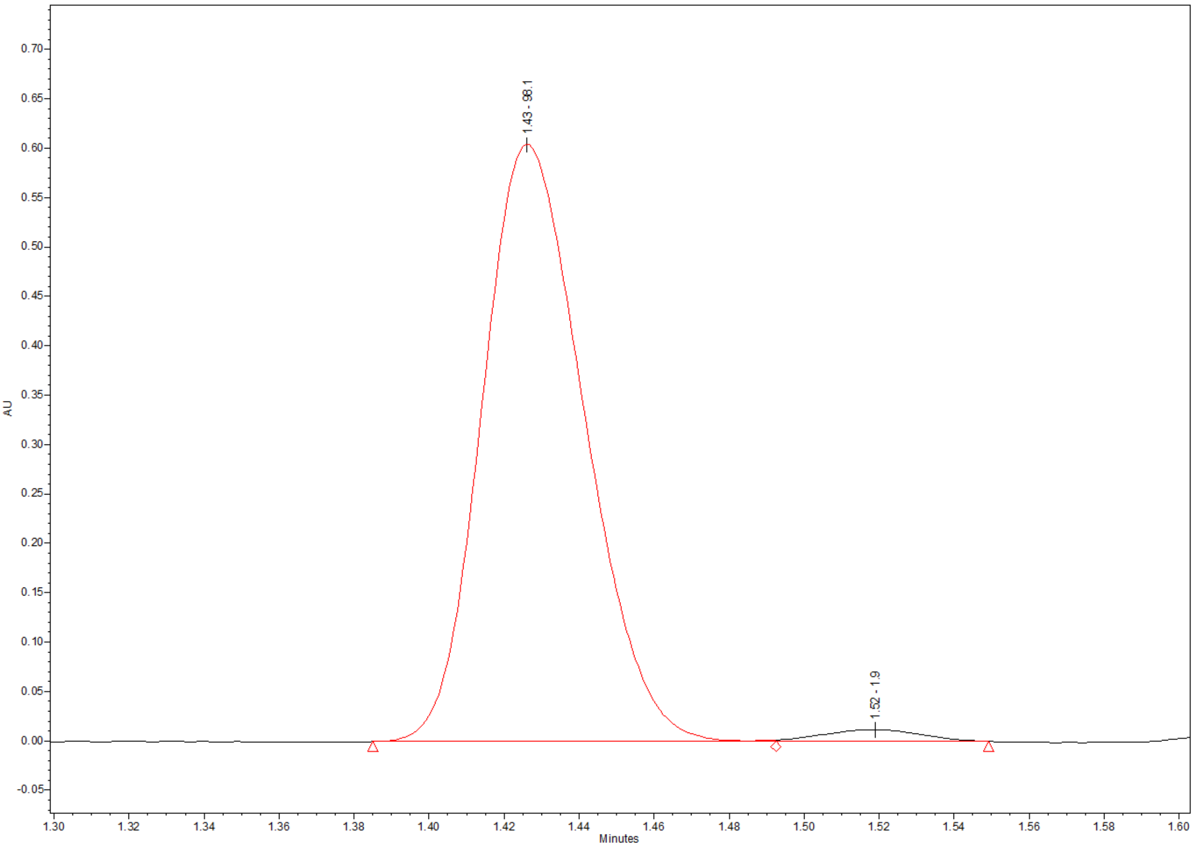

Figure S82: SFC traces of the racemic (±)-**3as** (top) and enantioenriched (–)-**3as** (bottom).

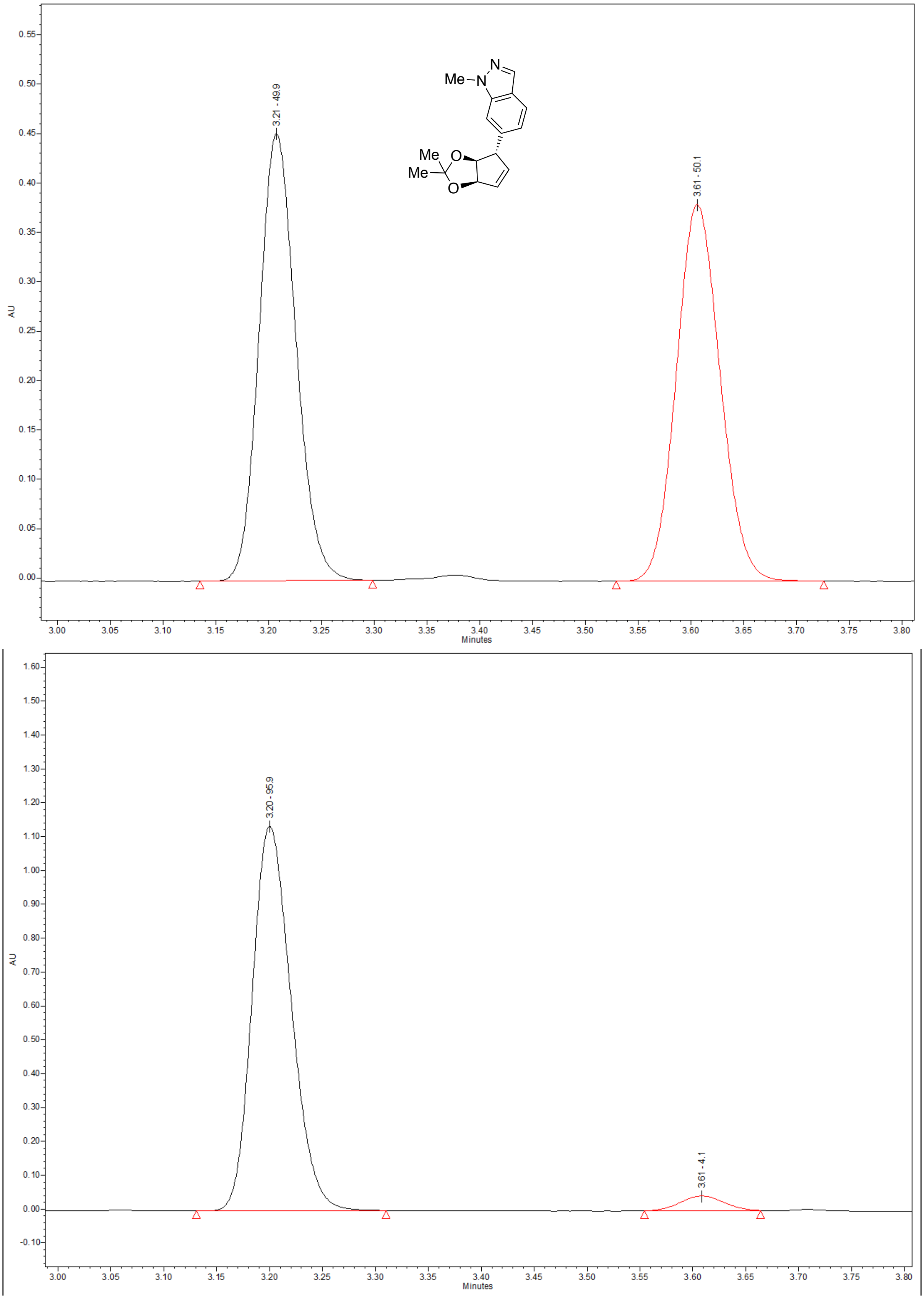

Figure S83: SFC traces of the racemic (±)-**3at** (top) and enantioenriched (–)-**3at** (bottom).

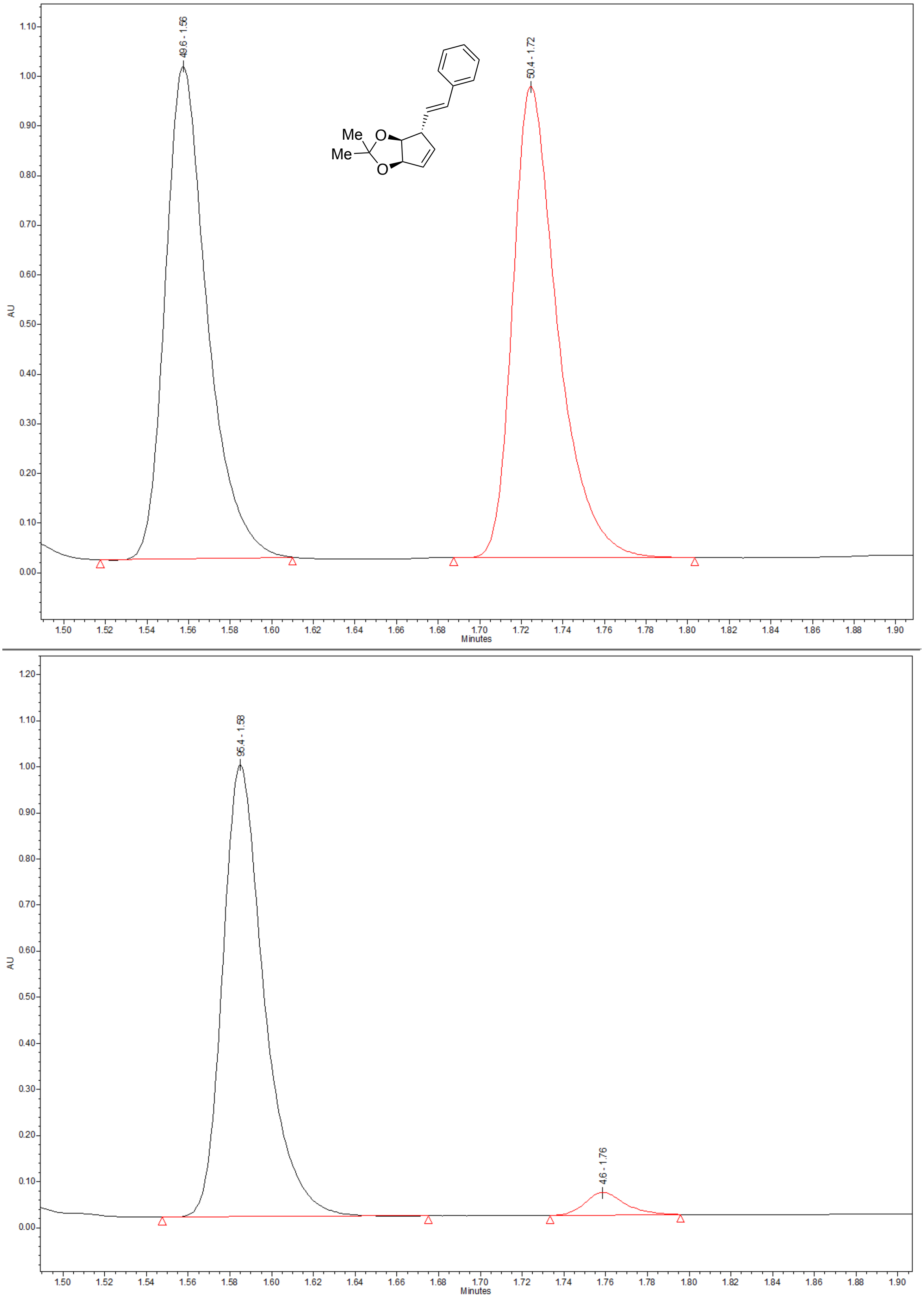

Figure S84: SFC traces of the racemic (±)-**3au** (top) and enantioenriched (–)-**3au** (bottom).

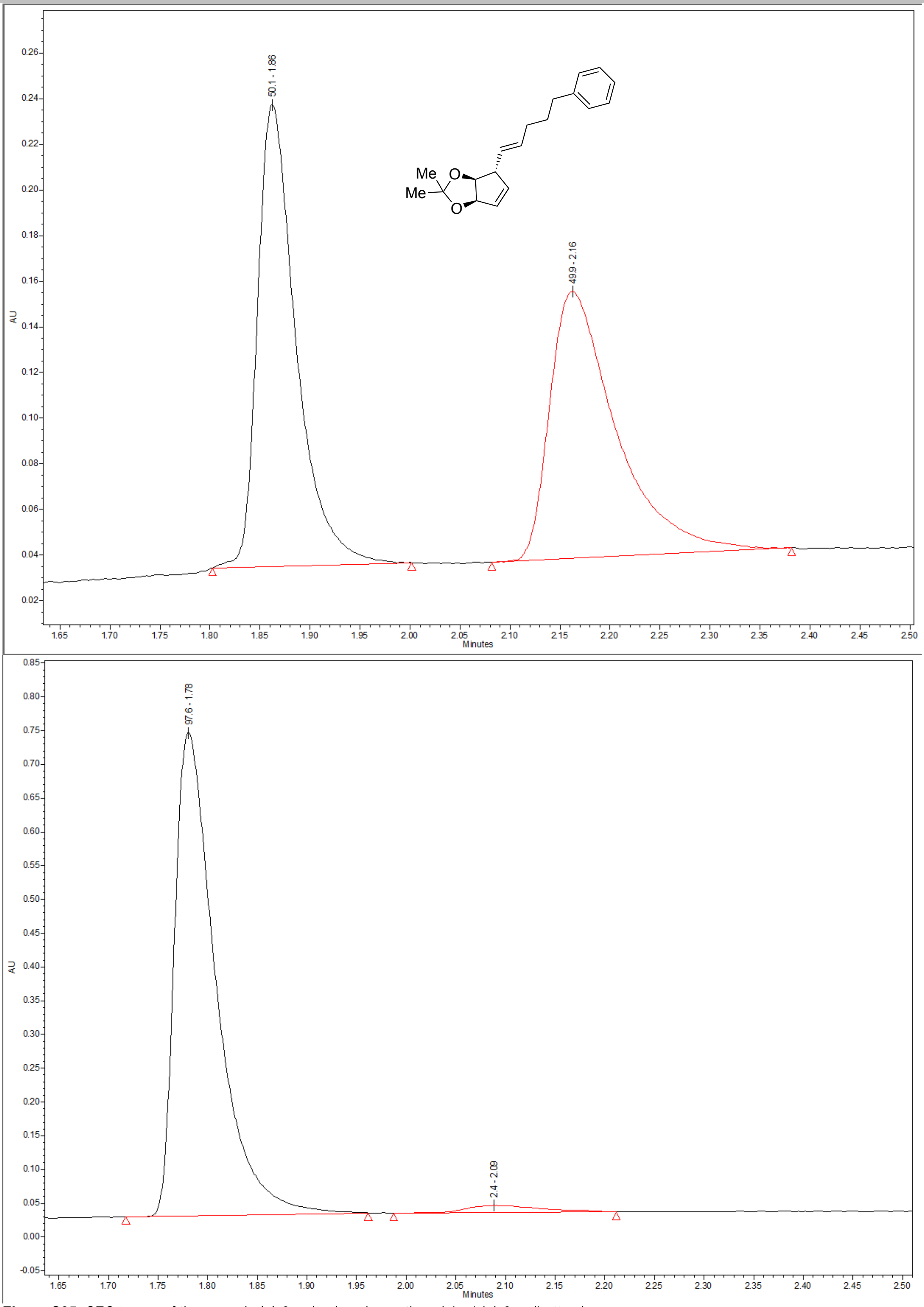

Figure S85: SFC traces of the racemic (±)-**3av** (top) and enantioenriched (–)-**3av** (bottom).

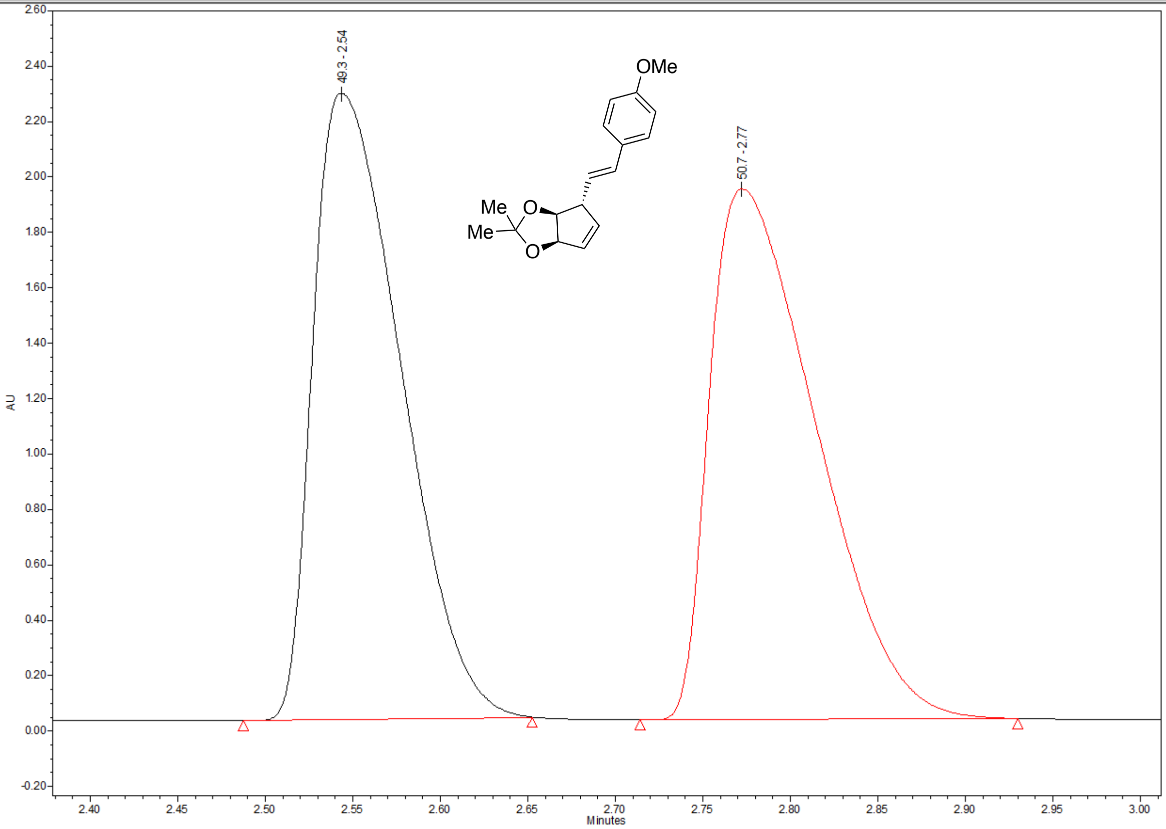

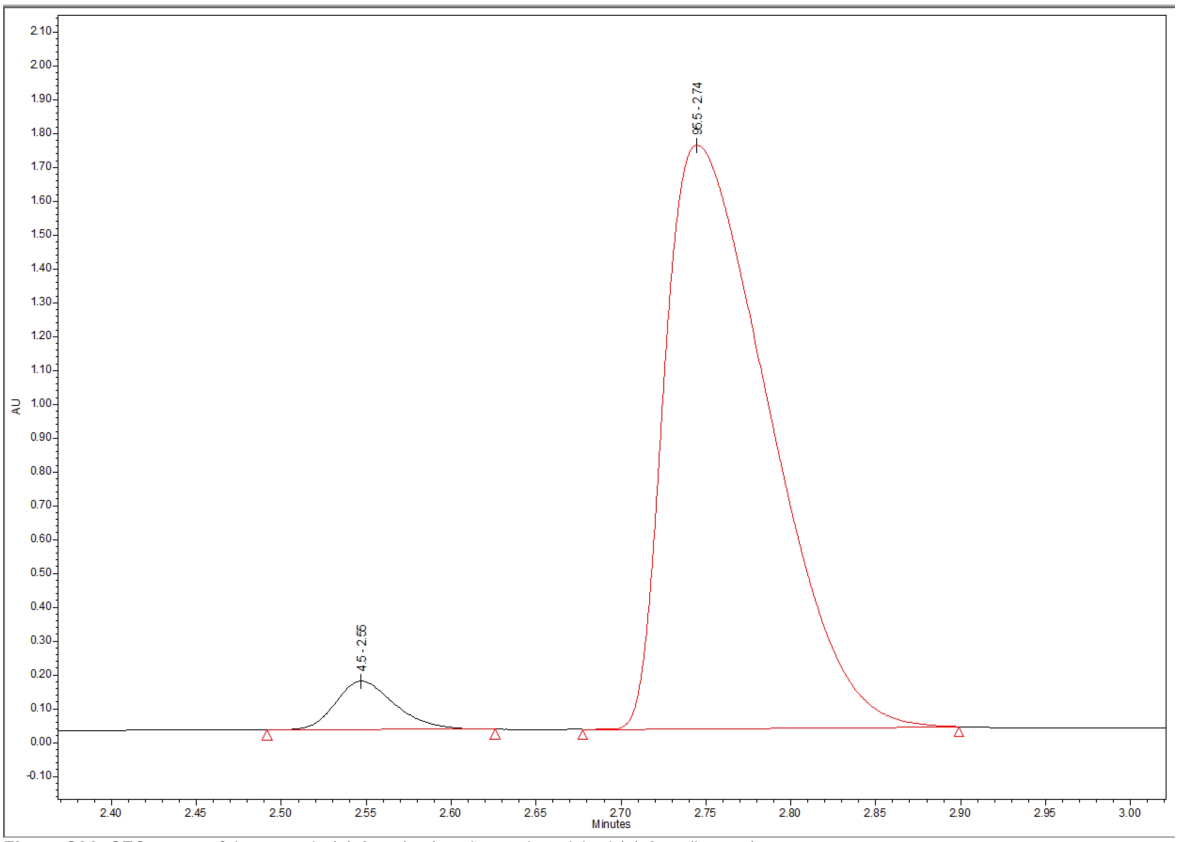

Figure S86: SFC traces of the racemic (±)-**3aw** (top) and enantioenriched (–)-**3aw** (bottom).

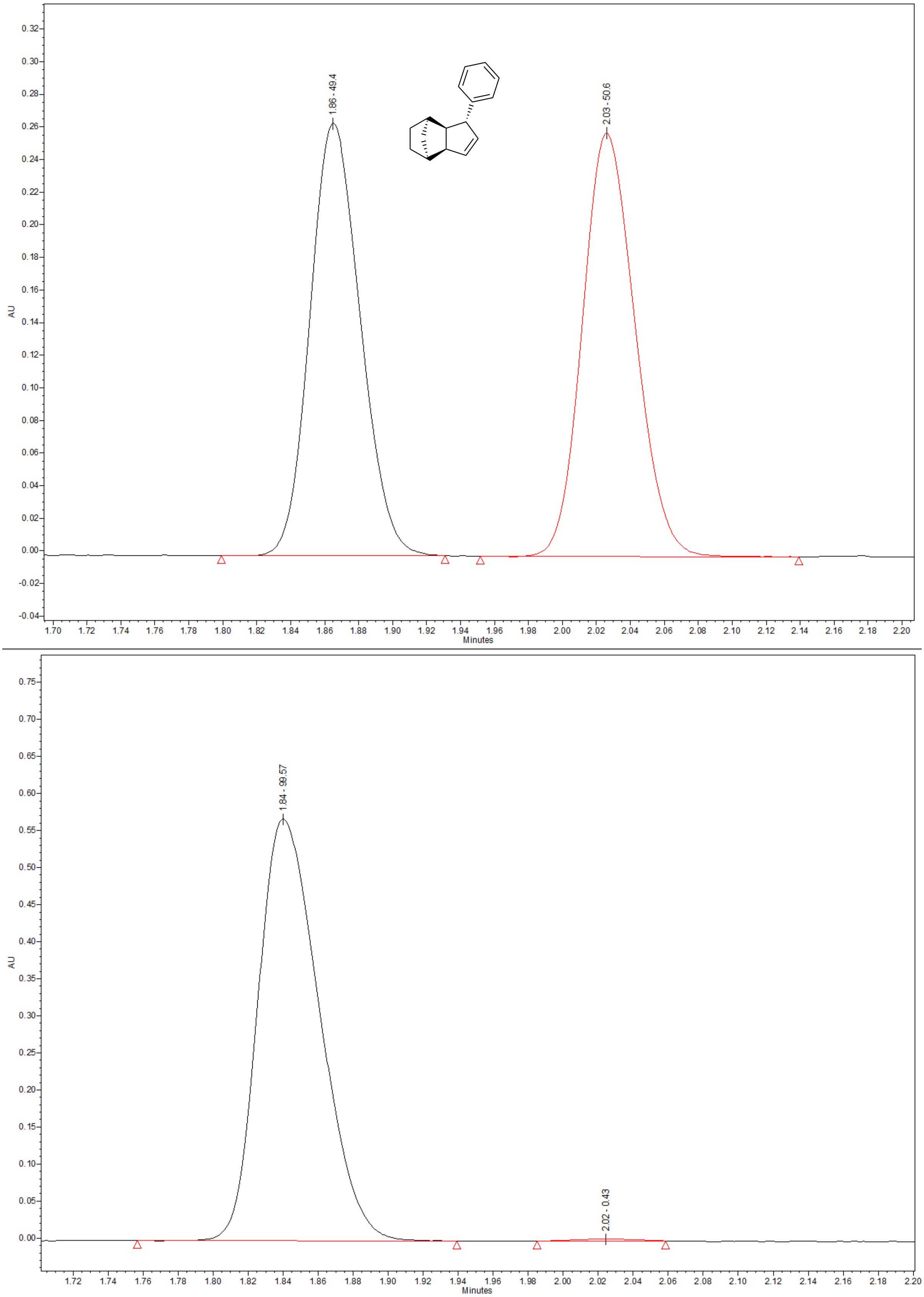

Figure S87: SFC traces of the racemic (±)-**3ba** (top) and enantioenriched (–)-**3ba** (bottom).

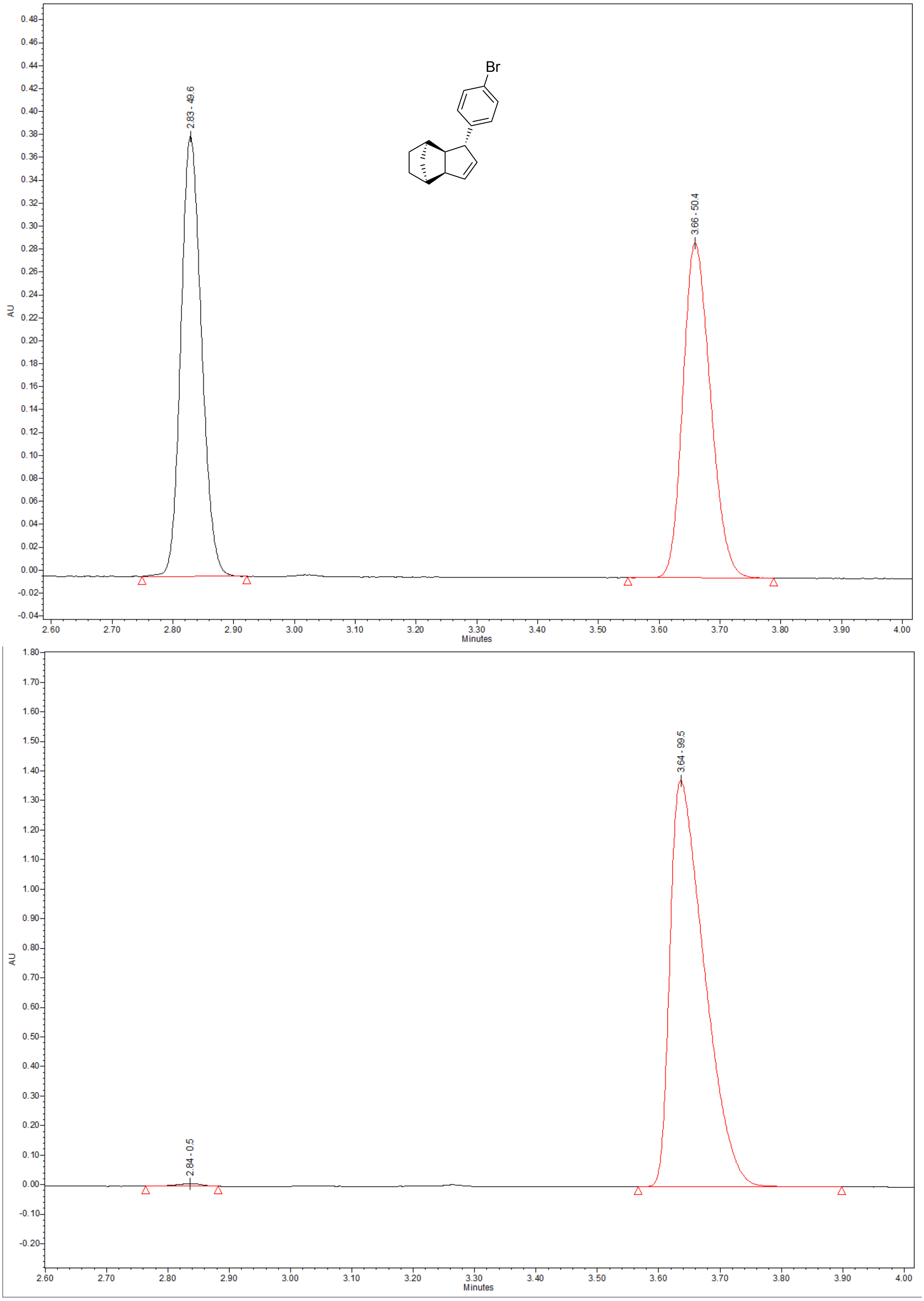

Figure S88: SFC traces of the racemic (±)-**3bb** (top) and enantioenriched (–)-**3bb** (bottom).

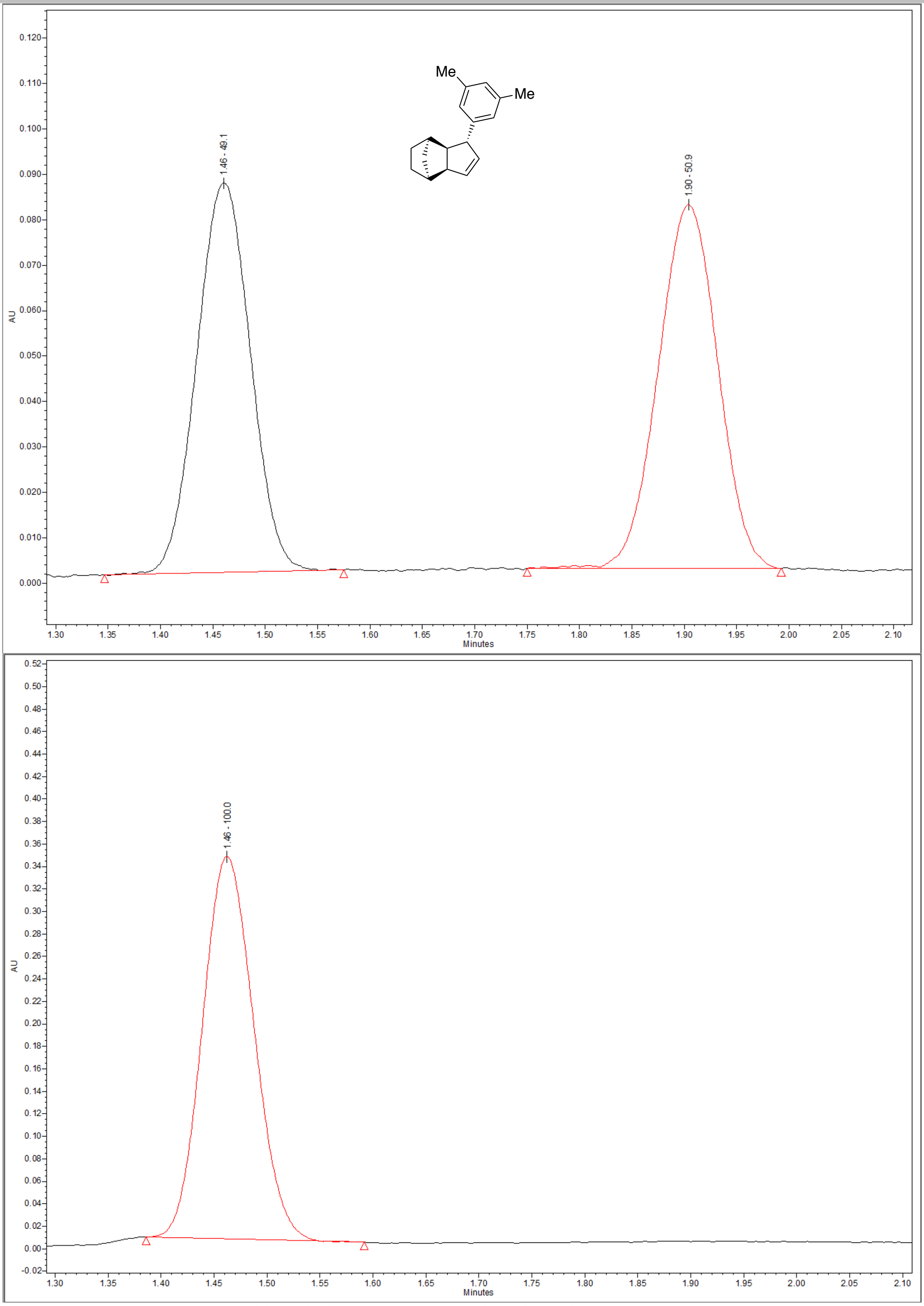

Figure S89: SFC traces of the racemic (±)-**3bc** (top) and enantioenriched (–)-**3bc** (bottom).

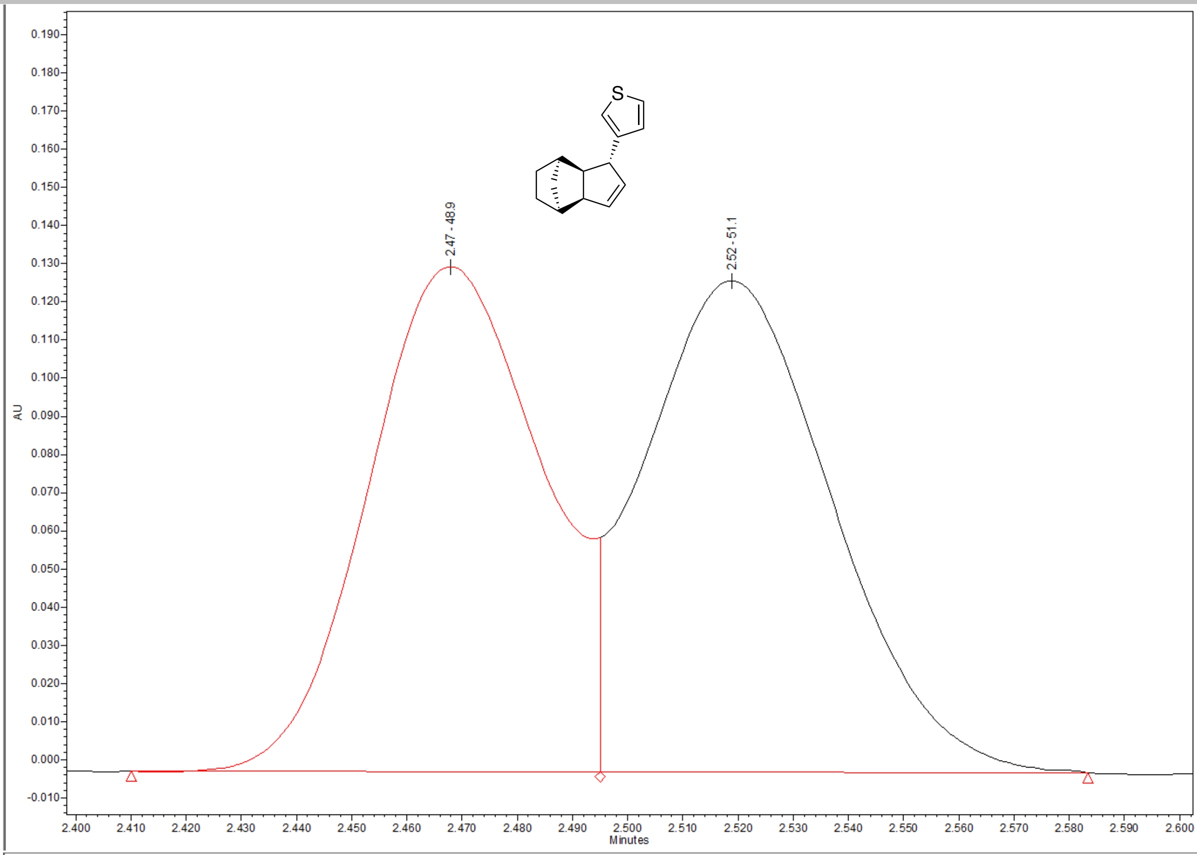

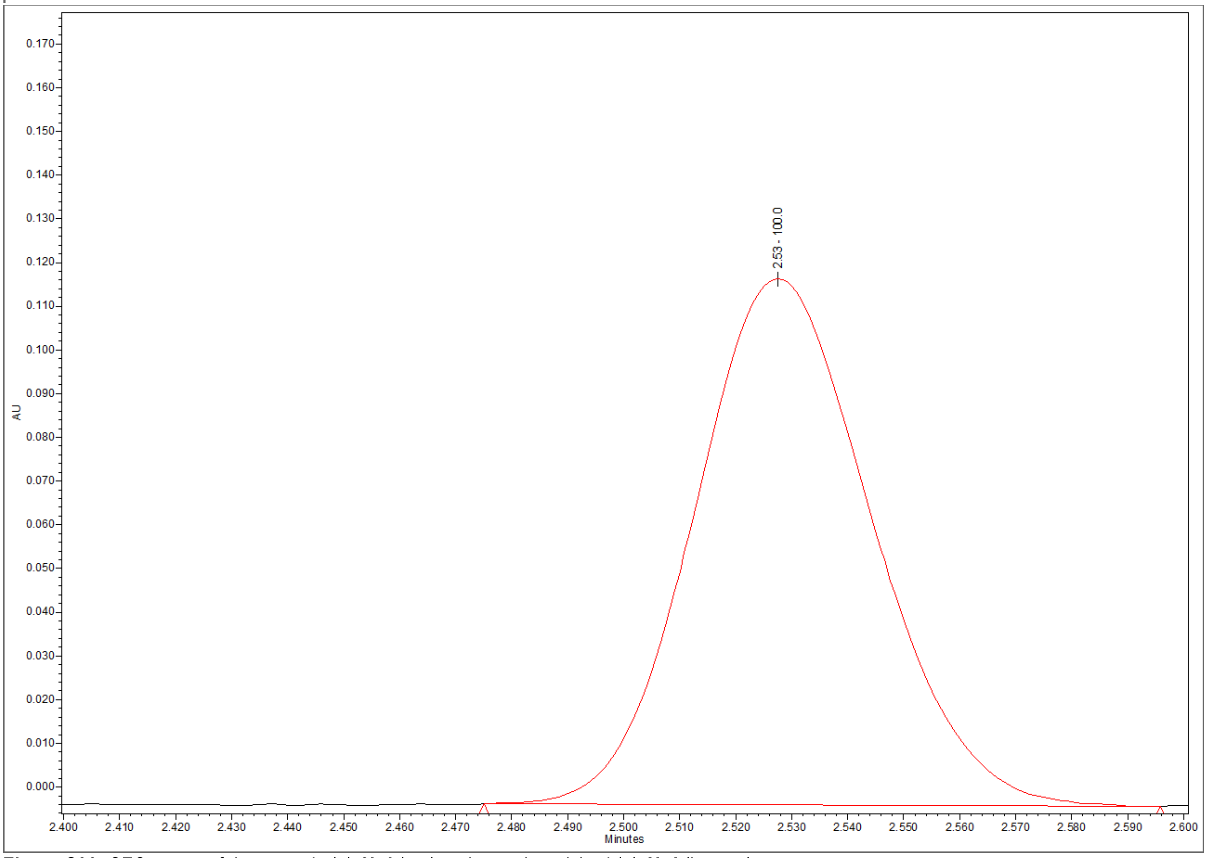

Figure S90: SFC traces of the racemic (±)-**3bd** (top) and enantioenriched (–)-**3bd** (bottom).

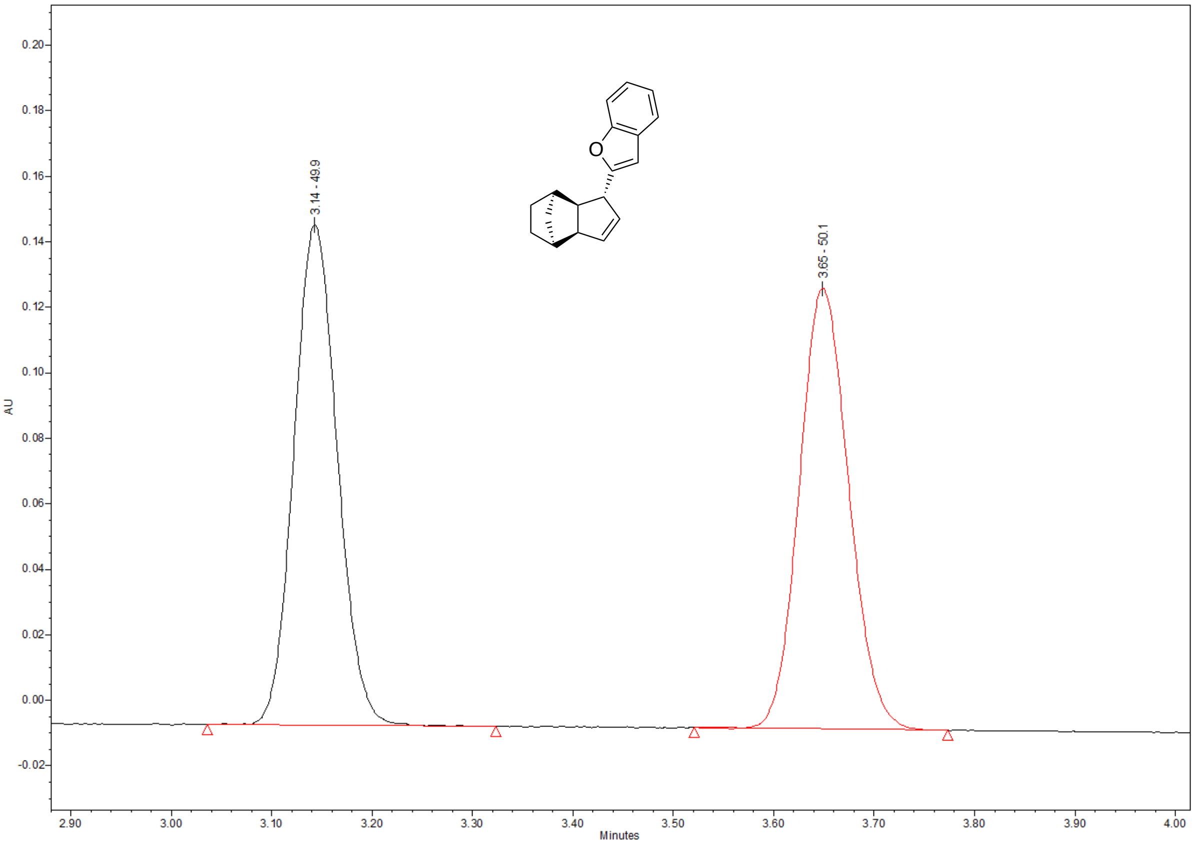

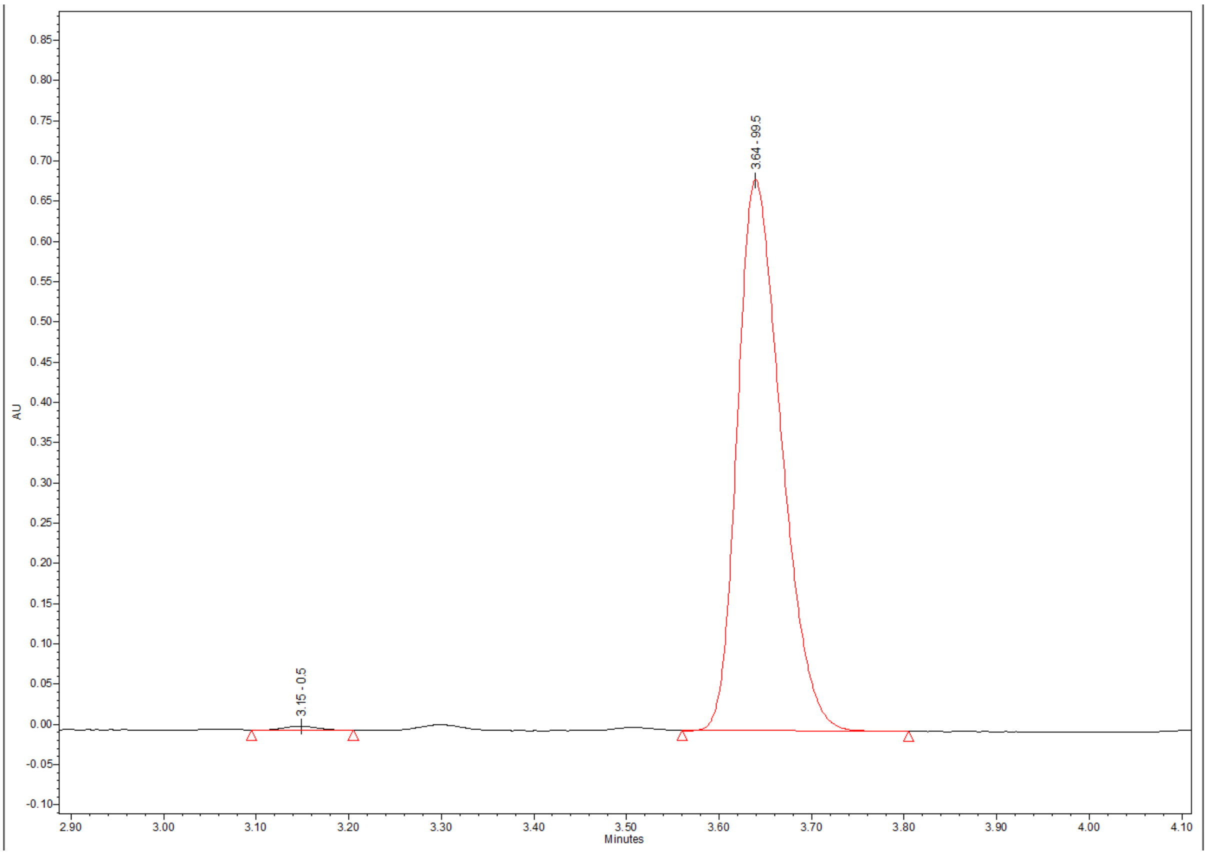

Figure S91: SFC traces of the racemic (±)-**3be** (top) and enantioenriched (–)-**3be** (bottom).

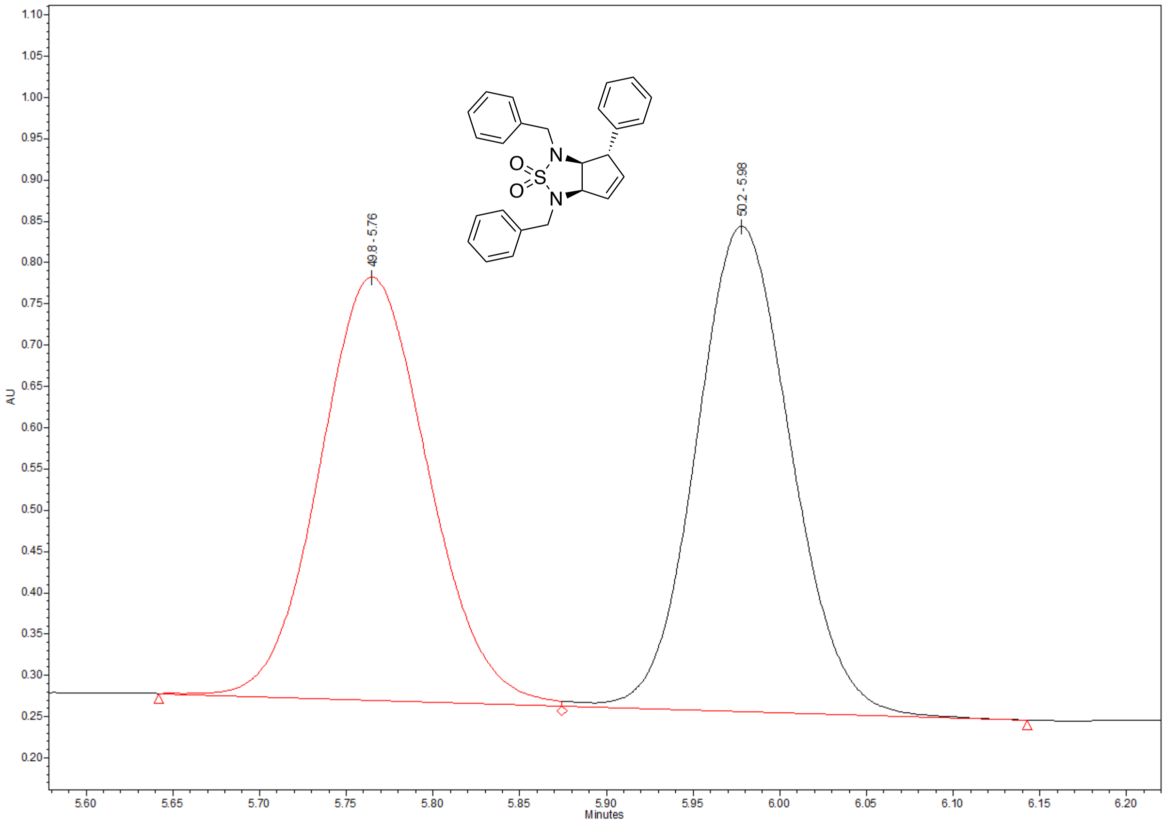

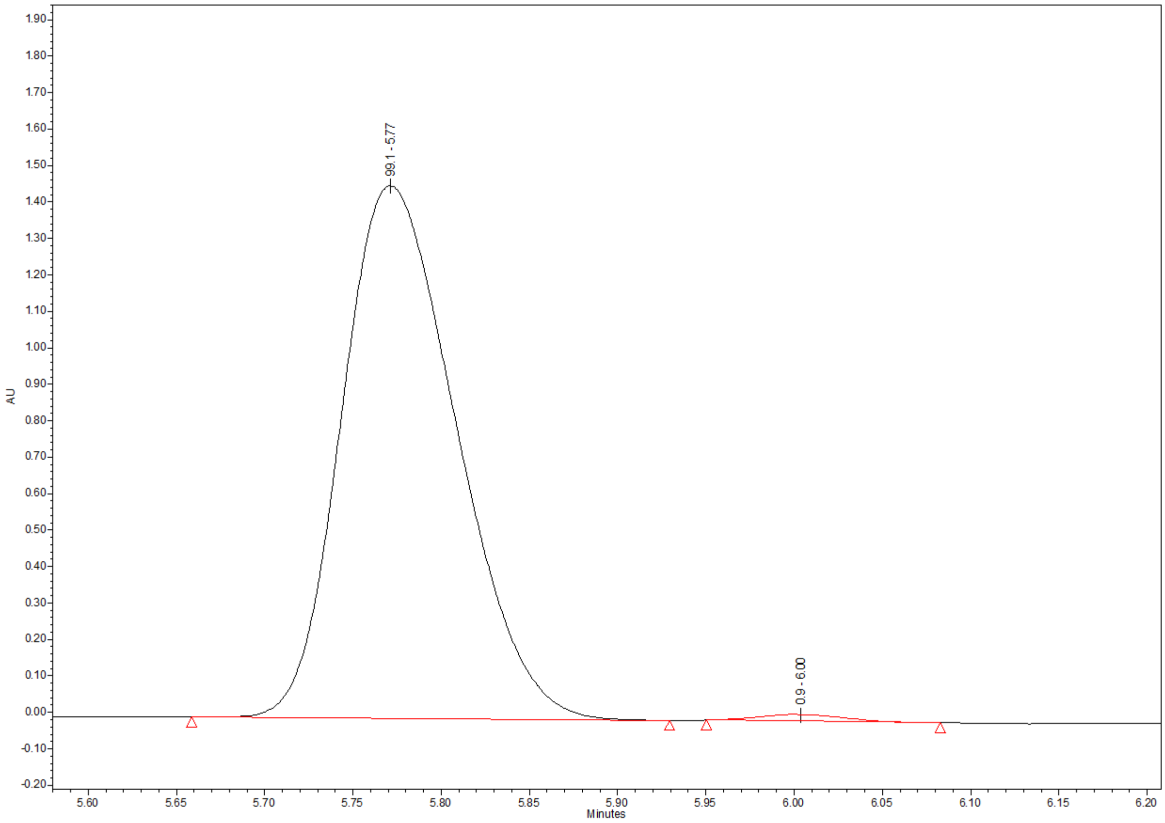

Figure S92: SFC traces of the racemic (±)-**3ca** (top) and enantioenriched (–)-**3ca** (bottom).

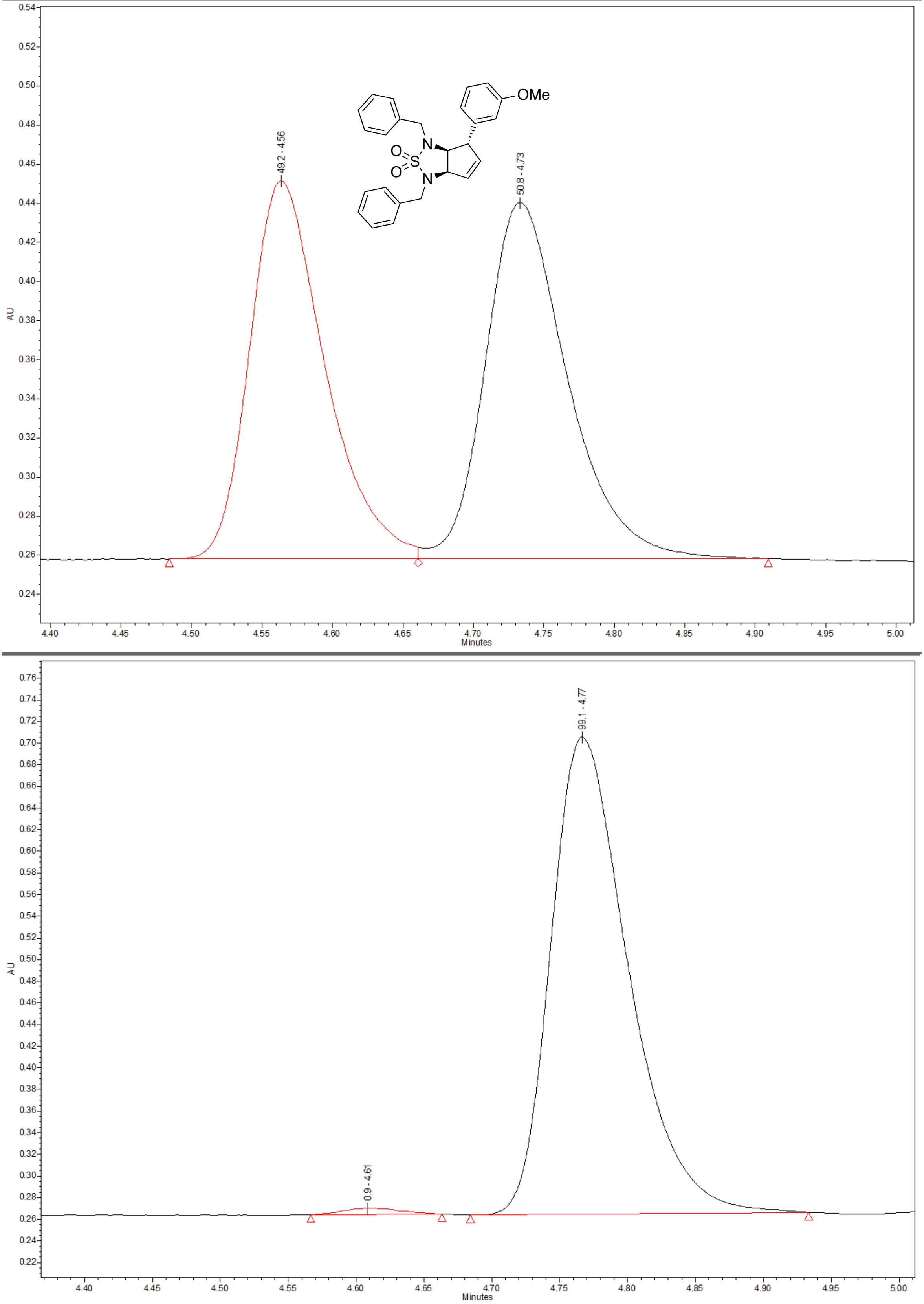

Figure S93: SFC traces of the racemic (±)-**3cb** (top) and enantioenriched (–)-**3cb** (bottom).

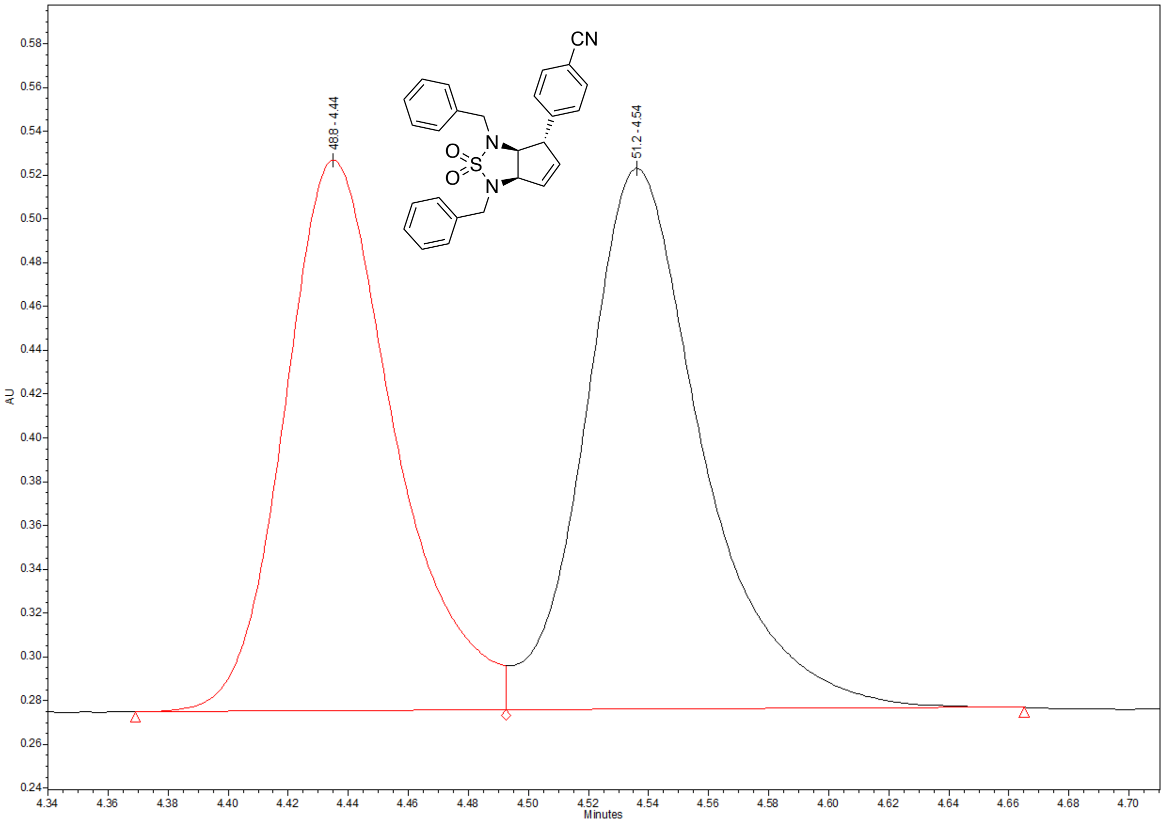

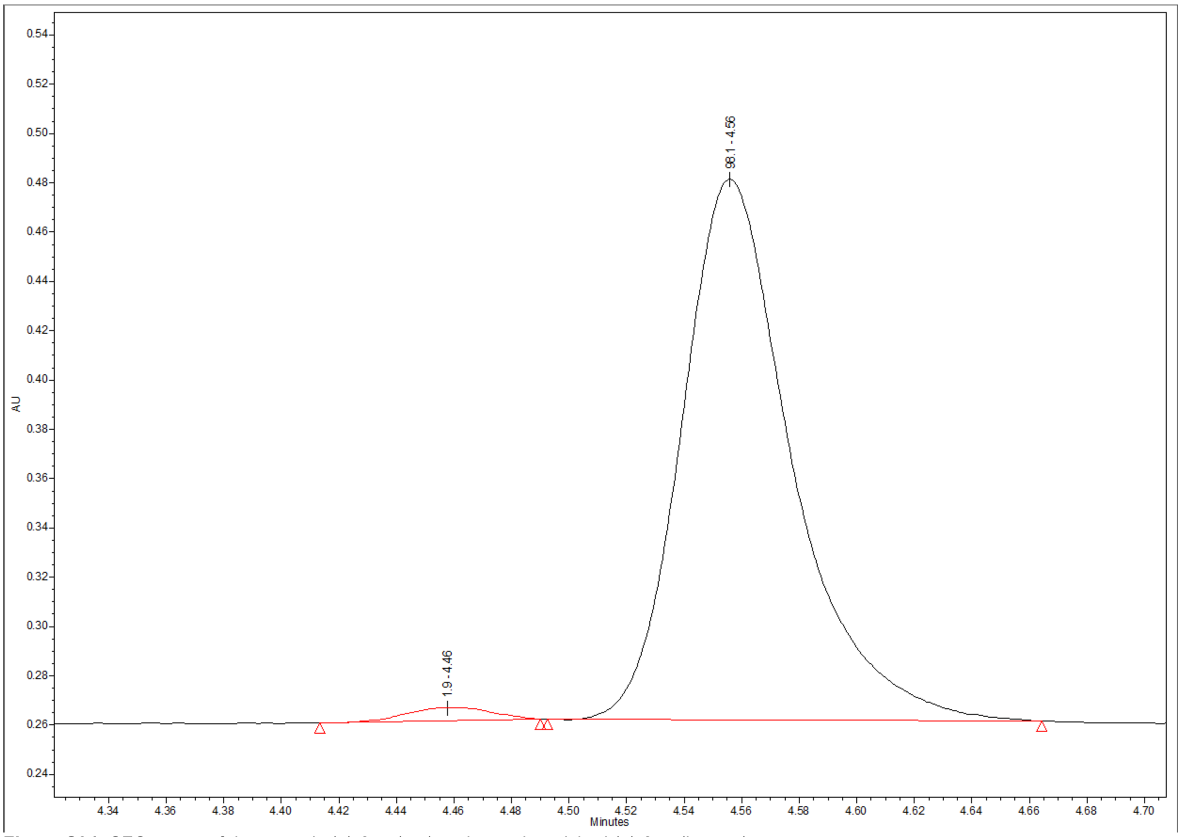

Figure S94: SFC traces of the racemic (±)-**3cc** (top) and enantioenriched (–)-**3cc** (bottom).

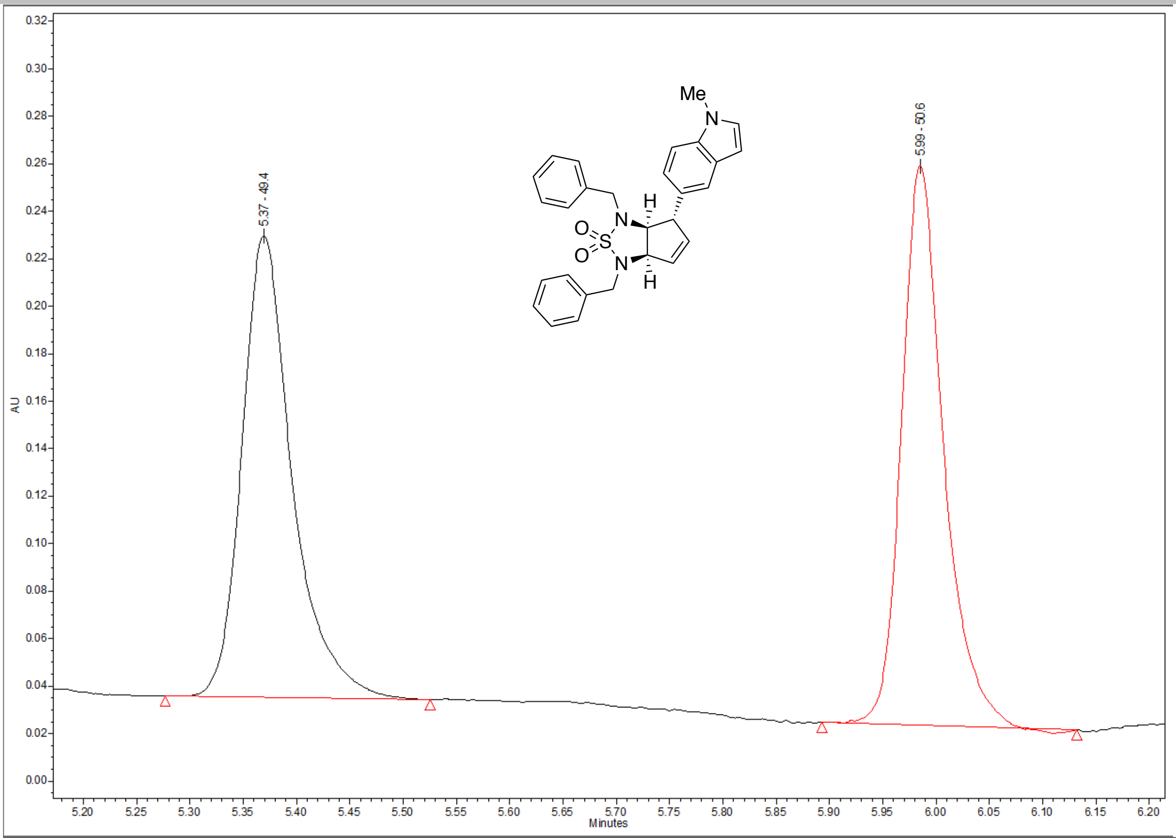

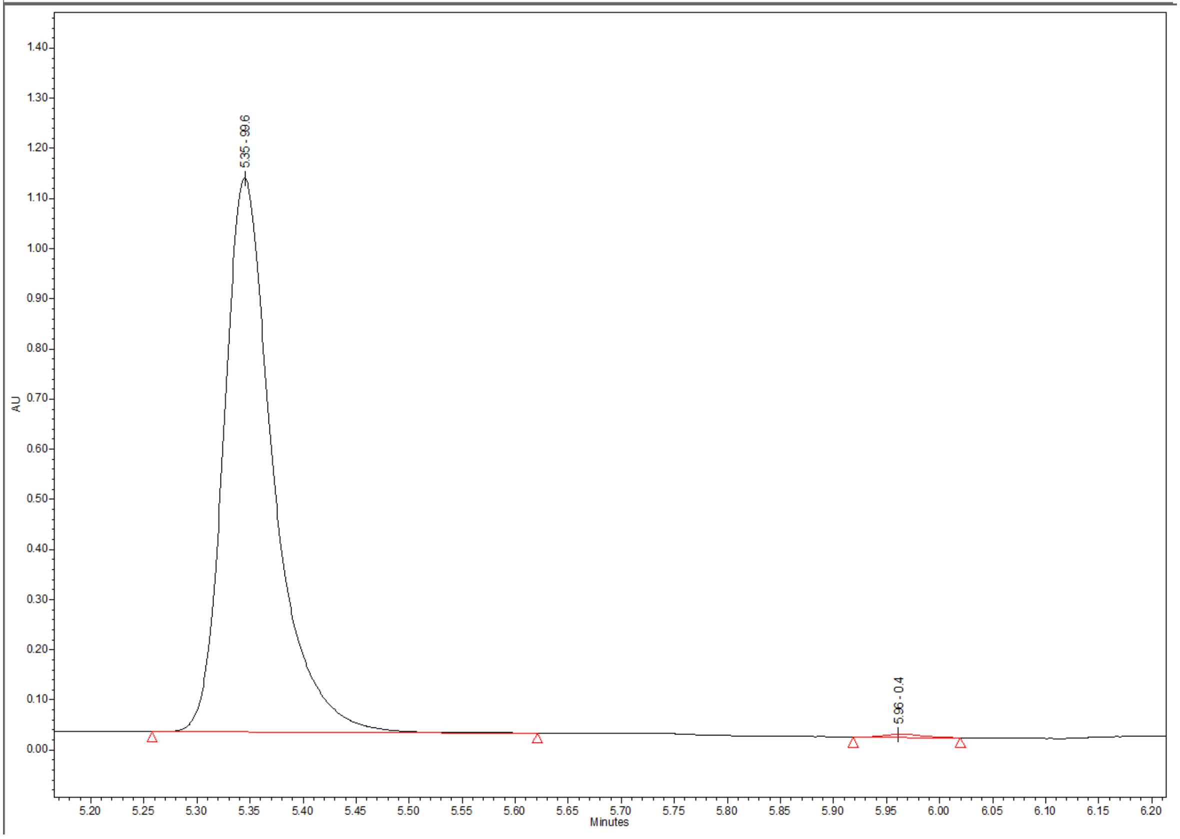

Figure S94: SFC traces of the racemic (±)-**3cd** (top) and enantioenriched (–)-**3cd** (bottom).

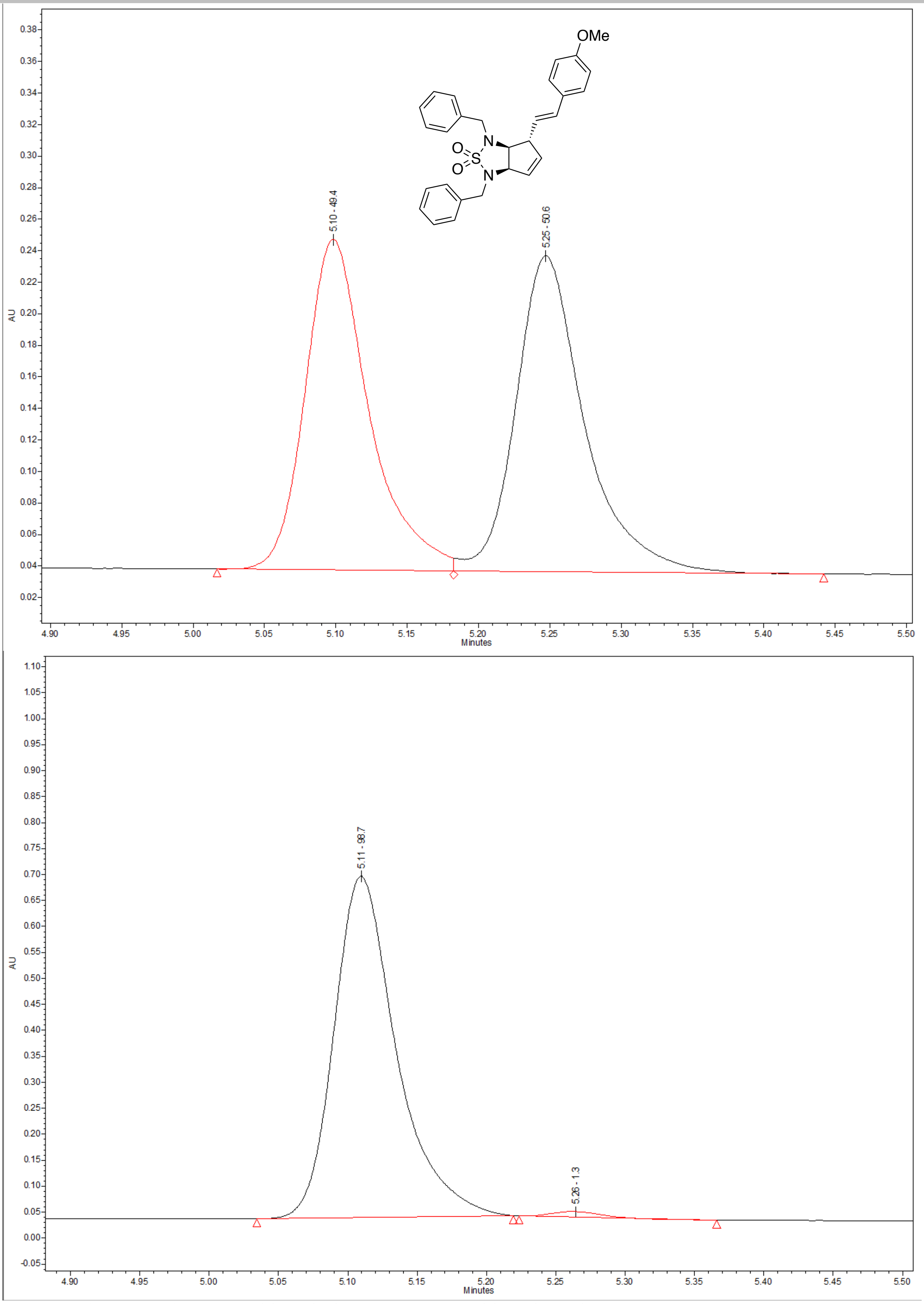

Figure S95: SFC traces of the racemic (±)-**3ce** (top) and enantioenriched (–)-**3ce** (bottom).

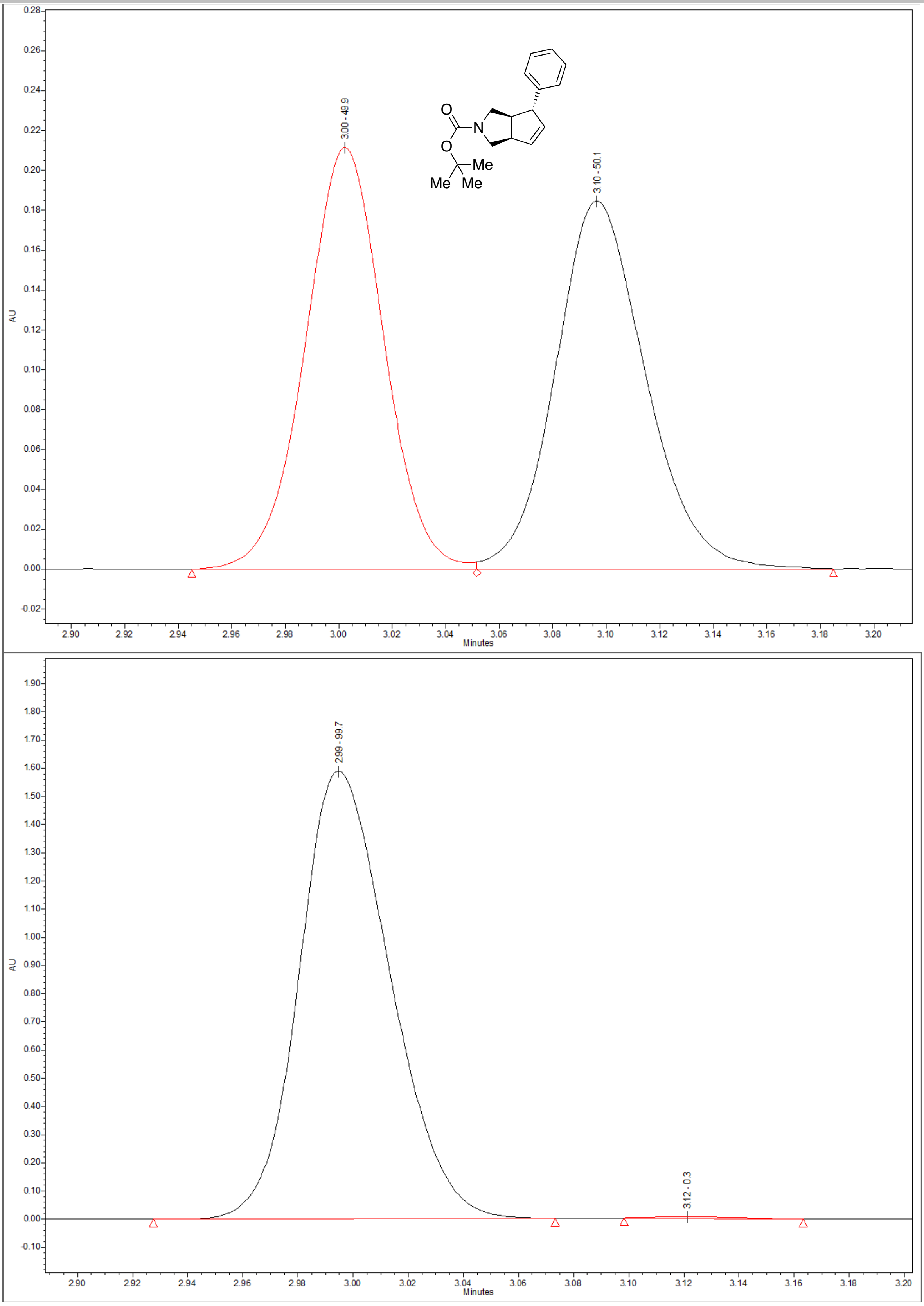

Figure S96: SFC traces of the racemic (±)-**3da** (top) and enantioenriched (–)-**3da** (bottom).

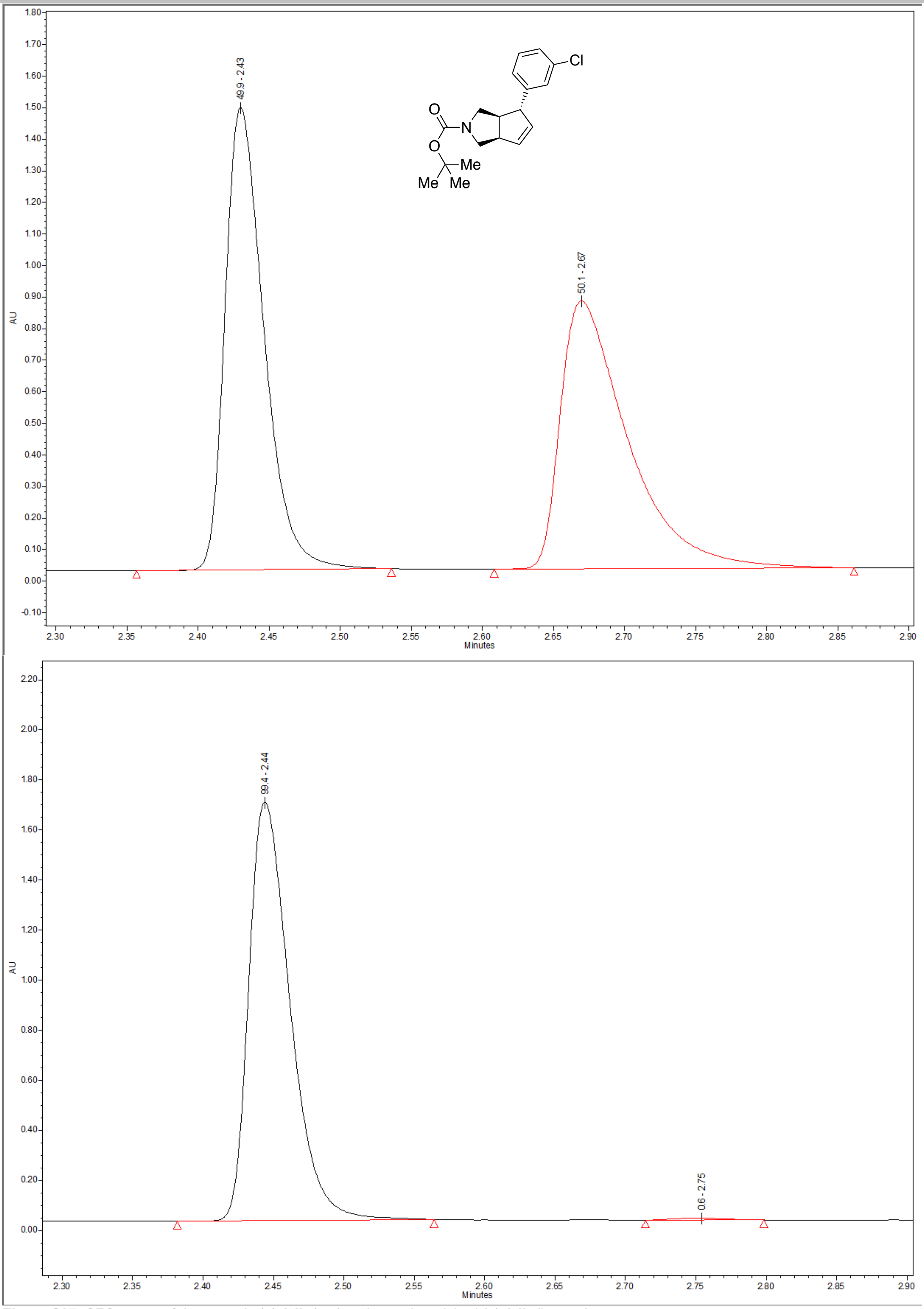

Figure S97: SFC traces of the racemic (±)-**3db** (top) and enantioenriched (–)-**3db** (bottom).

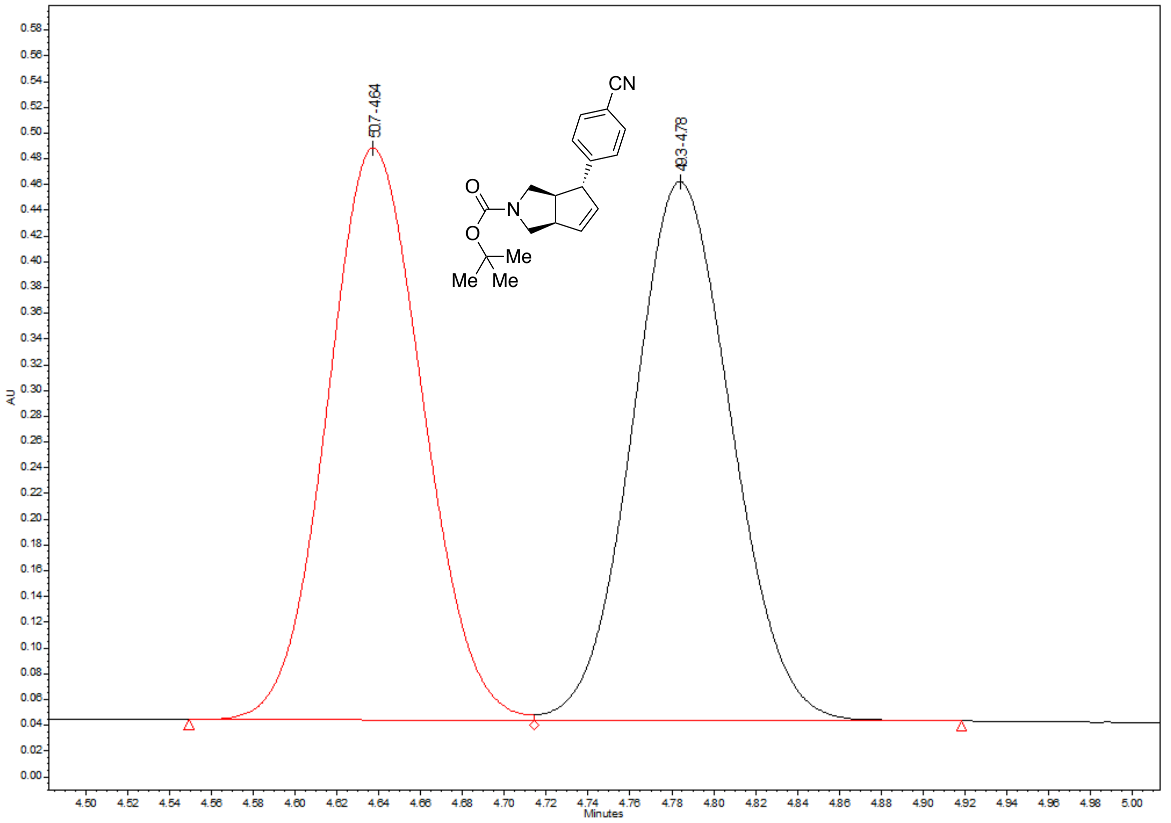

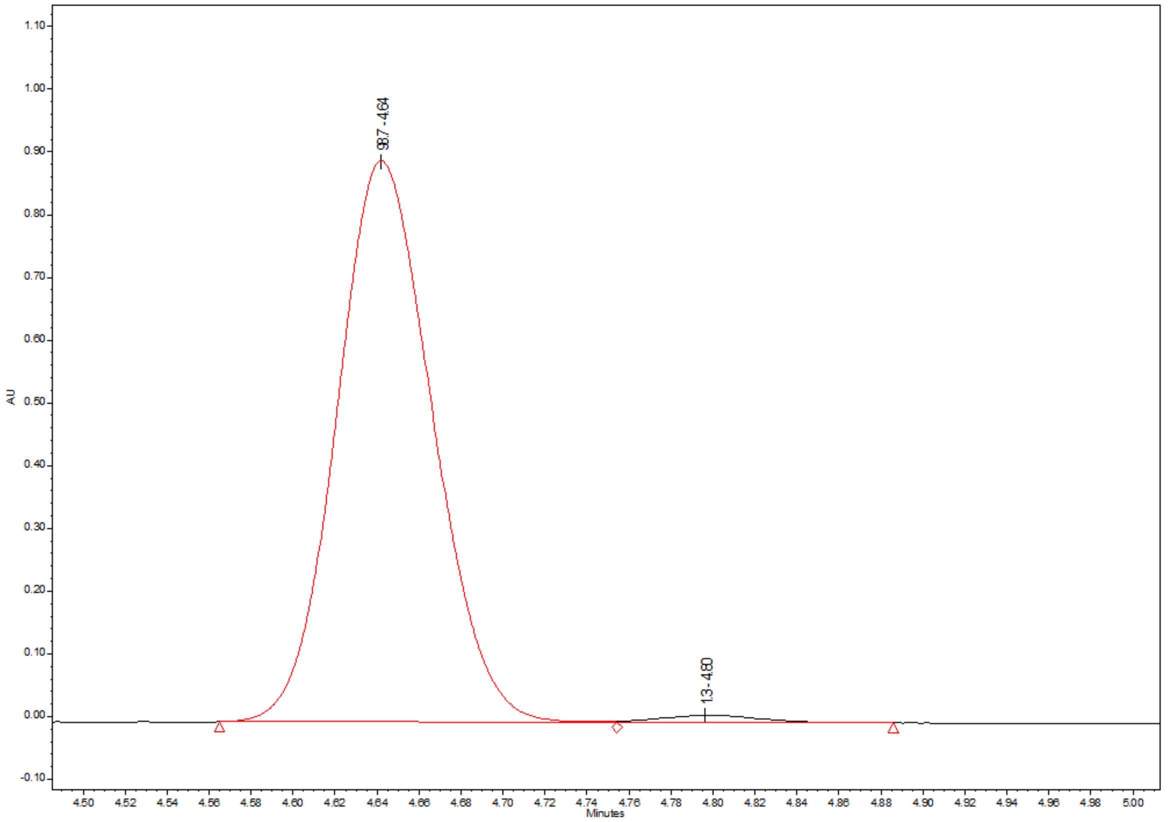

Figure S98: SFC traces of the racemic (±)-**3dc** (top) and enantioenriched (–)-**3dc** (bottom).

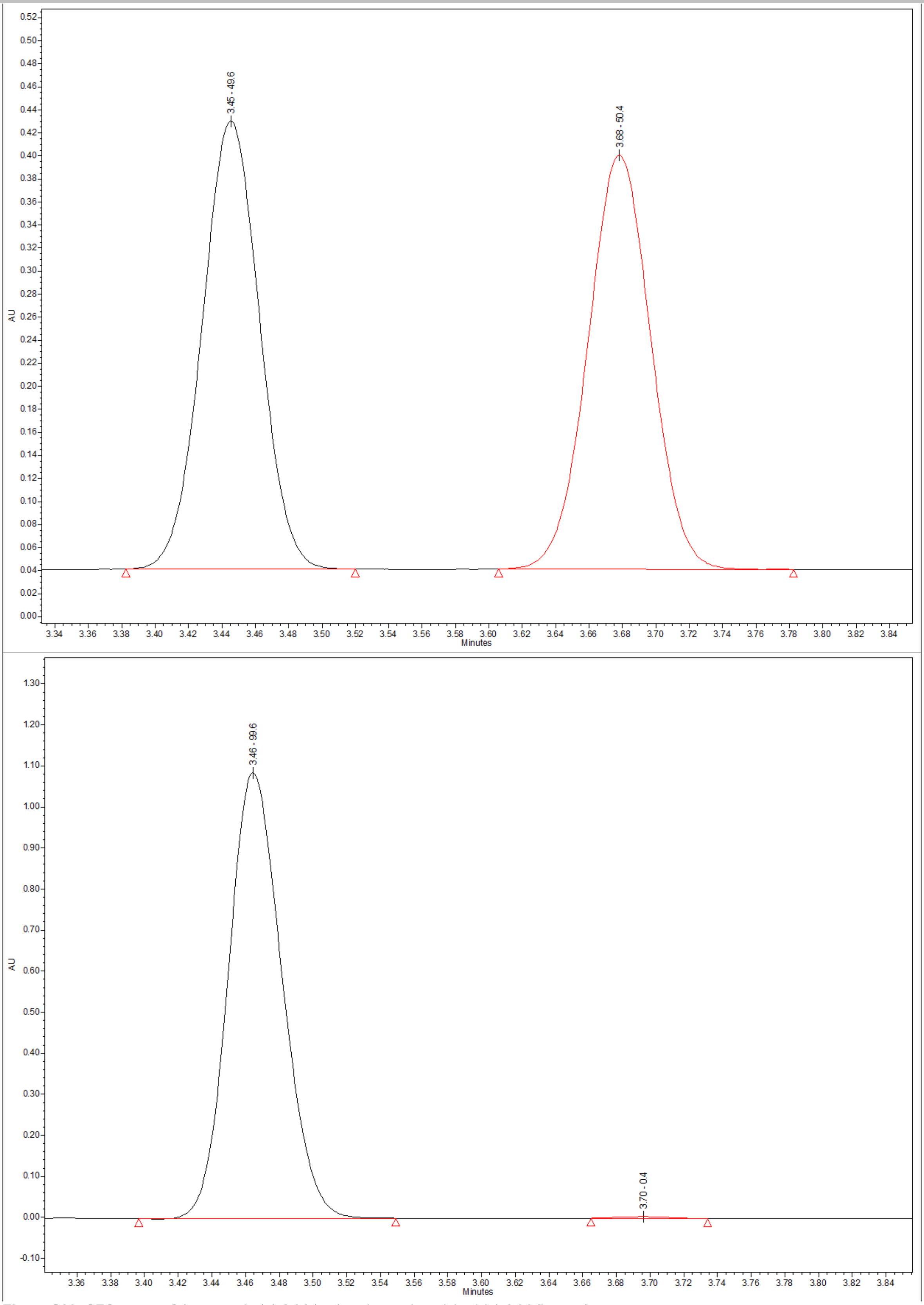

Figure S99: SFC traces of the racemic (±)-**3dd** (top) and enantioenriched (–)-**3dd** (bottom).

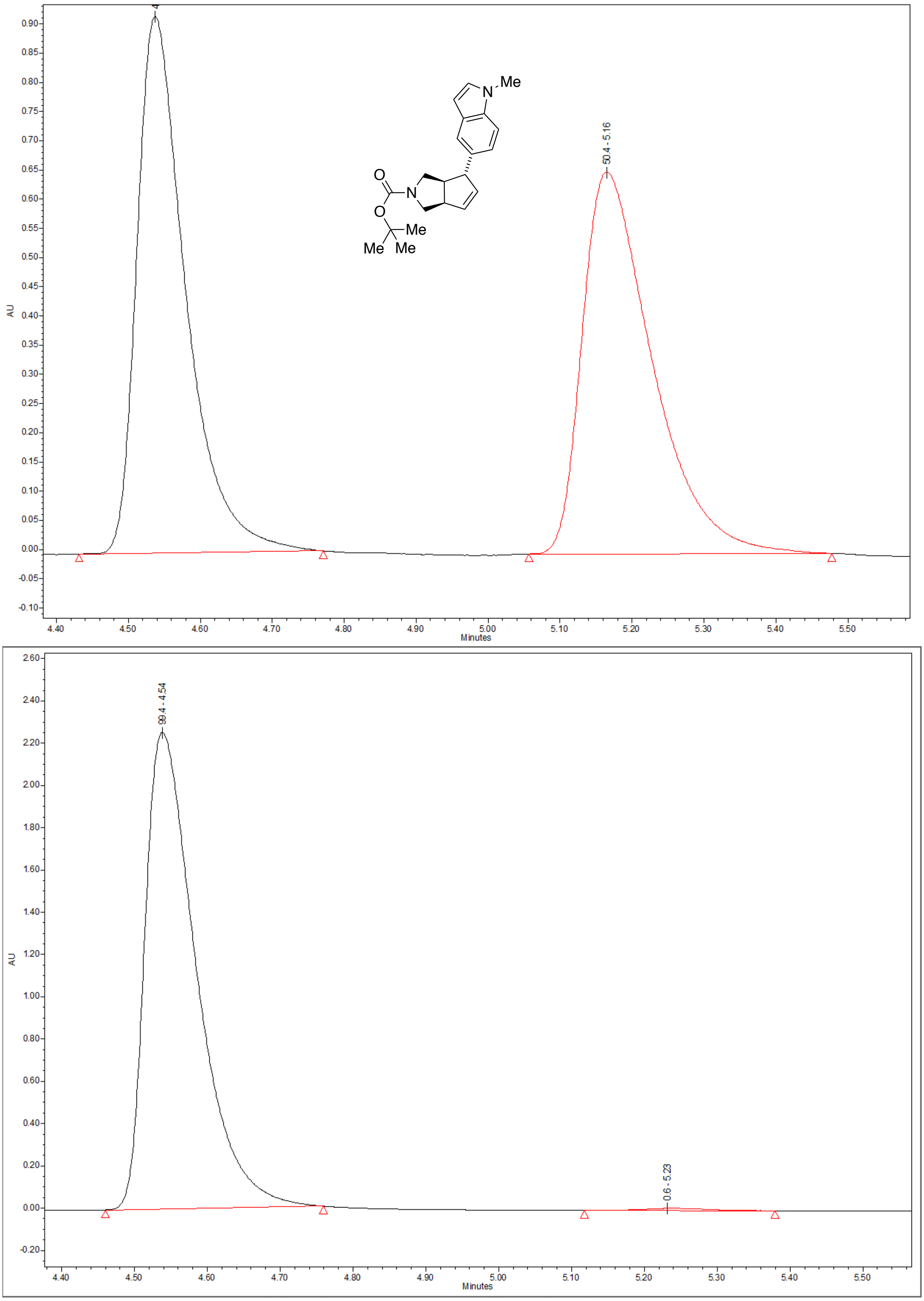

Figure S100: SFC traces of the racemic (±)-**3de** (top) and enantioenriched (–)-**3de** (bottom).

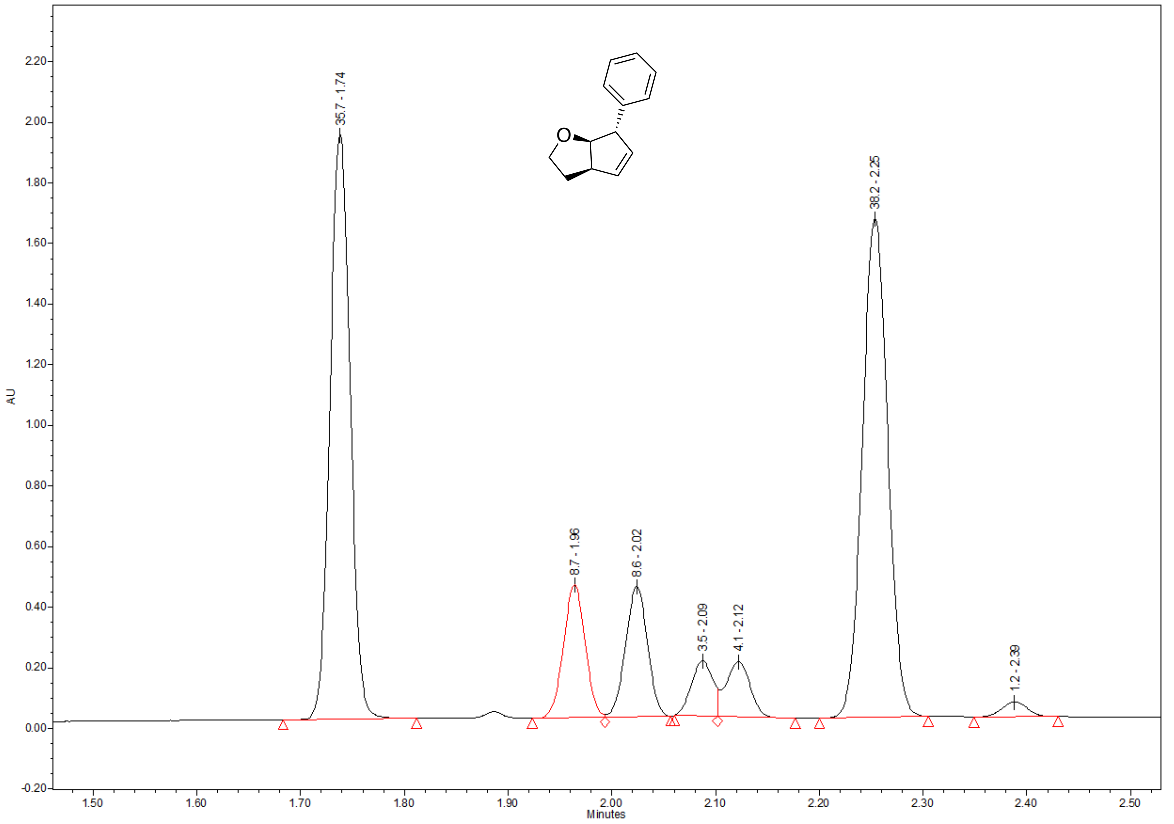

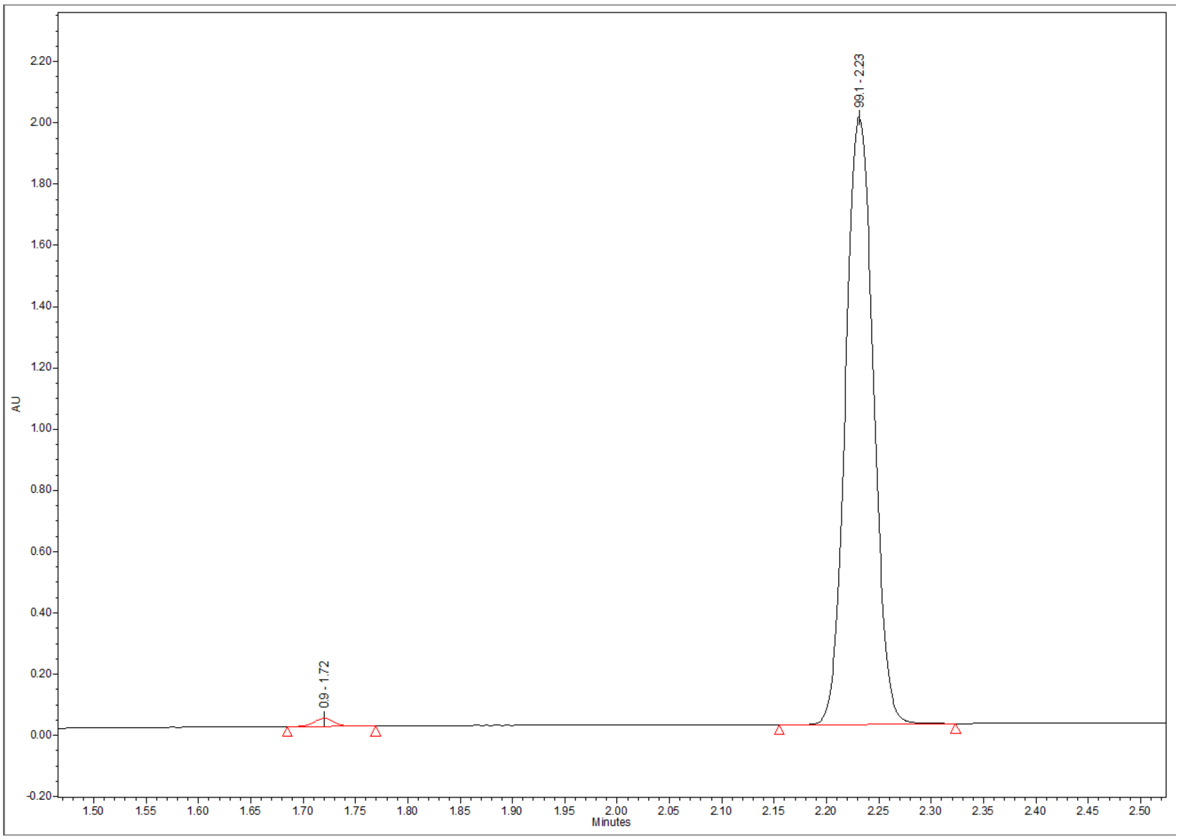

Figure S101: SFC traces of the racemic (±)-**3ea*** and (±)-**3ea** (top) and enantioenriched (–)-**3ea*** (bottom).

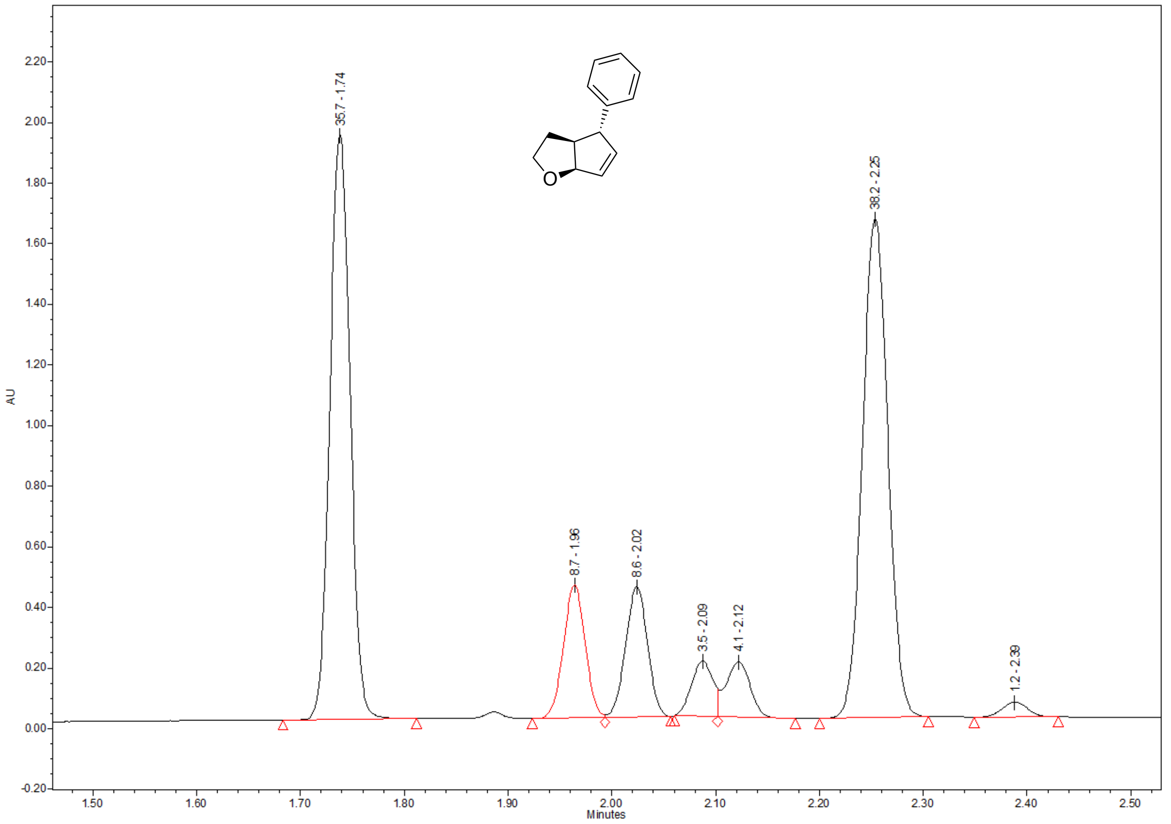

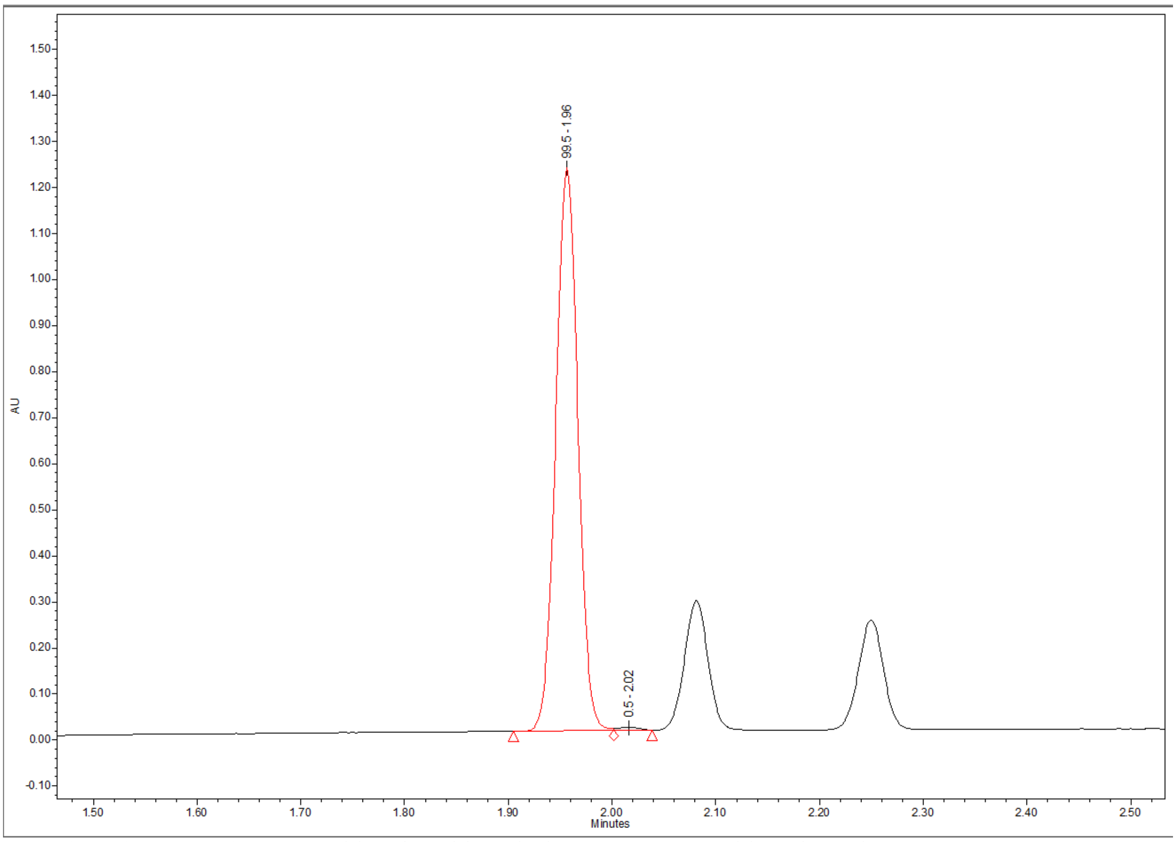

Figure S102: SFC traces of the racemic (±)-**3ea*** and (±)-**3ea** (top) and enantioenriched (–)-**3ea** (bottom).

Author Contributions

F. W G. and S. P. F. planned and designed the study. F. W. G. performed all experiments and S. P. F. and M. M. guided the research. F. W G. and S. P. F. analyzed data and wrote the manuscript.
